# Fluorescent Molecular Cages with Sucrose and Cyclotriveratrylene Units for the Selective Recognition of Choline and Acetylcholine

**DOI:** 10.1021/acs.joc.1c00019

**Published:** 2021-03-12

**Authors:** Łukasz Szyszka, Marcin Górecki, Piotr Cmoch, Sławomir Jarosz

**Affiliations:** Institute of Organic Chemistry, Polish Academy of Sciences, Kasprzaka 44/52, Warsaw 01-224, Poland

## Abstract

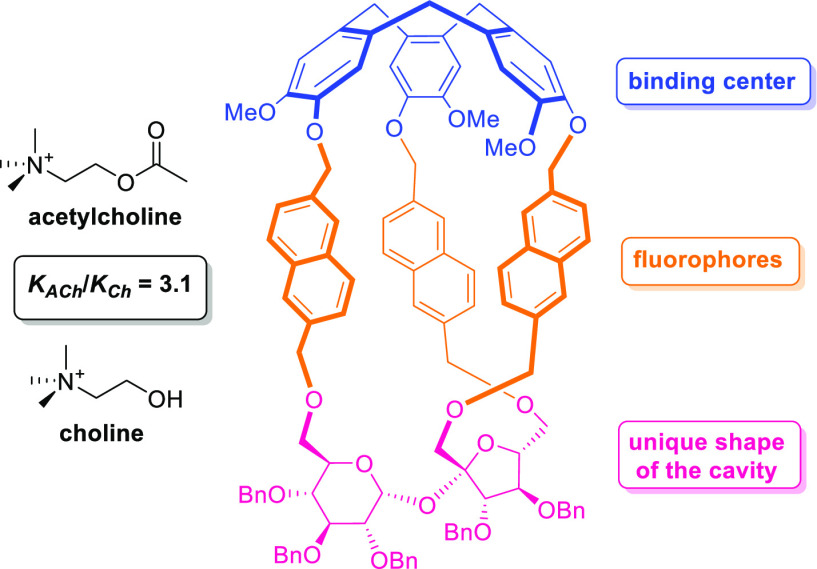

The synthesis of four fluorescent diastereoisomeric molecular cages containing cyclotriveratrylene and sucrose moieties connected *via* the naphthalene linkers is reported. These diastereoisomers were found to be selective and efficient receptors for acetylcholine and choline. Compound ***P*-5a** has a better affinity for choline over acetylcholine, while cage ***M*-5a** exhibits a higher association constant for acetylcholine over choline. The highest selectivity value was observed for compound ***M*-5a** (*K*_ACh_/*K*_Ch_ = 3.1). Cages ***P*-5a**, ***P*-5b**, ***M*-5a**, and ***M*-5b** were fully characterized by the advanced NMR techniques, and ECD spectroscopy was supported by DFT calculations. The binding constants *K*_a_ of these receptors were determined by fluorescence titration experiments in acetonitrile.

## Introduction

Molecular cages with fluorescent properties able to selectively recognize various biologically essential compounds have been recently extensively studied.^1^ Fluorescence imaging techniques are attractive and powerful tools for the nondestructive visualization of biological processes with high spatial resolution.^2^

The main advantages of fluorescence recognition studies are high sensitivity, fast response time, and technical simplicity, which makes this technique a useful tool for analytical detections and optical imaging.^3^ In the last decades, several macrocycle derivatives containing fluorophores with different geometries and cavities capable of encapsulating guest molecules have been reported.^4^

The synthesis of molecular cages is particularly attractive due to its selective recognition properties.^5^ These receptors can find application as catalysts,^6^ separators,^7^ sensors,^8^ porous materials,^9^ polymers,^10^ transporters,^11^ or drug delivery systems.^12^ Among them, cryptophanes and hemicryptophanes are of particular interest as they are able to recognize small organic compounds.^13^ This class of receptors is based on the rigid and bowl-shaped C_3_-symmetrical cyclotriveratrylene (CTV) unit.^14^

The significant contribution to the synthesis of the CTV-based cages was made by Martinez group.^15^ They obtained a wide range of molecular cages in which the CTV moiety is triply connected with tris(2-aminoethyl)amine or 1,3,5-tris(bromomethyl)benzene *via* different linkers. These synthetic receptors can selectively recognize carbohydrates,^16^ zwitterions,^17^ or neurotransmitters.^18^

Although chiral receptors are important in selective recognition, only a few examples of such derivatives have been reported so far due to the difficulties in their syntheses.^19^ Preparation of chiral macrocyclic receptors usually requires a multistep procedure, and the final yield is generally low for both steric and entropic reasons. The vast majority of these compounds are prepared as racemic mixtures.^15^

Acetylcholine (ACh) and choline (Ch), structurally related biologically important compounds, are the subject of interest for many years.^20^ Acetylcholine plays a crucial role in the human central nervous system, in particular, in memory processes and transmission of the nervous impulse. This neurotransmitter, released at nerve-muscle synapse, is hydrolyzed to acetic acid and choline by acetylcholinesterase to prevent its high concentrations in the synaptic cleft.^21^ Several diseases are connected with cholinergic failures, such as Parkinson’s disease, Alzheimer’s disease, Schizophrenia, or other mental diseases.^22^ Choline (Ch) is an essential nutrient and has a critical role in neurotransmitter function because of its impact on acetylcholine synthesis and dopaminergic function.^23^ Thus, the selective differentiation of both compounds could provide the understanding of the mechanism of the transmission of nervous signals.

In the last decade, several fluorescent receptors able to recognize ACh and Ch have been reported.,^20a^^24^ Martinez *et al.* obtained three fluorescent hemicryptophanes containing naphthalene^25^ or phenylacetylene^26^ linkers, which can efficiently distinguish ACh over Ch. In another paper, they reported a fluorescent heteroditopic host with the naphthalene units and a Zn(II) complex for the selective recognition of choline phosphate.^27^ Wu *et al.* reported a self-assembled triple anion helicate acting as a fluorescence displacement sensor, able to differentiate effectively choline, acetylcholine, glycine betaine, and l-carnitine.^28^ In contrast to Martinez’s hemicrypotophanes, this supramolecular host system displays high selectivity toward Ch over ACh. Sarmentero and Ballester developed a fluorescent hybrid cavitand-resorcin[4]arene receptor with the pH-modulated binding properties toward choline.^29^ Moreover, this receptor is able to form thermodynamically stable complexes with complementary ammonium cations in protic solvents.

For many years, our group is involved in the synthesis of macrocyclic derivatives with sucrose scaffold able to recognize chiral and achiral guests.^30^ We have prepared a vast array of chiral receptors based on this disaccharide that could effectively complex ammonium salts,^31^ amino acid esters,^32^ or simple anions.^33^

**Figure 1:**
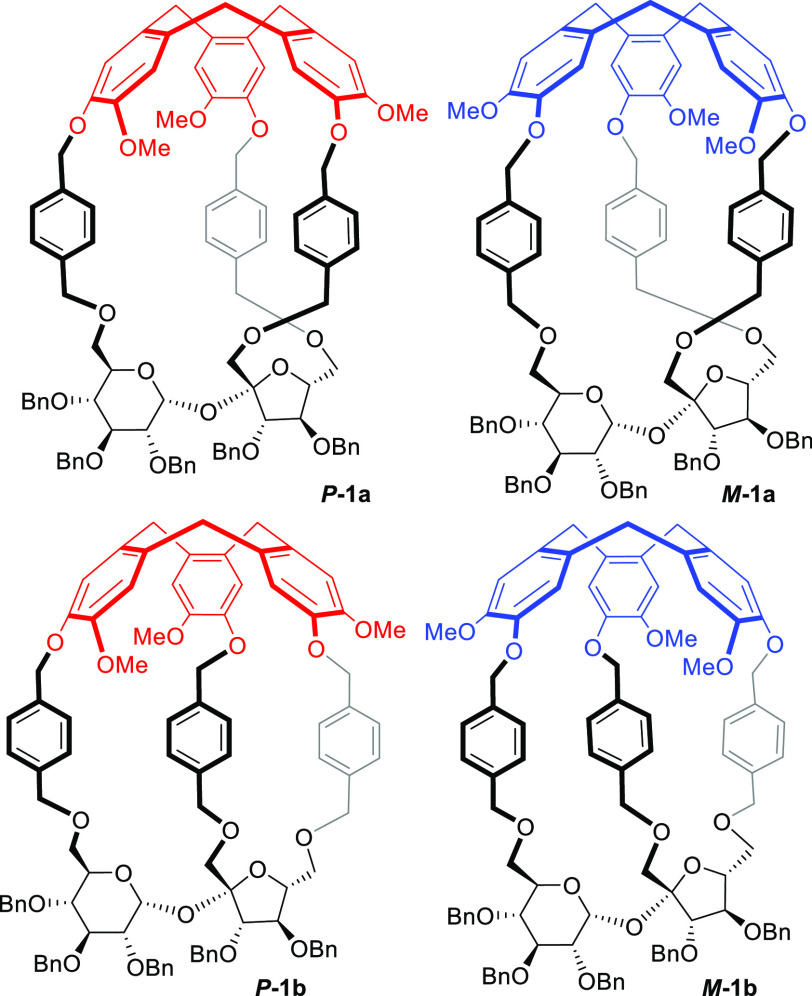
Structures of four molecular cages ***P*-1a**, ***M*-1a**, ***P*-1b**, and ***M*-1b** based on CTV and sucrose moieties connected *via p*-phenylene linkers.

Our current studies are concentrated on chiral molecular cages bearing CTV and sucrose scaffolds connected *via* different linkers. In 2019, we presented, for the first time, the water-soluble chiral molecular cages consisting of cyclotriveratrylene and sucrose units.^34^ Recently, we demonstrated an efficient, short, and high-yield route to four diastereoisomeric molecular cages ***P*-1a**, ***M*-1a**, ***P*-1b**, and ***M*-1b** connected *via* the *p*-phenylene linkers (Figure 1).^35^ These compounds, unfortunately, are not able to recognize choline or acetylcholine.

Herein, we report the synthesis of fluorescent chiral CTV-sucrose-based cages with the naphthalene linkers and disclose their recognition properties toward choline and acetylcholine.

We decided to combine (i) a CTV unit as a binding center for an ammonium part of neurotransmitters, (ii) a sucrose unit as a chiral scaffold, which provide a unique shape of the cavity, and (iii) the naphthalene linkers as fluorophores, which will ensure the fluorescence properties, rigid cavity, and additional π-system for supporting the recognition.

## Results and Discussion

**Scheme 1:**
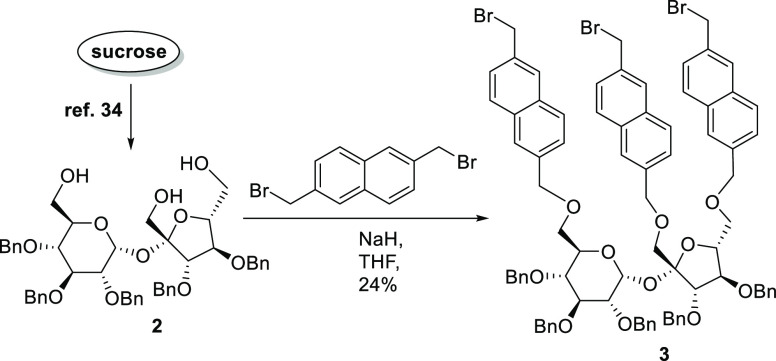
Synthesis of Sucrose Tribromide **3**

The synthesis of the CTV-sucrose-based cages was initiated from commercial sucrose, which was transformed into triol **2** in a three-step route, consisting of selective protection of secondary hydroxyl groups, according to our previously reported procedure.^34^ Alkylation of this triol with an excess of 2,6-bis(bromomethyl)naphthalene at room temperature gave tribromide **3** in 24% yield (Scheme 1). When this reaction was carried out at reflux, the decomposition of the main product **3** was observed. Racemic cyclotriguaiacylene (**4**) was synthesized according to the previously reported literature procedure.^34^

**Scheme 2:**
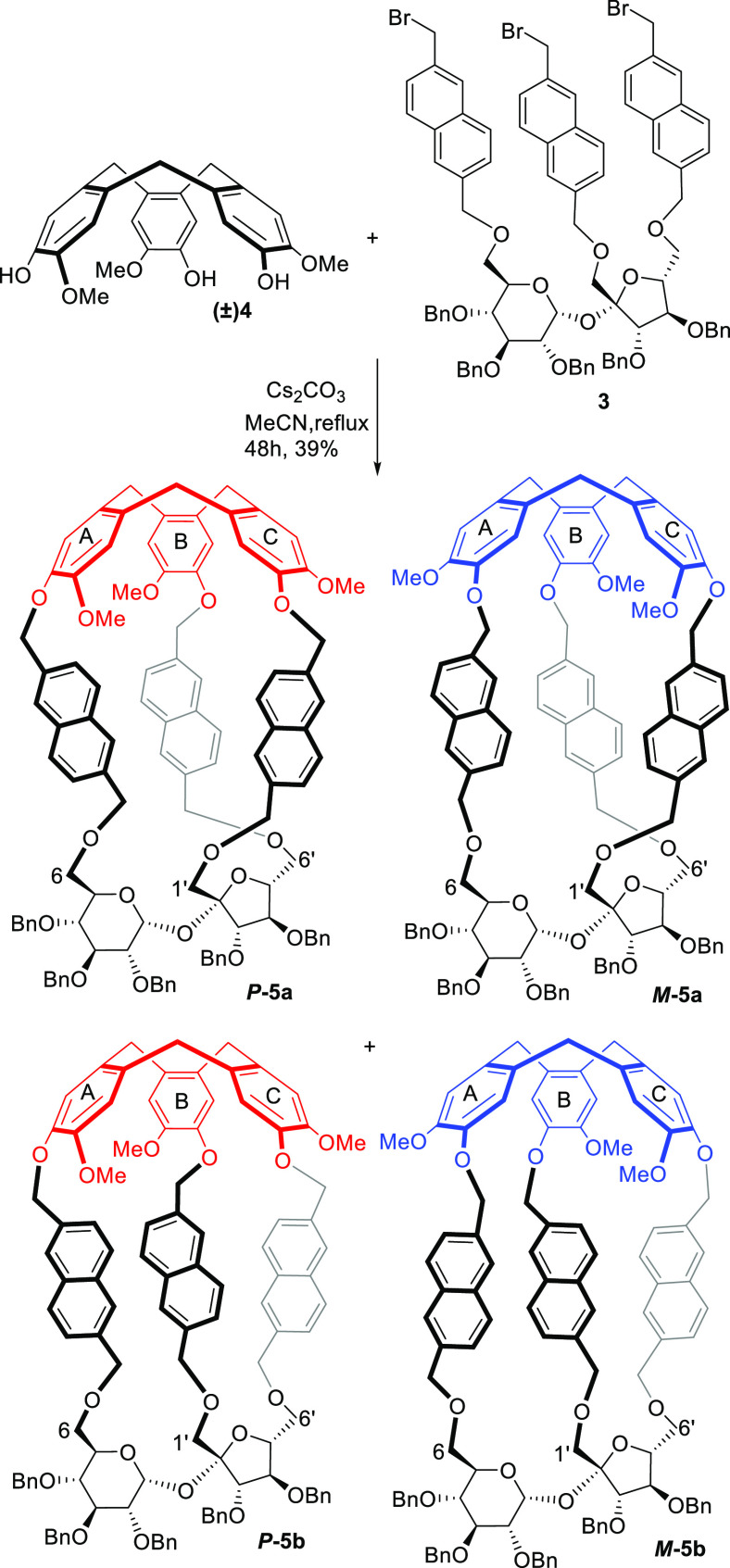
Syntheses of Four Diastereoisomeric Molecular Cages ***P*-5a**, ***M*-5a**, ***P*-5b**, and ***M*-5b**

The Cs_2_CO_3_-catalyzed macrocyclization of sucrose tribromide **3** with racemic cyclotriguaiacylene (**4**) in acetonitrile at very low concentration (*c* = 0.001 M) at reflux provided four diastereoisomeric molecular cages: ***P*-5a**, ***M*-5a**, ***P*-5b**, and ***M*-5b** in 13, 13, 6, and 7% yield, respectively (total yield of macrocyclization reaction: 39%, ratio 2:2:1:1) (Scheme 2). These diastereoisomers were successfully separated by preparative HPLC using as the eluent, a mixture of three solvents: hexanes/dichloromethane/ethyl acetate in a ratio 50:50:10 v/v.

The structures of these cages were fully characterized by the advanced NMR techniques (^1^H–^1^H COSY, TOCSY, ROESY, and ^1^H–^13^C HSQC, HMBC, HSQC–TOCSY), as well as ECD spectroscopy and ESI-HRMS.

### NMR Spectroscopy Results

The identification of all four separated isomers was more complex comparing to compounds described earlier.^35^ Each of these structures contains three naphthalene rings, the CTV scaffold, and five benzyl groups, which significantly complicate the identification process, due to big crowding of the ^1^H/^13^C chemical shifts in the range typical for aromatic rings. Careful analysis of all NMR spectra supported by additional results obtained from the ECD measurements allowed us to determine unambiguously the structures of each isomer. Correct assignments of the proper structures for each compound based only on the NMR data could be misleading since such simplified analysis would give ambiguous results in proper structure determination. For example, the correct structure of isomer ***P*-5a**, in which the C-1′ atom of fructose is connected with ring C of the CTV unit and the C-6′ atom with ring B, while the CTV scaffold has a *P*-stereodescriptor, cannot be assigned only from the NMR data. The set of the ^1^H/^13^C chemical shifts and ROESY effects observed in the CTV part may also suggest the structure ***M*-5b** for this cage. The unambiguous assignment can be done only when the NMR spectra are supported with the ECD experiments (for more information, see the next part of this article).

Based on correct assignments of the ^1^H/^13^C signals in the NMR spectra for each individual isomer, some interesting remarks could be drawn. In the case of cages ***P*-5a** and ***M*-5a**, the value of ^3^*J*(H–H) for the H-1 anomeric proton of glucose is *ca.* 3.3 Hz, whereas for counter pair ***P*-5b** and ***M*-5b** is *ca.* 3.8 Hz. It could be concluded that a change of direction in the CTV cap can cause an appropriate effect in the H-1/H-2 position of protons related to a change of the dihedral angle between them. Comparison of the ^1^H and especially ^13^C chemical shifts for both nontwisted (***P*-5a**/***M*-5a)** and twisted (***P*-5b**/***M*-5b)** molecules indicates the relatively good compatibility of these data in the sucrose part for both pairs (Table S1). The analysis of the NMR data, in particular, ^13^C NMR chemical shifts, shows that, in the formation process of ***P*-5** and ***M*-5** derivatives, much more significant changes are observed for the fructose ring. This is manifesting, depending on the form of the cage, in strong shielding/deshielding effects at the C-1′ and C-6′ nuclei. In the case of cages ***P*-5a** and ***M*-5a**, the combination of sucrose and CTV fragments is connected with strong shielding increase by *ca.* 5 ppm of the C-1′ nucleus, as compared to shielding in cages ***P*-5b** and ***M*-5b** (Table 1). The opposite effect, however less pronounced, is noticed for the C-6′ nuclei. It suggests that its chemical shifts depend more on the position of the C-6′ methylene group in a specific product, which is clearly evident in ^1^H chemical shifts for the H-6′ protons. In the ***M*-5b** structure, a very strong shielding effect for the C-6′ methylene protons is observed (δ = 2.25 and 2.95 ppm), as compared to other isomers (Table 1). The difference between positions of these diastereoisomeric protons is bigger for twisted structures ***P*-5b**/***M*-5b** (*ca.* 0.5–0.7 ppm) than for nontwisted ***P*-5a**/***M*-5a** (*ca.* 0–0.2 ppm). This observation is probably strongly connected with other arrangement of the fructose fragment in **5a**/**5b** isomers.

**Table 1:** Comparison of the Selected ^1^H and ^13^C NMR Chemical Shifts δ (ppm) of ***P*-5a**, ***P*-5b**, ***M*-5a**, and ***M*-5b** Cages

	^1^H and ^13^C NMR chemical shifts δ (ppm)			
atom’s number^a^	***P*-5a**	***P*-5b**	***M*-5a**	***M*-5b**
H-1	5.19	5.55	5.60	5.54
H-6′a/H-6′b	3.36/3.54	2.84/3.37	3.46/3.49	2.25/2.95
C4′–OCH_2_Ph	4.40/4.48	3.51/3.70	4.37/4.42	4.09/3.93
C4′–OCH_2_–H–Ph	7.19–7.29	6.58/6.41/6.30	7.16–7.27	7.07/7.06/6.96
H-26/H-26′/H-26″ (OCH_3_)	3.40/3.55/3.21	3.60/3.44/3.73	3.92/3.22/3.48	3.02/4.03/3.19
C-1′	69.9	74.8	69.1	74.8
C-6′	73.0	70.9	73.1	72.7

a For numbering of atom, see Experimental Section.

Another conclusion, which may be drawn from the NMR data is related to the signals of the benzyl groups. In the ^1^H NMR spectra of ***P*-5b** and ***M*-5b**, the chemical shifts of such groups at the C-4′ atom are significantly different, as compared to ***P*-5a** and ***M*-5a** isomers. This is especially visible for structure ***P*-5b**, where the signals of the methylene protons of the benzyl group appear at δ = 3.51 and 3.70 ppm (Table 1). In this case, also, phenyl ring protons of the benzyl group are in the special isolated range (δ = 6.30–6.60 ppm). For isomer ***M*-5b**, the above mentioned phenomena also exist, but the results are less highlighted. A similar trend is typical for phenyl ring protons of the benzyl group attached to the C-4 atom in compound ***M*-5a**. Signals of these protons (δ = 5.92–6.50 ppm) are separated and more shielded than most aromatic protons of this cage. The position of the methoxy groups in the CTV part is also different for all isomers. The ^1^H chemical shifts are in the typical range (δ ca. 3.0–4.0 ppm), but the structure of the cage determines the values of their shifts. The biggest difference between ^1^H chemical shifts for these methoxy groups is noted for compound ***M*-5b** (ca. 1.0 ppm, Table 1). Moreover, due to the specific through-space interactions of the methoxy groups and aromatic protons from the CTV fragment, the proper assignment of the ^1^H/^13^C chemical shifts and thus structure correctness can be verified. All these above-mentioned remarks can be used in the future to find a relation of the NMR data and spatial arrangements of atoms defining the specific structure.

### ECD Spectroscopy Results

For more detailed structural information that would allow more deeply to explore the stereochemistry of these diastereoisomeric molecular cages, we turned our attention to the electronic circular dichroism (ECD) spectroscopy, which is one of the most suitable spectroscopic tools for this purpose. It is based on the study of interactions of circularly polarized light in the UV–vis region for exploring the 3D environment of chiral nonracemic compounds and allows to monitor even the smallest subtle changes in their structures. The successful combination of the ECD spectroscopy with quantum chemical calculations expands significantly the range of applicability of this spectroscopy.^34−36^

**Figure 2:**
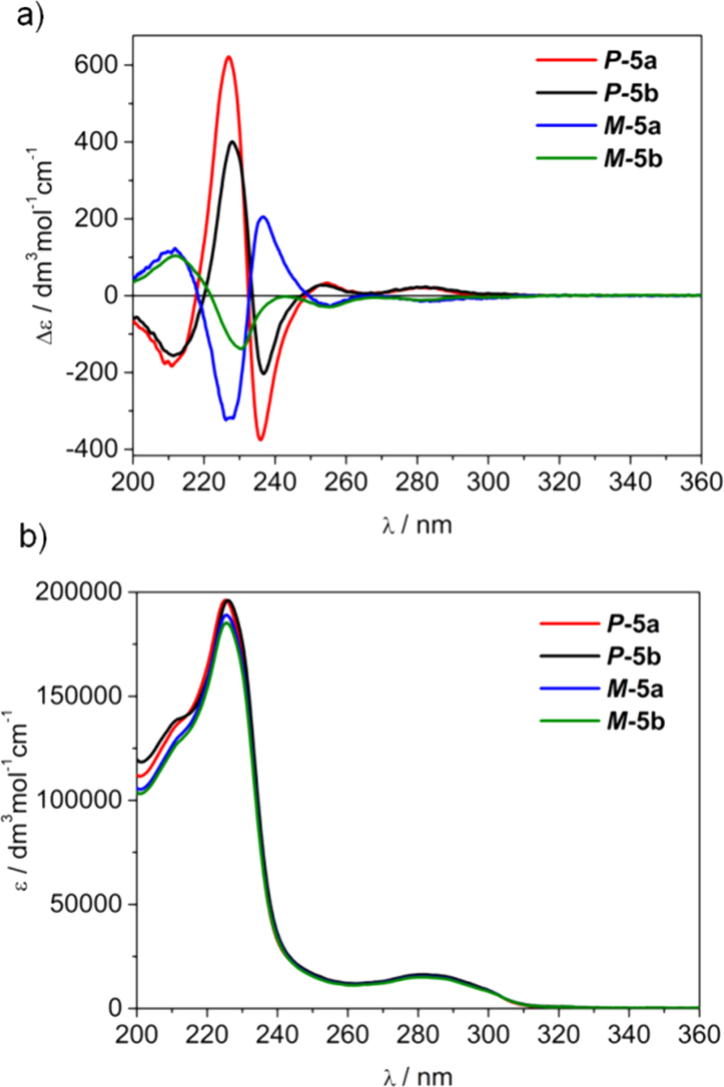
(a) ECD and (b) UV spectra of ***P*-5a**, ***M*-5a**, ***P*-5b**, and ***M*-5b** measured in CH_3_CN at room temperature.

Thus, the UV and ECD spectra of four diastereoisomeric molecular cages ***P*-5a**, ***M*-5a**, ***P*-5b**, and ***M*-5b** were recorded in CH_3_CN to assign their absolute stereochemistry (Figure 2).

The UV spectra of these compounds are almost identical and showed a manifold of bands at 280 and 225 nm associated with the CTV and naphthalene chromophores.

In the ECD spectra, there are a few very intense bands centered at around 235, 225, and 210 nm and rather intense ones at lower energy wavelengths at about 282 and 255 nm. In the case of molecular cage ***M*-5b**, being eluted as the fourth compound in the elution order under our conditions (see the Experimental Section part), the ECD curve showed some aberrations in the range 220–250 nm. Remarkably, the two curves, *i.e.*, ***P*-5a** and ***M*-5a** are associated with almost perfect mirror image of the ECD pattern. This is clearly evidenced by crossing exactly at zero values. In contrast, for ***P*-5b** and ***M*-5b**, the mirror image correlation was not perfect, as evidenced in Figure 2a by different absolute values of the ECD intensities in the range 220–250 nm, and by the fact that these two spectra do not cross precisely at zero values. Nevertheless, all spectra show characteristic features related to ^1^L_b_ and ^1^L_a_ transitions of aromatic chromophores. According to Collet *et al.*, the signs of the ^1^L_a_ bands can be used to assign the absolute configuration of the CTV unit: *M*-configuration is determined for molecules, which exhibit in their ECD spectra a sequence of signs *negative/positive* from *low* to *high* energy within this region, so analogously for *P*-configuration sequence is opposite.^37^ Thus, *ad hoc* for the first and second eluted peaks with *positive/negative* sequence of signs, the configuration was immediately assigned as ***P*-5a**/***P*-5b**, while for the third and fourth the opposite sequence indicates ***M*-5a**/***M*-5b** configuration.

### DFT Calculation Results

**Figure 3:**
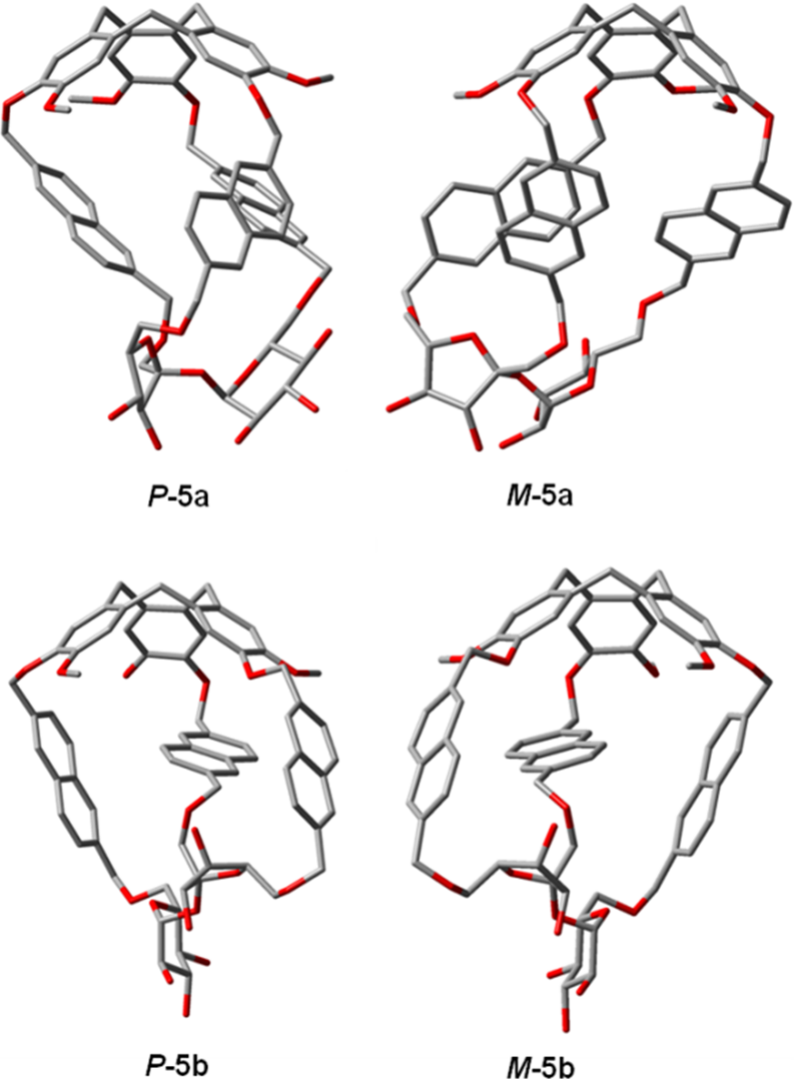
Lowest-energy conformers calculated at the B3LYP/6-31G(d)/PCM/CH_3_CN level of theory. Note: the hydrogen atoms are omitted for the sake of clarity.

To support this assignment, the quantum chemical calculations were carried out. First, the conformational search was done at the molecular mechanics level using a simplified structure in which benzyl groups (Bn) in the sucrose moiety were substituted with the hydrogen atom to facilitate the further computational predictions of ECD spectra. This approach preserves the main conformational landscapes of the investigated compounds and does not have any impact on the final stereochemical assignment. Then, the lowest energy structures within 3 kcal/mol were submitted for DFT optimization using Gaussian16 program^38^ at the B3LYP/6-31G(d) level of theory applying PCM for CH_3_CN. In this way, for each compound, two conformers were identified for ECD calculations. They mainly fluctuate in the rotation around the C–O bond(s) linking the CTV unit with naphthalene linker(s), while the rest of the molecule is well-kept. The lowest energy structures are presented in Figure 3. For TDDFT simulations, the following functional/basis-set combination was used: B3LYP/SVP with the polarizable continuum model (PCM) for CH_3_CN.

This level of approximation was indicated as one of the most successful in recent studies for investigating their ECD properties of systems with CTV moiety.^34,35,36b,36c^

**Figure 4:**
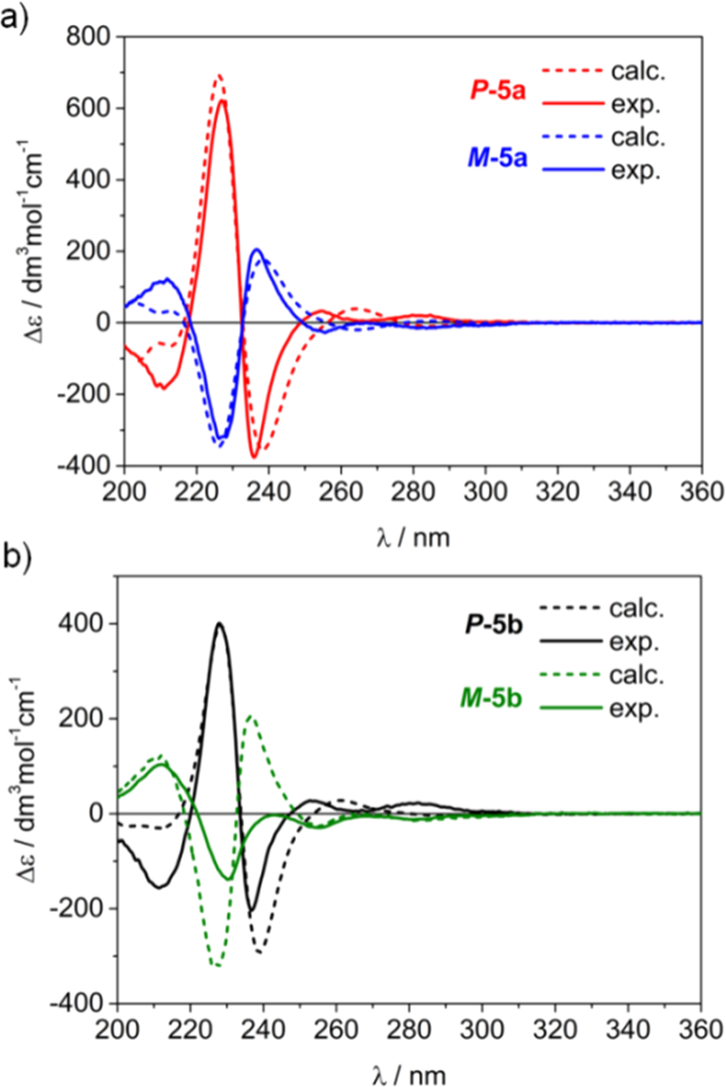
Comparison of calculated ECD spectra at the B3LYP/SVP/PCM (CH_3_CN) level of (a) ***P*-5a**, ***M*-5a**, and (b) ***P*-5b**, ***M*-5b** with experimental ones measured in CH_3_CN at room temperature. Note: all spectra are red-shifted by 5 nm and simulated using 0.15 eV Gaussian band-widths.

The simulated spectra are consistent with experimental ones (Figure 4); however, some minor inconsistencies are found in the range of ^1^L_b_ transitions. This is a well-known issue in TDDFT calculations of the ECD spectra since this band is simulated without taking into account a vibronic effect.^39^ Consequently, here, this subregion is excluded from our discussion.

The distinction of diastereoisomeric molecular cages was made by in-depth analysis of their chiroptical properties. Although the shapes of the ECD bands for two pairs of diastereoisomers are in line for ***P*-5a** and ***M*-5a**, the relative intensity of bands in the range 220–250 nm is higher in respect to the second pair ***P*-5b** and ***M*-5b**. The same observation can be found from TDDFT-calculated ECD spectra, which provides further strong evidence on the correctness of this stereochemical assignment.

### Recognition Studies

Then, we investigated the recognition properties of ***P*-5a**, ***P*-5b**, ***M*-5a**, and ***M*-5b** cages toward biologically interesting compounds, acetylcholine (ACh) and choline (Ch). The binding properties were determined by the fluorescence titration evaluating the emission spectra after the progressive addition of ACh or Ch solution to the host solution in the same solvent. We decided to choose this method due to the fast response time, high sensitivity, and the presence of the naphthalene linkers, which ensure fluorescence properties. The titration experiments were performed in acetonitrile, and iodide was chosen as a guest counter-ion because of good solubility.

**Figure 5:**
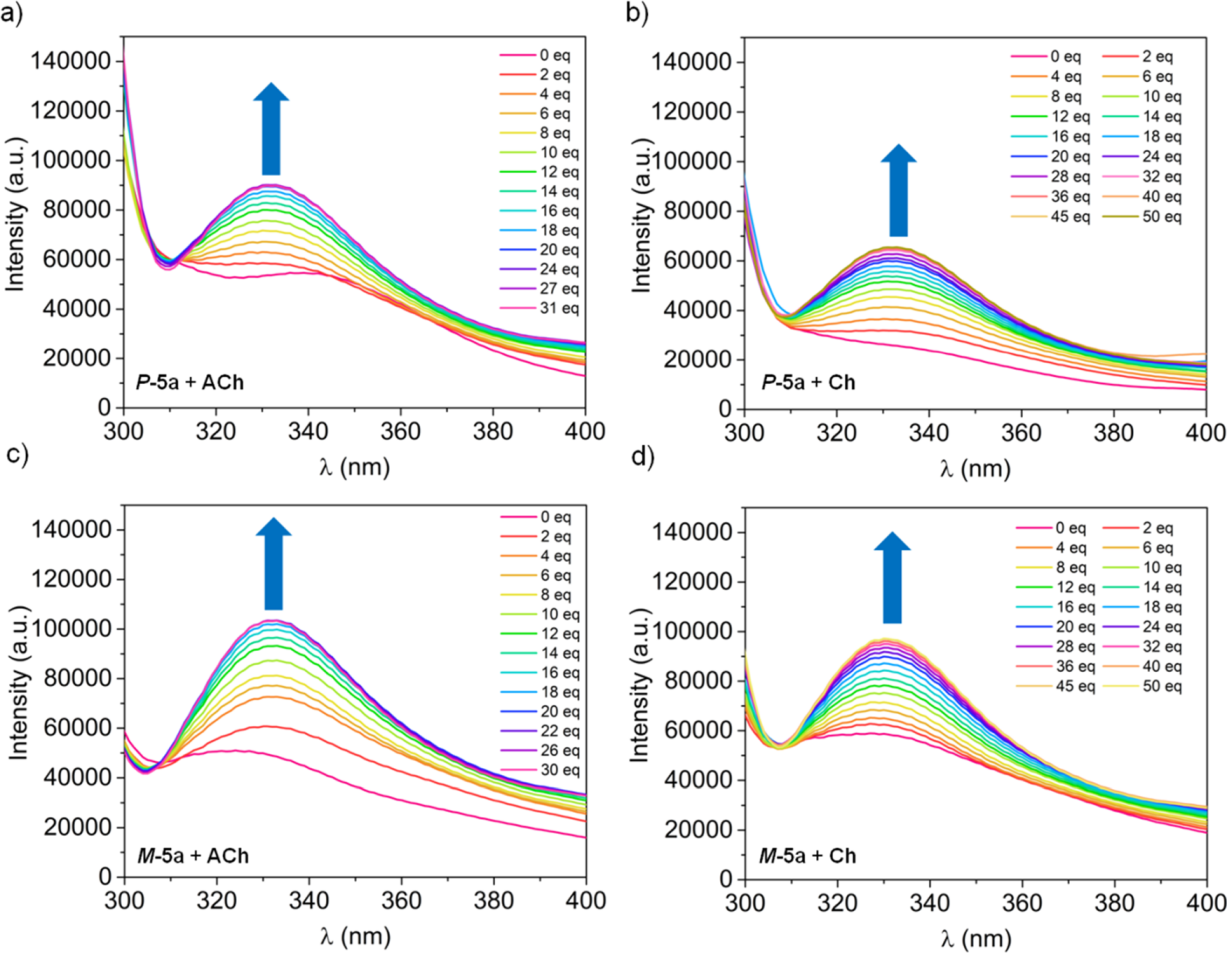
Fluorescent titration of hosts: (a) ***P*-5a** with ACh, (b) ***P*-5a** with Ch, (c) ***M*-5a** with ACh, and (d) ***M*-5a** with Ch in CH_3_CN at 298 K excited at 280 nm (counter-ion I^–^).

Fluorescence emission spectra of the hosts strongly differ after the addition of appropriate equivalents of guests. Indeed, an increase of fluorescence is observed for ***P*-5a** and ***M*-5a** hosts, whereas receptors ***P*-5b** and ***M*-5b** display a decrease of the fluorescence intensity upon the addition of acetylcholine or choline (Figure 5 and Figures S3, S5, S9, and S11). This opposite behavior might suggest the differences in formation of the host–guest complexes and the influence of chiral twisted/non-twisted structure of each diastereoisomer. The fluorescence enhancement might be assigned to the formation of rigid host–guest complex structures of nontwisted ***P*-5a** and ***M*-5a** cages stabilized by intermolecular hydrogen bonds.^26a^ Moreover, the twisted structures of compounds ***P*-5b** and ***M*-5b** ensure the different size and shape of the cavity than nontwisted ***P*-5a** and ***M*-5a**, which might also explain this binding differences.

The addition of acetylcholine resulted in a significant increase of the fluorescence of the ***P*-5a** and ***M*-5a** host at *ca.* 330 nm (Figure 5a,c). The binding constant (*K*_a_) for ***P*-5a** was 2.2 × 10^3^ M^–1^, whereas for ***M*-5a** was 5.6 × 10^3^ M^–1^. In the case of hosts ***P*-5b** and ***M*-5b**, the *K*_a_ values were 2.4 × 10^3^ and 0.6 × 10^3^ M^–1^, respectively (Table 2). These results show that compound ***M*-5a** is the most efficient host for ACh.

**Table 2:**
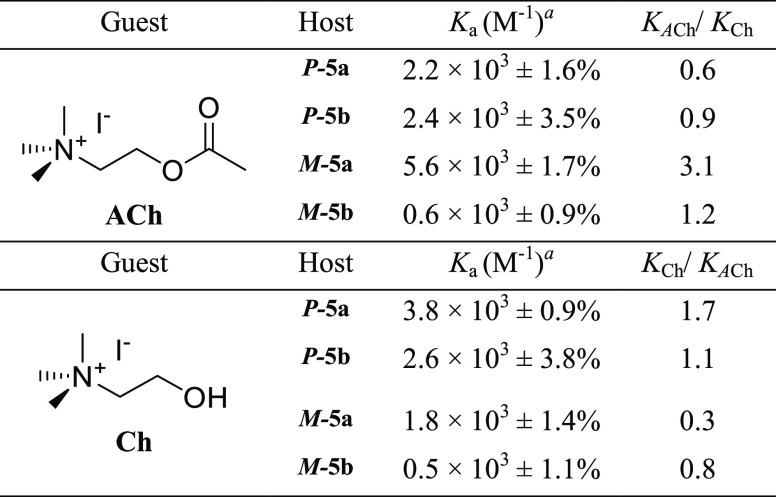
Comparison of Binding Constants *K_a_* (M^–1^) of ***P*-5a**, ***P*-5b**, ***M*-5a**, and ***M*-5b** Hosts with ACh and Ch

a Association constants *K*a were determined by fitting fluorescence titration curves (CH_3_CN, 298 K) using Bindfit program.^40^

During our recognition studies of choline, the remarkable increase of the fluorescence intensity was observed for ***P*-5a** and ***M*-5a** cages at *ca.* 330 nm (Figure 5b,d). In the case of ***P*-5b** and ***M*-5b**, quenching of fluorescence intensity was observed (Figures S9 and S11). The most significant value of binding constant *K*_a_ (3.8 × 10^3^ M^–1^) was achieved by receptor ***P*-5a**.

Lower binding constants were obtained for ***P*-5b** and ***M*-5a** cages, 2.6 × 10^3^ and 1.8 × 10^3^ M^–1^, respectively. The lowest *K*_a_ value was obtained for the ***M*-5b** host (0.5 × 10^3^ M^–1^) (Table 2). Comparing the recognition selectivity of ACh and Ch by these hosts, we can notice that compound ***P*-5a** is the most efficient sensor for choline (*K*_Ch_/*K*_ACh_ = 1.7), while ***M*-5a** is a more suitable receptor for acetylcholine (*K*_*A*Ch_/*K*_Ch_ = 3.1). The ***M*-5a** host could efficiently distinguish acetylcholine over choline. This selectivity is meaningful since both guests participate in the metabolic pathway. In the case of compounds ***P*-5b** and ***M*-5b**, no binding selectivity was observed. These differences in recognition of both guests could be rationalized by the structure of the molecular cages. As we can conclude from the DFT calculated structures, the chiral sucrose platform provides the unique shapes of the cavities of these receptors, which might allow to distinguish acetylcholine over choline and vice versa.

To supply the fluorescence recognition studies and get more information about the binding sites, the ^1^H NMR titration experiments of ***P*-5a** and ***M*-5a** cages with ACh and Ch were carried out (Figures S13, S16, S19, and S21). For this purpose, appropriate amounts of ACh or Ch solutions in CD_3_CN/CDCl_3_ (80:20) were gradually added to the host solution in the mixture of the same solvent. The ^1^H NMR studies show changes in the chemical shifts of both host and guest protons, which is in line with fast host–guest exchange on the NMR time scale. In all cases, the signals from (CH_3_)_3_N^+^ and methylene protons are shifted downfield with increasing amount of ACh or Ch guests (Figures S15 and S18). This could be explained by increasing the ratio of the unbounded guest.^26^ Compared to the spectra of pure ACh or Ch, the signals of both guests are shifted upfield during the titration studies, which confirms the encapsulation of both guests in the host cavities. There are a few reports about binding the (CH_3_)_3_N^+^ part of ACh and Ch inside the electron-rich CTV cavity.,^25,26a,27^ In the case of ***M*-5a**, the gradual addition of ACh provided downfield chemical shifts of the CTV aromatic protons and upfield shifts of the protons from naphthalene ring linkers (Figure S14). On the other hand, the recognition studies of Ch also show downfield shifts of the CTV aromatic protons, but upfield chemical shifts of naphthalene ring linkers were less significant (Figure S17).

The additional interactions of ACh ester group with the host may explain the higher binding constant and selectivity for ACh by ***M*-5a** cage. While the ^1^H NMR titration studies of ***P*-5a** cage with Ch show evident changes in the chemical shifts of aromatic part of CTV unit, as well as naphthalene rings, the binding studies with ACh demonstrate only slight changes of such chemical shifts (Figures S20 and S22). In this case, most likely, the shape of the cavity may cause the preference for Ch binding.

All these results indicate the formation of the corresponding host–guest complexes. Both guests are bind inside the electron-rich cavities, which is reflected in chemical shift changes observed during ^1^H NMR titration experiments, as well as in the changes of the fluorescence intensity. The observed differences in ACh and Ch recognition by these diastereoisomeric cages are, most likely, caused by original shapes of their cavities, created by various connections of both CTV and sucrose scaffolds. To further supply the binding properties of these cages and the stoichiometry of the complexation, ESI-MS measurements were carried out. Both hosts, ***P*-5a** and ***M*-5a**, form noncovalent complexes with choline cations [M + Ch]^+^ with an *m/z* value of 1761.81 (Figures S77 and S79), as well as with acetylcholine cations [M + ACh]^+^ with an *m/z* of value 1803.82 (Figures S78 and S80) in acetonitrile. These results show that adducts formed between cages ***P*-5a** or ***M*-5a** with Ach or Ch are relatively stable proving a 1:1 stoichiometry ratio. Adducts containing two guest molecules [M + 2ACh]^2+^ or [M + 2Ch]^2+^ were not detected.

**Figure 6:**
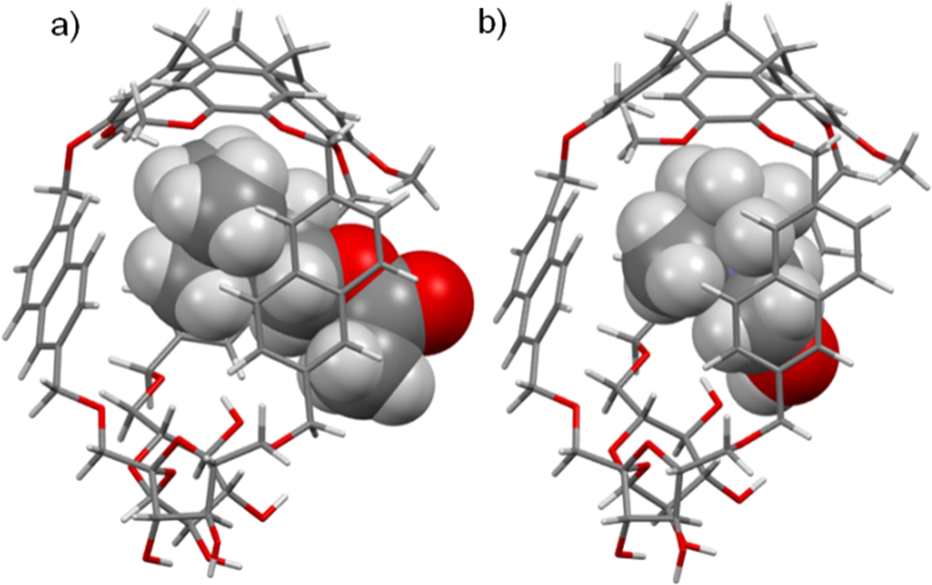
DFT-calculated structures of encapsulated complexes (a) ***M*-5a⊂ACh** and (b) ***M*-5a⊂Ch**.

Next, the DFT calculations of the host–guest inclusion complexes were performed to investigate further the selectivity of cage ***M*-5a** toward ACh over Ch. The optimized structures of both complexes show that ACh, as well as Ch, is partially encapsulated in the ***M*-5a** host cavity (Figure 6). These results are consistent with previously described CTV-based hemicryptophanes capable of binding ACh.^41^ In the case of ***M*-5a⊂ACh** complex, the ammonium unit is situated below the bowl-shaped CTV moiety, while the ester function is located between naphthalene linkers. Several CH−π interactions between the (CH_3_)_3_N^+^ part of ACh and phenyl rings of the CTV unit or naphthalene rings with distances ranging from 2.8 to 3.1 Å and from 2.5 to 3.0 Å, respectively, are observed. Moreover, cation−π interactions between positively charged nitrogen from ACh and CTV’s phenyl or naphthyl centroids occur with distances from 4.8 and 4.9 or 4.1 to 4.4 Å, respectively. Additionally, the interactions between (i) C=O or −O– from ACh and CH_3_ from the methoxy group (2.8 Å or 2.7 Å), (ii) C(O)CH_3_ from the ACh and naphthalene centroid (3.4 Å), and (iii) both CH_2_ from ACh and naphthalene centroids (distances from 2.4 to 3.4 Å) can be found. In the case of ***M*-5a⊂Ch**, complex similar cation-π, as well as CH−π, interactions between the (CH_3_)_3_N^+^ part of choline and aromatic rings of the CTV unit or naphthalene linkers are observed, but with greater distances. Indeed, the distance between positively charged nitrogen from choline and phenyl or naphthyl centroids from the host ranging from 5.1 to 5.6 or 4.2 to 4.5 Å, respectively. These results show that the ammonium part of Ch is bound weaker than ACh by the CTV unit. The CH−π interactions between (CH_3_)_3_N^+^ and phenyl or naphthyl centroids in ***M*-5a⊂Ch** are in distance ranging from 3.0 to 3.7 or 2.5 to 3.1 Å, respectively. Both CH_2_ groups from choline are also in distance from 3.1 to 3.5 Å in relation to naphthyl centroids. These DFT studies of both complexes give an insight in the binding details of the ***M*-5a** cage and support its selectivity toward ACh. The additional interactions of the ester group with ***M*-5a** cage and shorter distances between the CTV moiety and ammonium part of ACh could be responsible for this selectivity compared to Ch.

## Conclusions

In summary, we described the synthesis of four fluorescent diastereoisomeric molecular cages based on CTV and sucrose units connected *via* the naphthalene linkers. These compounds can act as efficient fluorogenic sensors for the detection of acetylcholine or choline. Application of the sucrose platform to the host structure is, as we assume, responsible for the unique shape of the cavity resulting in the selective recognition of these biologically important guests. Cage ***M*-5a** displays the strongest binding with acetylcholine, while cage ***P*-5a** mostly prefers choline. Both guests acetylcholine and choline could be, therefore, selectively recognized by these molecular cages using fluorescence spectroscopy.

## Experimental Section

### General Methods

All reagent-grade chemicals and solvents were received from commercial suppliers. TLC was performed on Merck silica gel 60F_254_ plates. Compounds were purified using an automatic flash chromatography system Knauer with UV and ELSD detection and Grace Resolv or Reveleris cartridges. Preparative HPLC was conducted on a Shimadzu SPD-6a spectrometer using a UV detector (254 nm) with a Vathsil 100 column (250 mm × 10 mm, particle size: 5 μm) and a 5 mL/min flow rate. The NMR spectra were recorded with a Varian VNMRS 600 MHz (at 600 MHz and 150 MHz for ^1^H and ^13^C NMR spectra, respectively) spectrometer for solutions in CDCl_3_ and TMS as internal standards. All significant resonances were assigned by COSY (^1^H–^1^H), ROESY (^1^H–^1^H), TOCSY (^1^H–^1^H), HSQC (^1^H–^13^C), and HMBC (^1^H–^13^C) correlations. Mass spectra were measured using a Synapt G2-S HDMS (Waters Inc.) mass spectrometer equipped with an electrospray ion source and q-TOF type mass analyzer or using an AutoSpec Premier (Waters Inc.) double-focusing magnetic sector mass spectrometer with an EBE geometry equipped with an EI (electron impact) ion source. Optical rotations were measured with a Jasco P 2000 apparatus in CHCl_3_ or CH_2_Cl_2_ with a sodium lamp at room temperature. Elemental analyses were obtained with a Perkin-Elmer 2400 CHN analyzer. The ECD and UV spectra were recorded in a CH_3_CN on a Jasco J-715 spectropolarimeter. The fluorescence titration experiments were performed using a Shimadzu RF-6000 fluorescence spectrometer.

2,6-Bis(bromomethyl)naphthalene was synthesized according to the literature procedure.^42^ All reactions were carried out under an argon atmosphere. Organic solutions were dried over anhydrous Na_2_SO_4_.

#### Synthesis of 2,3,3′,4,4′-Penta-*O*-benzyl-1′,6,6′-tri-*O*-[5-( bromomethyl)-naphthyl]-sucrose (**3**)

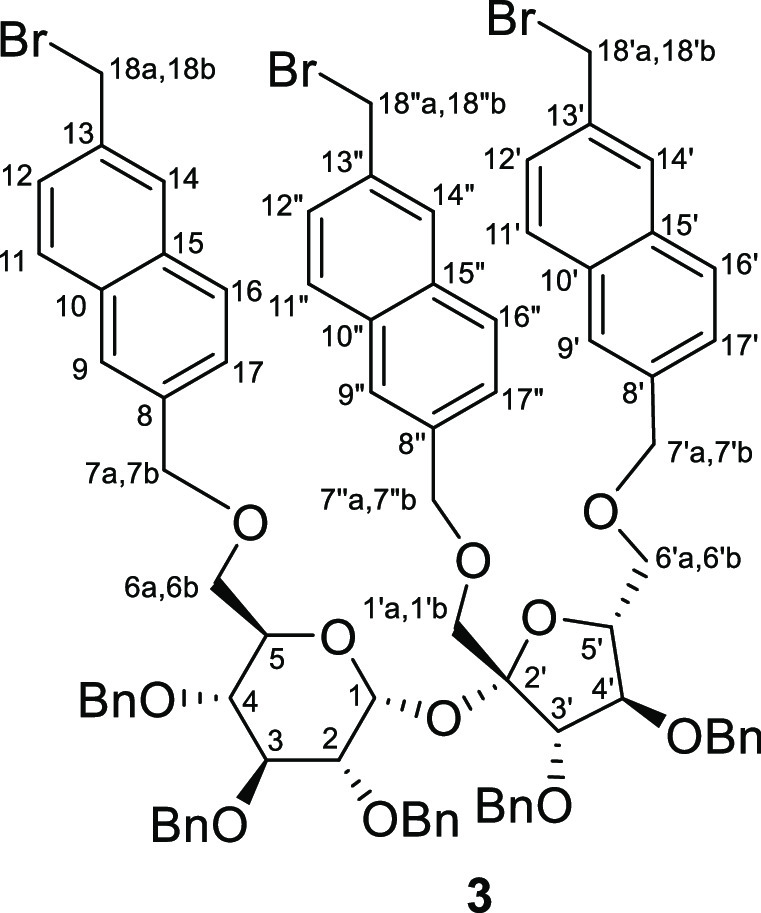



Sodium hydride (453.6 mg, 18.9 mmol, 60% dispersion in mineral oil) was added portionwise to a solution of triol **2** (1 g, 1.26 mmol) in dry THF (20 mL). After stirring for 20 min. at room temperature, 2,6-bis(bromomethyl)naphthalene (2.37 g, 7.56 mmol) was added in one portion, and the mixture was stirred at room temperature overnight. The reaction was quenched by careful addition of methanol (2 mL), and the mixture was poured onto dichloromethane (20 mL) and water (20 mL). The layers were separated, the aqueous one was extracted with dichloromethane (3 × 20 mL), combined organic phases were washed with brine (60 mL), dried over Na_2_SO_4_, and concentrated, and the residue was purified by flash chromatography (hexanes/ethyl acetate = from 100:0 to 75:25) to afford **3** (446 mg, 0.3 mmol, 24%) as a yellowish oil. [α]_*D*_^25^ + 34.3 (*c* 0.5, CHCl_3_). ^1^H NMR (600 MHz, CDCl_3_): δ = 7.76 (s, 1H, H-Naphth), 7.65–7.74 (m, 9H, 9 × H-Naphth), 7.62 (d, *J* = 7.6 Hz, 1H, H-Naphth), 7.62 (s, 1H, H-Naphth), 7.43 (dd, *J* = 18.2 Hz, *J* = 1.7 Hz, 1H, H-Naphth), 7.43 (t, 1H, H-Naphth), 7.35–7.41 (m, 4H, 4 × H-Naphth), 7.11–7.24 (m, 23H, 23 × H-Ph), 7.01–7.04 (m, 2H, 2 × H-Ph), 5.78 (d, *J*_1,2_ = 3.5 Hz, 1H, H-1), 4.83 (d, *J* = 10.8 Hz, 1H, benzylic H), 4.80 (d, *J* = 11.0 Hz, 1H, benzylic H), 4.64 (s, 2H, 2 × C*H*_2_–Br), 4.61 (s, 2H, 2 × C*H*_2_–Br), 4.70 (m, 1H, H-7″a), 4.67 (m, 2H, H-7′a, benzylic H), 4.66 (m, 1H, benzylic H), 4.64 (m, 1H, H-7′b), 4.60 (m, 1H, H-7a), 4.58 (s, 2H, 2 × C*H*_2_–Br), 4.57 (m, 1H, H-7″b), 4.56 (d, *J* = 12.4 Hz, 1H, benzylic H), 4.52 (d, *J* = 11.1 Hz, 1H, benzylic H), 4.50 (d, *J* = 11.0 Hz, 1H, benzylic H), 4.46 (m, 1H, H-3′), 4.43 (m, 1H, H-7b), (4.41–4.47 (m, 3H, 3 × benzylic H), 4.24 (dd, *J*_4′,3′_ = 7.5 Hz, *J*_4′,5′_ = 7.5 Hz 1H, H-4′), 4.14–4.17 (m, 1H, H-5′), 4.09 (m, 1H, H-5), 3.92 (dd, *J*_3,4_ = 9.3 Hz, *J*_3,2_ = 9.3 Hz, 1H, H-3), 3.72–3.80 (m, 3H, H-1′a, H-6′a, H-6′b), 3.62 (dd, *J*_4,5_ = 9.8 Hz, *J*_4,3_ = 9,4 Hz, 1H, H-4), 3.59 (d, *J*_1′b,1′a_ = 11.0 Hz, 1H, H-1′b), 3.48 (dd, *J*_6b,5_ = 3.5 Hz, *J*_6b,6a_ = 10.3 Hz 1H, H-6a), 3.46 (dd, *J*_2,1_ = 3.5 Hz, *J*_2,3_ = 9.5 Hz, 1H, H-2), 3.39 (dd, *J*_6b,5_ = 1.7 Hz, *J*_6b,6a_ = 10.6 Hz, 1H, H-6b) ppm. ^13^C{^1^H} NMR (150 MHz, CDCl_3_): δ = 139.0, 138.6, 138.4, 138.2, 138.2 (C_quat_, 5 × C-Ph), 136.7, 136.5, 136.4 (C-8, C-8′, C-8″), 135.3, 135.2, 135.1 (C-13, C-13′, C-13″), 133.1, 133.0, 132.9, 132.8, 132.8, 132.7 (C-10, C-10′, C-10″, C-15, C-15′, C-15″), 125.9–128.9 (m, 43C, 25 × C-Ph, 18 × C-Naphth), 104.7 (C-2′), 90.0 (C-1), 84.0 (C-3′), 82.3 (C-4′), 82.1 (C-3), 80.0 (C-2), 79.7 (C-5′), 77.8 (C-4), 75.6 (C3–O*C*H_2_Ph), 74.9 (C4–O*C*H_2_Ph), 73.5 (C-7″), 73.4 (C-7), 73.3 (C-7′), 73.2 (C3′–O*C*H_2_Ph), 72.7 (C4′–O*C*H_2_Ph), 72.3 (C2–O*C*H_2_Ph), 71.6 (C-1′), 71.6 (C-6′), 70.7 (C-5), 68.8 (C-6), 34.2, 34.2, 34.2 (C-18, C-18′, C-18″) ppm. MS (ESI-TOF) *m*/*z*: [M + Na]^+^ calcd for C_83_H_79_O_11_Br_3_Na 1511.31; found 1511.35. Anal. calcd for C_83_H_79_O_11_Br_3_: C, 66.81; H, 5.34; found: C, 66.69; H, 5.54.

#### Syntheses of CTV-Sucrose-Based Cages ***P*-5a**, ***M*-5a**, ***P*-5b**, and ***M*-5b**

To a solution of (±)**4** (24.5 mg, 0.06 mmol) in dry acetonitrile (40 mL), Cs_2_CO_3_ (175 mg, 0.54 mmol) was added and the mixture was stirred at room temperature for 30 min. The solution of compound **3** (89 mg, 0.06 mmol) in dry acetonitrile (20 mL) was added dropwise by a syringe pump within 4 h, and the mixture was stirred at reflux for additional 48 h. After cooling to room temperature, the mixture was filtered through Celite and the solvent was removed under vacuum. The residue was dissolved in CH_2_Cl_2_ (20 mL) and washed with water (20 mL). The aqueous phase was extracted with CH_2_Cl_2_ (3 × 20 mL), and the combined organic solutions were washed with brine (30 mL), dried, and concentrated. The resulting residue was purified by preparative HPLC (hexanes/dichloromethane/ethyl acetate = 50:50:10) to afford pure compounds ***P*-5a** (13 mg, 0.0078 mmol, 13%, colorless solid), ***P*-5b** (6 mg, 0.0036 mmol, 6%, white solid), ***M*-5a** (13 mg, 0.0078 mmol, 13%, colorless solid), and ***M*-5b** (7 mg, 0.0042 mmol, 7%, colorless solid). Total yield: 39%.

#### Characterization of Compound ***P*-5a**

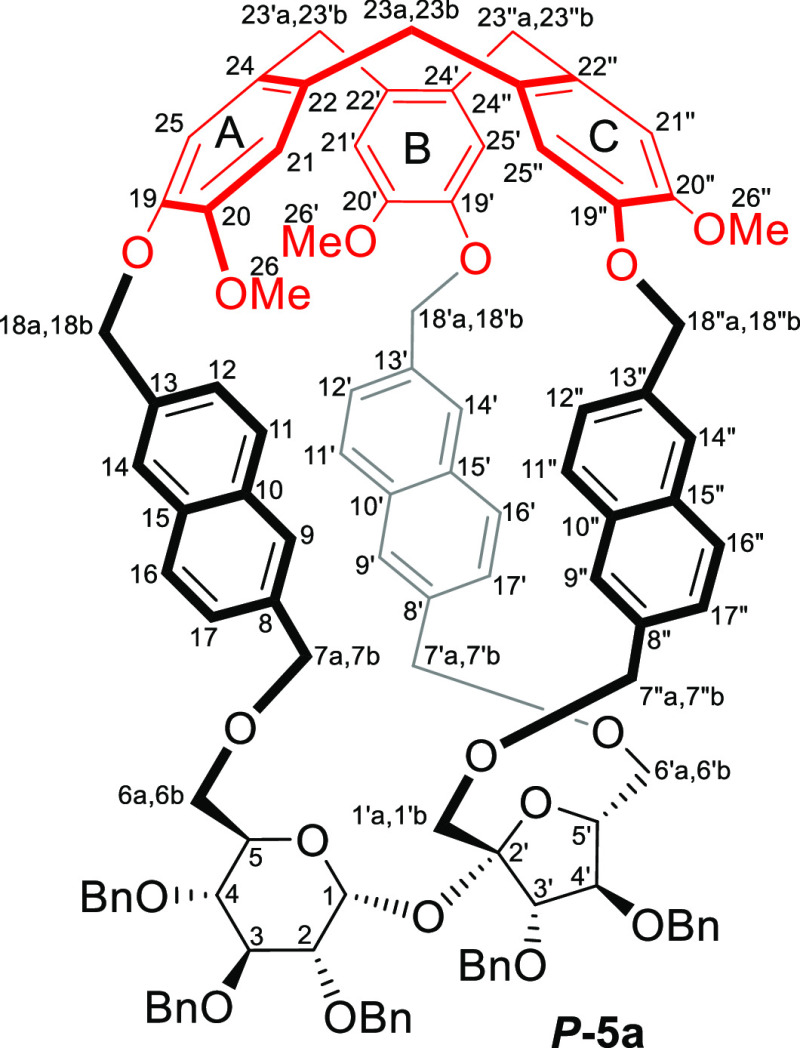



[α]_*D*_^25^ + 129.5 (*c* 0.22, CH_2_Cl_2_). ^1^H NMR (600 MHz, CDCl_3_): δ = 7.77 (s, 1H, H-14″), 7.65 (d, *J*_11″,12″_ = 8.7 Hz, 1H, H-11″), 7.64 (s, 1H, H-9″), 7.58 (d, *J*_16″,17″_ = 8.5 Hz, 1H, H-16″), 7.51 (d, *J*_11,12_ = 8.1 Hz, 1H, H-11), 7.51 (s, 1H, H-9′), 7.47 (s, 1H, H-9), 7.46 (s, 1H, H-14), 7.44 (d, *J*_11′,12′_ = 8.5 Hz, 1H, H-11′), 7.39 (d, *J*_16,17_ = 8.4 Hz, 1H, H-16), 7.36 (m, 1H, H-17″), 7.35 (m, 1H, H-12″), 7.34–7.36 (m, 2H, 2 × H-Ph), 7.21–7.29 (m, 15H, 15 × H-Ph), 7.16–7.19 (m, 4H, 4 × H-Ph), 7.16 (m, 1H, H-12), 7.11 (d, 1H, H-12′), 7.03 (d, 1H, H-17), 7.00–7.02 (m, 4H, 4 × H-Ph), 6.99 (s, 1H, H-25″), 6.95 (s, 1H, H-25′), 6.94 (s, 1H, H-14′), 6.88 (m, 1H, H-17′), 6.86 (m, 1H, H-25), 6.68 (d, *J*_16,17_ = 7.8 Hz, 1H, H-16′), 6.67 (s, 1H, H-21′), 6.64 (s, 1H, H-21), 6.61 (s, 1H, H-21″), 5.36 (d, *J*_18″a,18″b_ = 13.1 Hz, 1H, H-18″a), 5.26 (d, *J*_18″a,18″b_ = 12.9 Hz, 1H, H-18′a), 5.24 (d, *J*_18a,18b_ = 10.8 Hz, 1H, H-18a), 5.19 (d, *J*_1,2_ = 3.3 Hz, 1H, H-1), 5.16 (d, 1H, H-18″b), 5.15 (d, 1H, H-18′b), 4.81 (d, 1H, H-18b), 4.76 (d, *J*_7″a,7″b_ = 12.4 Hz, 1H, H-7″a), 4.76 (d, *J* = 11.2 Hz, 1H, C3′–OC*H*_2_Ph), 4.70 (m, 3H, H-23a, H-23′a, C4–OC*H*_2_Ph), 4.68 (m, 2H, H-23″a, C3–OC*H*_2_Ph), 4.63 (d, *J* = 11.4 Hz, 1H, C3′–OC*H*_2_Ph), 4.49 (m, 1H, C3–OC*H*_2_Ph), 4.48 (m, 3H, 2 × C2–OC*H*_2_Ph, C4′–OC*H*_2_Ph) 4.46 (m,1 H, H-7′a), 4.45 (m, 1H, H-7a), 4.41 (m, 1H, C4–OC*H*_2_Ph), 4.40 (m, 1H, C4′–OC*H*_2_Ph), 4.38 (m, 1H, H-3′), 4.30 (d, *J*_7′b,7′a_ = 13.4 Hz, 1H, H-7′b), 4.27 (d, 1H, H-7″b), 4.15 (d, *J*_7b,7a_ = 12.4 Hz, 1H, H-7b), 4.07 (m, 1H, H-5′), 3.83 (m, 1H, H-4′), 3.81 (m, 1H, H-5), 3.79 (m, 1H, H-3), 3.65 (d, *J*_1′a,1′b_ = 11.0 Hz, 1H, H-1′a), 3.56 (m, 1H, H-4), 3.55 (s, 3H, H-26′), 3.54 (m, 1H, H-6′a), 3.53 (m, 1H, H-6a), 3.51 (m, 1H, H-23b), 3.48 (m, 1H, H-23″b), 3.47 (m, 1H, H-23′b), 3.45 (d, 1H, H-1′b), 3.40 (s, 3H, H-26), 3.36 (dd, *J*_6′b,5′_ = 8.00 Hz, *J*_6′b,6′a_ = 11.9 Hz 1H, H-6′b), 3.30 (dd, *J*_2,3_ = 9.7 Hz, *J*_2,1_ = 3.4 Hz, 1H, H-2), 3.21 (s, 3H, H-26″), 3.17 (dd, *J*_6b,5_ = 1.4 Hz, *J*_6b,6a_ = 10.8 Hz, 1H, H-6b) ppm. ^13^C{^1^H} NMR (150 MHz, CDCl_3_): δ = 149.1 (C-20′), 148.8 (C-20″), 148.5 (C-20), 147.0 (C-19″), 146.1 (C-19), 145.9 (C-19′), 138.8, 138.6, 138.3, 138.2, 138.2 (C_quat_, 5 × C-Ph), 136.9 (C-8′), 135.9 (C-8), 135.8 (C-8″), 135.3 (C-13″), 134.2 (C-13), 133.7 (C-22′), 133.3 (C-13′), 133.3 (C-22″), 132.8 (C-10/C-15), 132.7 (C-22), 132.6 (C-15″), 132.6 (C-10″), 132.6 (C-10′), 132.5 (C-15′), 132.0 (C-24″), 132.0 (C-10/C-15), 131.7 (C-24′), 131.6 (C-24), 128.1 (C-11″), 127.9 (C-16″), 127.8 (C-11), 127.7 (C-16′), 127.7 (C-16), 127.2–128.3 (m, 25 × C-Ph), 126.8 (C-14′), 126.3 (C-14), 126.3 (C-17″), 126.2 (C-9″), 126.2 (C-17), 126.1 (C-9), 126.1 (C-11′), 125.6 (C-17′), 125.6 (C-12′), 125.4 (C-14″), 125.4 (C-12), 124.8 (C-12″), 124.5 (C-9′), 118.6 (C-25′), 117.6 (C-25″), 116.3 (C-25), 113.4 (C-21″), 113.4 (C-21′), 113.0 (C-21), 104.1 (C-2′), 90.7 (C-1), 84.1 (C-3′), 82.4 (C-4′), 81.6 (C-3), 80.9 (C-5′), 79.9 (C-2), 77.5 (C-4), 75.4 (C3–O*C*H_2_Ph), 74.7 (C4–O*C*H_2_Ph), 73.1 (C-7), 73.0 (C-7″), 73.0 (C-6′), 72.9 (C3′–O*C*H_2_Ph), 72.7 (C-18″), 72.6 (C-18′), 72.5 (C2–O*C*H_2_Ph), 72.5 (C-7′), 72.4 (C4′–O*C*H_2_Ph), 71.4 (C-18), 70.6 (C-5), 69.9 (C-1′), 68.1 (C-6), 55.9 (C-26′), 55.5 (C-26), 55.5 (C-26″), 36.4 (C-23), 36.4 (C-23′), 36.2 (C-23″) ppm. HRMS (ESI-TOF) *m*/*z*: [M + Na]^+^ calcd for C_107_H_100_O_17_Na 1679.6858; found 1679.6844. Anal. calcd for C_107_H_100_O_17_ + H_2_O: C, 76.68; H, 6.13; found: C, 76.55; H, 6.25.

#### Characterization of Compound ***P*-5b**

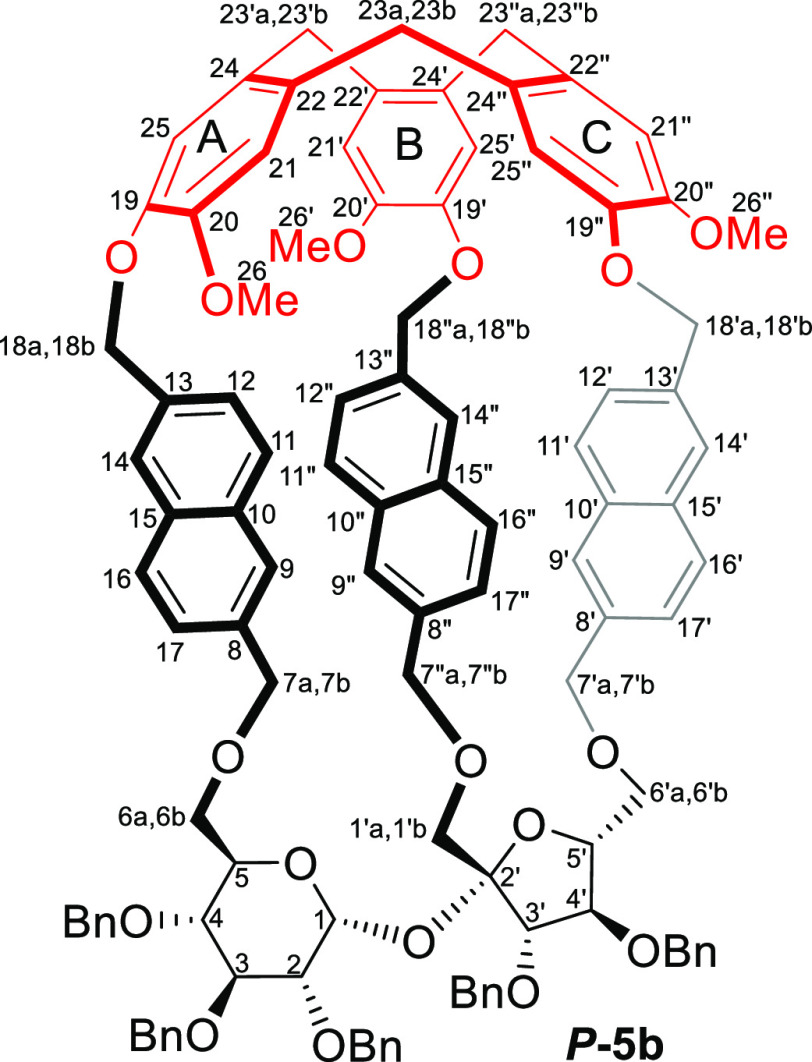



[α]_*D*_^25^ + 103.2 (*c* 0.22, CH_2_Cl_2_). ^1^H NMR (600 MHz, CDCl_3_): δ = 7.65 (d, *J*_11,12_ = 8.3 Hz, 1H, H-11), 7.62 (s, 1H, H-9), 7.57 (s, 1H, H-14), 7.56 (s, 1H, H-14″), 7.55 (m, 2H, H-11′, H-16), 7.52 (d, *J*_16″,17″_ = 8.4 Hz, 1H, H-16″), 7.49 (s, 1H, H-9″), 7.47 (s, 1H, H-9′), 7.38 (m, 1H, H-17), 7.32 (m, 1H, H-12′), 7.27 (m, 1H, H-12), 7.26–7.40 (m, 16H, 16 × H-Ph), 7.23 (d, *J*_11″,12″_ = 8.2 Hz, 1H, H-11″), 7.09 (s, 1H, H-25′), 7.07 (s, 1H, H-25″), 7.06 (s, 1H, H-14′), 7.02–7.05 (m, 5H, H-17″, 4 × H-Ph), 6.91 (d, *J*_17′,16′_ = 8.1 Hz, 1H, H-17′), 6.88 (s, 1H, H-25), 6.87 (m, 1H, H-12″), 6.86 (s, 1H, H-21″), 6.81 (s, 1H, H-21′), 6.79 (d, *J*_16′,17′_ = 8.4 Hz 1H, H-16′), 6.70 (s, 1H, H-21), 6.58 (t, *J* = 7.6 Hz, 2H, 2 × H-Ph), 6.41 (d, *J* = 7.4 Hz, 2H, 2 × H-Ph), 6.30 (t, *J* = 7.4 Hz, 1H, H-Ph), 5.55 (d, *J*_1,2_ = 3.8 Hz, 1H, H-1), 5.46 (d, *J*_18′a,18′b_ = 13.3 Hz, 1H, H-18′a), 5.35 (d, 1H, H-18′b), 5.27 (d, *J*_18″a,18″b_ = 12.7 Hz, 1H, H-18″a), 5.09 (d, *J*_18a,18b_ = 11.7 Hz, 1H, H-18a), 4.94 (d, 1H, H-18″b), 4.93 (d, *J* = 11.0 Hz, 1H, C3–OC*H*_2_Ph), 4.89 (d, *J* = 11.6 Hz, 1H, C4–OC*H*_2_Ph), 4.87 (d, 1H, H-18b), 4.79 (m, 1H, C3–OC*H*_2_Ph), 4.78 (m, 1H, H-7a), 4.77 (m, 3H, H-23a, H-23′a, H-23″a), 4.74 (d, *J* = 12.0 Hz, 1H, C2–OC*H*_2_Ph), 4.67 (d, *J* = 11.6 Hz, 1H, C2–OC*H*_2_Ph), 4.66 (m, 2H, H-7″a, C4–OC*H*_2_Ph), 4.58 (d, *J*_7″b,7″a_ = 12.1 Hz, 1H, H-7″b), 4.46 (d, *J* = 11.2 Hz, 1H, C3′–OC*H*_2_Ph), 4.24 (d, *J*_3′,4′_ = 8.2 Hz, 1H, H-3′), 4.23 (d, *J*_7b,7a_ = 11.9 Hz, 1H, H-7b), 4.21 (d, *J*_7′a,7′b_ = 12.9 Hz, 1H, H-7′a), 4.19 (d, *J* = 11.4 Hz, 1H, C3′–OC*H*_2_Ph), 4.13 (m, 1H, H-5), 3.95 (d, 1H, H-7′b), 3.92 (dd, *J*_3,2_ = 9.1 Hz, *J*_3,4_ = 9.3, 1H, H-3), 3.73 (s, 3H, H-26″), 3.71 (d, *J*_1′a,1′b_ = 11.9 Hz, 1H, H-1′a), 3.70 (d, *J* = 11.5 Hz, 1H, C4′–OC*H*_2_Ph) 3.61 (m, 1H, H-2), 3.60 (s, 3H, H-26), 3.59 (m, 1H, H-23″b), 3.58 (m, 1H, H-1′b), 3.57 (m, 2H, H-4, H-23′b), 3.55 (m, 1H, H-23b), 3.53 (m, 1H, H-4′), 3.51 (d, *J* = 10.9 Hz, 1H, C4′–OC*H*_2_Ph), 3.44 (s, 3H, H-26′), 3.37 (dd, *J*_6′a,6′b_ = 10.9 Hz, *J*_6′a,5′_ = 11.1 Hz, 1H, H-6′a), 3.32 (m, 1H, H-5′), 3.31 (m, 1H, H-6a), 3.26 (dd, *J*_6b,5_ = 4.0 Hz, *J*_6b,6a_ = 10.5 Hz, 1H, H-6b), 2.84 (dd, *J*_6′a,5′_ = 1.9 Hz, 1H, H-6′b) ppm. ^13^C{^1^H} NMR (150 MHz, CDCl_3_): δ = 149.7 (C-20″), 149.1 (C-20′), 148.4 (C-20), 147.6 (C-19′) 147.3 (C-19), 145.3 (C-19″), 139.0, 138.8, 138.4, 137.8, 137.8 (C_quat_, 5 × C-Ph), 136.5 (C-8″), 136.1 (C-8′), 135.3 (C-8), 135.1 (C-13), 134.7 (C-13″), 134.0 (C-22″), 133.6 (C-22′), 133.5 (C-13′), 132.8 (C-22), 132.8 (C-10″/C-15″), 132.8 (C-15), 132.6 (C-10), 132.5 (C-24′), 132.4 (C-10′), 132.3 (C-10″/C-15″), 132.3 (C-24″), 132.2 (C-15′), 131.6 (C-24), 128.2 (C-16), 127.8 (C-11), 127.7 (C-16″), 127.6 (C-16′, C-11′), 127.4–128.4 (m, 22 × C-Ph), 127.0 (C-9), 126.8, 126.7 (3 × C-Ph), 126.5 (C-17), 126.3 (C-14″, C-14′), 126.3 (C-17′), 126.0 (C-9′), 125.9 (C-14), 125.8 (C-12′), 125.5 (C-9″), 125.4 (C-12″, C-17″), 125.3 (C-11″, C-12), 120.0 (C-25″), 118.5 (C-25′), 116.4 (C-25), 114.4 (C-21′), 113.9 (C-21″), 113.5 (C-21), 104.0 (C-2′), 88.7 (C-1), 82.5 (C-3′), 82.3 (C-3), 81.3 (C-4′), 79.3 (C-2), 79.2 (C-5′), 78.0 (C-4), 75.4 (C3–O*C*H_2_Ph), 74.8 (C-1′), 74.7 (C4–O*C*H_2_Ph), 74.4 (C-7″), 73.9 (C-18″), 73.0 (C-7), 72.6 (C-7′), 72.3 (C-18′), 72.2 (C2–O*C*H_2_Ph), 72.2 (C3′–O*C*H_2_Ph), 71.4 (C4′–O*C*H_2_Ph), 71.3 (C-18), 70.9 (C-6′), 69.9 (C-5), 67.6 (C-6), 56.3 (C-26″), 56.2 (C-26′), 56.0 (C-26), 36.6 (C-23′), 36.5 (C-23″), 36.3 (C-23) ppm. HRMS (ESI-TOF) *m*/*z*: [M + Na]^+^ calcd for C_107_H_100_O_17_Na 1679.6858; found 1679.6836. Anal. calcd for C_107_H_100_O_17_ + H_2_O: C, 76.68; H, 6.13; found: C, 76.23; H, 6.25.

#### Characterization of Compound ***M*-5a**

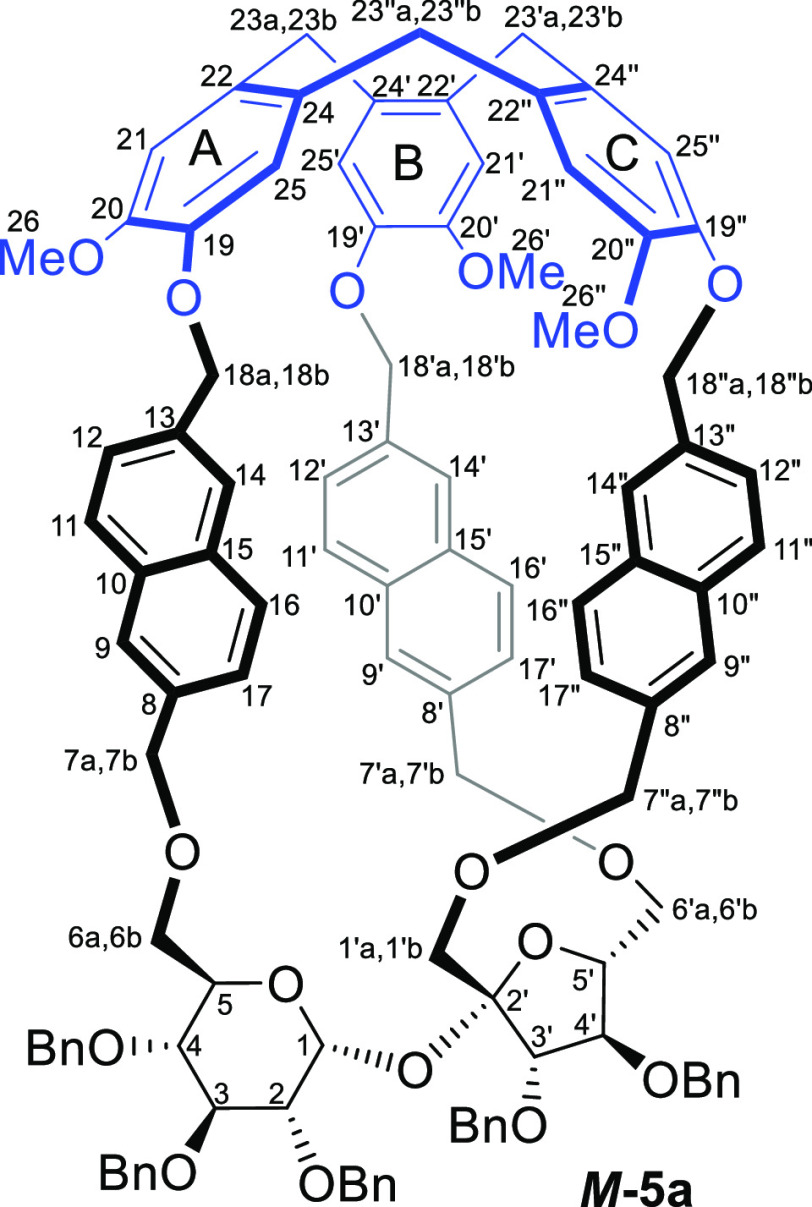



[α]_*D*_^25^ – 90.5 (*c* 0.22, CH_2_Cl_2_). ^1^H NMR (600 MHz, CDCl_3_): δ = 7.83 (s, 1H, H-9″), 7.79 (d, *J*_16″,17″_ = 8.4 Hz, 1H, H-16″), 7.76 (d, *J*_11″,12″_ = 8.4 Hz, 1H, H-11″), 7.73 (s, 1H, H-14″), 7.48 (d, 1H, H-17″), 7.45 (s, 1H, H-14), 7.44 (d, 1H, H-11′), 7.40 (s, 1H, H-9′), 7.38 (m, 1H, H-12″), 7.36 (m, 1H, H-9), 7.30 (m, 1H, H-11), 7.29–7.40 (m, 5H, 5 × H-Ph), 7.23 (m, 1H, H-16), 7.21 (m, 1H, H-12), 7.17 (m, 1H, H-12′), 7.16–7.27 (m, 13H, 13 × H-Ph), 7.12 (s, 1H, H-25′), 7.09 (s, 1H, H-25), 7.02 (m, 1H, H-17), 7.01 (m, 1H, H-17′), 6.99 (m, 1H, H-21), 6.98 (d, *J*_16′,17′_ = 8.6 Hz, 1H, H-16′), 6.98–7.04 (m, 2H, 2 × H-Ph), 6.93 (s, 1H, H-21″), 6.84 (s, 1H, H-14′), 6.76 (s, 1H, H-25″), 6.64 (s, 1H, H-21′), 6.50 (d, *J* = 7.5 Hz, 2H, 2 × H-Ph), 6.39 (t, *J* = 7.6 Hz, 2H, 2 × H-Ph), 5.92 (t, *J* = 7.3 Hz, 1H, 1 × H-Ph), 5.60 (d, *J*_1,2_ = 3.3 Hz, 1H, H-1), 5.40 (d, *J*_18a,18b_ = 13.5 Hz, 1H, H-18a), 5.31 (d, 1H, H-18b), 5.21 (d, *J*_18″a,18″b_ = 11.9 Hz, 1H, H-18″a), 5.20 (d, *J*_18′a,18′b_ = 11.9 Hz, 1H, H-18′a), 5.07 (d, *J*_7″a,7″b_ = 12.5 Hz, 1H, H-7″a), 4.97 (d, 1H, H-18″b), 4.79 (m, 1H, H-23a), 4.76 (m, 2H, 2 × C3′–OC*H*_2_Ph), 4.74 (m, 2H, H-23′a, H-23″a), 4.64 (d, *J*_7a,7b_ = 12.7 Hz, 1H, H-7a), 4.60 (d, *J* = 11.9 Hz, 1H, C2–OC*H*_2_Ph), 4.55 (d, 1H, C2–OC*H*_2_Ph), 4.54 (d, *J* = 10.8 Hz, 1H, C3–OC*H*_2_Ph), 4.50 (d, *J* = 10.8 Hz, 1H, C4–OC*H*_2_Ph), 4.50 (d, 1H, H-7″b), 4.42 (d, *J* = 11.4 Hz, 1H, C4′–OC*H*_2_Ph), 4.40 (d, 1H, H-18′b), 4.37 (d, 1H, C4′–OC*H*_2_Ph), 4.35 (m, 1H, H-3′), 4.34 (m, 2H, H-7′a, H-7′b), 4.15 (d, 1H, C3–OC*H*_2_Ph), 4.04 (m, 2H, H-7b, C4–OC*H*_2_Ph), 4.03 (m, 1H, H-4′), 3.92 (m, 1H, H-5′), 3.92 (s, 3H, H-26), 3.90 (m, 1H, H-5), 3.72 (m, 2H, H-1′a, H-3), 3.66 (d, *J*_1′b,1′a_ = 12.2 Hz, 1H, H-1′b), 3.61 (m, 2H, H-23b, H-23′b), 3.51 (d, *J*_23″b,23″a_ = 13.5 Hz, 1H, H-23″b), 3.49 (m, 1H, H-6′a), 3.48 (s, 3H, H-26″), 3.46 (m, 1H, H-6′b), 3.41 (m, 1H, H-4), 3.39 (m, 1H, H-6a), 3.38 (m, 1H, H-2), 3.22 (s, 3H, H-26′), 3.06 (dd, *J*_6b,5_ = 1.2 Hz, *J*_6b,6a_ = 10.4 Hz, 1H, H-6b) ppm. ^13^C{^1^H} NMR (150 MHz, CDCl_3_): δ = 150.3 (C-20′), 148.6 (C-20), 148.0 (C-20″), 147.3 (C-19″), 146.8 (C-19′), 145.8 (C-19), 138.7, 138.6, 138.1, 138.0, 137.8 (C_quat_, 5 × C-Ph), 136.3 (C-8′), 135.9 (C-8″), 135.4 (C-8), 134.9 (C-13″), 134.7 (C-22′), 134.3 (C-13), 133.6 (C-13′), 133.2 (C-22), 132.8, 132.8, 132.6, 132.5, 132.5 (C_quat_, 5 × C-Naphth) 132.4 (C-24″), 132.1 (C_quat_, C-Naphth), 132.1 (C-24′), 131.9 (C-24), 131.8 (C-22″), 128.4 (C-11″), 128.2 (C-14′), 128.0 (C-16″), 128.0 (C-16), 127.9 (C-16′), 127.8 (C-11), 127.4 (C-9″), 127.3–128.3 (m, 25 × C-Ph), 127.2 (C-17″), 127.2 (C-11′), 126.7 (C-9), 126.6 (C-14), 126.6 (C-12′), 126.3 (C-17), 126.3 (C-14″), 125.9 (C-12), 125.5 (C-12″), 125.0 (C-17′), 124.4 (C-9′), 121.9 (C-25′), 116.7 (C-25), 115.0 (C-21″), 113.8 (C-21), 113.5 (C-21′), 113.3 (C-25″), 104.6 (C-2′), 91.4 (C-1), 84.9 (C-3′), 83.1 (C-4′), 81.4 (C-3), 80.8 (C-5′), 79.7 (C-2), 77.7 (C-4), 76.2 (C-18′), 75.1 (C3–O*C*H_2_Ph), 74.3 (C4–O*C*H_2_Ph), 73.9 (C-7″), 73.9 (C-7), 73.3 (C3′–O*C*H_2_Ph), 73.1 (C-6′), 72.7 (C4′–O*C*H_2_Ph), 72.5 (C2–O*C*H_2_Ph), 72.3 (C-7′), 71.3 (C-18″), 70.4 (C-5), 70.2 (C-18), 69.1 (C-1′), 67.9 (C-6), 56.3 (C-26), 55.7 (C-26″), 55.5 (C-26′), 36.5 (C-23′), 36.4 (C-23″), 36.2 (C-23) ppm. HRMS (ESI-TOF) *m*/*z*: [M + Na]^+^ calcd for C_107_H_100_O_17_Na 1679.6858; found 1679.6866. Anal. calcd for C_107_H_100_O_17_ + H_2_O: C, 76.68; H, 6.13; found: C, 76.40; H, 6.28.

#### Characterization of Compound ***M*-5b**

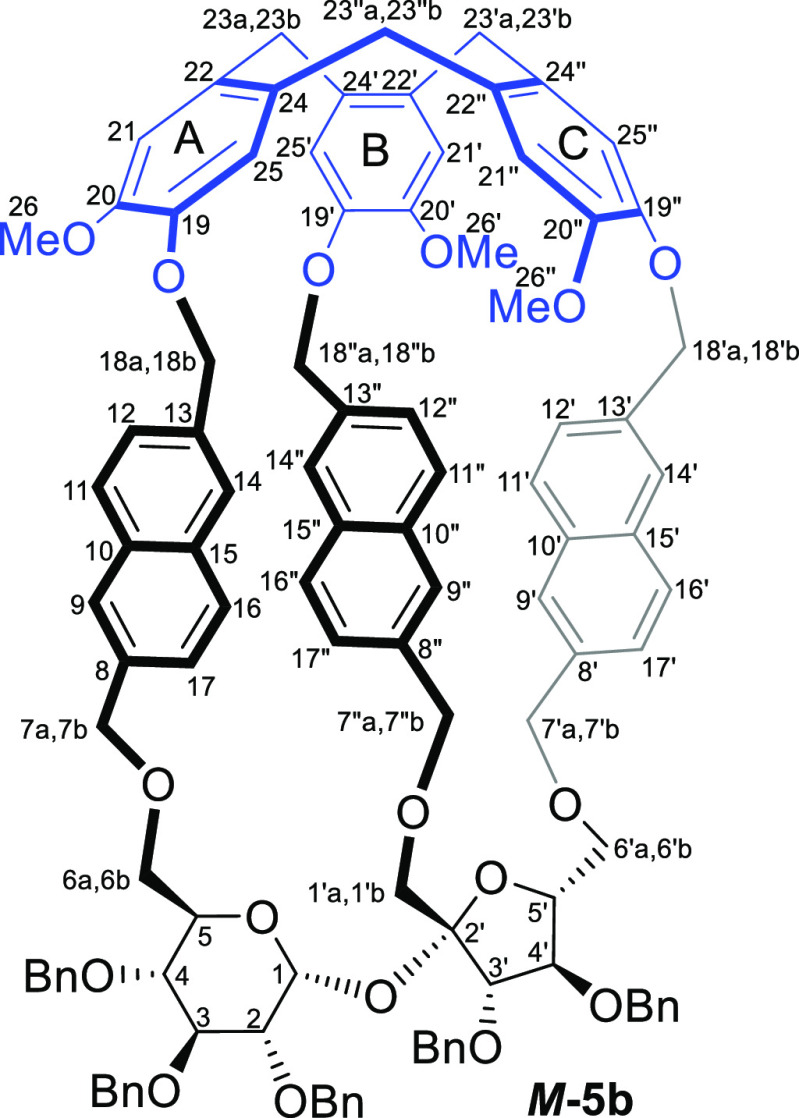



[α]_*D*_^25^ – 82.1 (*c* 0.21, CH_2_Cl_2_). ^1^H NMR (600 MHz, CDCl_3_): δ = 7.78 (s, 1H, H-9), 7.70 (s, 1H, H-14″), 7.69 (d, *J*_16,17_ = 8.6 Hz, 1H, H-16), 7.67 (s, 1H, H-11), 7.62 (s, 1H, H-9″), 7.58 (s, 1H, H-14), 7.52 (d, 1H, H-17), 7.49 (d, *J*_11″,12″_ = 8.4 Hz, 1H, H-11″), 7.49 (d, *J*_16″,17″_ = 8.3 Hz, 1H, H-16″), 7.34 (s, 1H, H-25″), 7.32 (m, 1H, H-12″), 7.24–7.42 (m, 20H, 20 × H-Ph), 7.21 (d, 1H, H-17″), 7.19 (s, 1H, H-21′), 7.18 (d, 1H, H-11′), 7.16 (s, 1H, H-9′), 7.14 (m, 1H, H-12), 7.13 (s, 1H, H-25′), 7.12 (d, *J*_12′,11′_ = 8.4 Hz, 1H, H-12′), 7.04–7.09 (m, 4H, 4 × H-Ph), 6.96 (t, *J* = 6.9 Hz, 1H, H-Ph), 6.55 (s, 1H, H-14′), 6.54 (s, 1H, H-21), 6.49 (d, *J*_17′,16′_ = 8.2 Hz, 1H, H-17′), 6.42 (s, 1H, H-25), 6.33 (s, 1H, H-21″), 6.14 (d, 1H, H-16′), 5.64 (d, *J*_18″a,18″b_ = 14.7 Hz, 1H, H-18″a), 5.54 (d, *J*_1,2_ = 3.8 Hz, 1H, H-1), 5.45 (d, 1H, H-18″b), 5.35 (d, *J*_18′a,18′b_ = 11.2 Hz, 1H, H-18′a), 5.00 (d, 1H, H-18′b), 4.94 (m, 1H, H-7a), 4.93 (m, 2H, C3–OC*H*_2_Ph, C4–OC*H*_2_Ph), 4.87 (d, *J*_18a,18b_ = 9.3 Hz, 1H, H-18a), 4.83 (d, *J* = 11.1 Hz, 1H, C3–OC*H*_2_Ph), 4.79 (d, *J*_23′a,23′b_ = 13.5 Hz, 1H, H-23′a), 4.77 (d, *J* = 11.2 Hz, 1H, C4–OC*H*_2_Ph), 4.74 (d, *J*_23a,23b_ = 13.4 Hz, 1H, H-23a), 4.71 (d, 1H, H-23″a), 4.70 (d, *J* = 13.0 Hz, 1H, C3′–OC*H*_2_Ph), 4.69 (m, 2H, C2–OC*H*_2_Ph), 4.67 (d, 1H, H-7″a), 4.59 (d, *J*_7″b,7″a_ = 11.9 Hz, 1H, H-7″b), 4.45 (d, *J* = 11.1 Hz, 1H, C3′–OC*H*_2_Ph), 4.29 (d, *J*_3′,4′_ = 8.1 Hz, 1H, H-3′), 4.23 (d, *J*_7b,7a_ = 12.0 Hz, 1H, H-7b), 4.19 (m, 1H, H-5), 4.09 (d, *J* = 11.2 Hz, 1H, C4′–OC*H*_2_Ph), 4.03 (s, 3H, H-26′), 3.96 (m, 1H, H-7′a), 3.94 (m, 1H, H-18b), 3.93 (m, 2H, H-3, C4′–OC*H*_2_Ph), 3.80 (d, *J*_1′a,1′b_ = 11.5 Hz, 1H, H-1′a), 3.76 (m, 1H, H-5′), 3.73 (m, 1H, H-7′b), 3.70 (m, 1H, H-4), 3.67 (d, 1H, H-23′b), 3.64 (dd, *J*_2,3_ = 9.6 Hz, 1H, H-2), 3.57 (d, 1H, H-23b), 3.46 (dd, *J*_6a,5_ = 2.7 Hz, *J*_6a,6b_ = 10.4 Hz, 1H, H-6a), 3.43 (d, *J*_1′b,1′a_ = 11.6 Hz, 1H, H-1′b), 3.37 (d, *J*_23″b,23″a_ = 13.6 Hz, 1H, H-23″b), 3.20 (dd, *J*_6b,5_ = 2.2 Hz, 1H, H-6b), 3.19 (s, 3H, H-26″), 3.06 (dd, *J*_4′,3′_ = 9.1 Hz, *J*_4′,5′_ = 9.2 Hz, 1H, H-4′), 3.02 (s, 3H, H-26), 2.95 (dd, *J*_6′a,5′_ = 8.7 Hz, *J*_6′a,6′b_ = 11.9 Hz, 1H, H-6′a), 2.25 (d, 1H, H-6′b) ppm. ^13^C{^1^H} NMR (150 MHz, CDCl_3_): δ = 151.1 (C-20″), 148.0 (C-20′), 147.6 (C-20), 147.1 (C-19), 146.9 (C-19′), 144.4 (C-19″), 139.1, 139.1, 138.3, 138.2, 138.1 (C_quat_, 5 × C-Ph), 136.6 (C-8″), 136.5 (C-8′), 135.6 (C-22″), 135.4 (C-8), 135.2 (C-13″), 134.2 (C-13), 133.0 (C-22′), 132.8 (C-15), 132.8 (C-10″), 132.8 (C-10), 132.5 (C-15″), 132.4 (C-24′), 132.2 (C-10′), 131.6 (C-24″), 131.4 (C-24), 131.1 (C-15′), 131.1 (C-13′), 130.9 (C-22), 129.9 (C-14′), 128.8 (C-11″), 128.5 (C-9), 128.2 (C-16), 128.2 (C-17), 128.1 (C-14), 128.0 (C-12′), 128.0 (C-11), 127.6 (C-16″), 127.2 (C-12), 127.2–128.3 (m, 25 × C-Ph), 126.9 (C-16′), 126.4 (C-11′), 125.3 (C-17″), 125.1 (C-9″), 124.9 (C-17′), 124.1 (C-14″), 124.1 (C-25″), 123.7 (C-12″), 123.1 (C-9′), 113.8 (C-25′), 113.7 (C-21′), 112.9 (C-21″), 112.4 (C-25), 111.8 (C-21), 103.0 (C-2′), 88.9 (C-1), 83.4 (C-3′), 82.4 (C-3), 81.8 (C-4′), 79.5 (C-2), 77.9 (C-4), 77.6 (C-5′), 77.0 (C-18′), 75.4 (C3–O*C*H_2_Ph), 74.8 (C-1′), 74.6 (C4–O*C*H_2_Ph), 74.2 (C-7″), 72.8 (C-7), 72.7 (C-6′), 72.6 (C2–O*C*H_2_Ph), 72.5 (C4′–O*C*H_2_Ph), 72.4 (C3′–O*C*H_2_Ph), 71.6 (C-7′), 70.6 (C-18), 69.4 (C-5), 69.4 (C-18″), 65.9 (C-6), 56.4 (C-26′), 55.4 (C-26″), 54.5 (C-26), 36.6 (C-23), 36.2 (C-23″), 36.0 (C-23′) ppm. HRMS (ESI-TOF) *m*/*z*: [M + Na]^+^ calcd for C_107_H_100_O_17_Na 1679.6858; found 1679.6860. Anal. calcd for C_107_H_100_O_17_ + H_2_O: C, 76.68; H, 6.13; found: C, 76.62; H, 6.22.

### ECD Spectra

ECD spectra were measured in acetonitrile at room temperature. All spectra were collected between 180–400 nm at room temperature using solutions at concentrations 2.5 × 10^–5^ M in quartz cells with path length 0.2 or 0.5 cm. All spectra were recorded using a 100 nm/min scanning speed, a step size of 0.2 nm, a bandwidth of 1 nm, a response time of 0.5 s, and an accumulation of 3 scans. The spectra were background corrected using acetonitrile.

### Computational Details

Conformational search was carried out at the molecular mechanics level using a simplified structure in which all benzyl groups (Bn) in a sucrose moiety were exchanged by a hydrogen atom to save computational time and facilitate the computational predictions of ECD spectra for all investigated diastereoisomers. Next, the lowest energy structures (#10) within 3 kcal/mol were submitted for DFT optimization using Gaussian16 program^38^ at the B3LYP/6-31G(d) level of theory applying PCM for CH_3_CN. In each case, structures were confirmed to contain no imaginary frequencies. Finally, for the most abundant structures (#2), TDDFT calculations were carried out using the B3LYP and CAM-B3LYP functionals with SVP basis set using the PCM model for CH_3_CN. Since they gave fully coherent results, we are only presenting results from the B3LYP functional and SVP basis set. Other basis sets, *i.e.*, TZVP, 6-311+G(d,p), were also checked for improving consistency of the obtained results. The UV and ECD spectra are simulated by overlapping Gaussian functions for 350 transitions. An optimum Gaussian band-shape and UV-correction were selected according to the similarity analysis with experimental data in CH_3_CN performed using SpecDis.^43^ The ***M*-5a⊂ACh** and ***M*-5a⊂Ch** encapsulated complexes were optimized at the B3LYP/6-31G(d) level of theory using Gaussian16 program.^38^

#### Fluorescence Titration Experiments

The stock solutions of the hosts ***P*-5a**, ***P*-5b**, ***M*-5a**, and ***M*-5b** were prepared at concentrations *ca.* 0.001 M in acetonitrile. A volume of 2.5 mL of acetonitrile was taken to the quartz cuvette, and the appropriate amount of host stock solution was added to obtain concentrations between 2.26 and 2.44 × 10^–5^ M. The guest solutions were prepared by dissolving the required amount of acetylcholine iodide or choline iodide in a host stock solution to provide constant host concentration during the titration studies. Portions of the guest solution were gradually added to the cuvette containing appropriate host solution, mixed, and incubated for 30 s before irradiation at 280 nm at 25 °C. The corresponding emission spectra during titration were recorded. The measured emission spectra for the host during the titration studies were plotted as a function of the guest/host ratio using nonlinear regression *via* Bindfit program.^40^ The value of association constant *K*_a_ was calculated by nonlinear least-squares using as input parameters 1:1 binding model and the Nelder–Mead method.

#### ^1^H NMR Titration Experiments

The ^1^H NMR titration experiments were conducted by measuring the ^1^H NMR spectra at 400 MHz with a Bruker Avance II apparatus at 303 K. The solutions of the hosts ***P*-5a** and ***M*-5a** were prepared at concentrations 3.73 × 10^–3^ and 1.78 × 10^–3^ M, respectively, in the mixture of solvents CD_3_CN/CDCl_3_ = 80:20. The guest solutions were prepared by dissolving the required amount of acetylcholine iodide or choline iodide salts in a host stock solution to ensure constant host concentration during the experiment. Next, to the NMR tube containing appropriate host solution, portions of the guest solution were gradually added and the ^1^H NMR spectrum was recorded.

## Supporting Information Available

Table with comparison of ^1^H and ^13^C NMR signals of ***P*-5a**, ***M*-5a**, ***P*-5b**, and ***M*-5b**; fluorescence titration data; Cartesian coordinates of calculated structures; and copies of NMR spectra (PDF)
