# Supplementary material for: Fluorescent Molecular Cages with Sucrose and Cyclotriveratrylene Units for the Selective Recognition of Choline and Acetylcholine
Source: J Org Chem. 2021 Mar 12;86(7):5129–41. doi: 10.1021/acs.joc.1c00019 (PMC8041319; doi:10.1021/acs.joc.1c00019)
Supplement: Supplementary file 1 — jo1c00019_si_001.pdf [file jo1c00019_si_001.pdf]

Supporting Information for

Fluorescent Molecular Cages with Sucrose and Cyclotriveratrylene Units for the Selective Recognition of Choline and Acetylcholine

Łukasz Szyszka,* Marcin Górecki, Piotr Cmoch, and Sławomir Jarosz*

Institute of Organic Chemistry, Polish Academy of Sciences, Kasprzaka 44/52,

01-224 Warsaw, Poland

Table of Contents

Table S1. Comparison of ^{1}H and ^{13}C NMR signals of ***P*-5a**,***P*-5b**, ***M*-5a,** and ***M*-5b**. S3
Figure S1-S12. Fluorescence titration experiments.. S5
Table S2. Fit parameters... S9
Figure S13-S22. ^{1}H NMR titration experiments.. S9
Figure S23-S71. NMR spectra S15
Figure S72-S80. MS spectra S64
Geometry of calculated structures S73

Table S1. Comparison of ^{1}H and ^{13}C NMR signals of ***P*-5a**, ***P*-5b**, ***M*-5a,** and ***M*-5b**.

	**^{1}H and ^{13}C NMR chemical shifts δ (ppm)**			
**Glucose part**	***P*-5a**	***P*-5b**	***M*-5a**	***M*-5b**
H-1/C-1	5.19/90.7	5.55/88.7	5.60/91.4	5.54/88.9
H-2/C-2	3.30/79.9	3.61/79.3	3.38/79.7	3.64/79.5
H-3/C-3	3.79/81.6	3.92/82.3	3.72/81.4	3.93/82.4
H-4/C-4	3.56/77.5	3.57/78.0	3.41/77.7	3.70/77.9
H-5/C-5	3.81/70.6	4.13/69.9	3.90/70.4	4.19/69.4
H-6a+H-6b/C-6	3.17+3.53/68.1	3.26+3.31/67.6	3.06+3.39/67.9	3.20+3.46/65.9
**Fructose part**				
H-1'a+H-1'b/C-1'	3.45+3.65/69.9	3.58+3.71/74.8	3.66+3.72/69.1	3.43+3.80/74.8
C-2'	104.1	104.0	104.6	103.0
H-3'/C-3'	4.38/84.1	4.24/82.5	4.35/84.9	4.29/83.4
H-4'/C-4'	3.83/82.4	3.53/81.3	4.03/83.1	3.06/81.8
H-5'/C-5'	4.07/80.9	3.32/79.2	3.92/80.8	3.76/77.6
H-6'a+H-6'b/C-6'	3.36+3.54/73.0	2.84+3.37/70.9	3.46+3.49/73.1	2.25+2.95/72.7
**Naphthalene linkers**				
H-7a+H-7b/C-7	4.15+4.45/73.1	4.23+4.78/73.0	4.04+4.64/73.4	4.23+4.94/72.8
H-7'a+H-7'b/C-7'	4.30+4.46/72.5	3.95+4.21/72.6	4.34/72.3	3.73+3.96/71.6
H-7"a+H-7"b/C-7"	4.27+4.76/73.0	4.58+4.66/74.4	4.50+5.07/73.9	4.59+4.67/74.2
C-8	135.9	135.3	135.4	135.4
C-8'	136.9	136.1	136.3	136.5
C-8"	135.8	136.5	135.9	136.6
H-9/C-9	7.47/126.1	7.62/127.0	7.36/126.7	7.78/128.5
H-9'/C-9'	7.51/124.5	7.47/126.0	7.40/124.4	7.16/123.1
H-9"/C-9"	7.64/126.2	7.49/125.5	7.83/127.4	7.62/125.1
C-10	132.78/132.0	132.6	nd	132.8
C-10'	132.6	132.4	nd	132.2
C-10"	132.6	132.82/132.3	nd	132.8
H-11/C-11	7.51/127.8	7.65/127.8	7.30/127.8	7.67/128.0
H-11'/C-11'	7.46/126.1	7.55/127.6	7.44/127.2	7.18/126.4
H-11"/C-11"	7.65/128.1	7.23/125.3	7.76/128.4	7.49/128.8
H-12/C-12	7.16/125.4	7.27/125.3	7.21/125.9	7.14/127.2
H-12'/C-12'	7.11/125.6	7.32/125.8	7.17/126.6	7.12/128.0
H-12"/C-12"	7.35/124.8	6.87/125.4	7.38/125.5	7.32/123.7
C-13	134.2	135.1	134.3	134.2
C-13'	133.3	133.5	133.6	131.1
C-13"	135.3	134.7	134.9	135.2
H-14/C-14	7.46/126.3	7.57/125.9	7.45/126.6	7.58/128.1
H-14'/C-14'	6.94/126.8	7.06/126.3	6.84/128.2	6.55/129.9
H-14"/C-14"	7.77/125.4	7.56/126.3	7.73/126.3	7.70/124.1
C-15	132.78/132.0	132.8	nd	132.8
C-15'	132.5	132.2	nd	131.1
C-15"	132.6	132.82/132.3	nd	132.5
H-16/C-16	7.39/127.7	7.55/128.2	7.23/128.0	7.69/128.2
H-16'/C-16'	6.68/127.7	6.79/127.6	6.98/127.9	6.14/126.9
H-16"/C-16"	7.58/127.9	7.52/127.7	7.79/128.0	7.49/127.6
H-17/C-17	7.02/126.2	7.38/126.5	7.02/126.3	7.52/128.2
H-17'/C-17'	6.88/125.6	6.91/126.3	7.01/125.0	6.49/124.9
H-17"/C-17"	7.36/126.3	7.04/125.4	7.48/127.2	7.21/125.3
H-18a+H-18b/C-18	4.81+5.24/71.4	4.87+5.09/71.3	5.31+5.40/70.2	3.94+4.87/70.6
H-18'a+H-18'b/C-18'	5.15+5.26/72.6	5.35+5.46/72.3	4.40+5.20/76.2	5.00+5.35/77.0
H-18"a+H-18"b/C-18"	5.15+5.36/72.7	4.94+5.27/73.9	4.97+5.21/71.3	5.45+5.64/69.4
**CTV-ring A**				
C-19	146.1	147.3	145.8	147.1
C-20	148.5	148.4	148.6	147.6
H-21/C-21	6.64/113.0	6.70/113.5	6.99/113.8	6.54/111.8
C-22	132.7	132.8	133.2	130.9

H-23a+H-23b/C-23	3.51+4.70/36.4	3.55+4.77/36.3	3.61+4.79/36.2	3.57+4.74/36.6
C-24	131.6	131.6	131.9	131.4
H-25/C-25	6.86/116.3	6.88/116.4	7.09/116.7	6.42/112.4
H-26/C-26 (OCH_3)	3.40/55.5	3.60/56.0	3.92/56.3	3.02/54.5
**CTV-ring B**				
C-19’	145.9	147.6	146.8	146.9
C-20’	149.1	149.1	150.3	148.0
H-21’/C-21’	6.67/113.4	6.81/114.4	6.64/113.5	7.19/113.7
C-22’	133.7	133.6	134.7	133.0
H-23’a+H-23’b/C-23’	3.47+4.70/36.4	3.57+4.77/36.6	3.51+4.74/36.5	3.67+4.79/36.0
C-24’	131.7	132.5	132.1	132.4
H-25’/C-25’	6.95/118.6	7.09/118.5	7.12/121.9	7.13/113.8
H-26’/C-26’ (OCH_3)	3.55/55.9	3.44/56.2	3.22/55.5	4.03/56.4
**CTV-ring C**				
C-19”	147.0	145.3	147.3	144.4
C-20”	148.8	149.7	148.0	151.1
H-21”/C-21”	6.61/113.4	6.86/113.9	6.93/115.0	6.33/112.9
C-22”	133.3	134.0	131.8	135.6
H-23”a+H-23”b/C-23”	3.48+4.68/36.2	3.59+4.77/36.5	3.51+4.74/36.4	3.37+4.71/36.2
C-24”	132.0	132.3	132.4	131.6
H-25”/C-25”	6.99/117.6	7.07/120.0	6.76/113.3	7.34/124.1
H-26”/C-26” (OCH_3)	3.21/55.5	3.73/56.3	3.48/55.7	3.19/55.4

Fluorescence titration experiments

Fluorescence titration curves for acetylcholine iodide

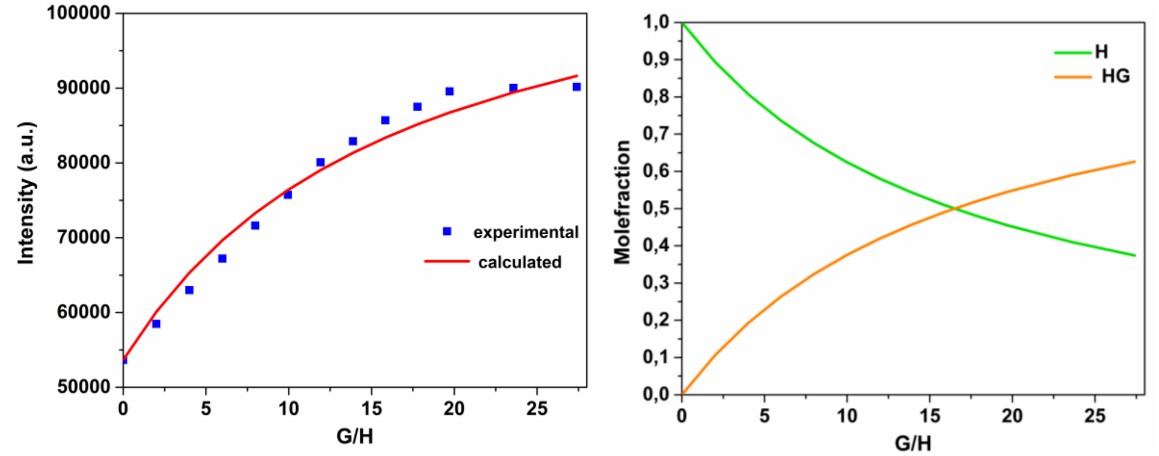

FigureS1. Fluorescence intensity and molefraction as a functions of guest/host ratio for cage ***P*-5a.**

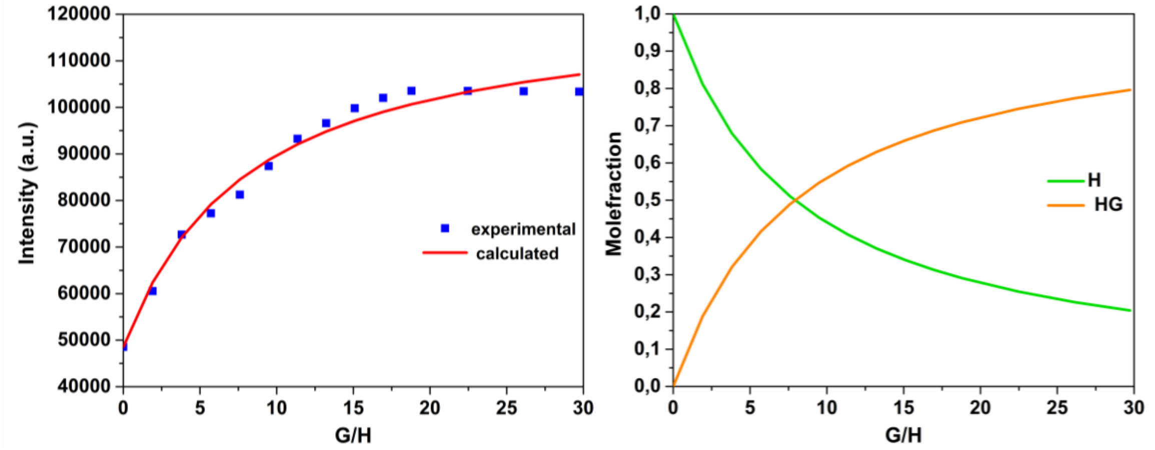

FigureS2. Fluorescence intensity and molefraction as a functions of guest/host ratio for cage ***M*-5a.**

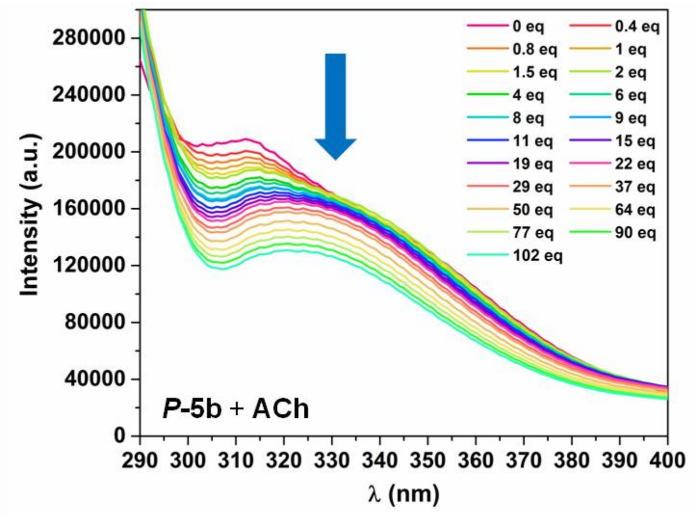

FigureS3. Fluorescence titration of host ***P*-5b** with ACh in CH_3CN excited at 280 nm.

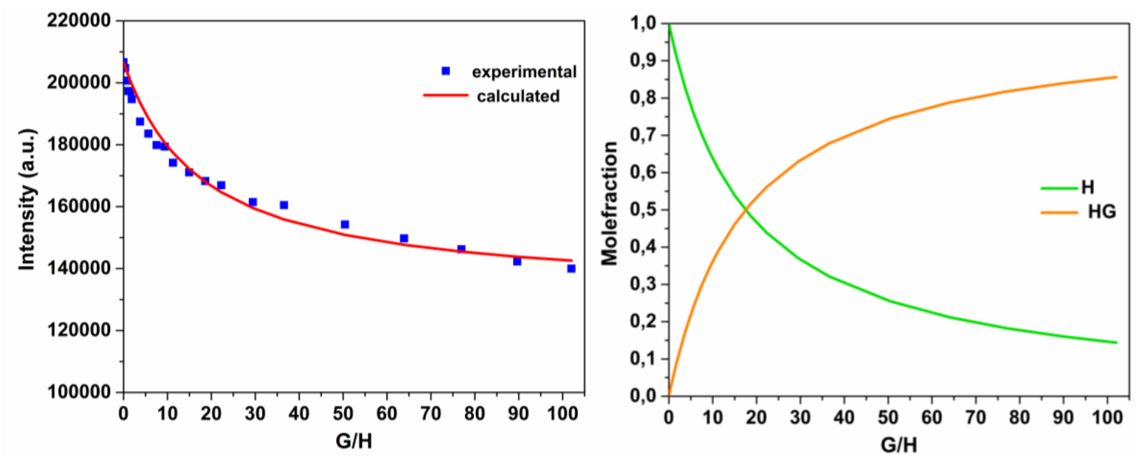

FigureS4. Fluorescence intensity and molefraction as a functions of guest/host ratio for cage ***P*-5b.**

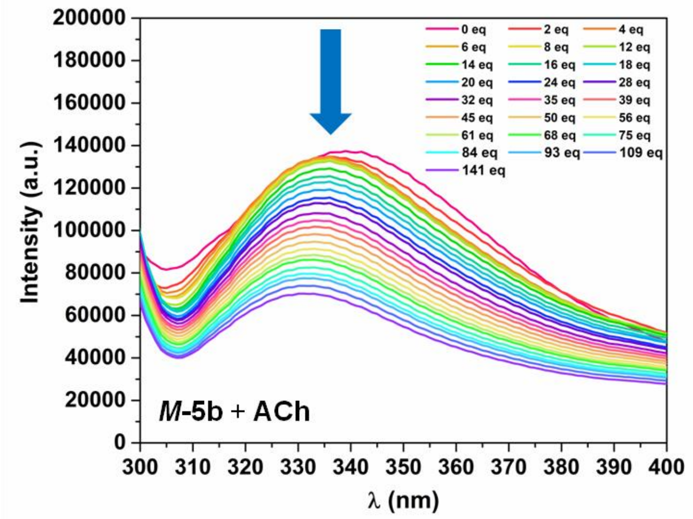

Figure S5. Fluorescence titration of host ***M*-5b** with ACh in CH_3CN excited at 280 nm.

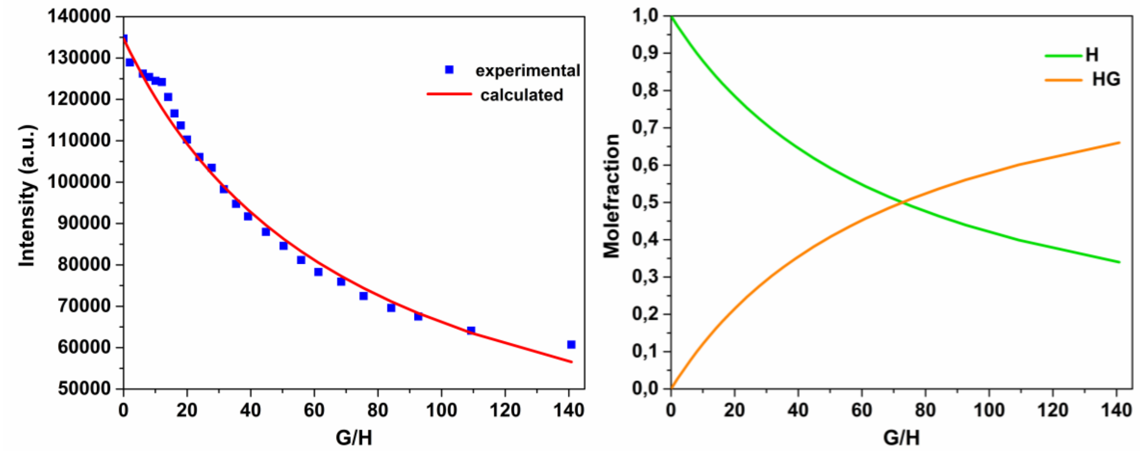

Figure S6. Fluorescence intensity and molefraction as a functions of guest/host ratio for cage ***M*-5b.**

Fluorescence titration curves for choline iodide

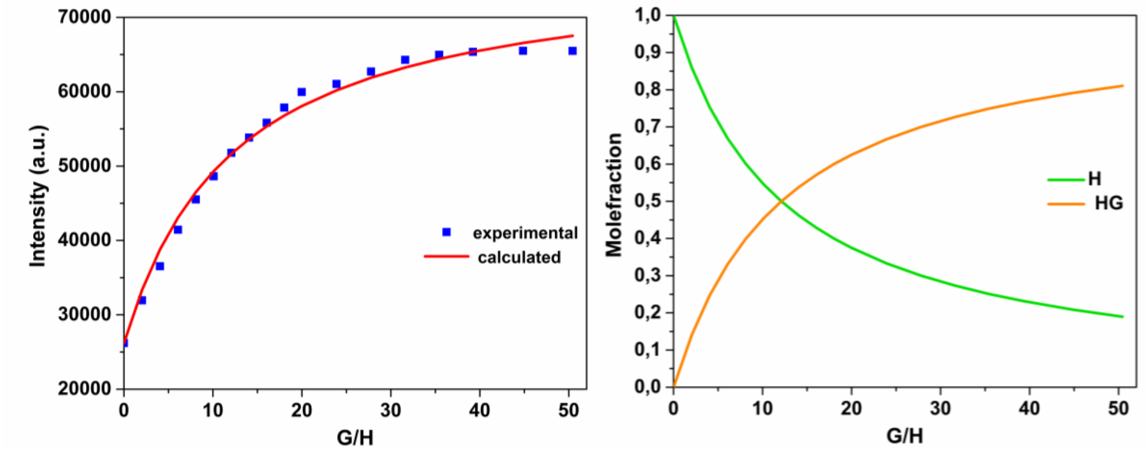

Figure S7. Fluorescence intensity and molefraction as a functions of guest/host ratio for cage ***P*-5a.**

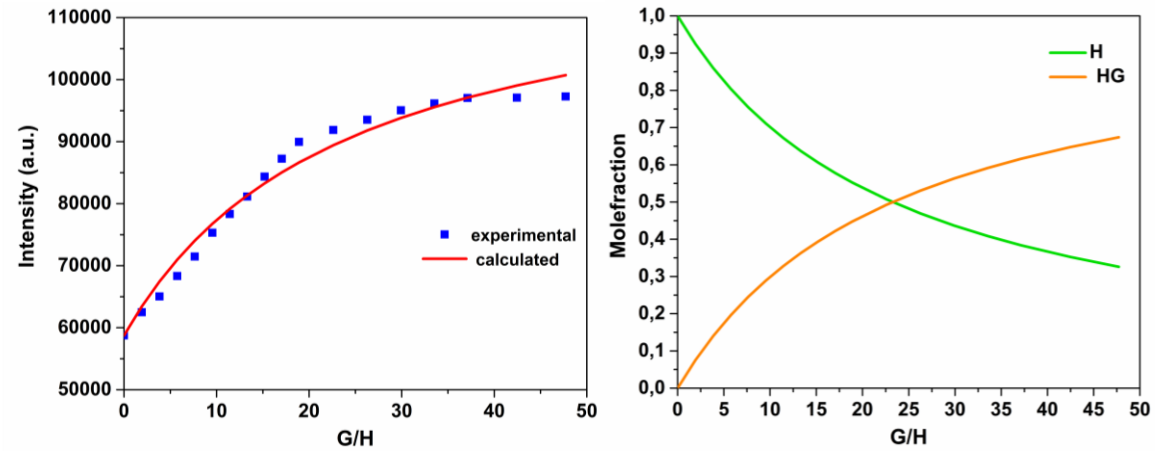

Figure S8. Fluorescence intensity and molefraction as a functions of guest/host ratio for cage ***M*-5a.**

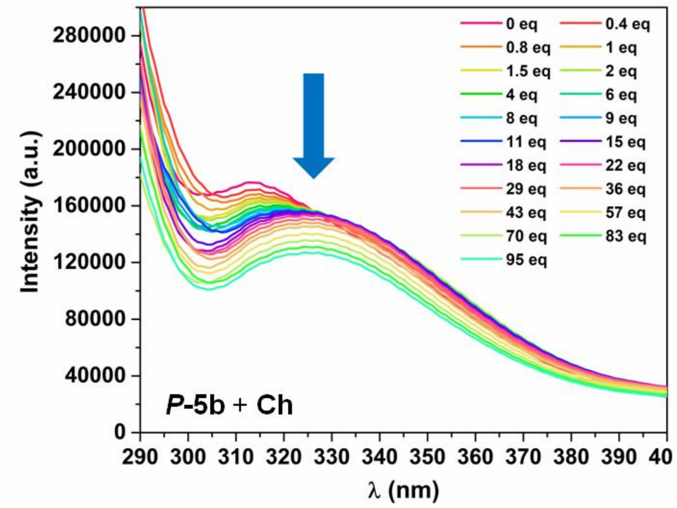

Figure S9. Fluorescence titration of host ***P*-5b** with Ch in CH_3CN excited at 280 nm.

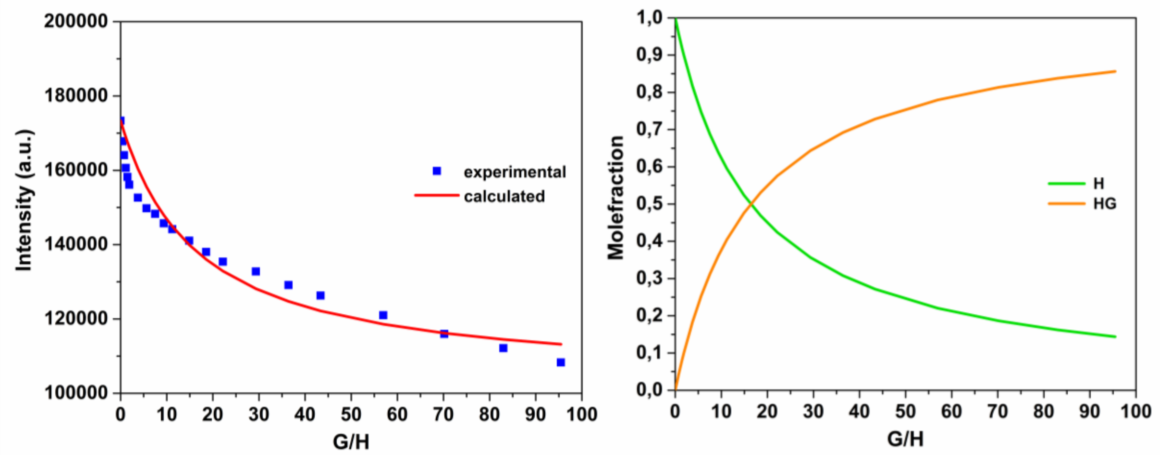

Figure S10. Fluorescence intensity and molefraction as a functions of guest/host ratio for cage ***P*-5b.**

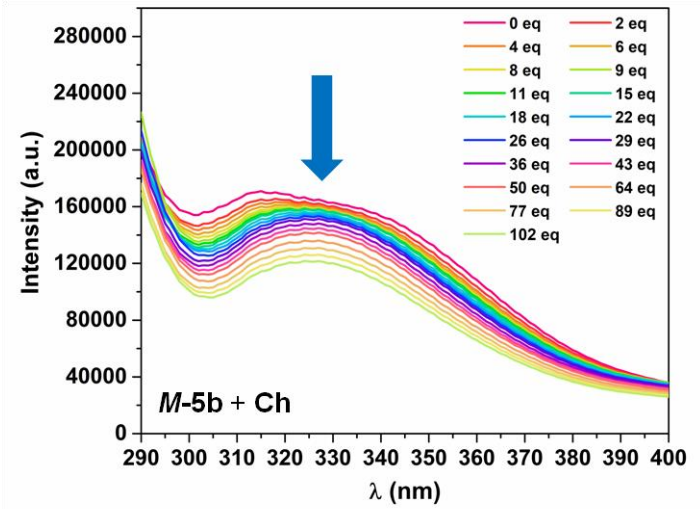

Figure S11. Fluorescence titration of host ***M*-5b** with Ch in CH_3CN excited at 280 nm.

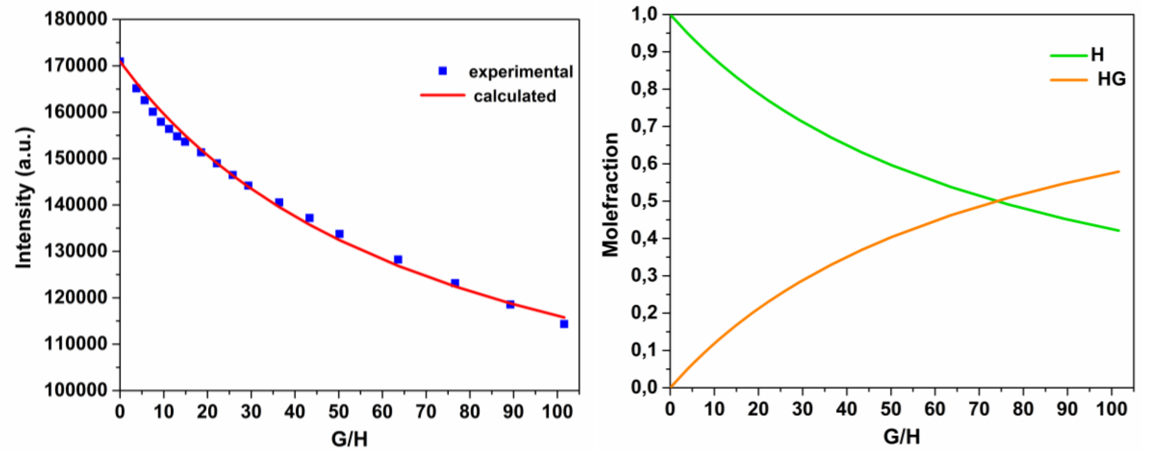

Figure S12. Fluorescence intensity and molefraction as a functions of guest/host ratio for cage ***M*-5b.**

Fit parameters

Table S2. Fit parameters obtained from the fluorescence titrations.

Host	Guest	K_a (M^{-1})	**RMS** (Intensity a.u.)	**Covariance**
***P*-5a**	ACh	$2.2 \times 10^3 \pm 1.6\%$	1.0×10^3	2.1×10^{-2}
	Ch	$3.8 \times 10^3 \pm 0.9\%$	1.6×10^3	8.5×10^{-3}
***M*-5a**	ACh	$5.6 \times 10^3 \pm 1.7\%$	2.2×10^3	1.8×10^{-2}
	Ch	$1.8 \times 10^3 \pm 1.4\%$	1.8×10^3	2.2×10^{-2}
***P*-5b**	ACh	$2.4 \times 10^3 \pm 3.5\%$	6.1×10^3	7.2×10^{-2}
	Ch	$2.6 \times 10^3 \pm 3.8\%$	2.6×10^3	2.8×10^{-2}
***M*-5b**	ACh	$0.6 \times 10^3 \pm 0.9\%$	2.8×10^3	1.7×10^{-2}
	Ch	$0.5 \times 10^3 \pm 1.1\%$	2.6×10^3	2.8×10^{-2}

^{1}H NMR titration experiments

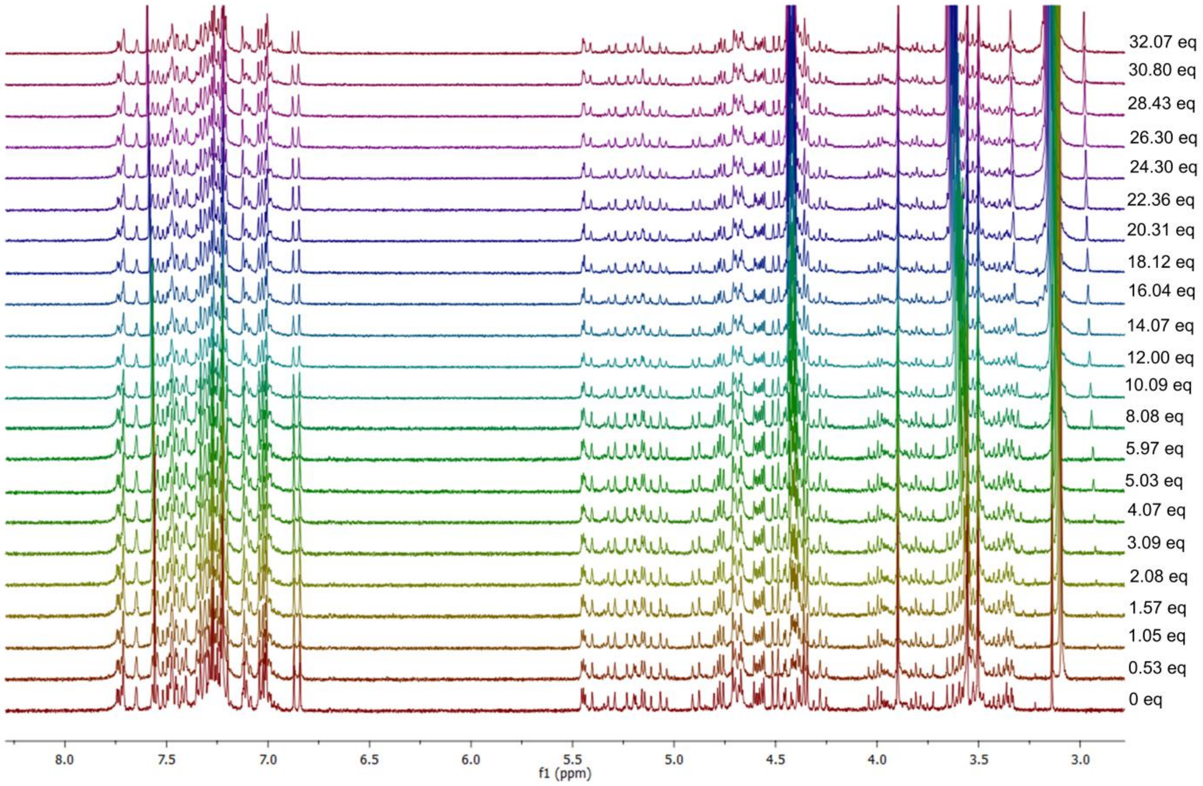

Figure S13. ^{1}H NMR (400 MHz, $CD_3CN/CDCl_3$ = 80:20) spectra of ***M*-5a** after gradual addition of acetylcholine iodide in the same solvent**.**

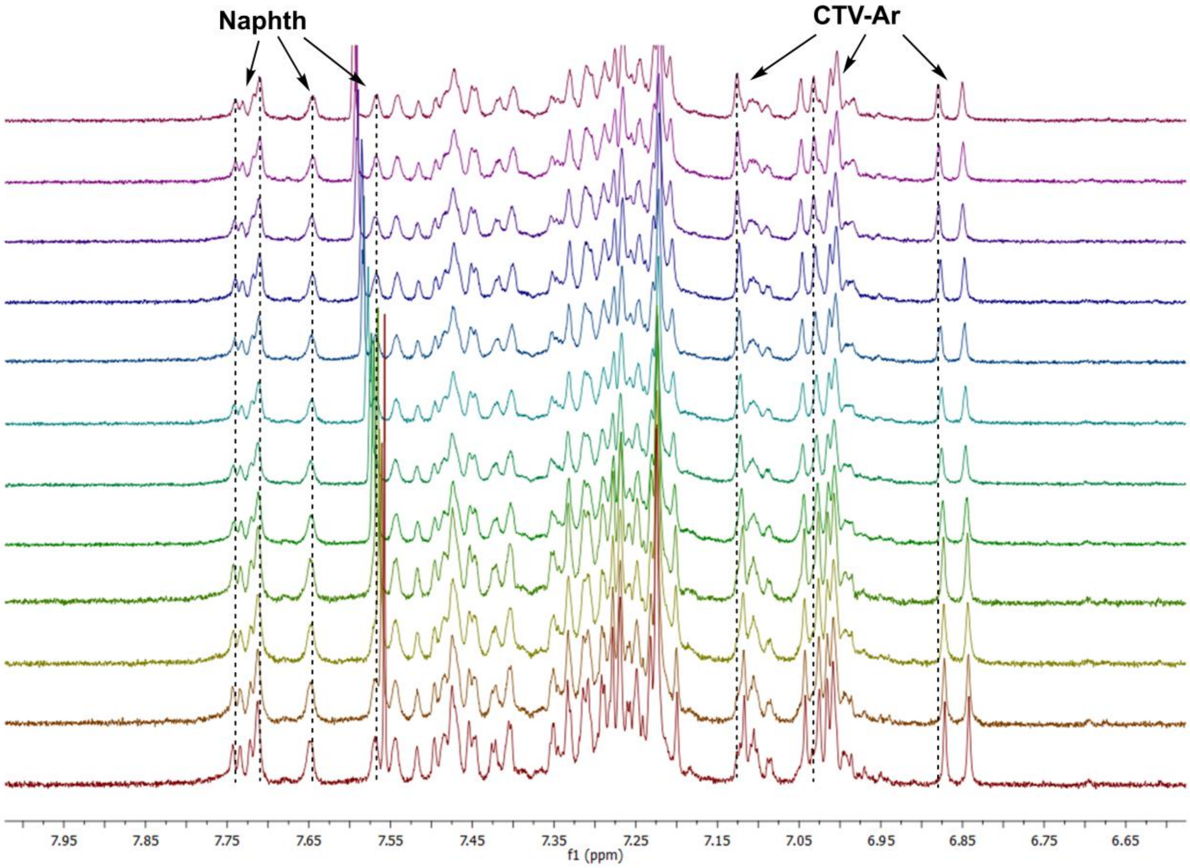

Figure S14. ^{1}H NMR (400 MHz, $CD_3CN/CDCl_3$ = 80:20) spectra of aromatic part of ***M*-5a** after gradual addition of acetylcholine iodide in the same solvent**.**

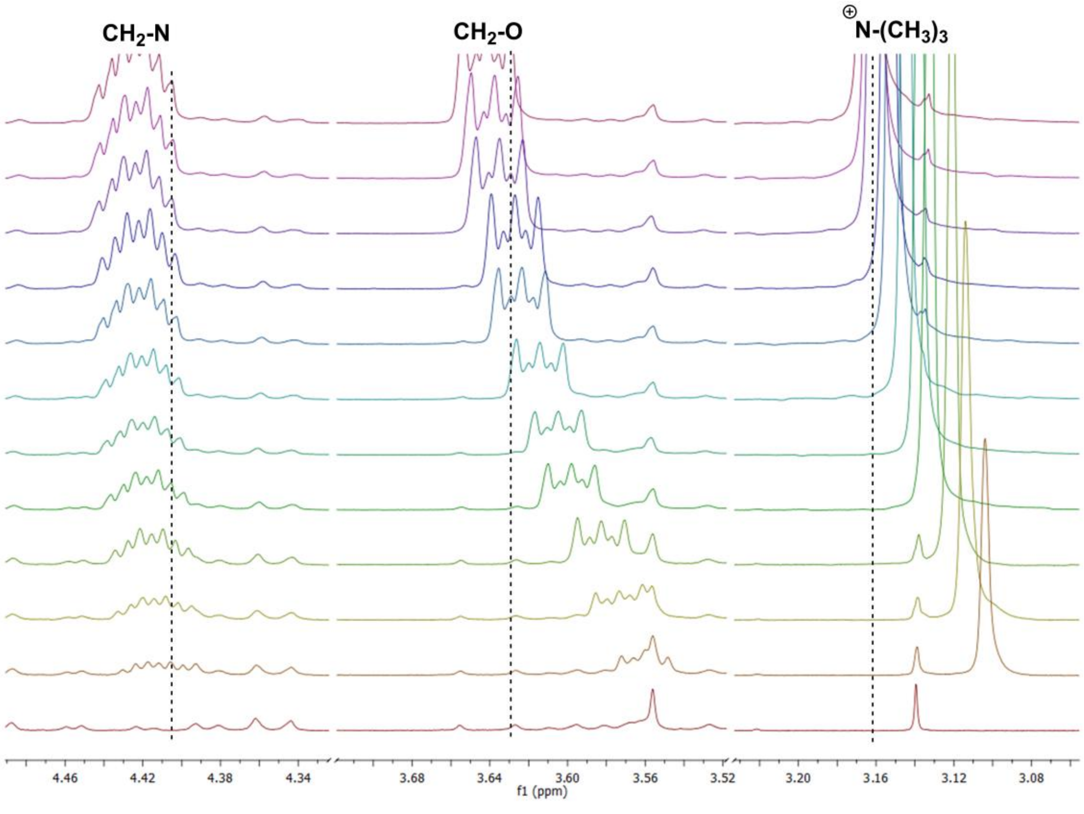

Figure S15. ^{1}H NMR (400 MHz, $CD_3CN/CDCl_3$ = 80:20) spectra of ACh chemical shifts during titration studies of ***M*-5a** with acetylcholine iodide**.**

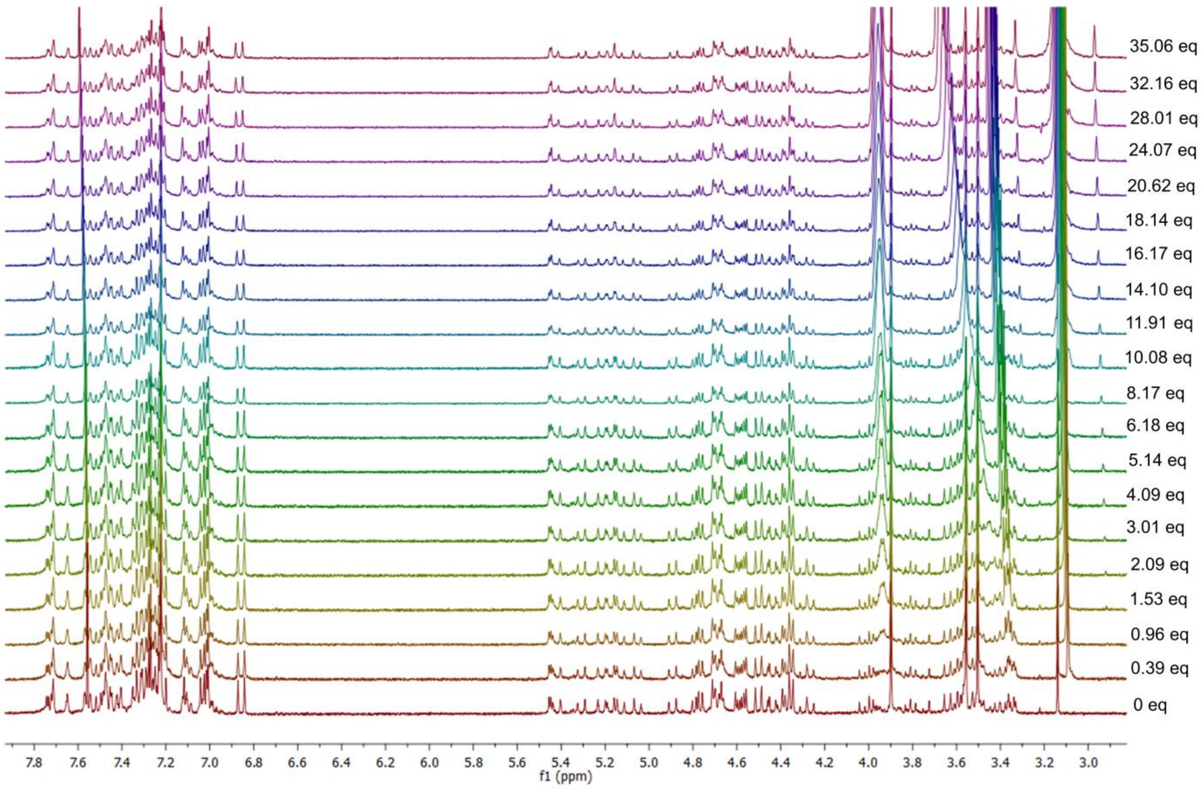

Figure S16. ^{1}H NMR (400 MHz, $CD_3CN/CDCl_3$ = 80:20) spectra of ***M*-5a** after gradual addition of choline iodide in the same solvent**.**

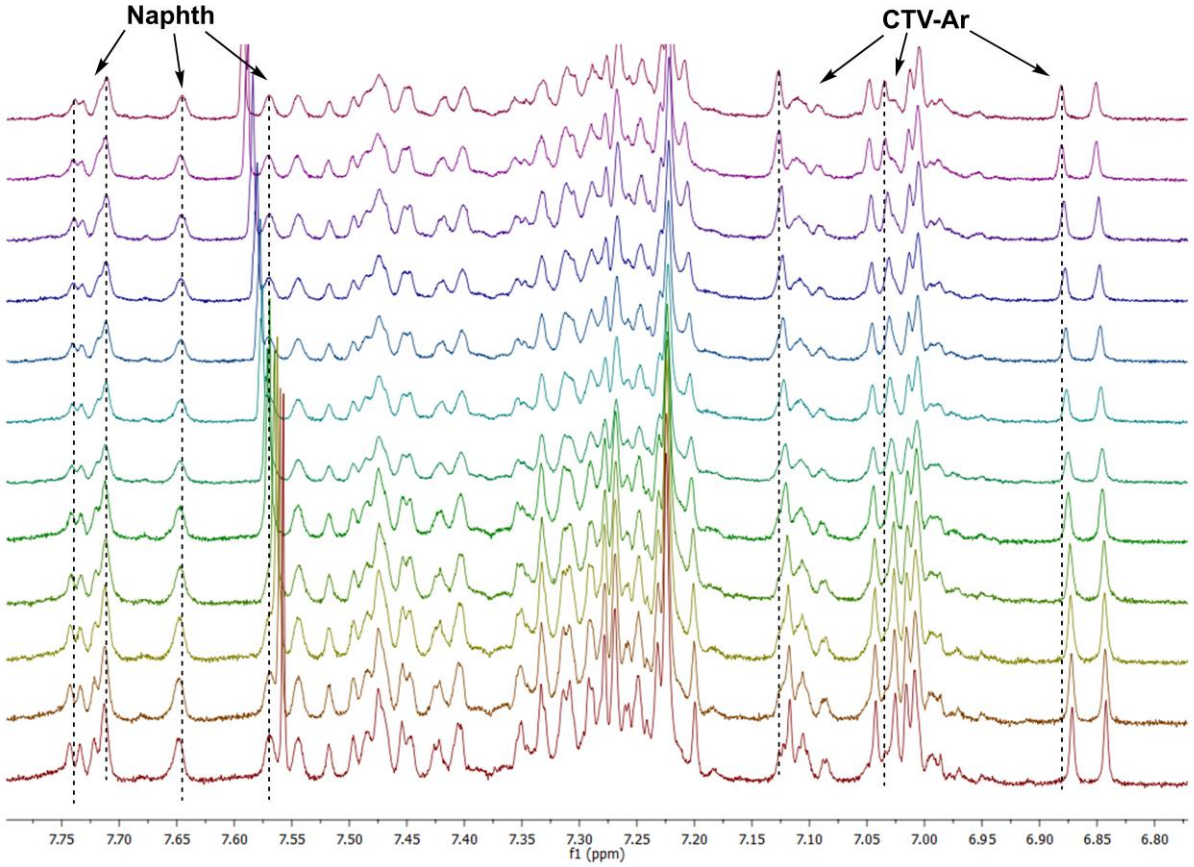

Figure S17. ^{1}H NMR (400 MHz, $CD_3CN/CDCl_3$ = 80:20) spectra of aromatic part of ***M*-5a** after gradual addition of choline iodide in the same solvent**.**

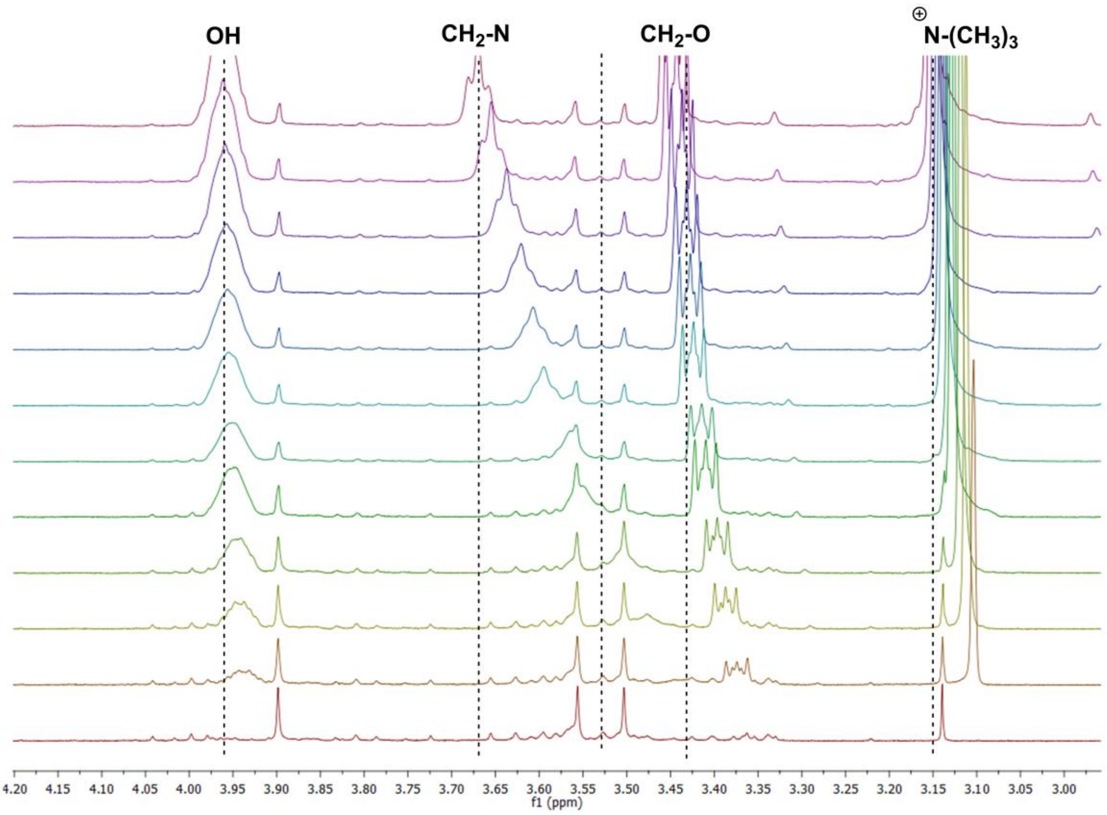

Figure S18. ^{1}H NMR (400 MHz, $CD_3CN/CDCl_3$ = 80:20) spectra of Ch chemical shifts during titration studies of ***M*-5a** with choline iodide**.**

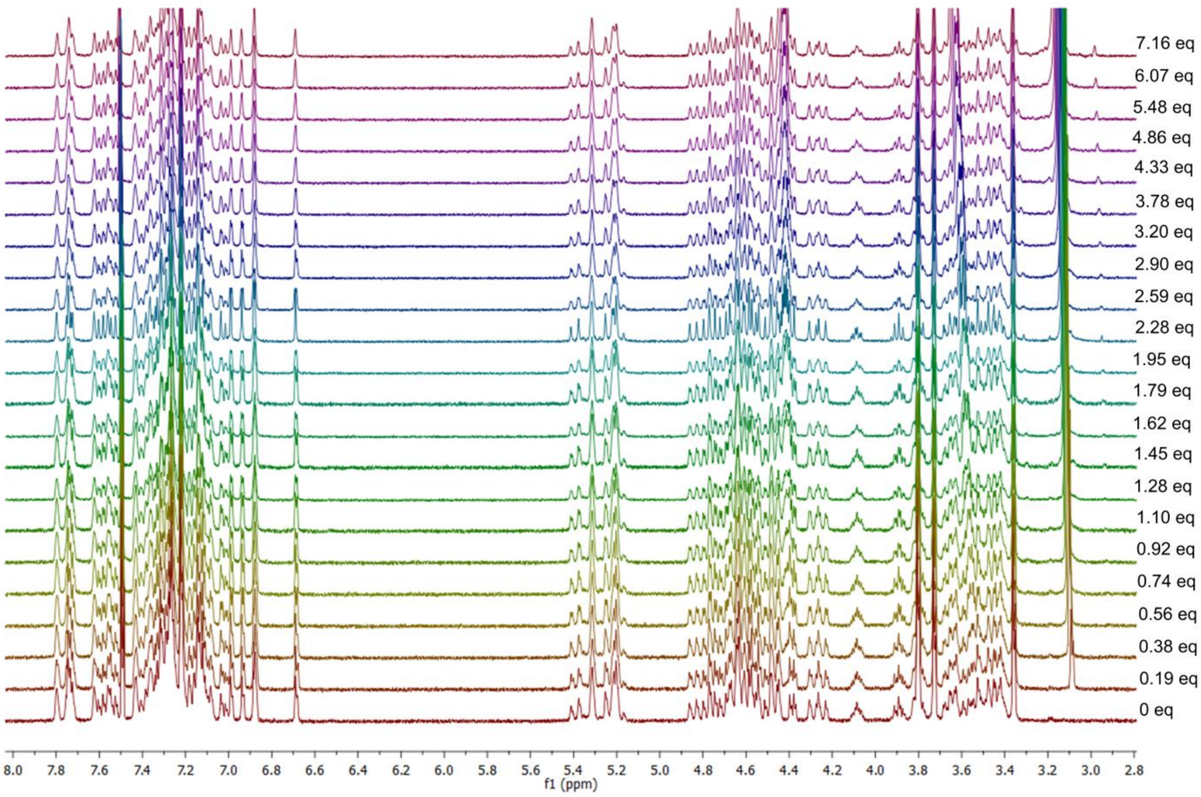

Figure S19. ^{1}H NMR (400 MHz, $CD_3CN/CDCl_3$ = 80:20) spectra of ***P*-5a** after gradual addition of acetylcholine iodide in the same solvent**.**

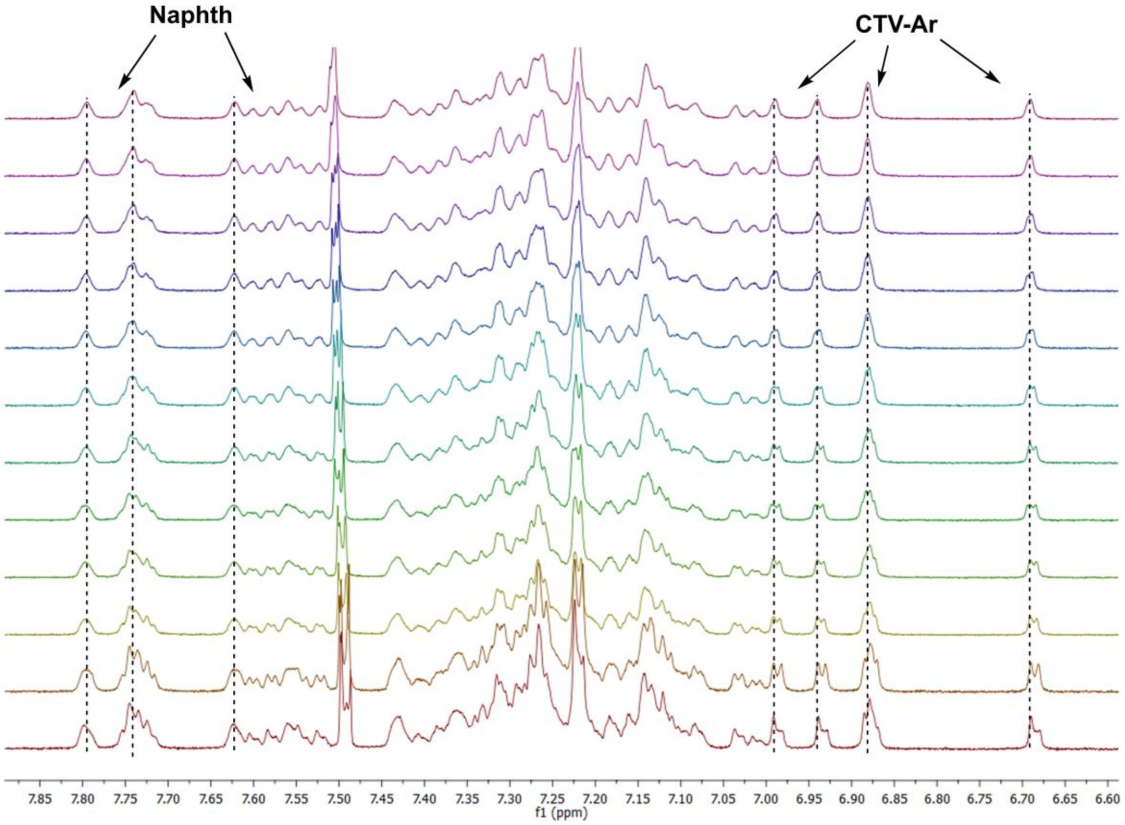

Figure S20. ^{1}H NMR (400 MHz, $CD_3CN/CDCl_3$ = 80:20) spectra of aromatic part of ***P*-5a** after gradual addition of acetylcholine iodide in the same solvent**.**

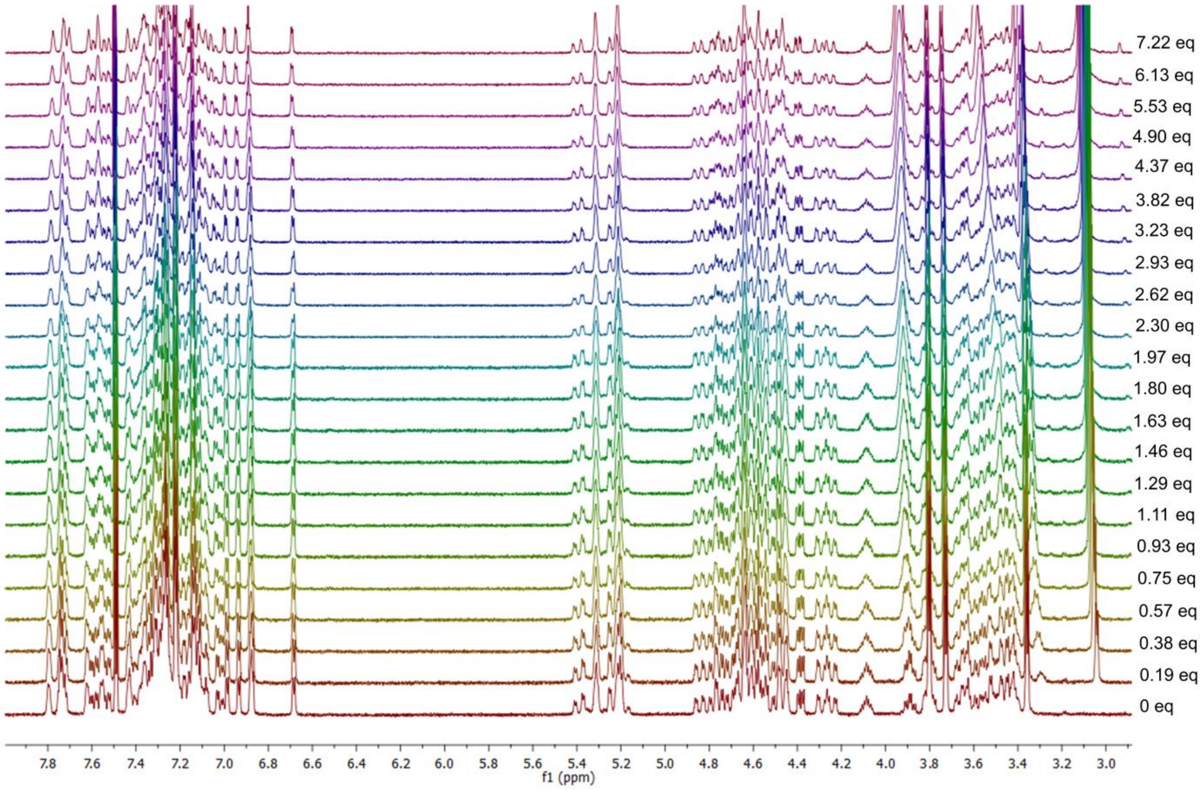

Figure S21. ^{1}H NMR (400 MHz, $CD_3CN/CDCl_3$ = 80:20) spectra of ***P*-5a** after gradual addition of choline iodide in the same solvent**.**

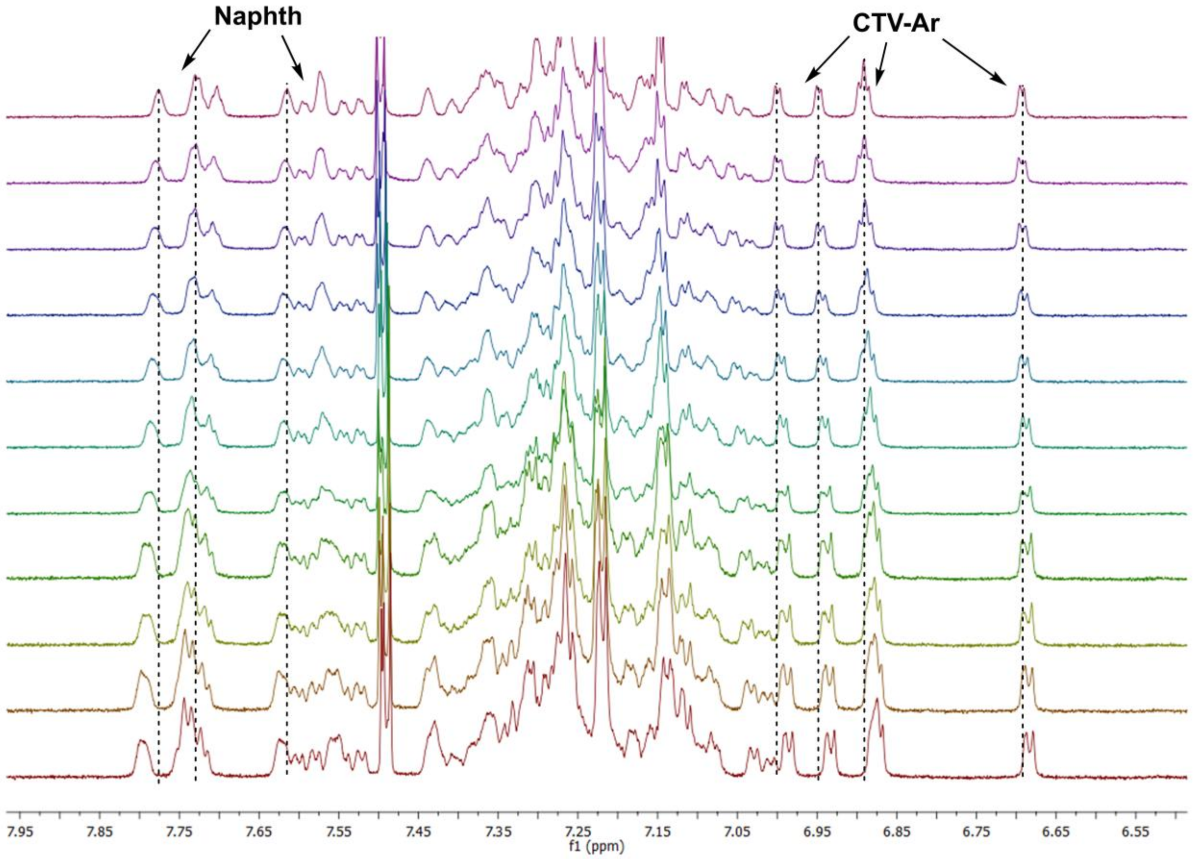

Figure S22. ^{1}H NMR (400 MHz, $CD_3CN/CDCl_3$ = 80:20) spectra of aromatic part of ***P*-5a** after gradual addition of choline iodide in the same solvent**.**

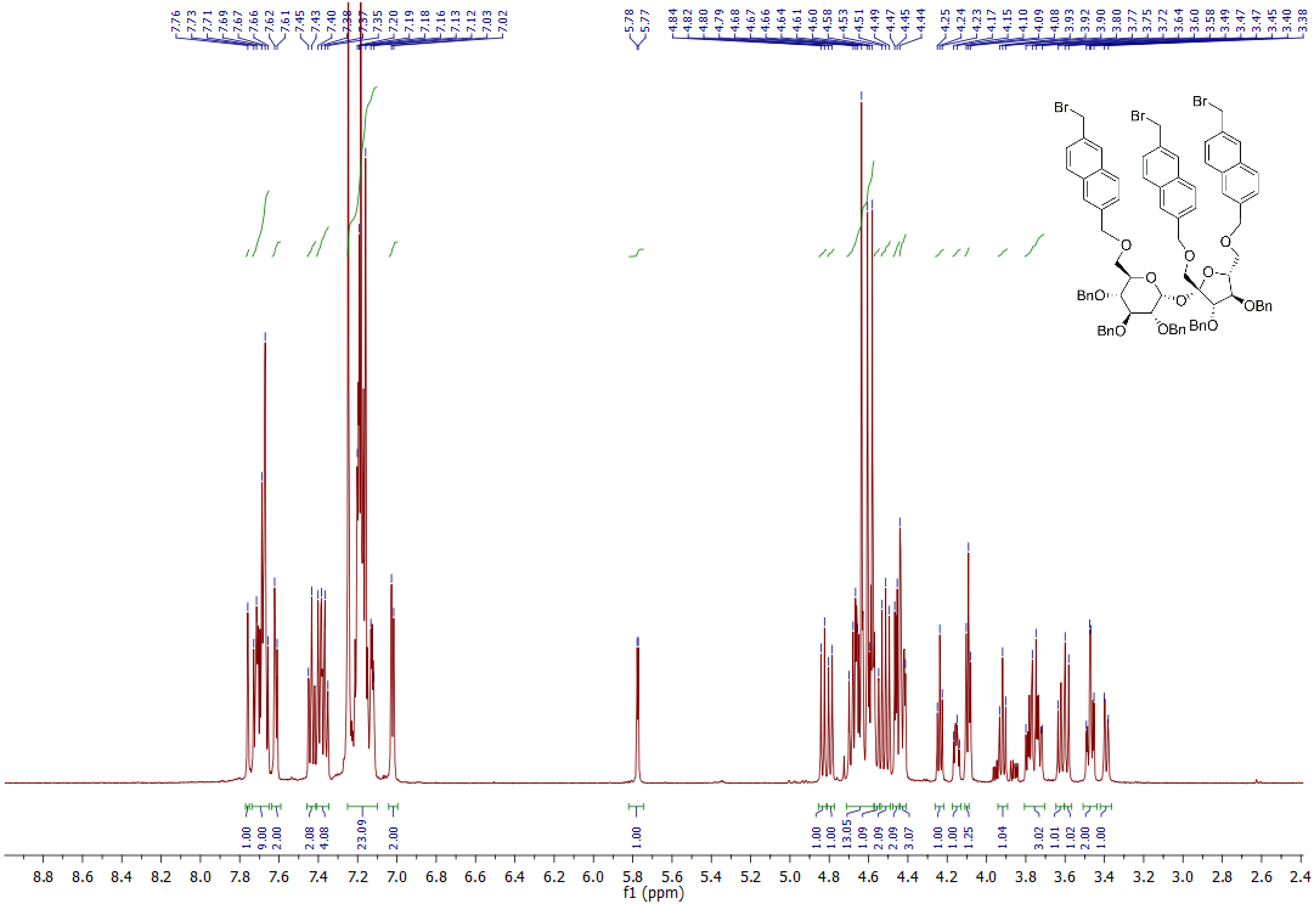

Figure S23. ^{1}H NMR (600 MHz, $CDCl_3$) spectrum of compound **3.**

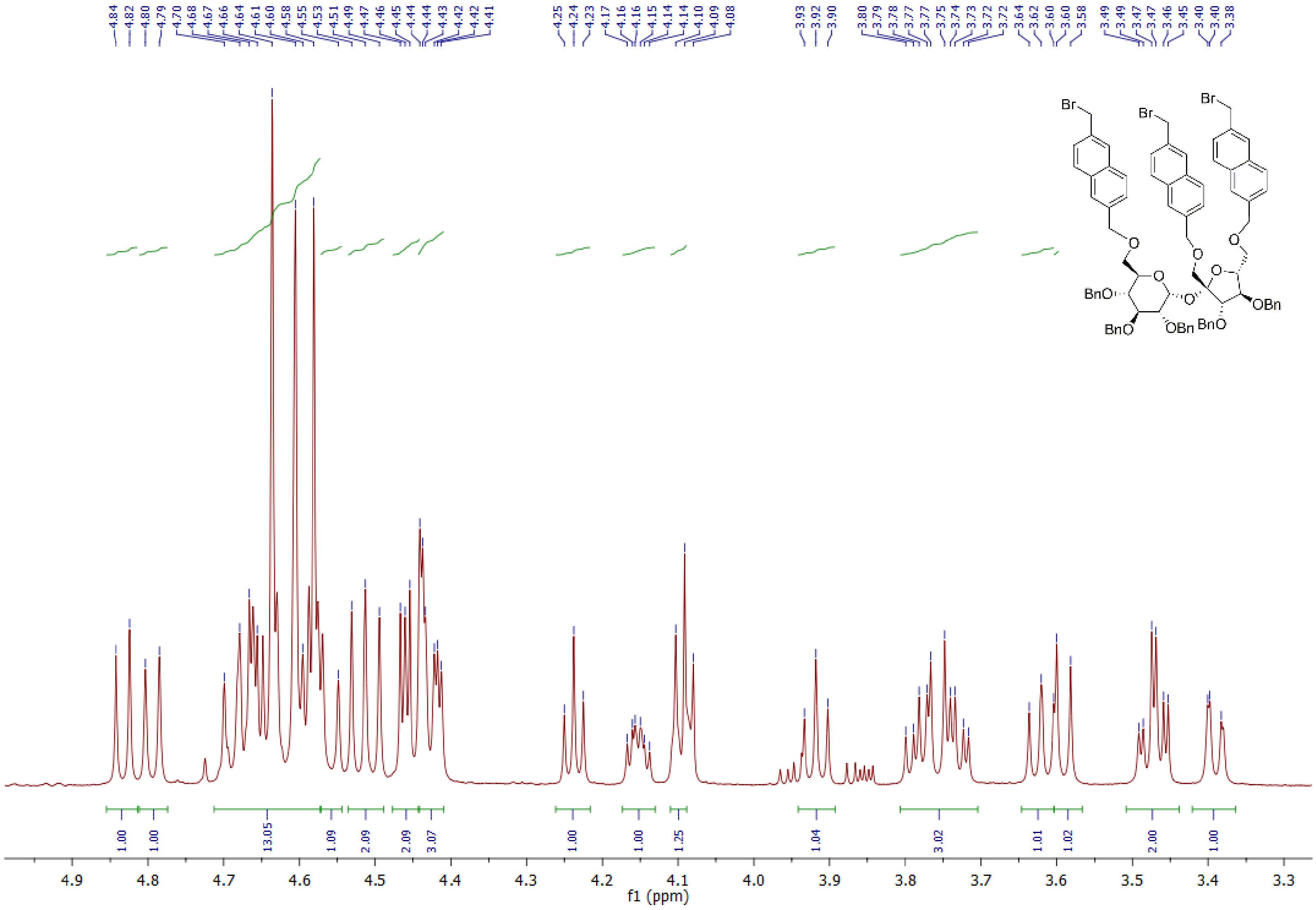

Figure S24. ^{1}H NMR (600 MHz, $CDCl_3$) spectrum of compound **3** (aliphatic part).

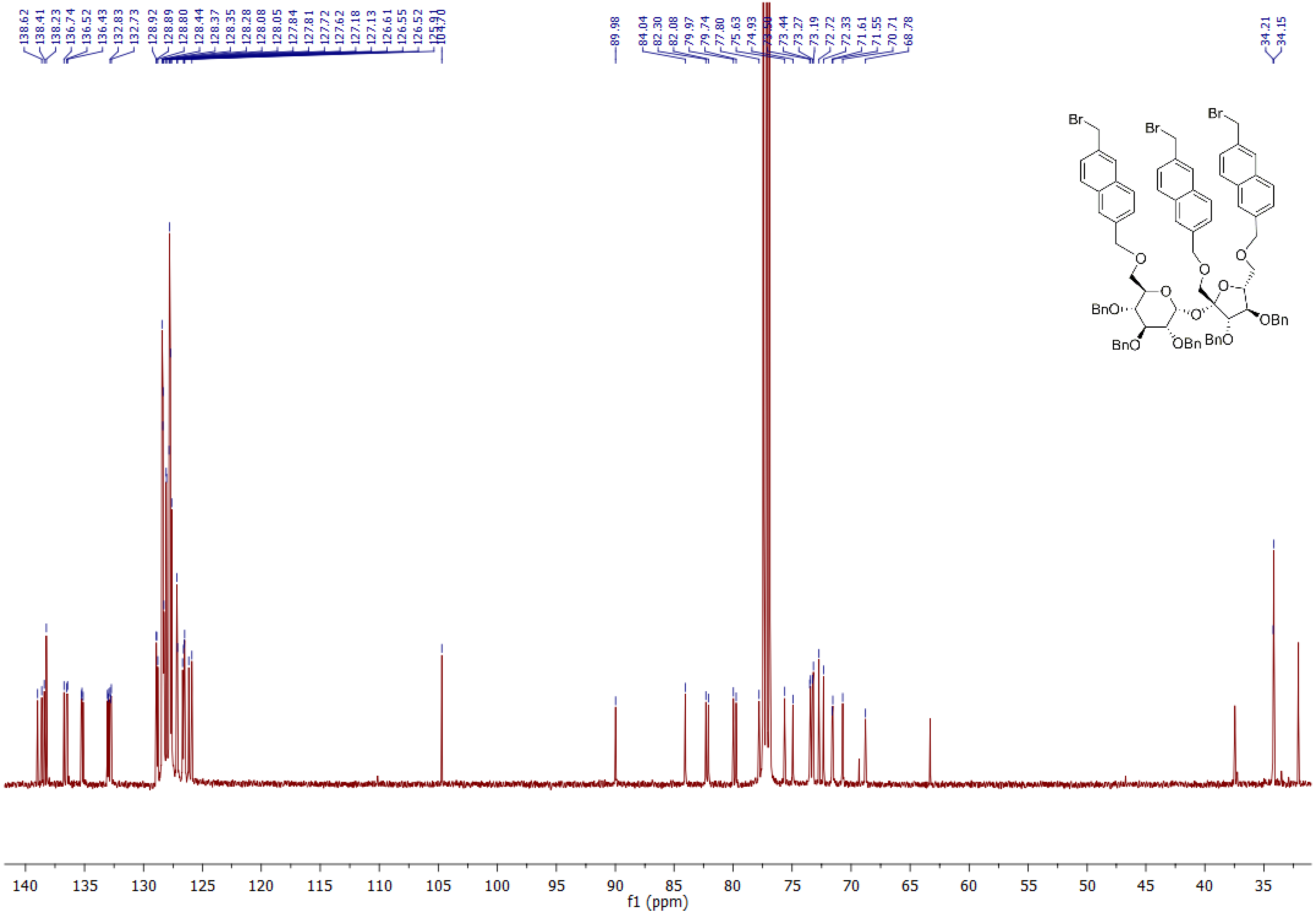

Figure S25. $^{13}C\{^{1}H\}$ NMR (150 MHz, $CDCl_3$) spectrum of compound **3**.

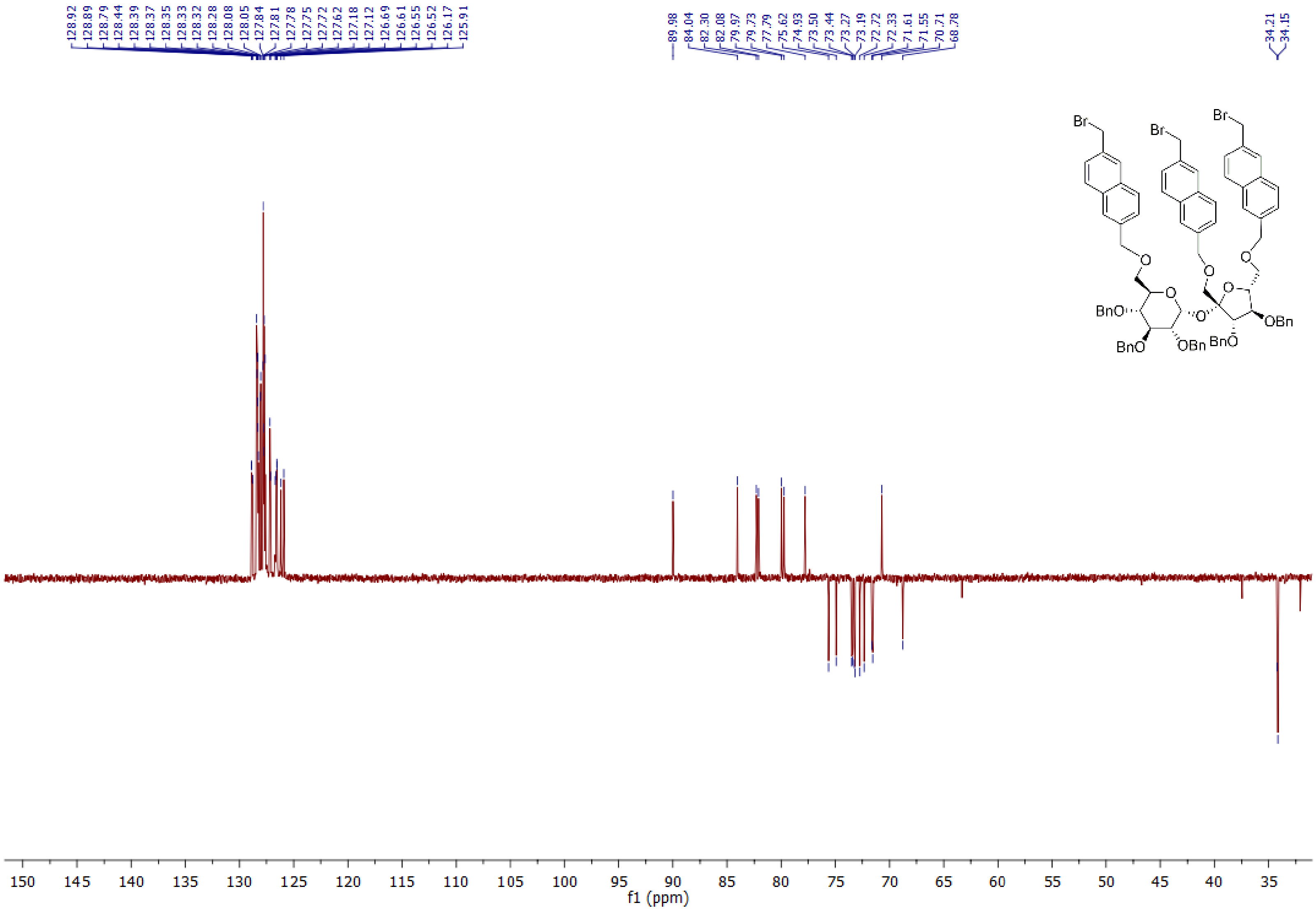

Figure S26. $^{13}C\{^{1}H\}$ DEPT (150 MHz, $CDCl_3$) spectrum of compound **3**.

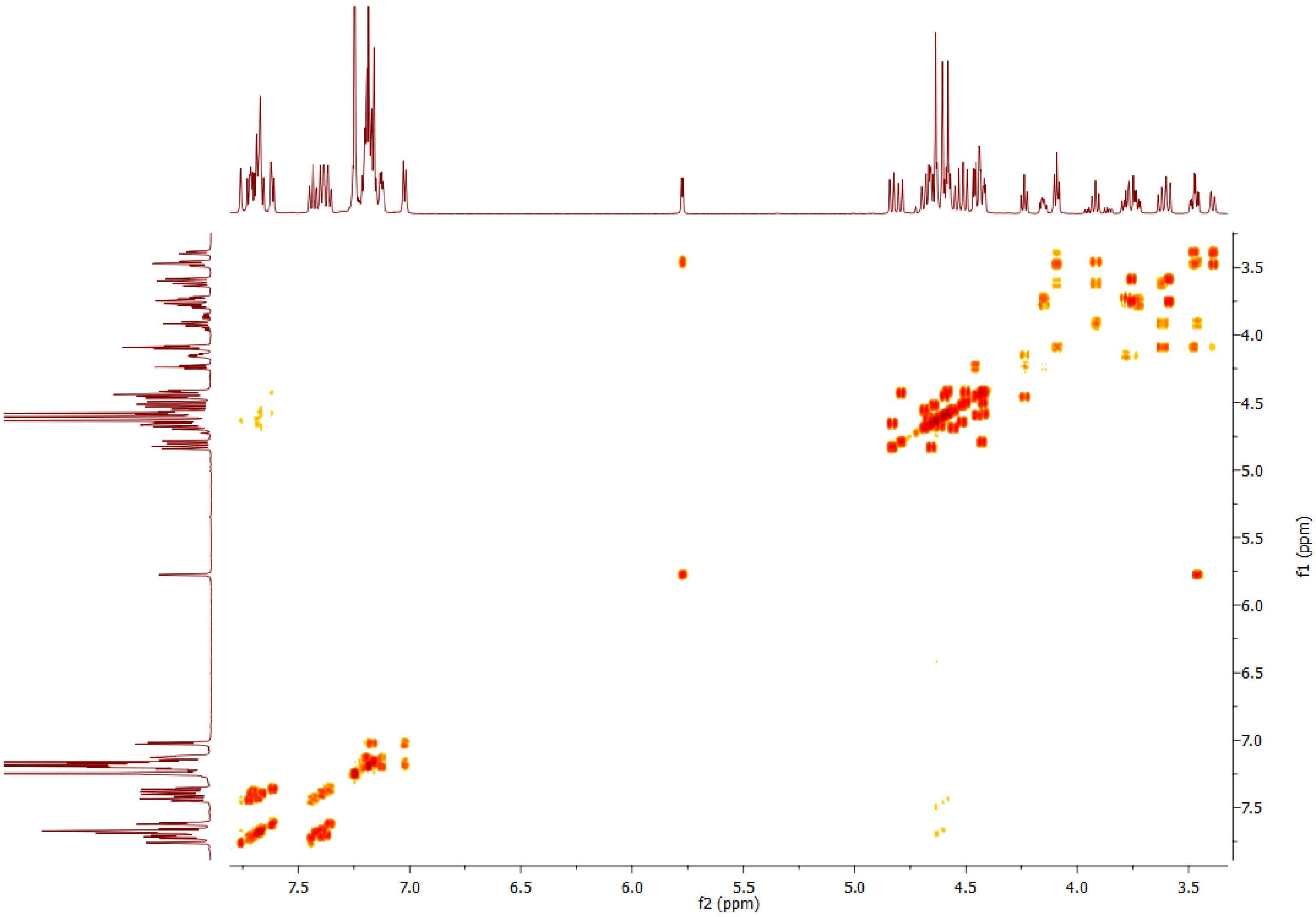

Figure S27. ^{1}H–^{1}H COSY (600 MHz, $CDCl_3$) spectrum of compound **3**.

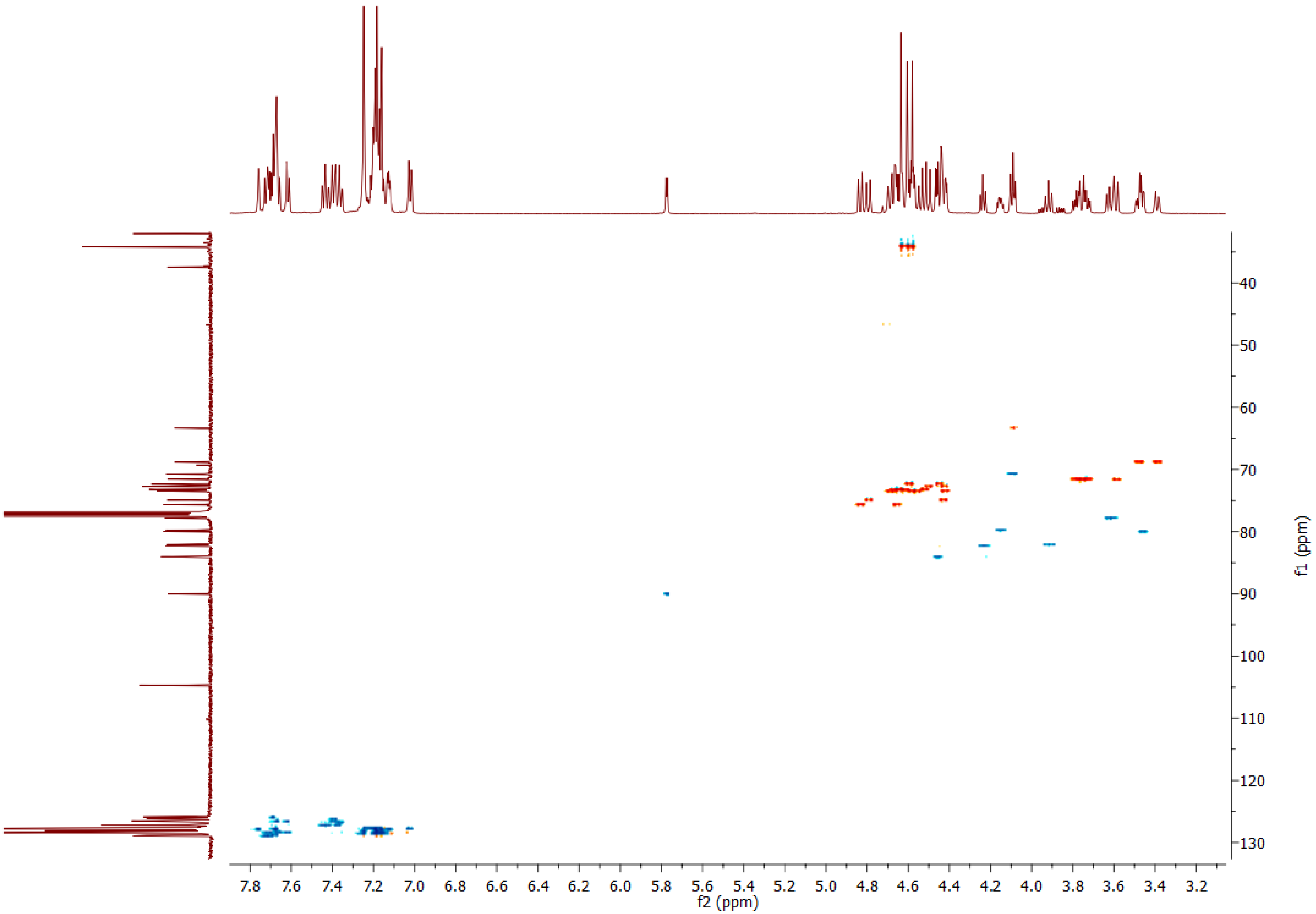

Figure S28. ^{1}H–^{13}C HSQC (600/150 MHz, $CDCl_3$) spectrum of compound **3**.

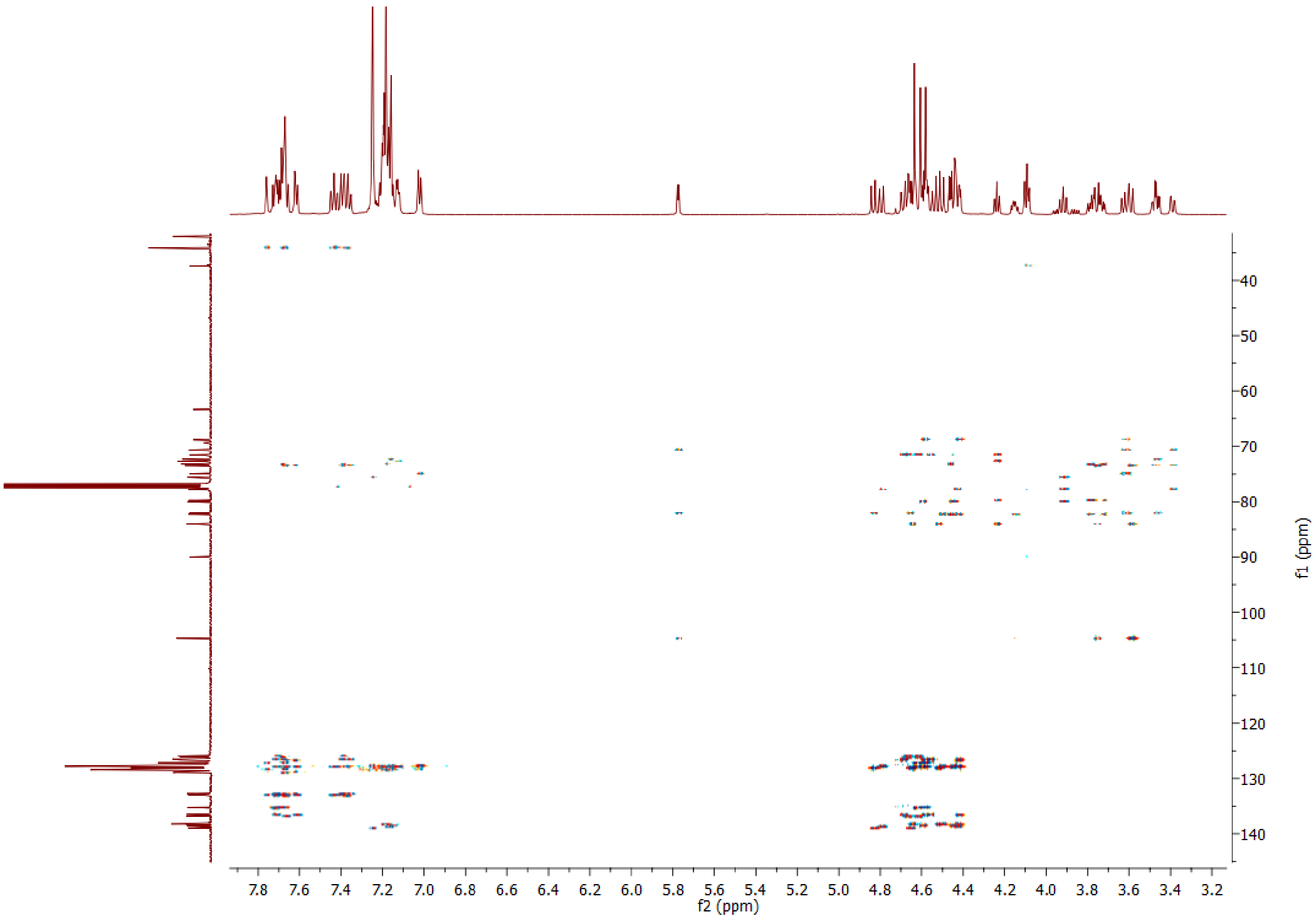

Figure S29. ^{1}H–^{13}C HMBC (600/150 MHz, $CDCl_3$) spectrum of compound **3**.

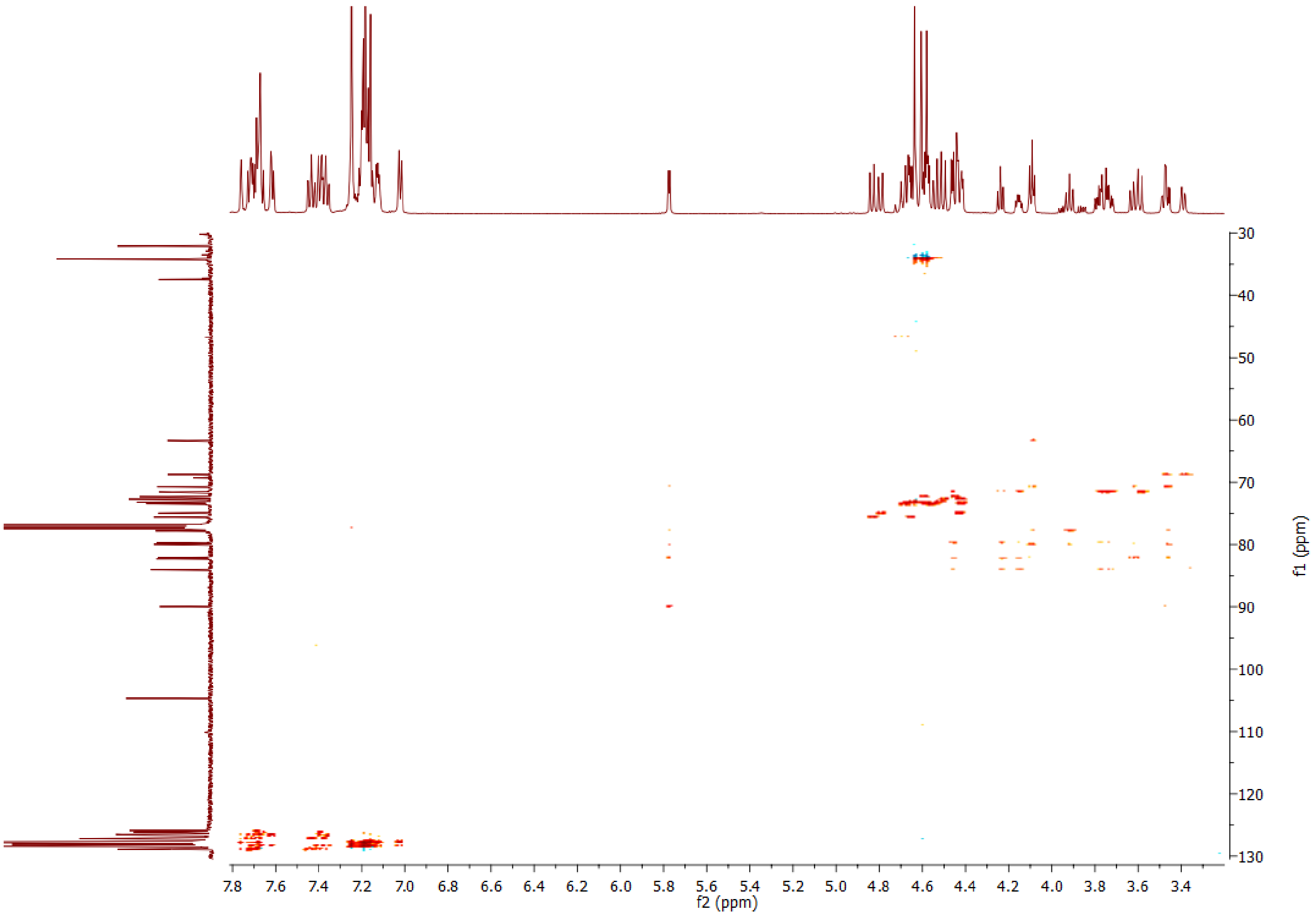

Figure S30. ^1H–^{13}C HSQC-TOCSY (600/150 MHz, $CDCl_3$) spectrum of compound **3**.

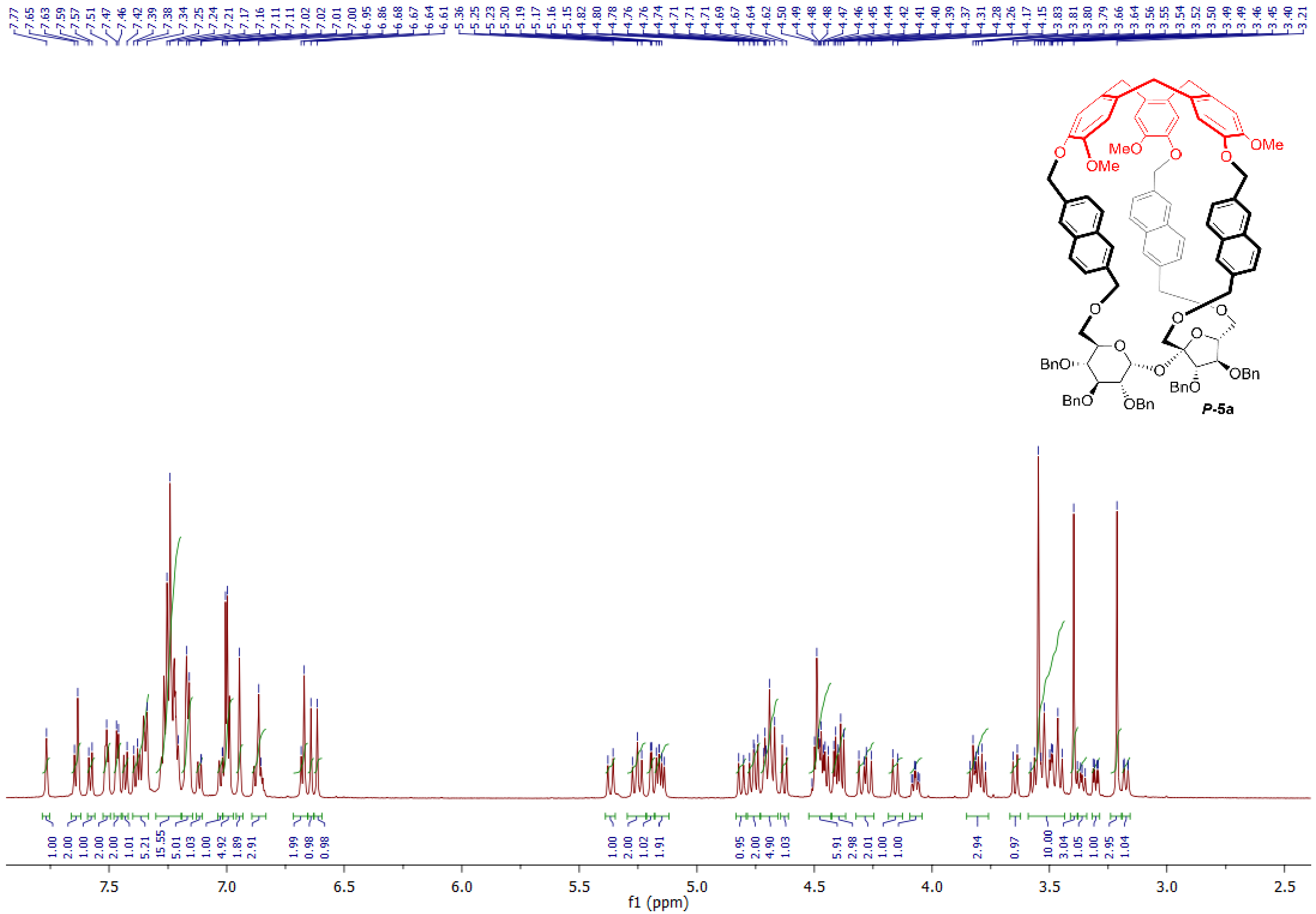

Figure S31. ^{1}H NMR (600 MHz, $CDCl_3$) spectrum of compound ***P*-5a.**

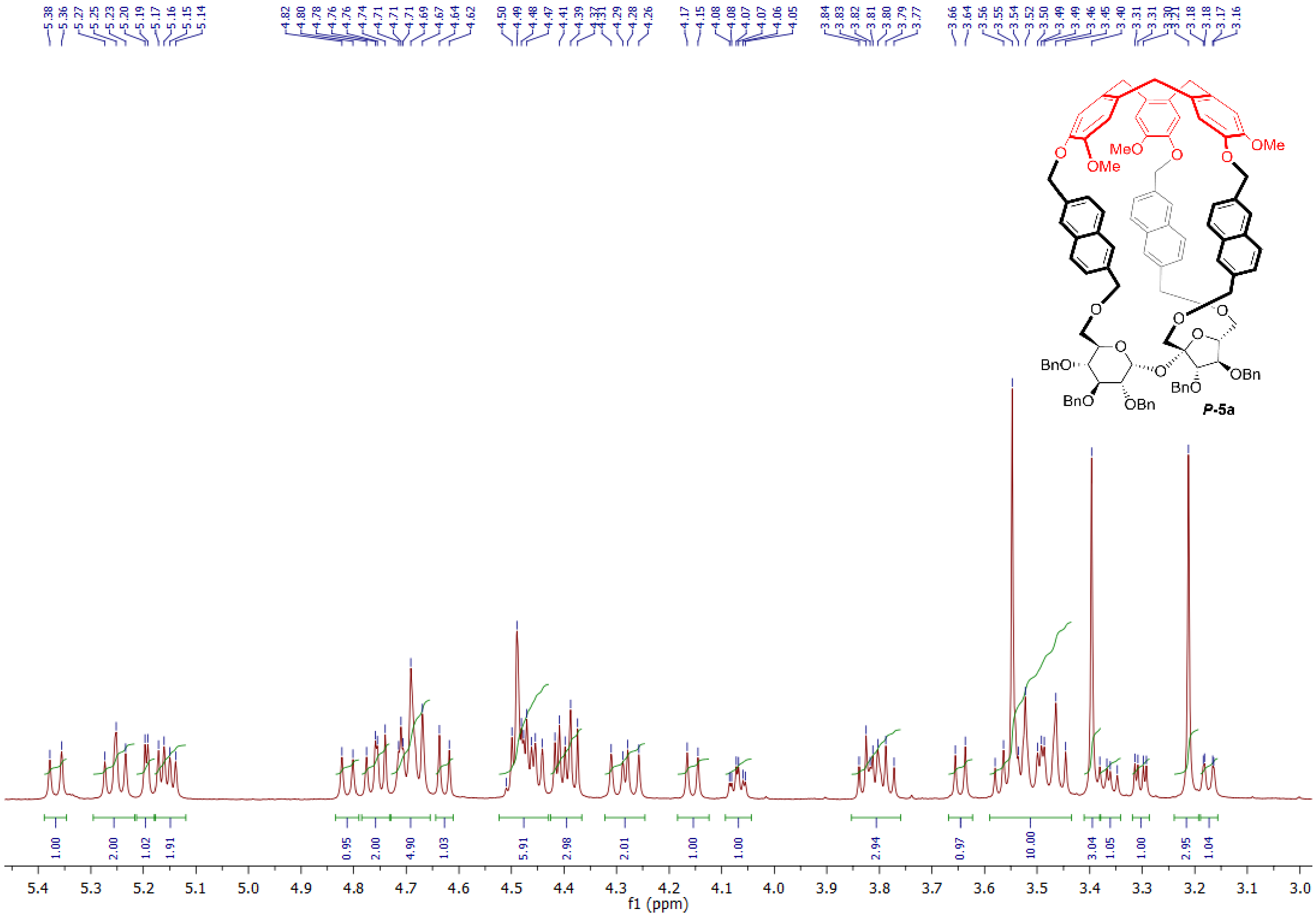

Figure S32. ^{1}H NMR (600 MHz, $CDCl_3$) spectrum of compound ***P*-5a** (aliphatic part).

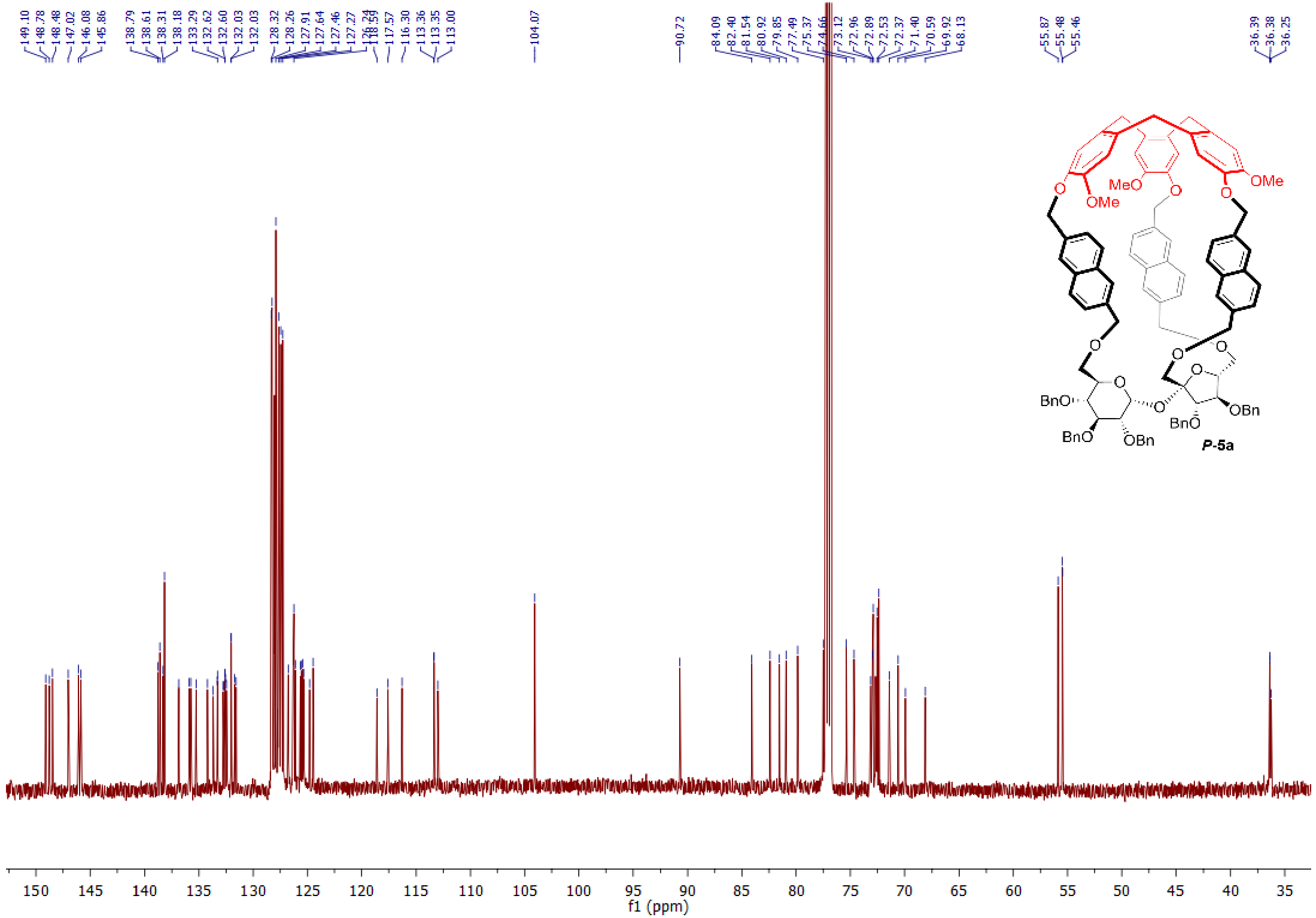

Figure S33. $^{13}C\{^{1}H\}$ NMR (150 MHz, $CDCl_3$) spectrum of compound ***P*-5a.**

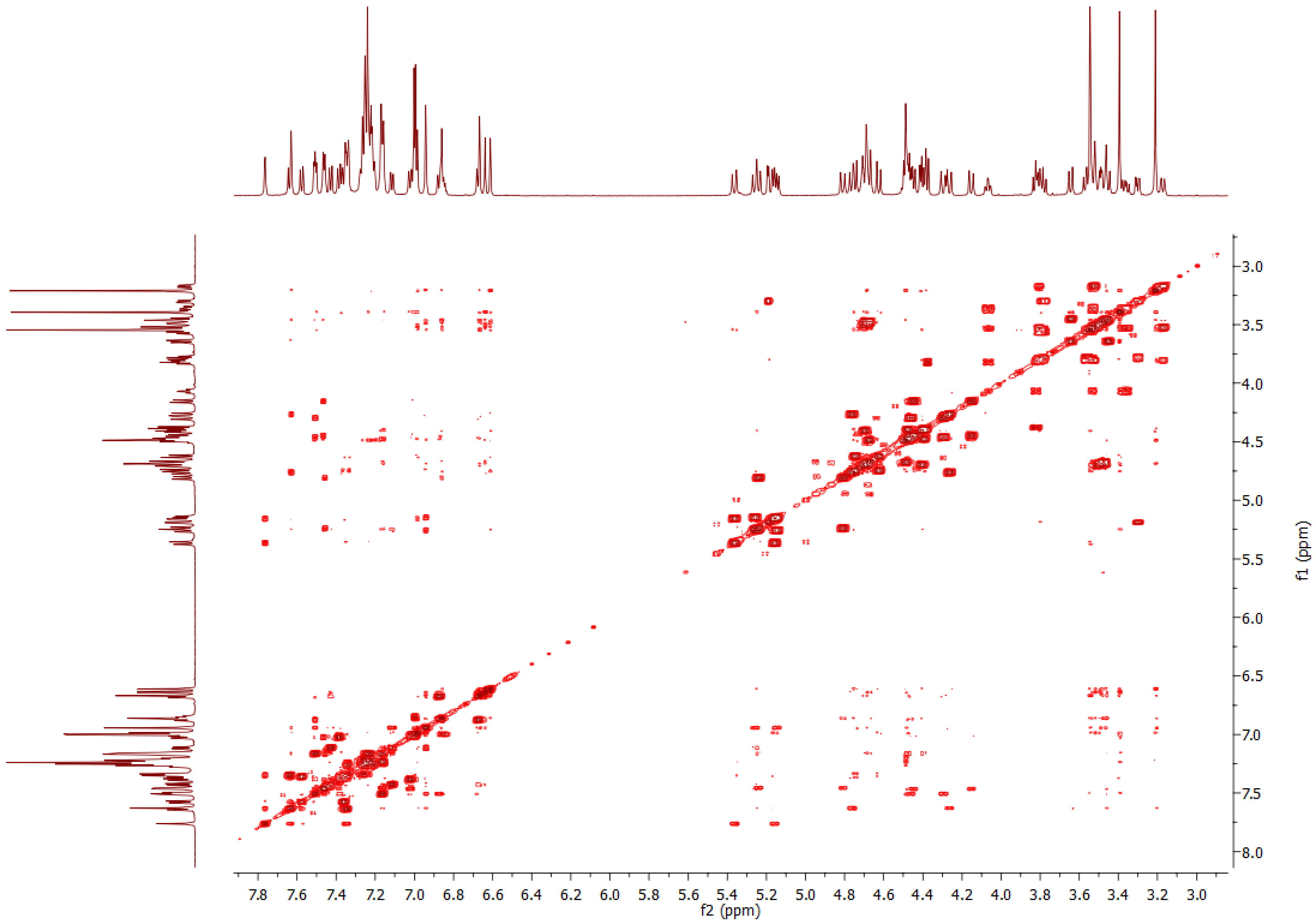

Figure S34. ^{1}H–^{1}H COSY (600 MHz, $CDCl_3$) spectrum of compound ***P*-5a**.

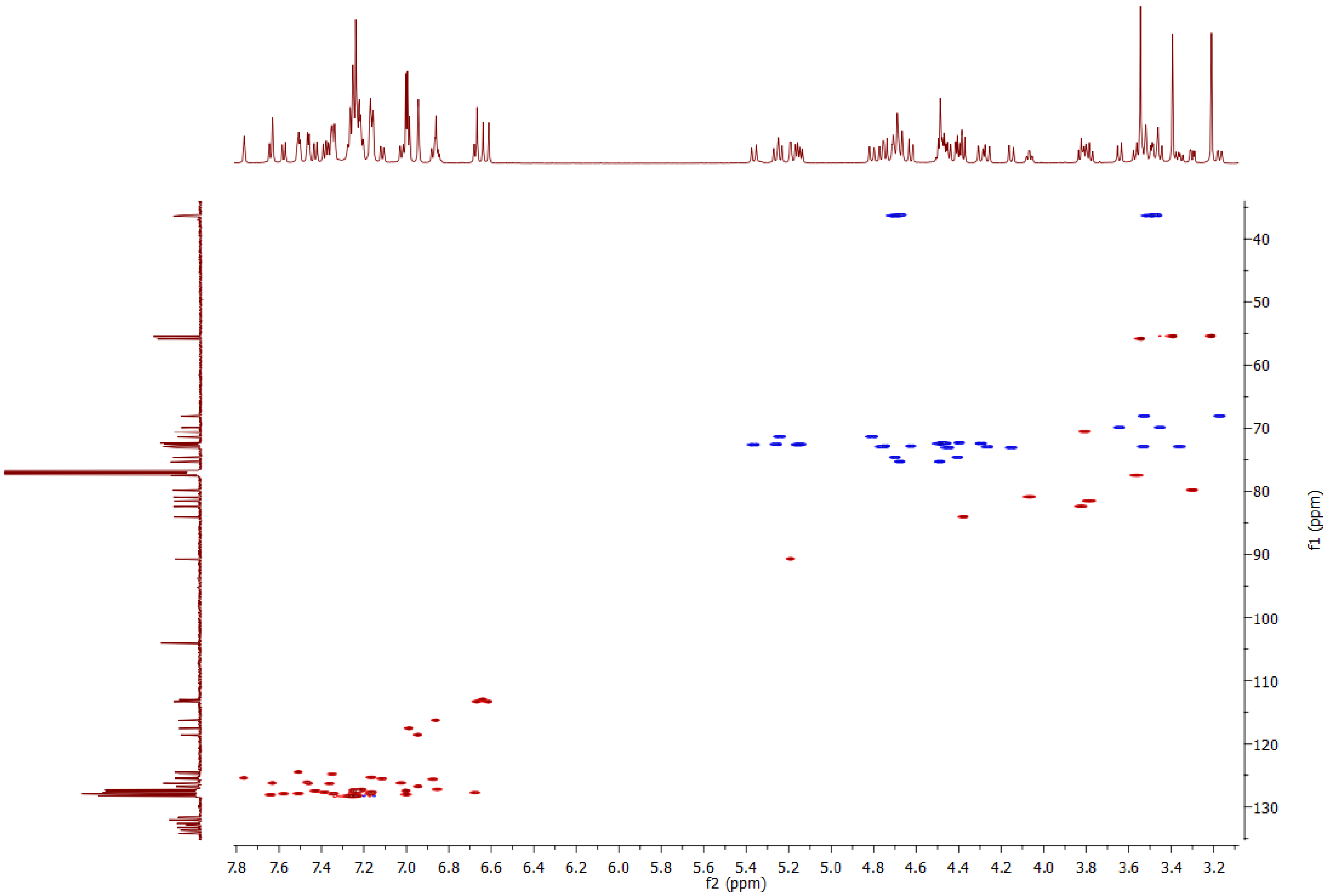

Figure S35. ^{1}H–^{13}C HSQC (600/150 MHz, $CDCl_3$) spectrum of compound ***P*-5a**.

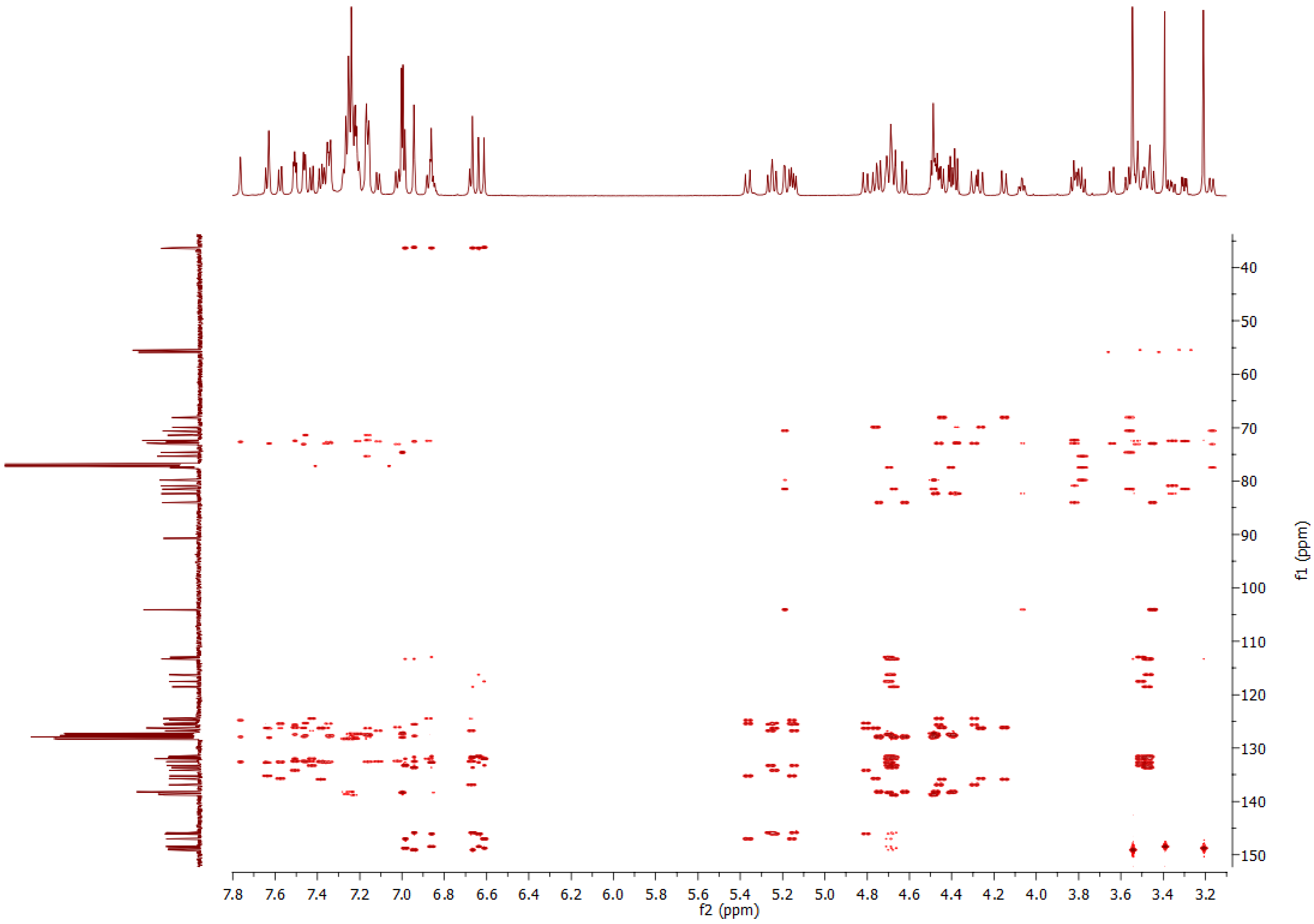

Figure S36. $^{1}H–^{13}C$ HMBC (600/150 MHz, $CDCl_3$) spectrum of compound ***P*-5a.**

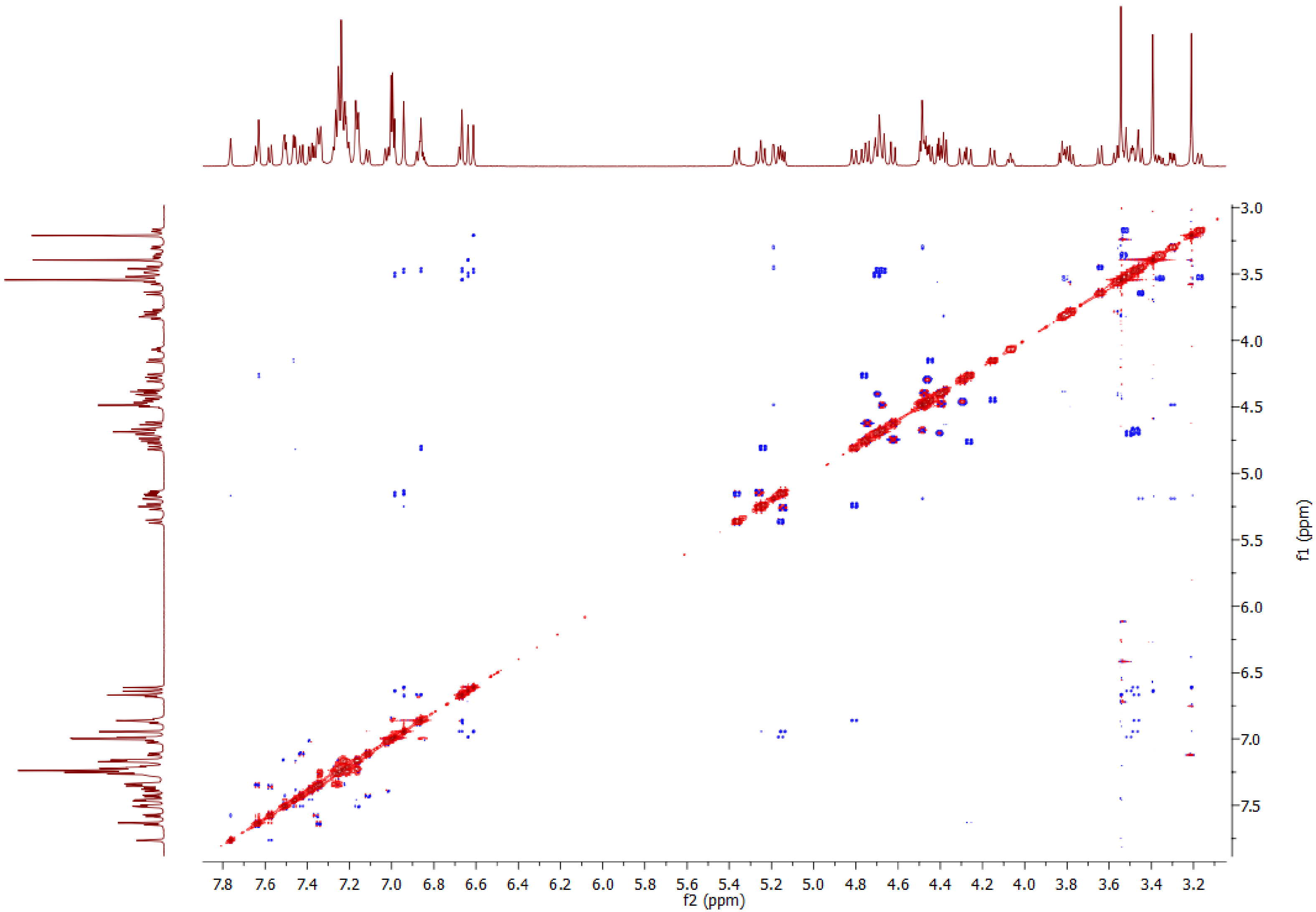

Figure S37. ^{1}H–^{1}H ROESY (600 MHz, $CDCl_3$) spectrum of compound ***P*-5a.**

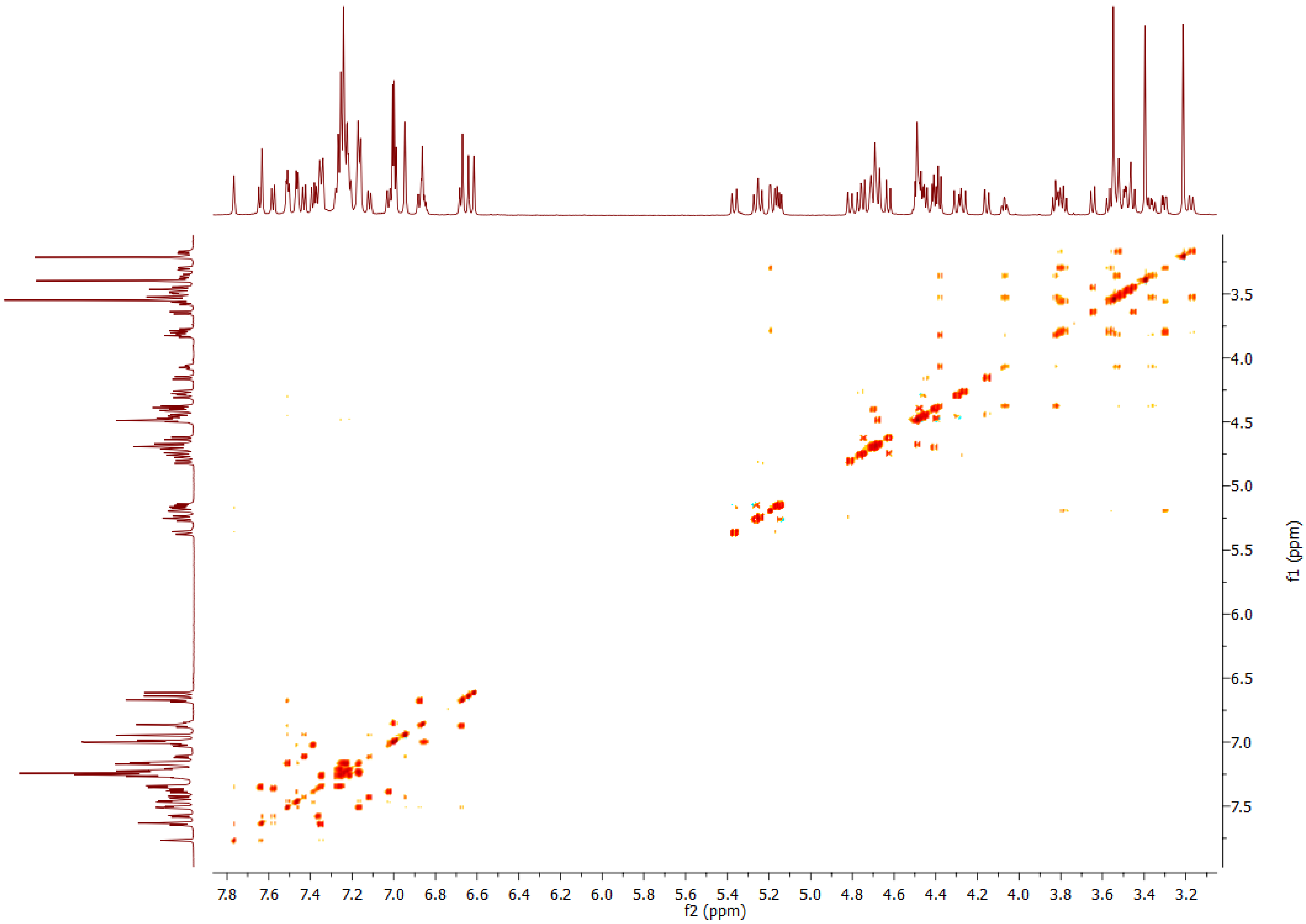

Figure S38. ^{1}H–^{1}H TOCSY (600 MHz, $CDCl_3$) spectrum of compound ***P*-5a.**

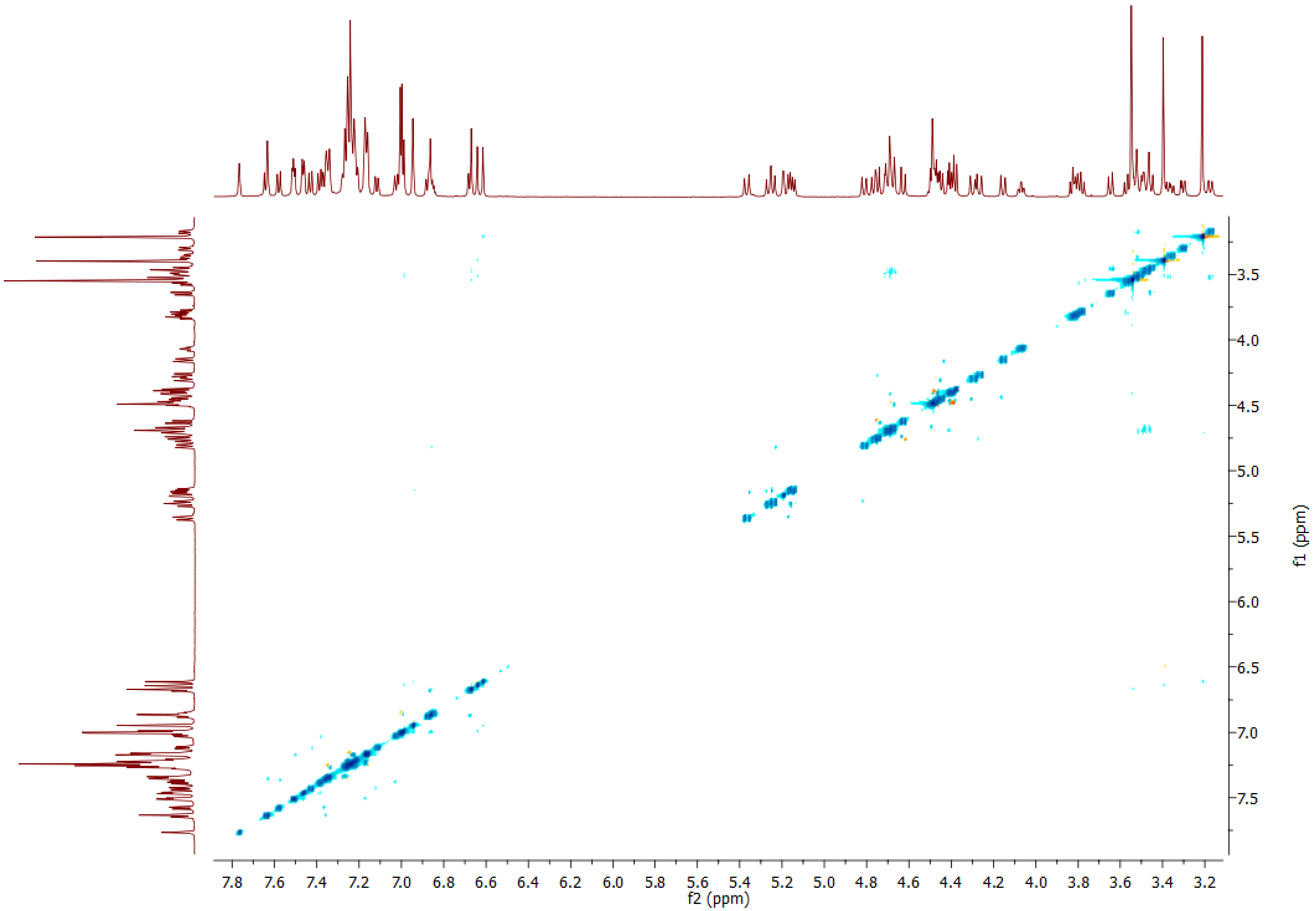

Figure S39. ^{1}H–^{1}H NOESY (600 MHz, $CDCl_3$) spectrum of compound ***P*-5a.**

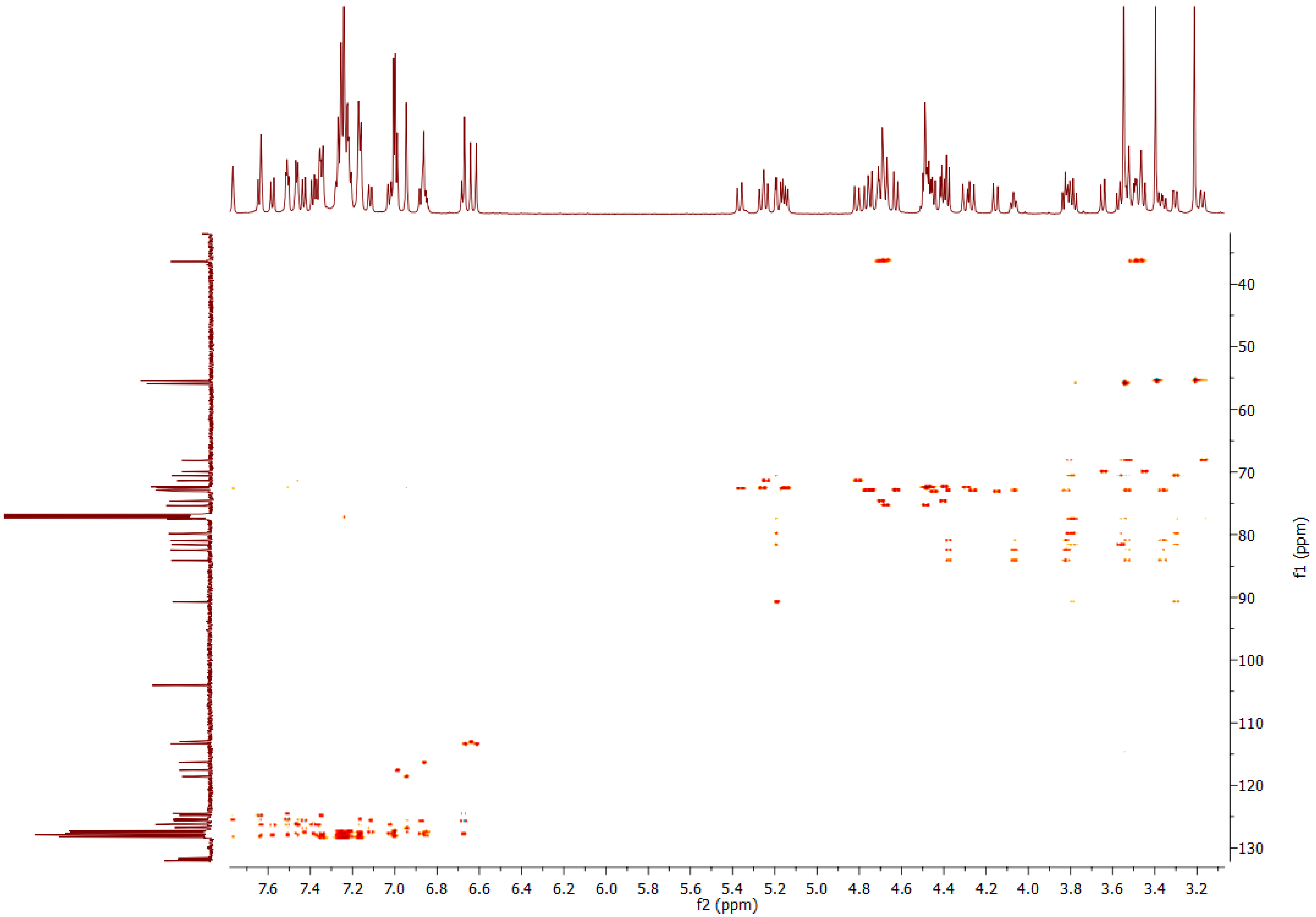

Figure S40. ^1^H–^13^C HSQC-TOCSY (600/150 MHz, $CDCl_3$) spectrum of compound ***P*-5a.**

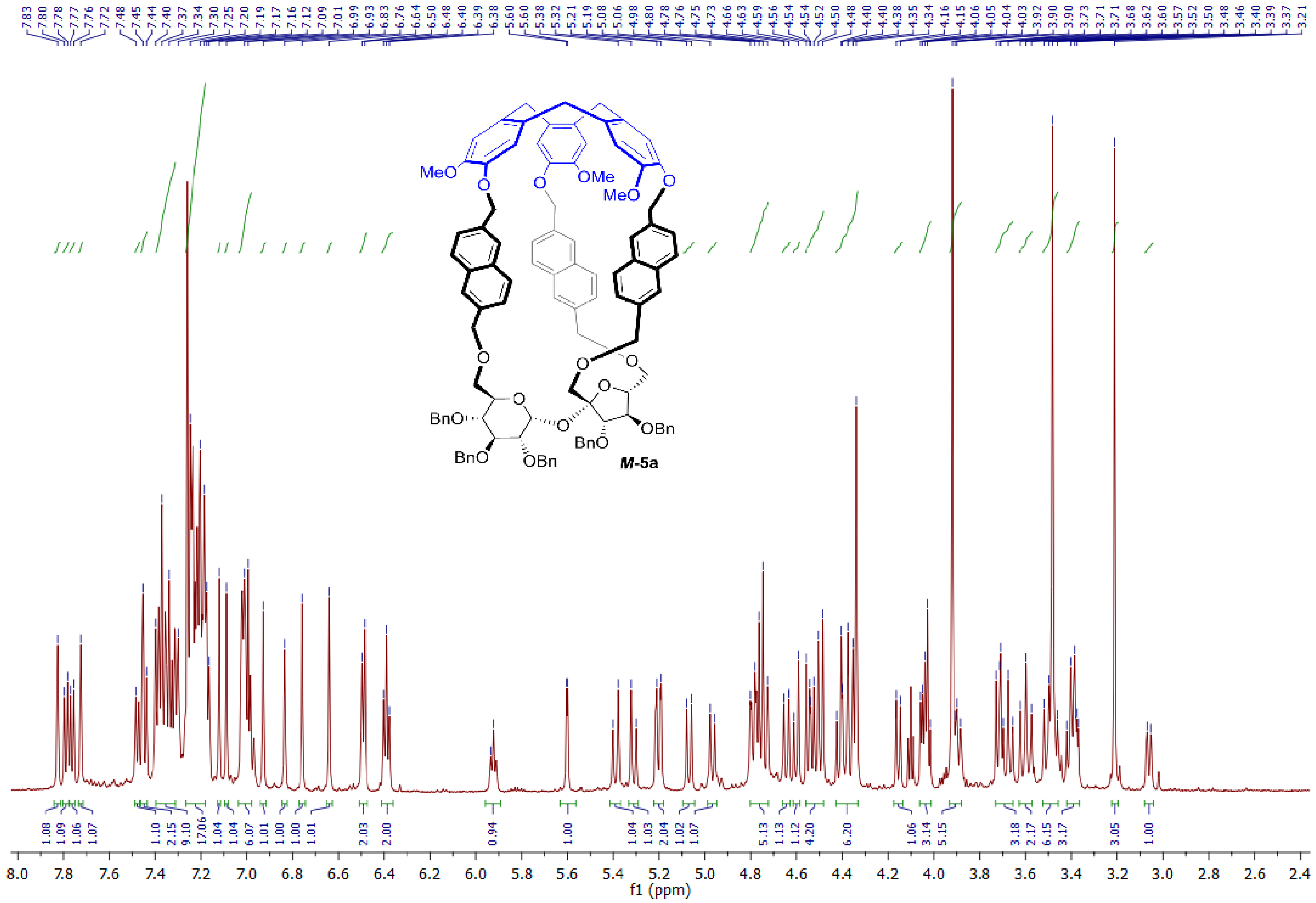

Figure S41. ^{1}H NMR (600 MHz, $CDCl_3$) spectrum of compound ***M*-5a.**

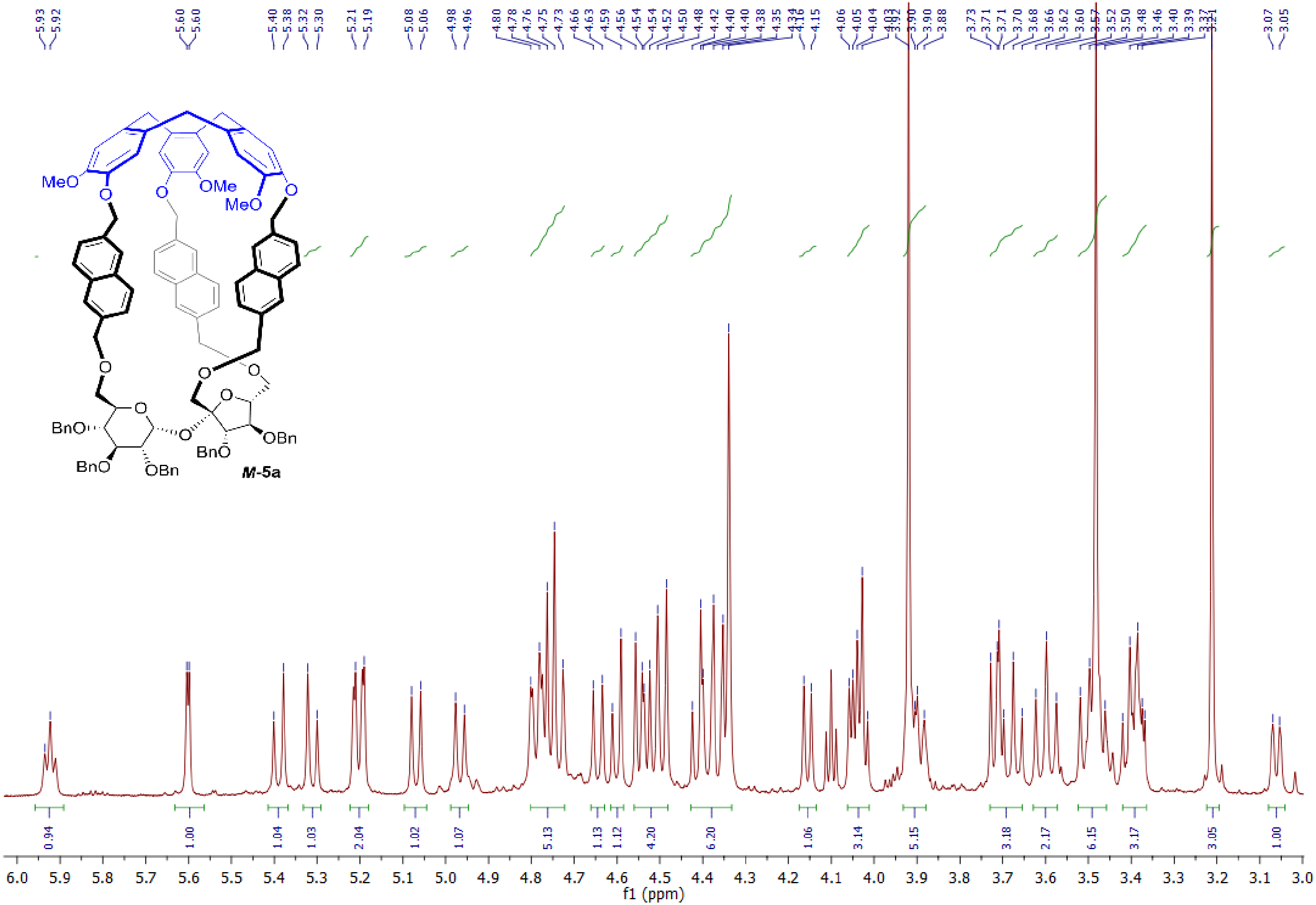

Figure S42. ^{1}H NMR (600 MHz, $CDCl_3$) spectrum of compound ***M*-5a** (aliphatic part).

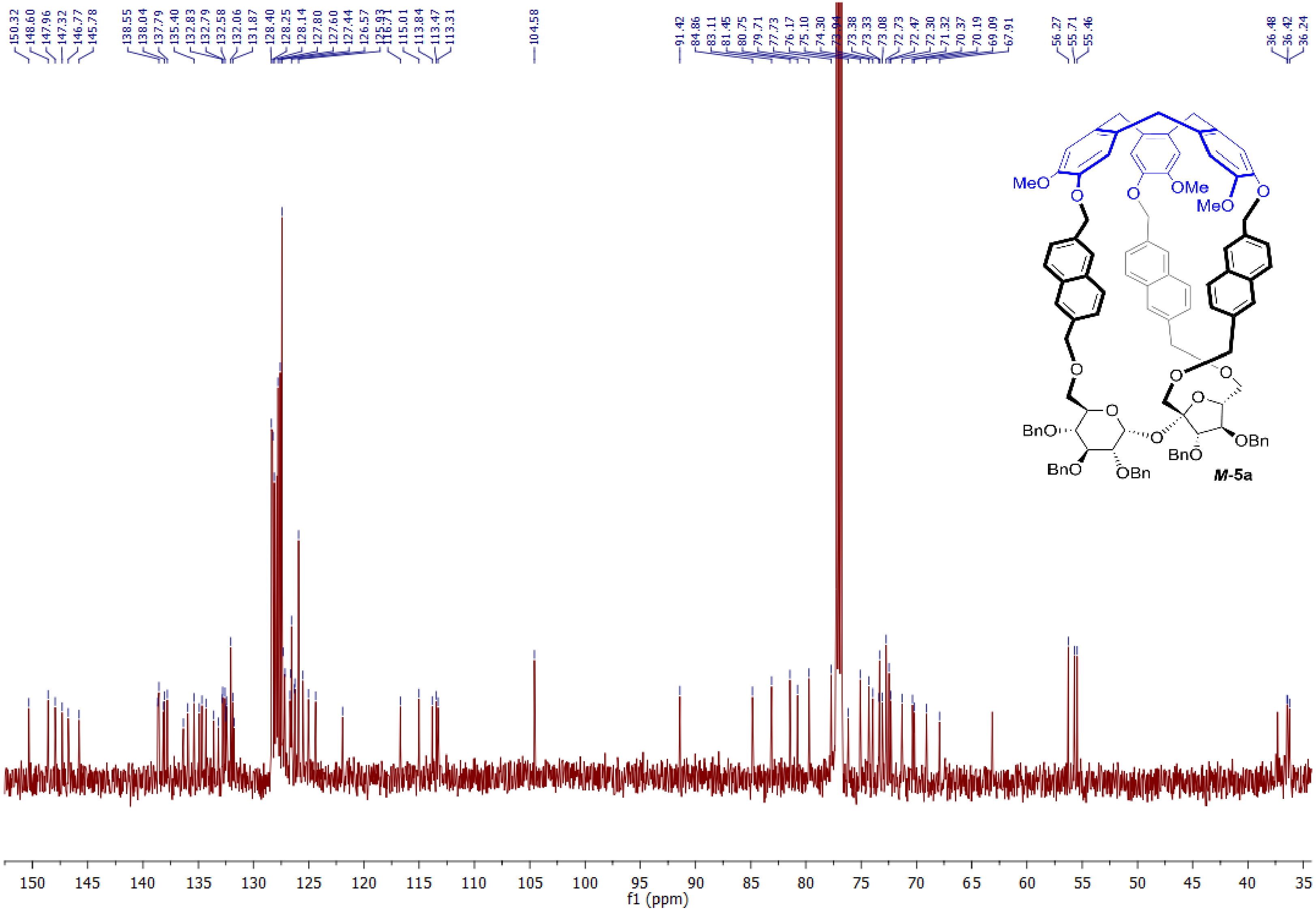

Figure S43. $^{13}C\{^{1}H\}$ NMR (150 MHz, $CDCl_3$) spectrum of compound ***M*-5a.**

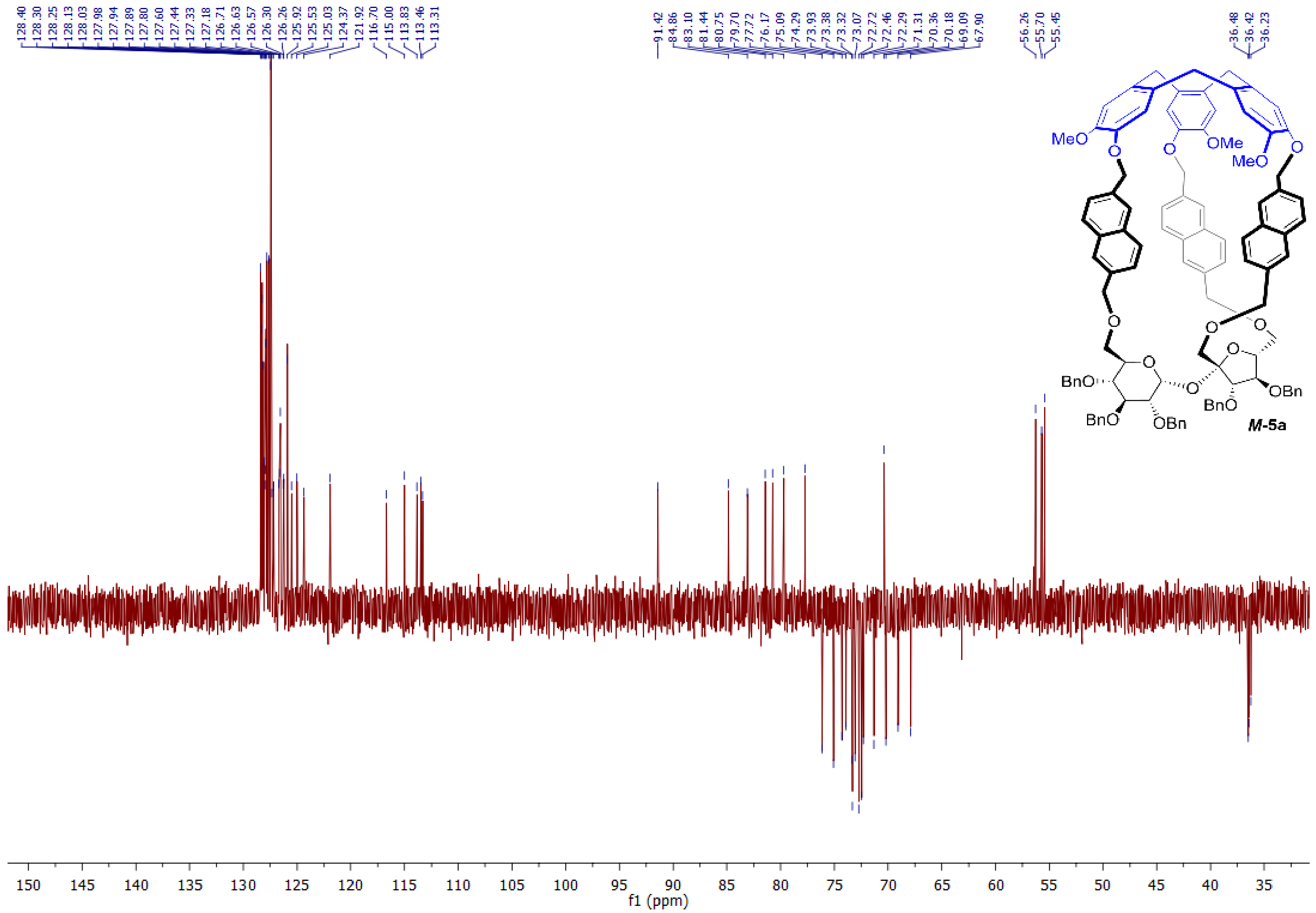

Figure S44. $^{13}C\{^1H\}$ DEPT (150 MHz, $CDCl_3$) spectrum of compound ***M*-5a.**

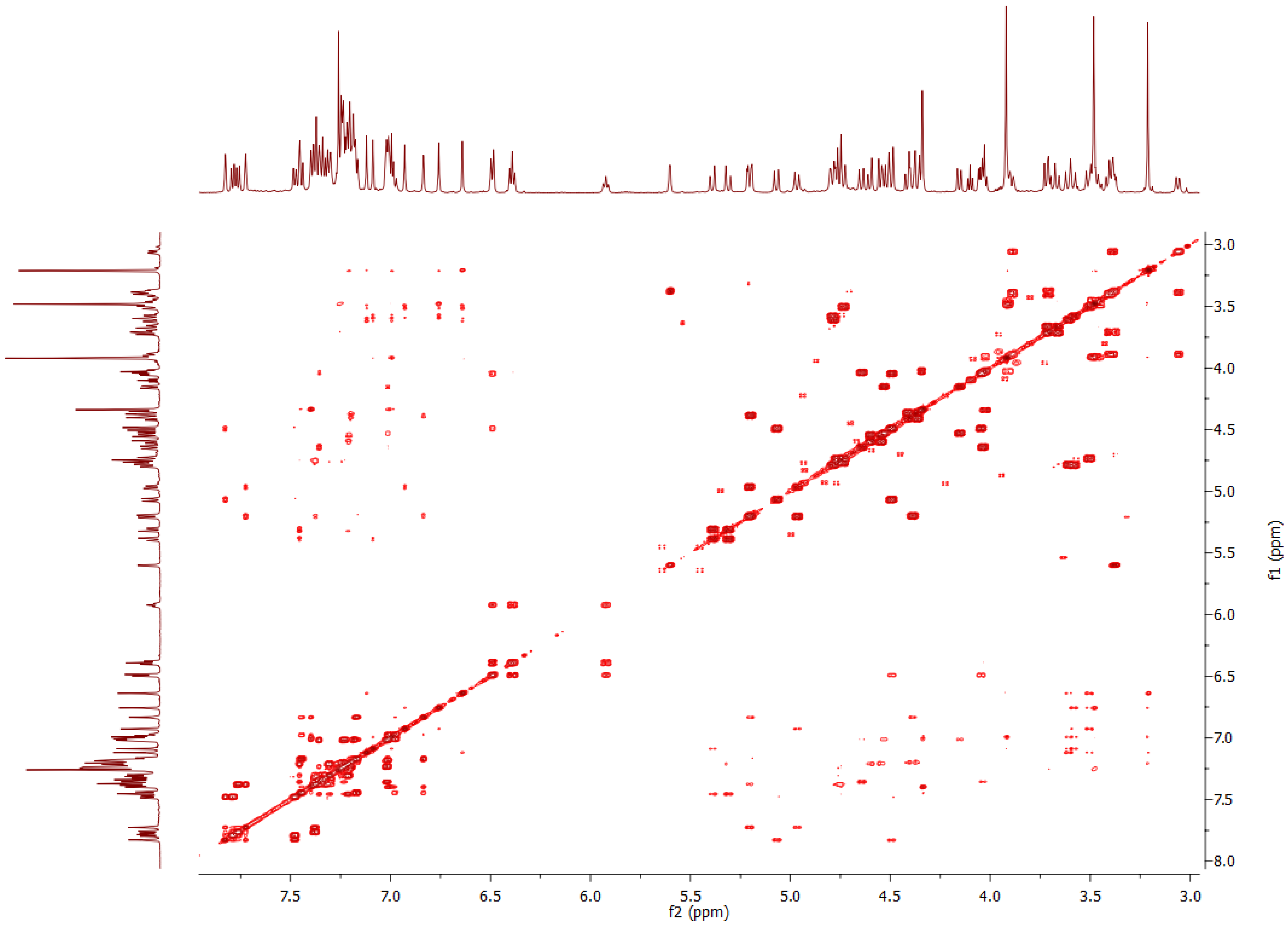

Figure S45. ^1H–^1H COSY (600 MHz, $CDCl_3$) spectrum of compound ***M*-5a.**

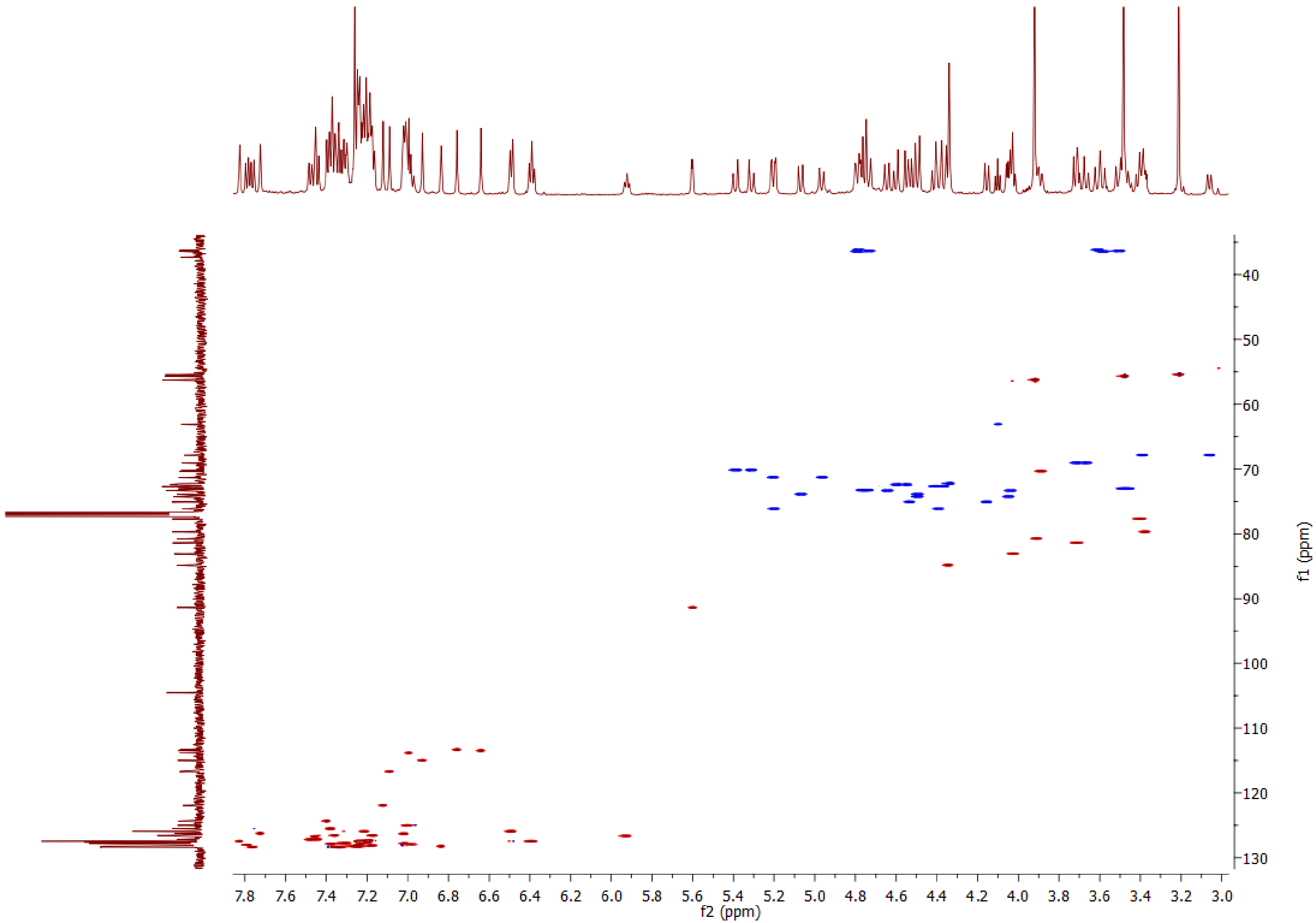

Figure S46. ^{1}H–^{13}C HSQC (600/150 MHz, $CDCl_3$) spectrum of compound ***M*-5a**.

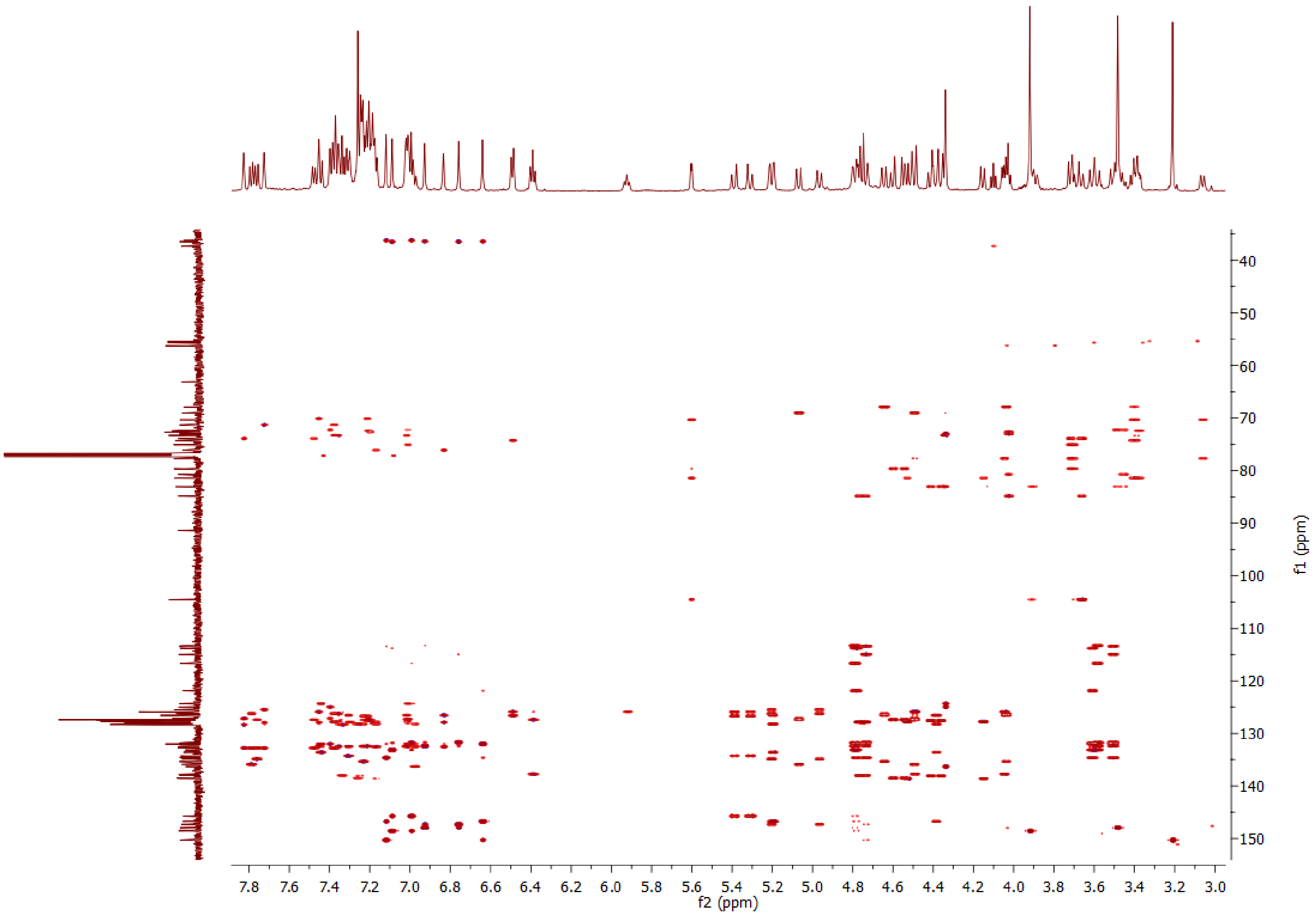

Figure S47. ^1H–^{13}C HMBC (600/150 MHz, $CDCl_3$) spectrum of compound ***M*-5a.**

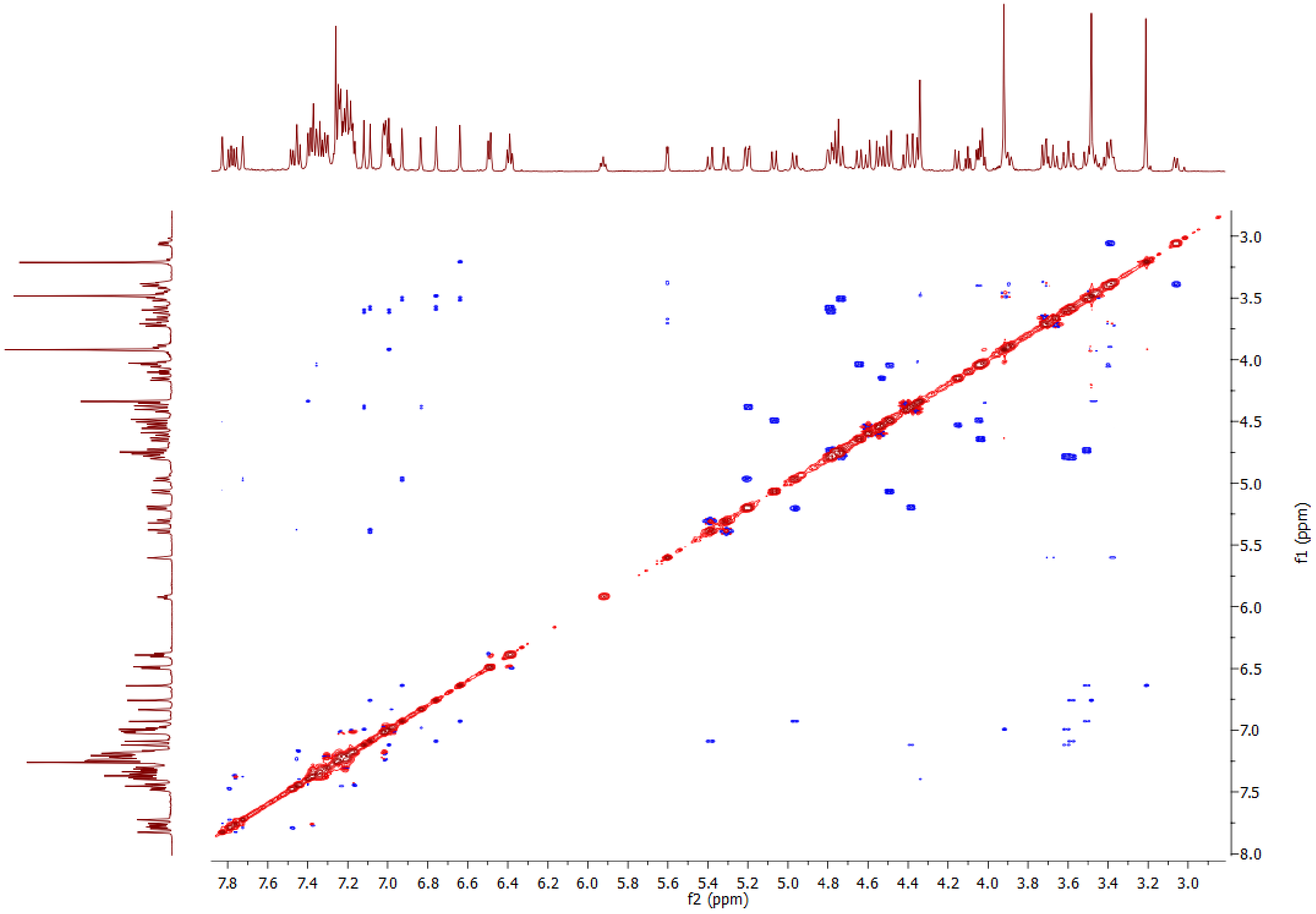

Figure S48. ^1H–^1H ROESY (600 MHz, $CDCl_3$) spectrum of compound ***M*-5a.**

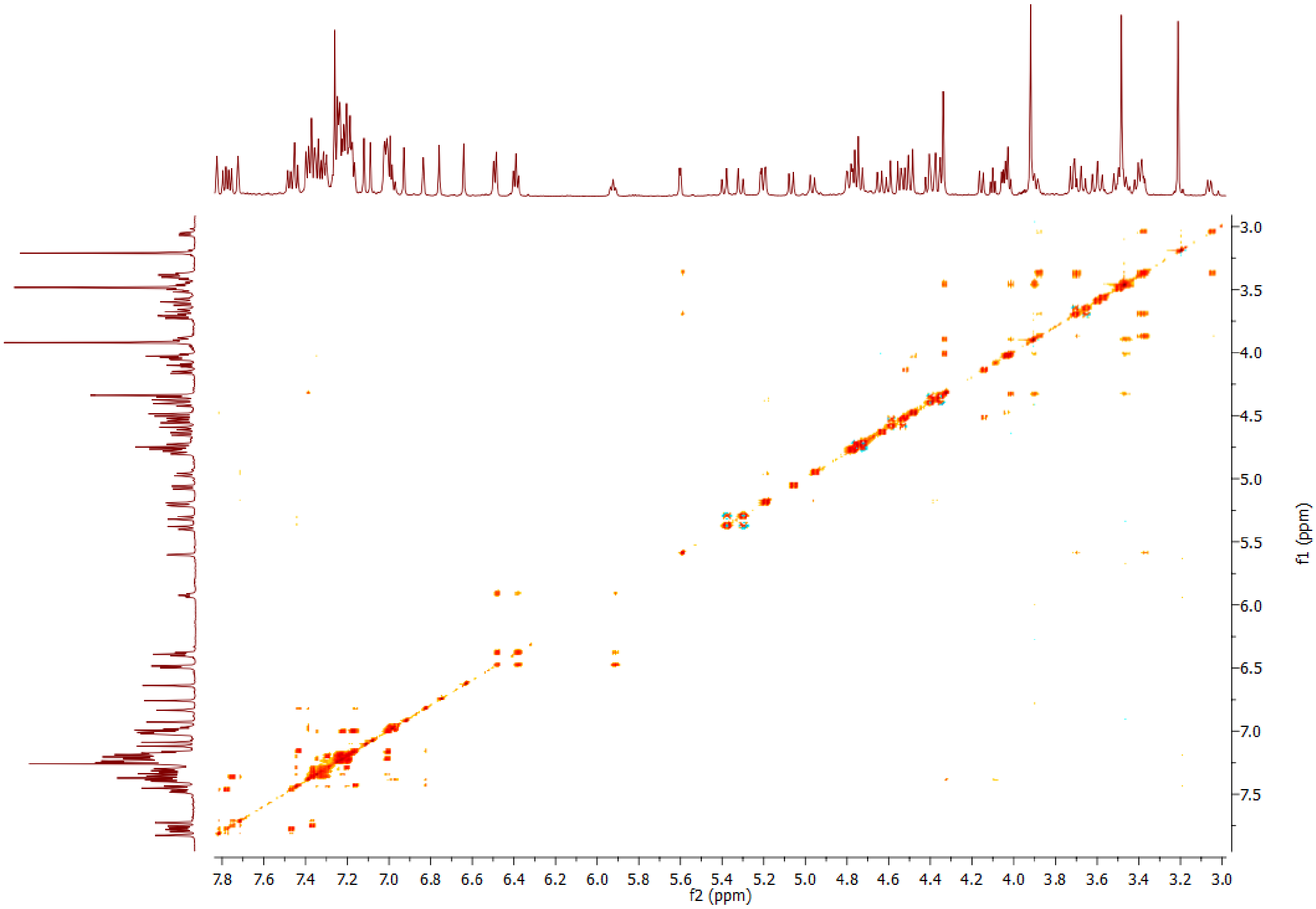

Figure S49. ^{1}H–^{1}H TOCSY (600 MHz, $CDCl_3$) spectrum of compound ***M*-5a.**

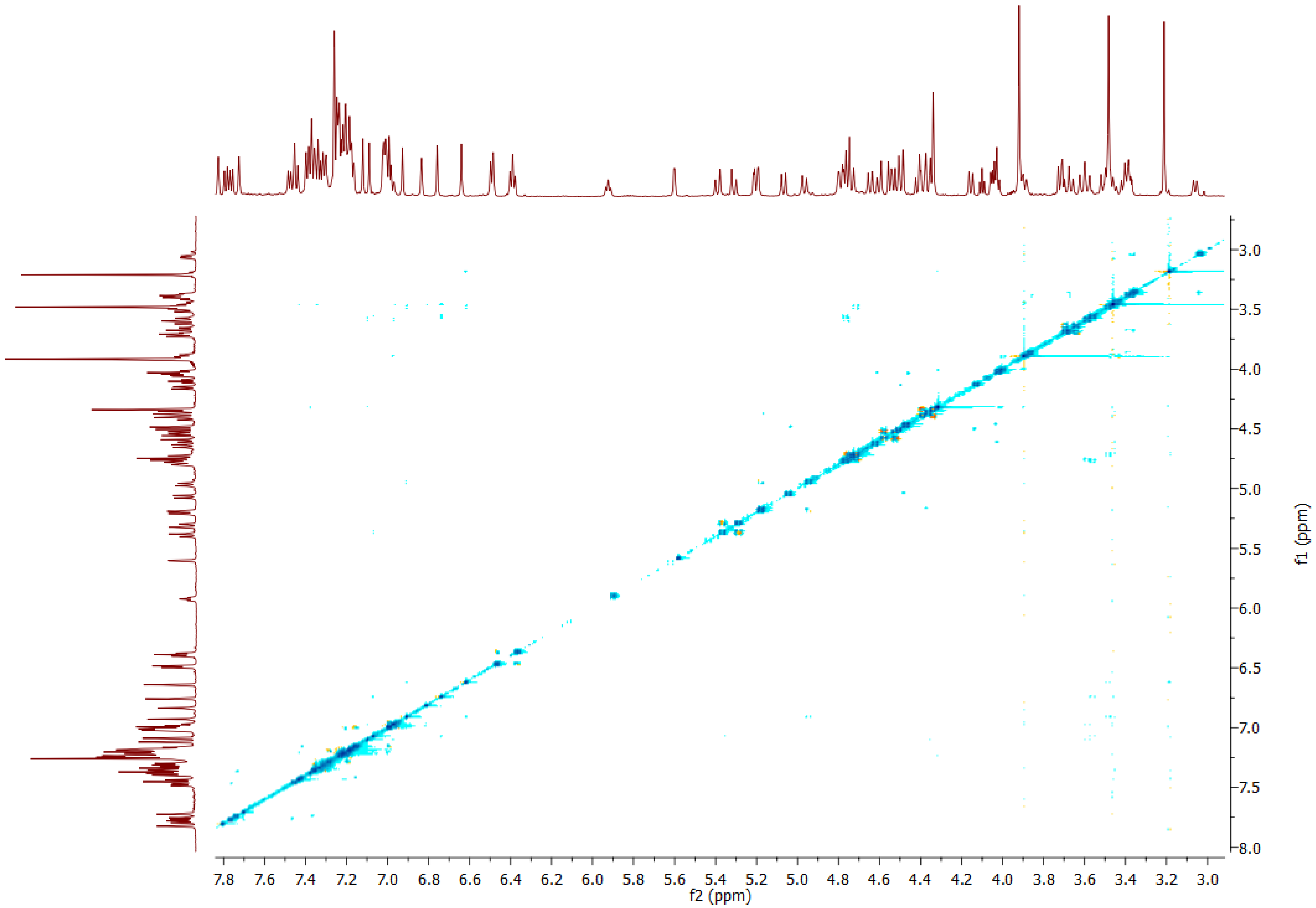

Figure S50. ${}^{1}H$–${}^{1}H$ NOESY (600 MHz, $CDCl_3$) spectrum of compound ***M*-5a.**

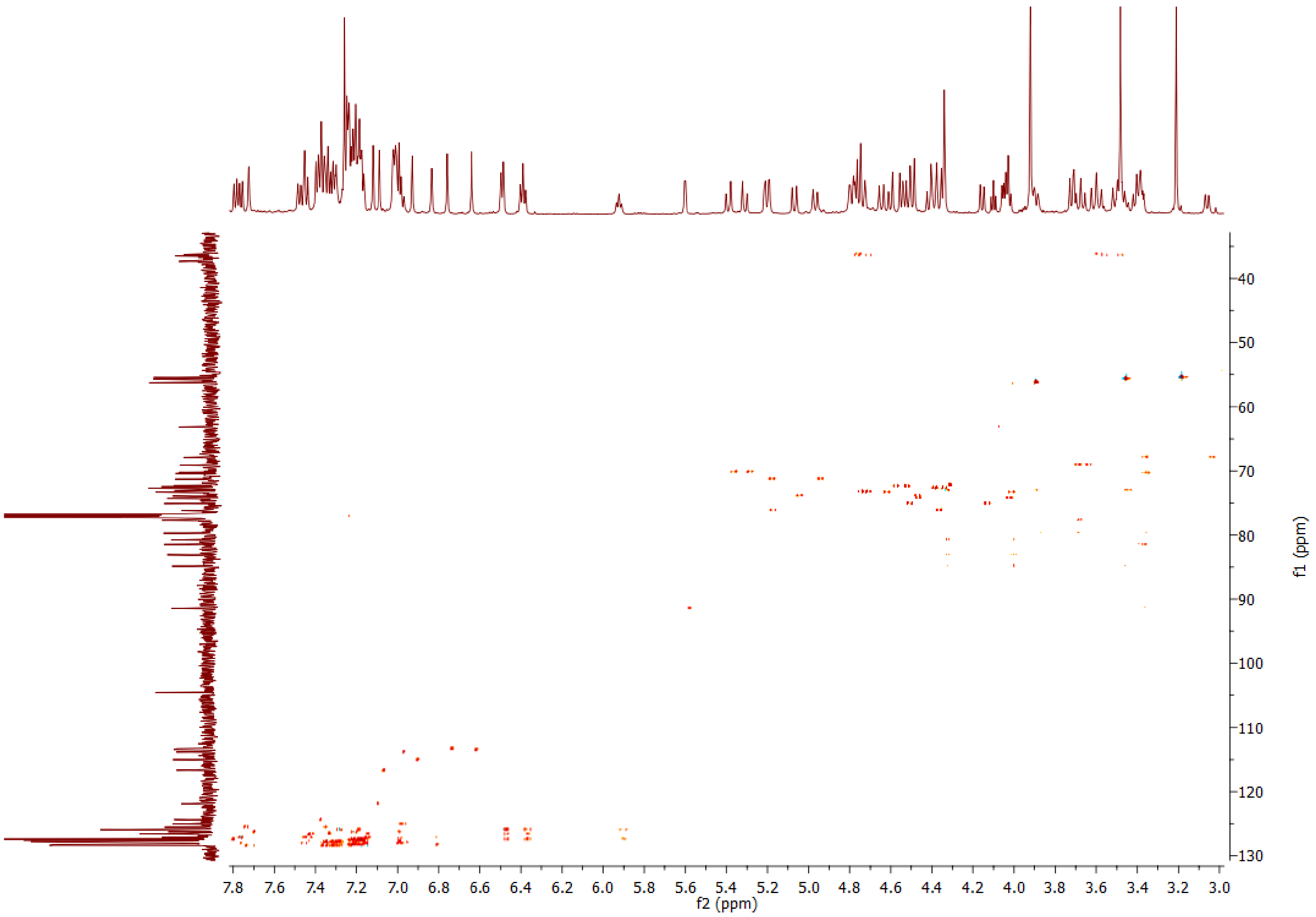

Figure S51. ^{1}H–^{13}C HSQC-TOCSY (600/150 MHz, $CDCl_3$) spectrum of compound ***M*-5a.**

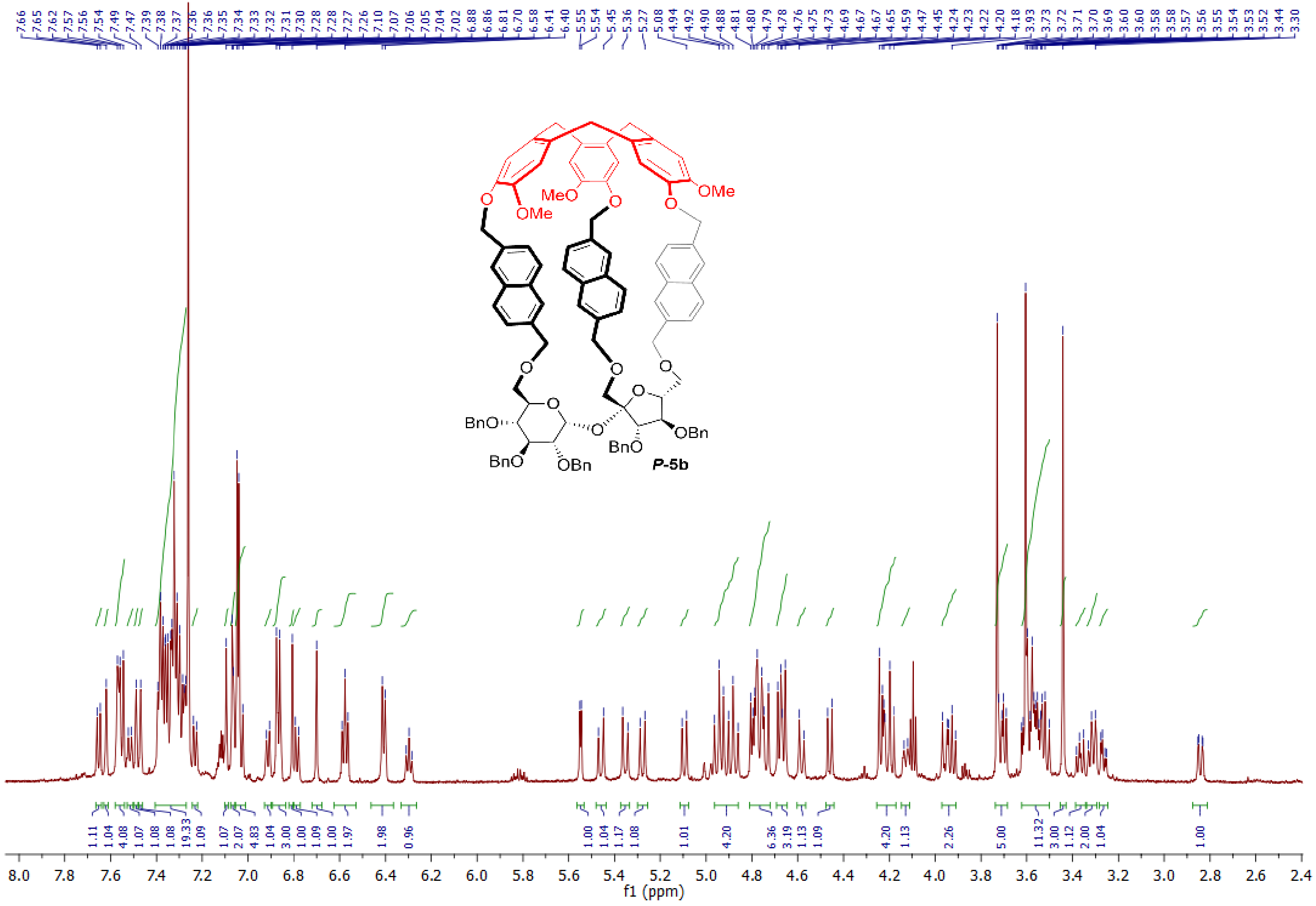

Figure S52. ^{1}H NMR (600 MHz, $CDCl_3$) spectrum of compound ***P*-5b.**

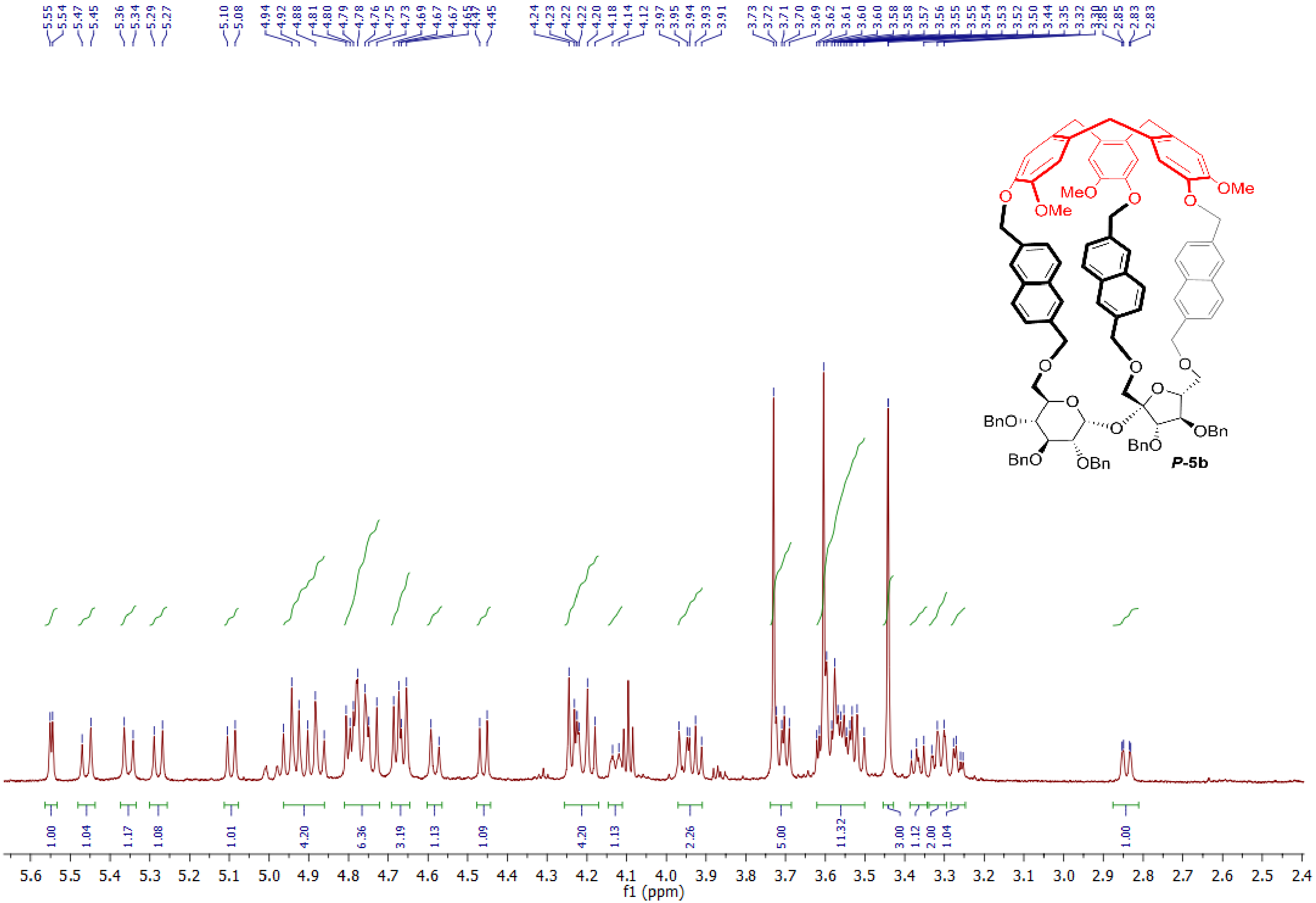

Figure S53. ^{1}H NMR (600 MHz, $CDCl_3$) spectrum of compound ***P*-5b** (aliphatic part).

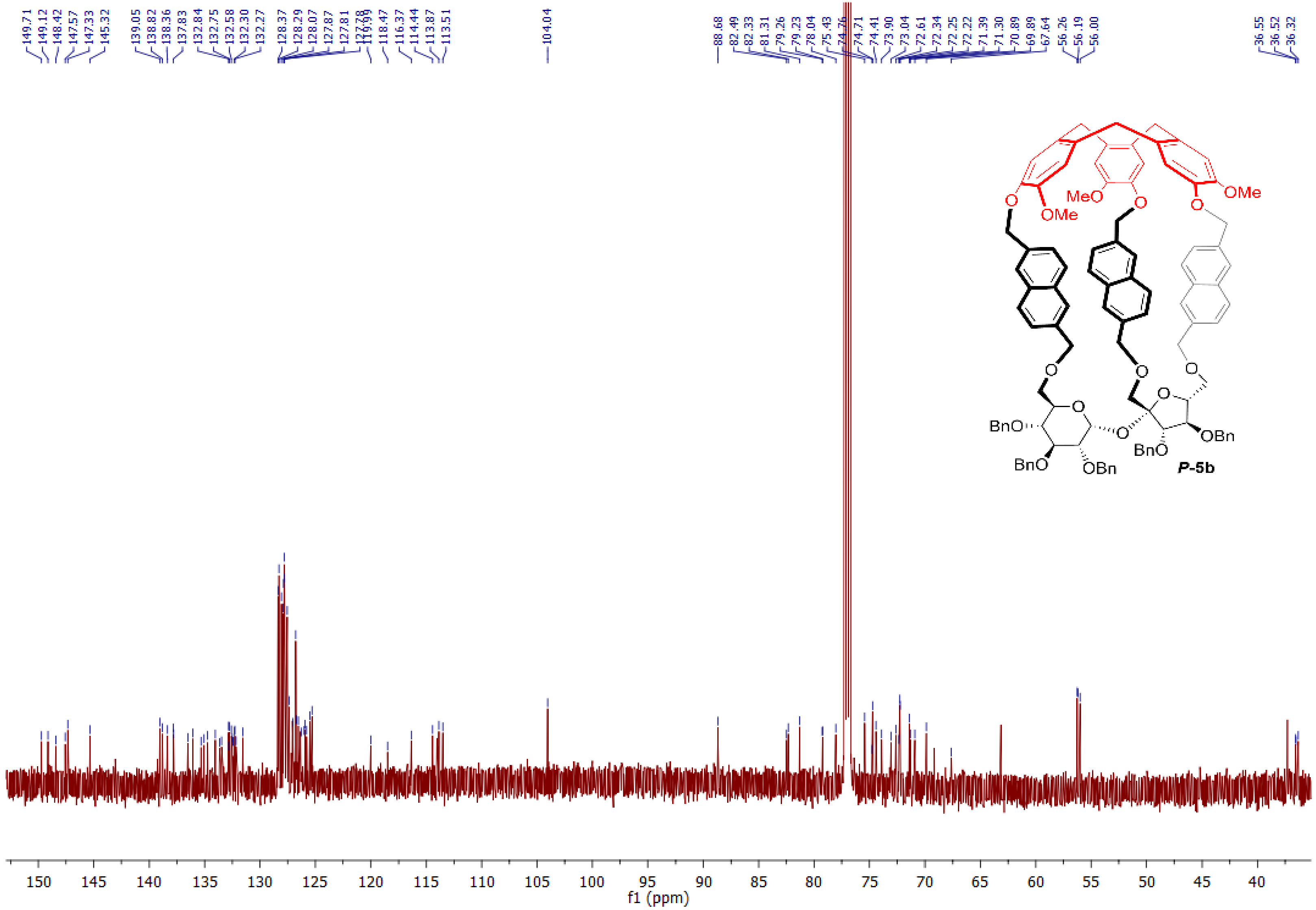

Figure S54. $^{13}C\{^{1}H\}$ NMR (150 MHz, $CDCl_3$) spectrum of compound ***P*-5b.**

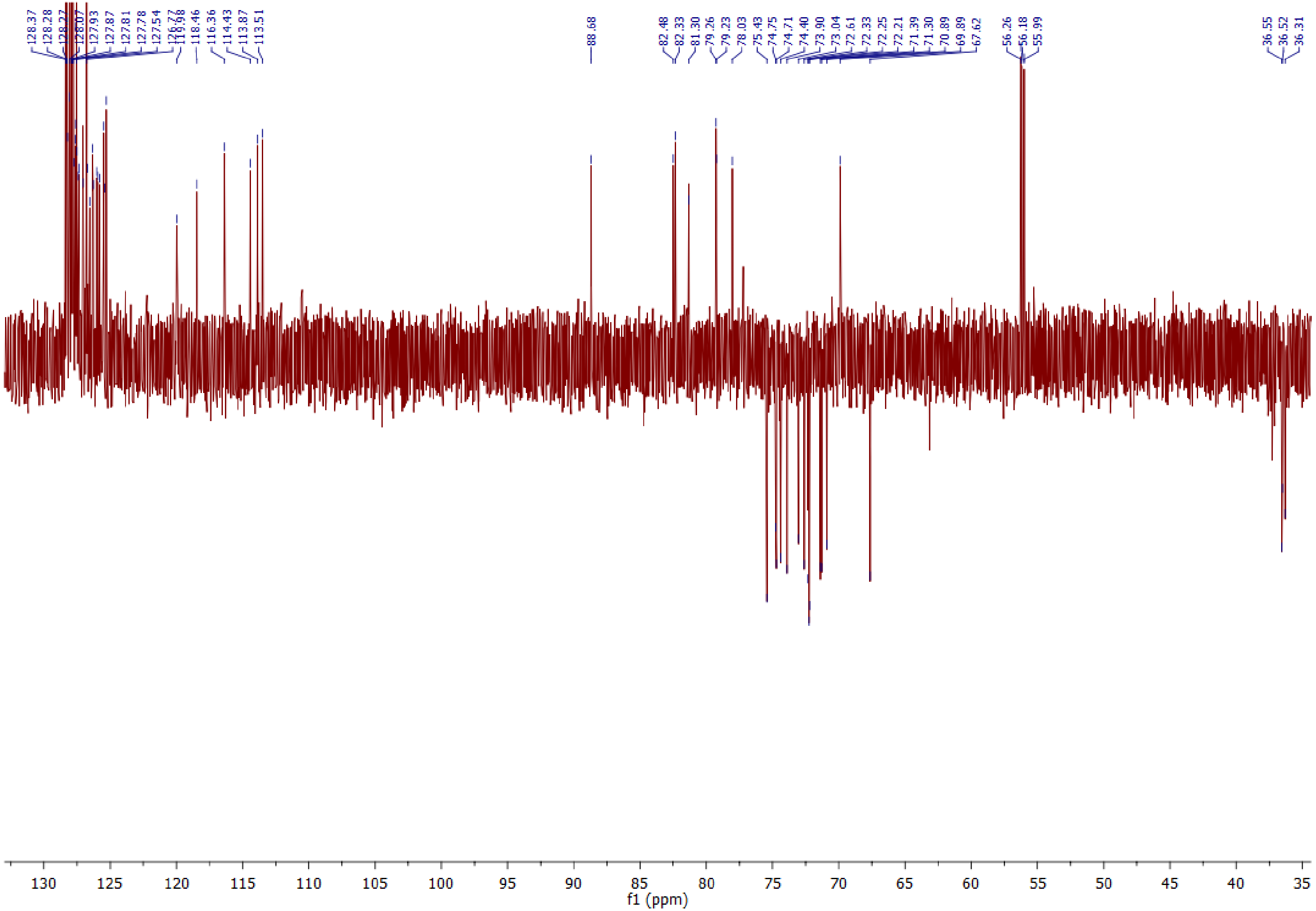

Figure S55. $^{13}C\{^{1}H\}$ DEPT (150 MHz, $CDCl_3$) spectrum of compound ***P*-5b.**

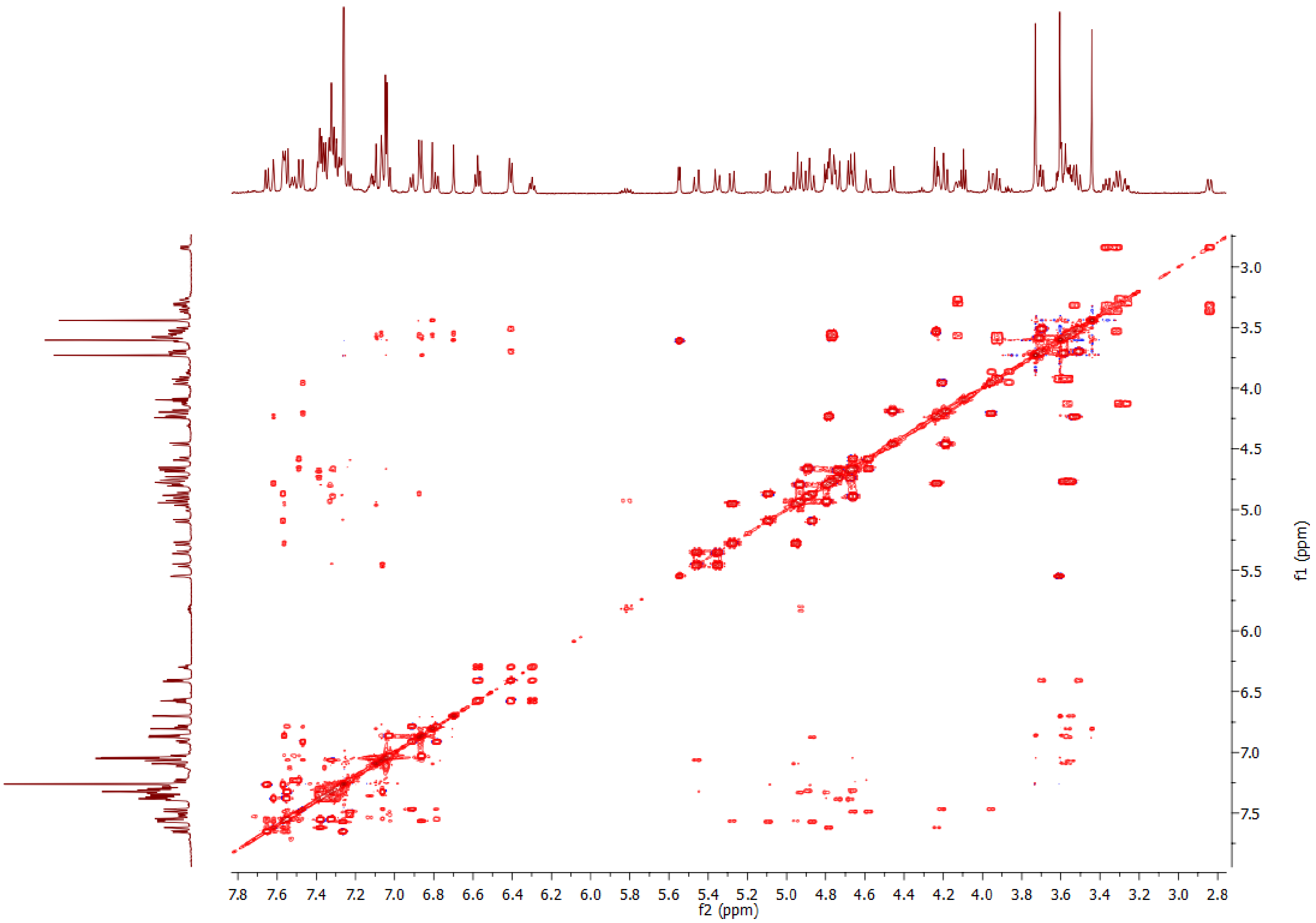

Figure S56. ^{1}H–^{1}H COSY (600 MHz, $CDCl_3$) spectrum of compound ***P*-5b**.

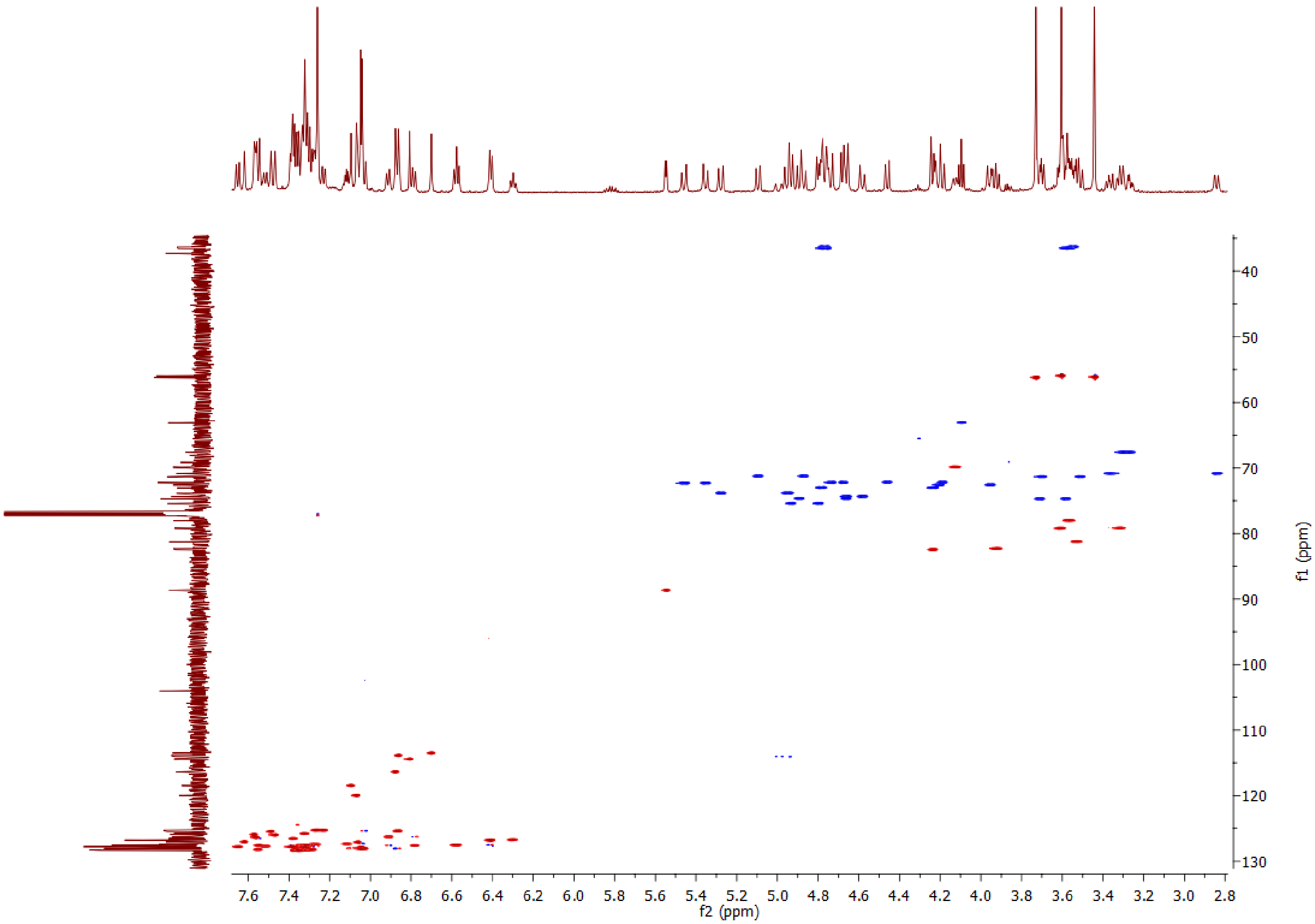

Figure S57. ^{1}H–^{13}C HSQC (600/150 MHz, $CDCl_3$) spectrum of compound ***P*-5b**.

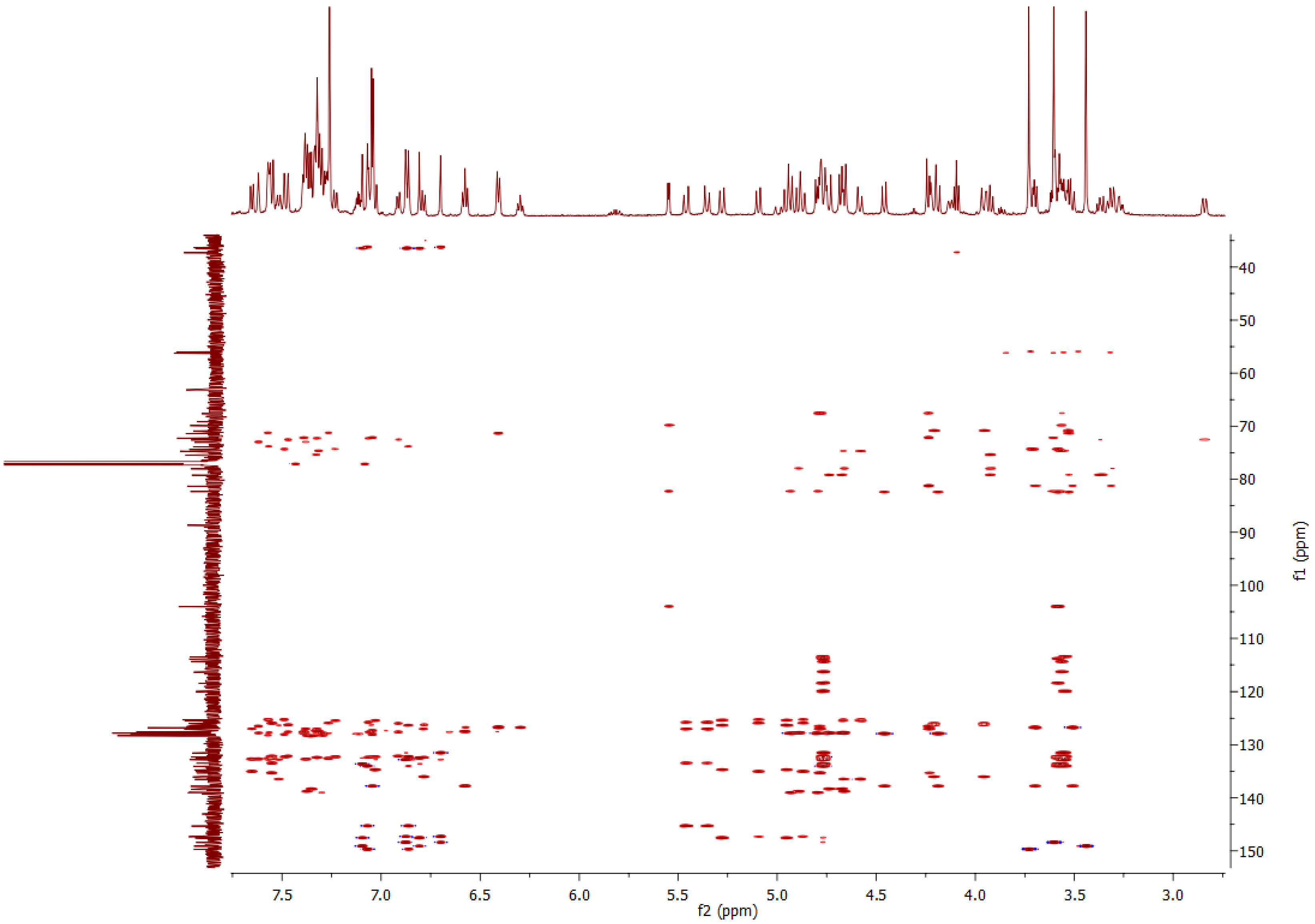

Figure S58. $^{1}H–^{13}C$ HMBC (600/150 MHz, $CDCl_3$) spectrum of compound ***P*-5b.**

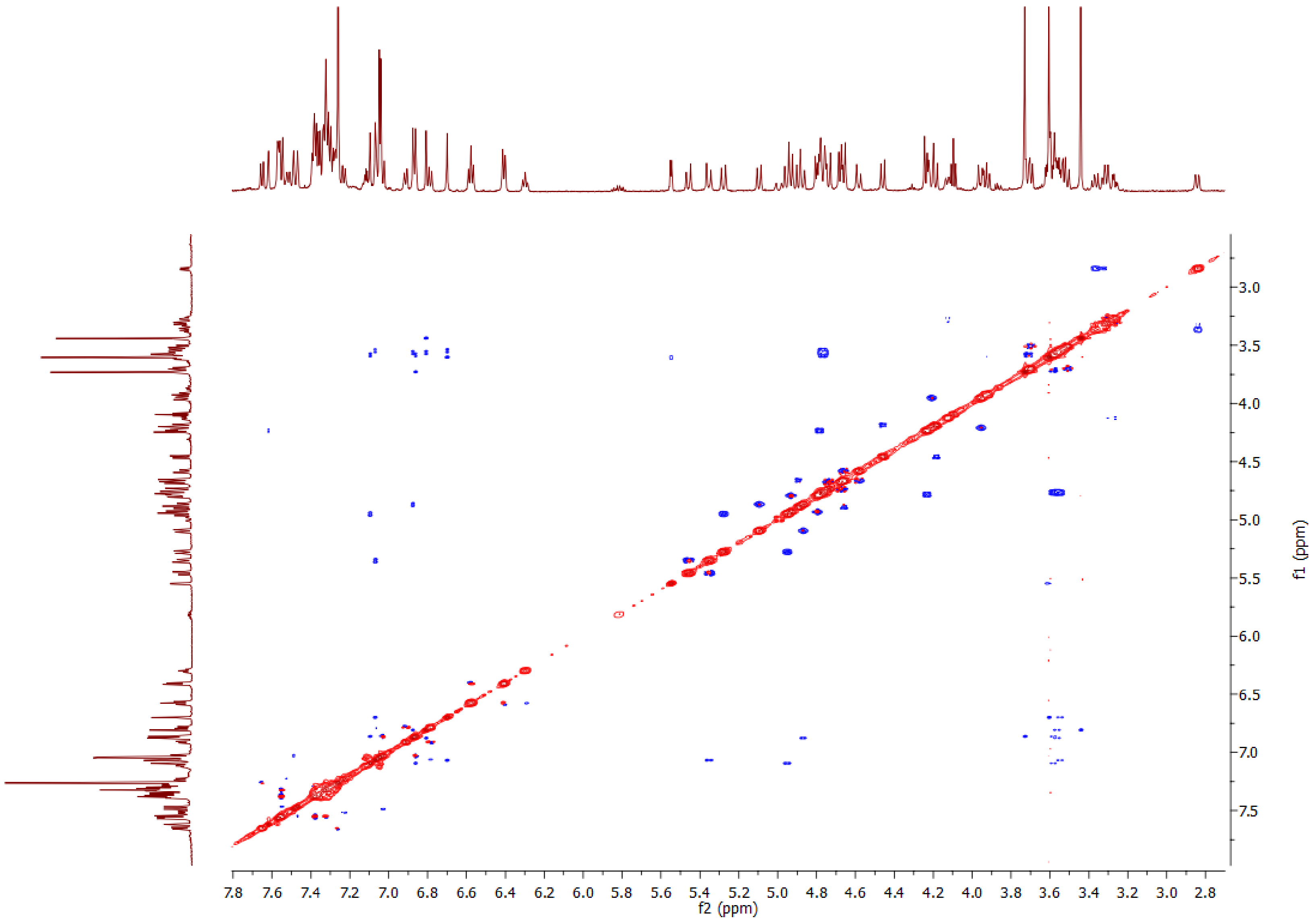

Figure S59. ^{1}H–^{1}H ROESY (600 MHz, $CDCl_3$) spectrum of compound ***P*-5b.**

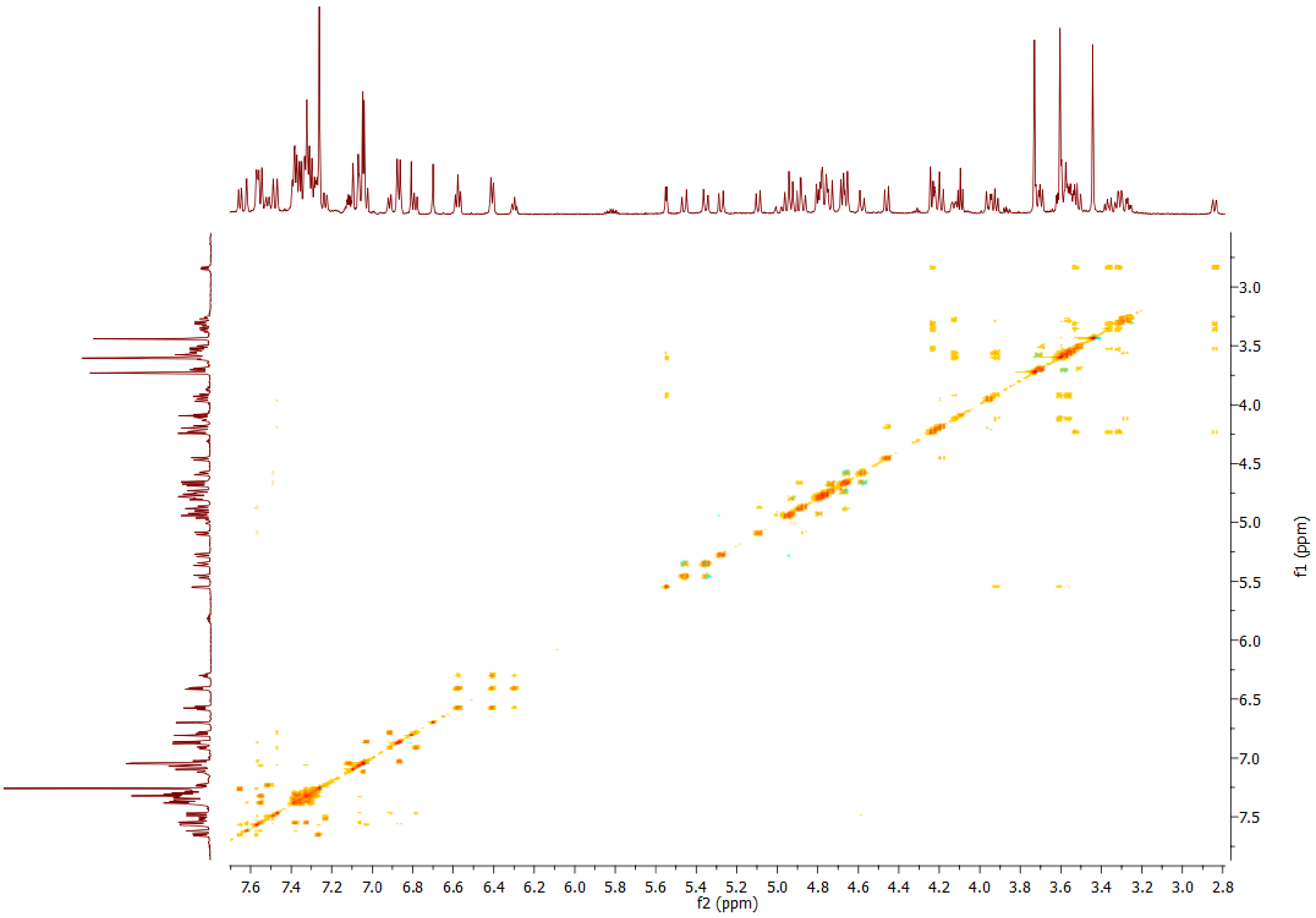

Figure S60. ^{1}H–^{1}H TOCSY (600 MHz, $CDCl_3$) spectrum of compound ***P*-5b.**

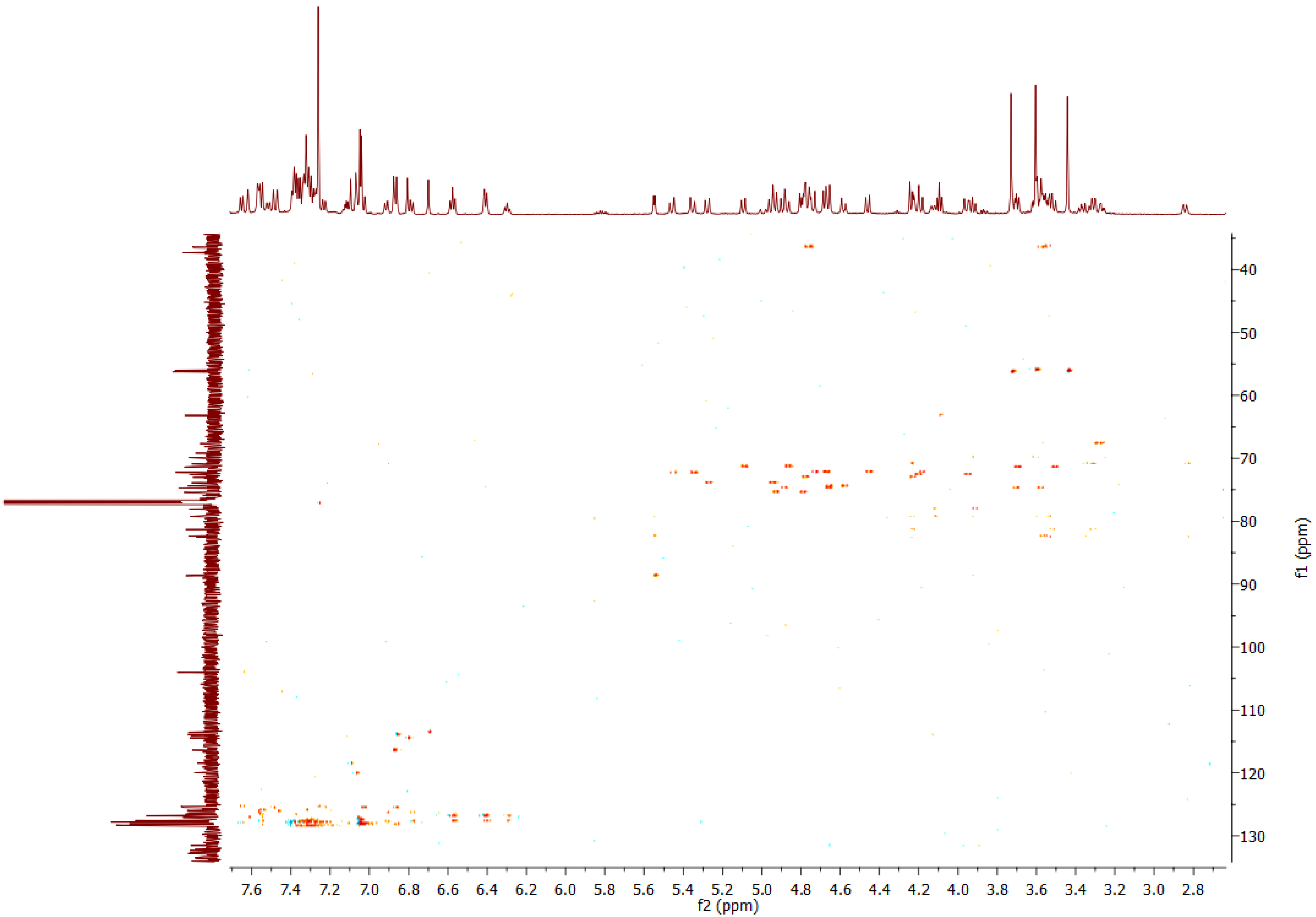

Figure S61. ^{1}H–^{13}C HSQC-TOCSY (600/150 MHz, $CDCl_3$) spectrum of compound ***P*-5b.**

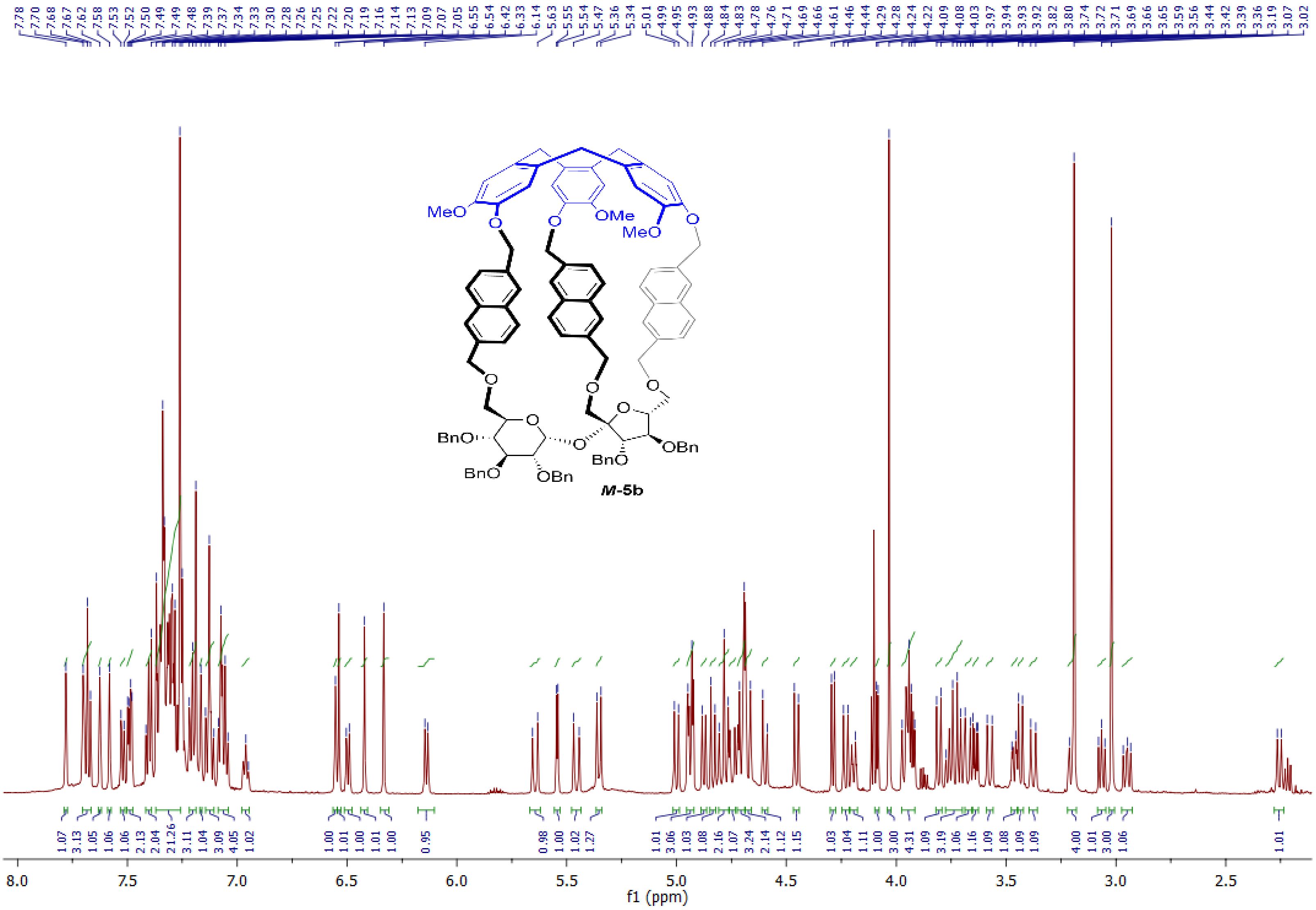

Figure S62. ^{1}H NMR (600 MHz, $CDCl_3$) spectrum of compound ***M*-5b.**

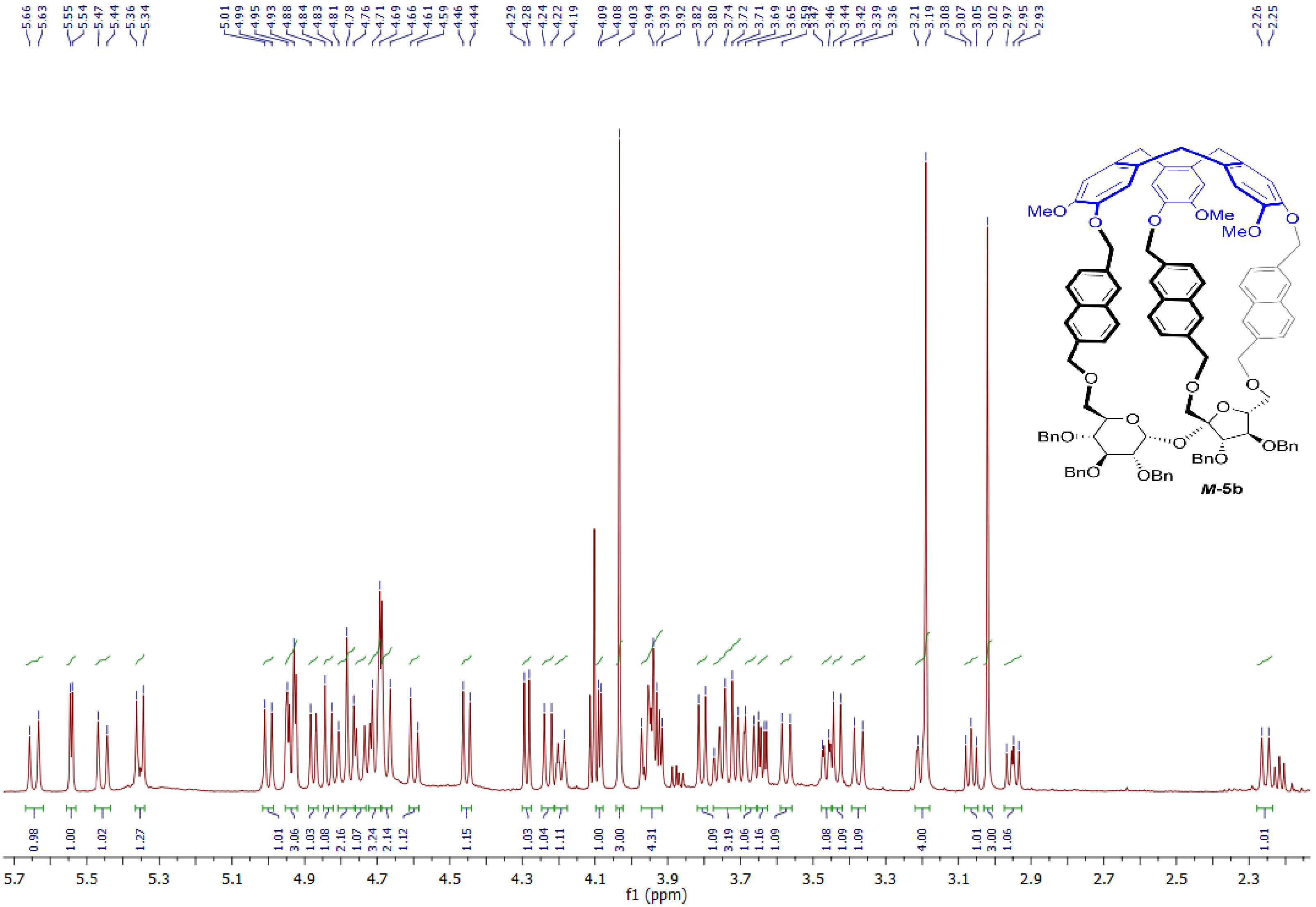

Figure S63. ^{1}H NMR (600 MHz, $CDCl_3$) spectrum of compound ***M*-5b** (aliphatic part).

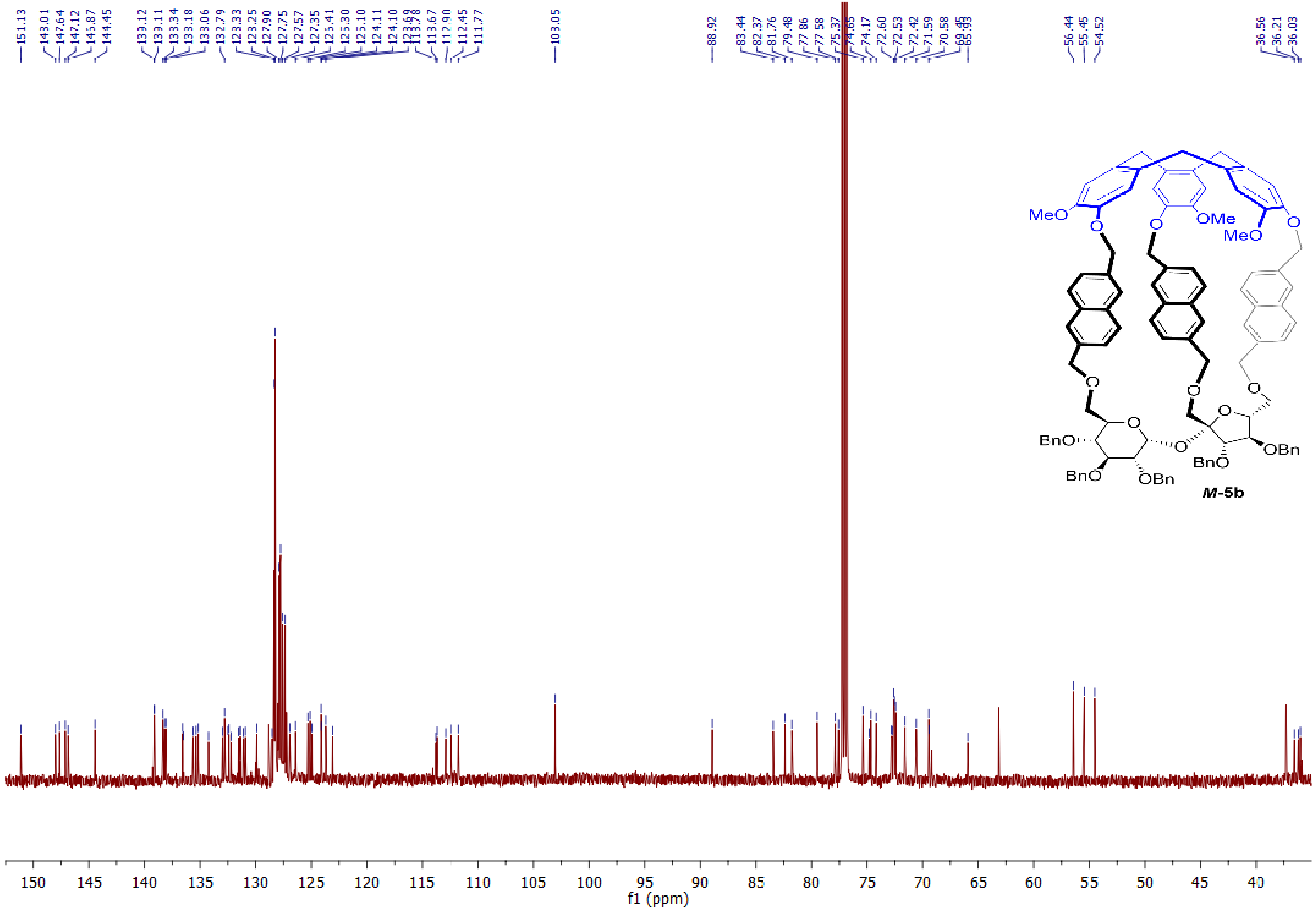

Figure S64. $^{13}C\{^{1}H\}$ NMR (150 MHz, $CDCl_3$) spectrum of compound ***M*-5b.**

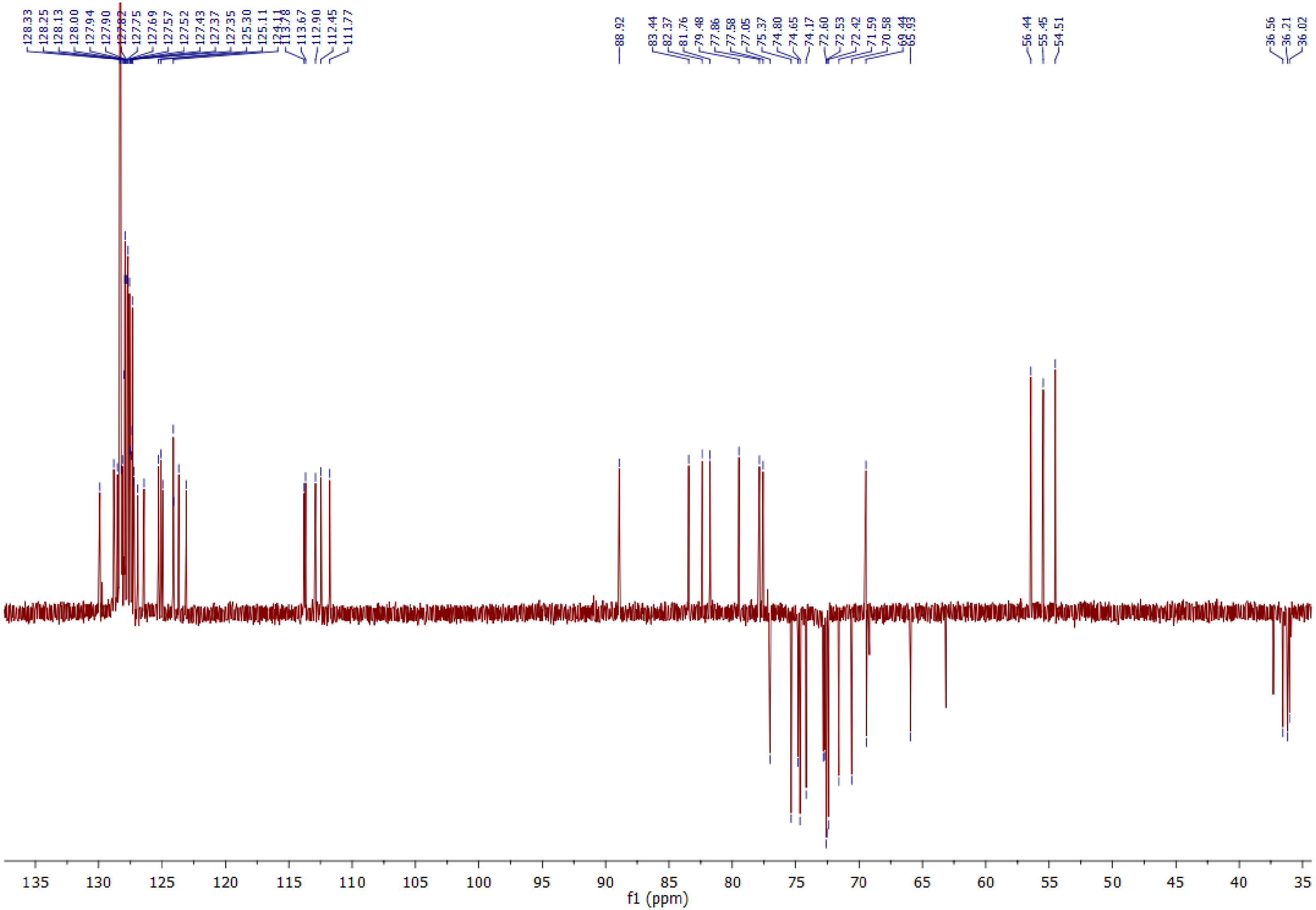

Figure S65. $^{13}C\{^{1}H\}$ DEPT (150 MHz, $CDCl_3$) spectrum of compound ***M*-5b.**

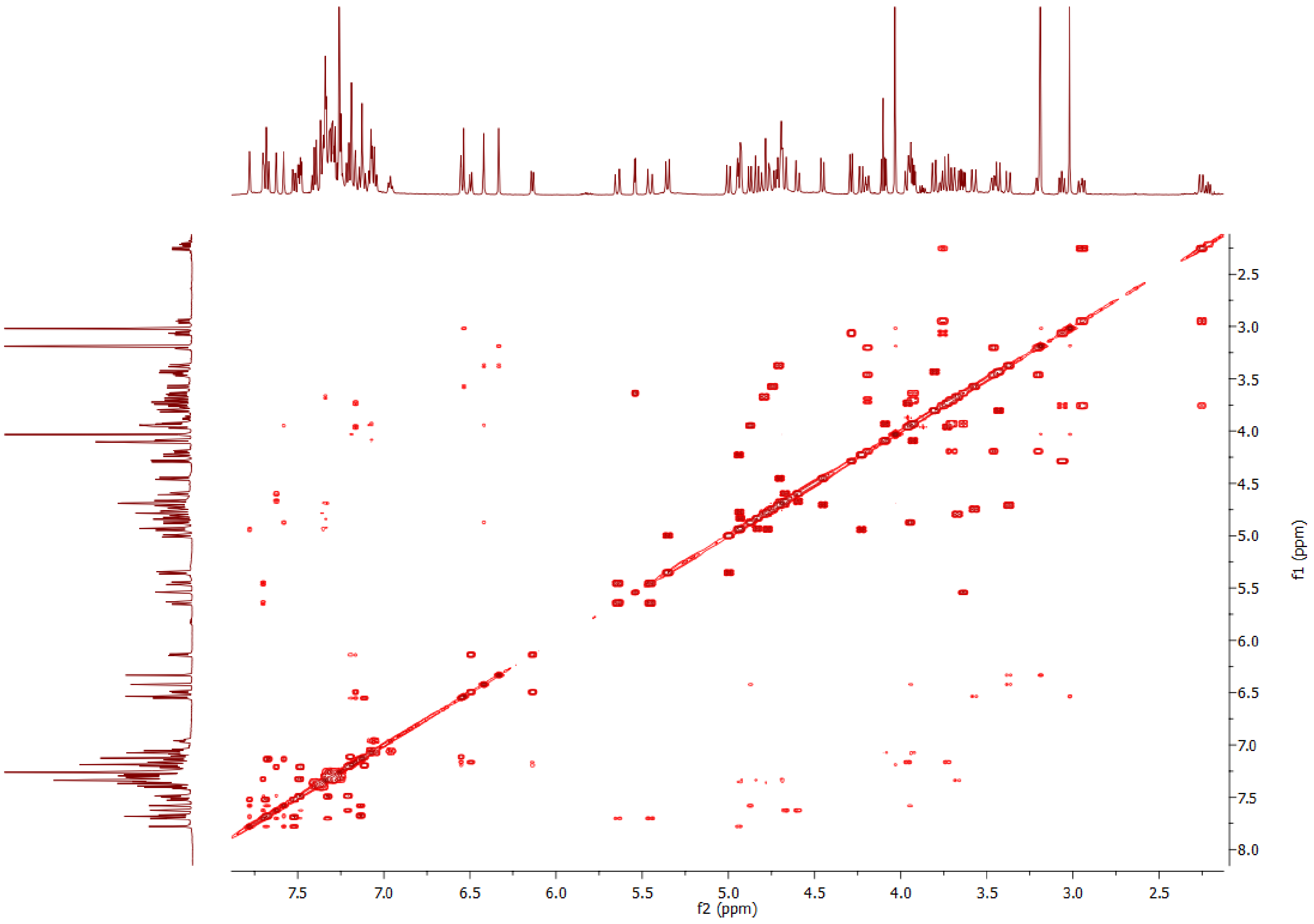

Figure S66. ^{1}H–^{1}H COSY (600 MHz, $CDCl_3$) spectrum of compound ***M*-5b**.

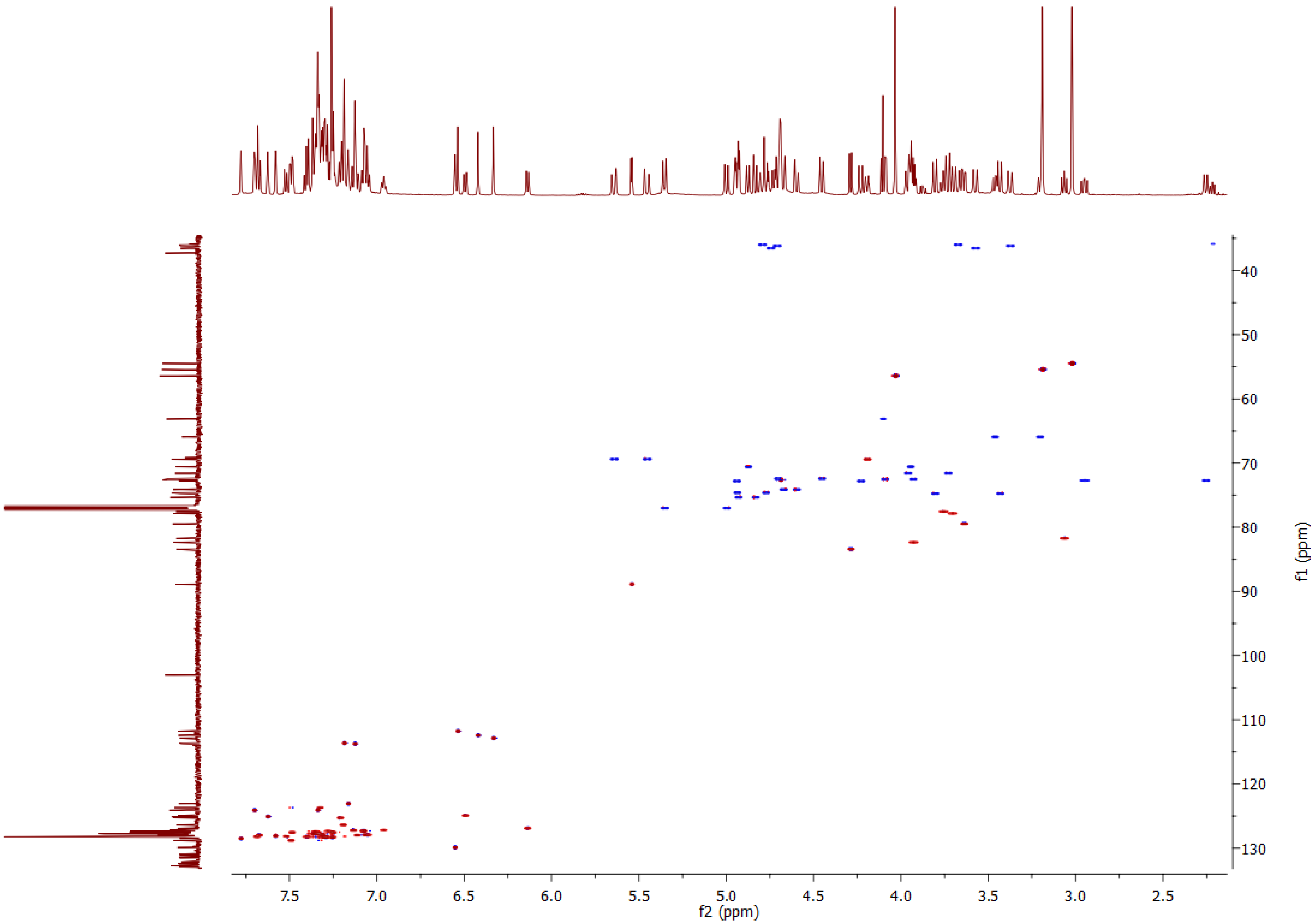

Figure S67. ^{1}H–^{13}C HSQC (600/150 MHz, $CDCl_3$) spectrum of compound ***M*-5b**.

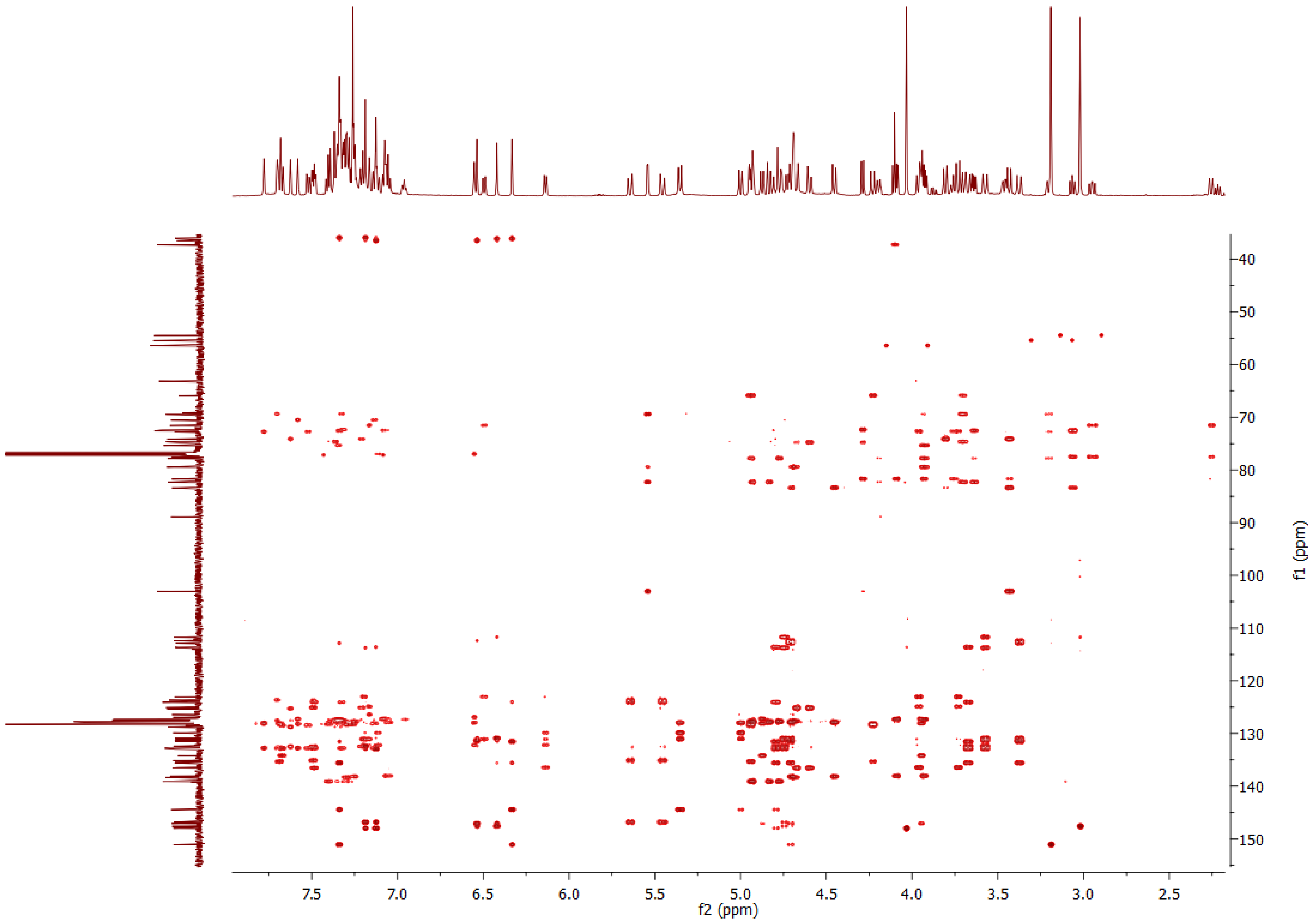

Figure S68. ^{1}H–^{13}C HMBC (600/150 MHz, $CDCl_3$) spectrum of compound ***M*-5b.**

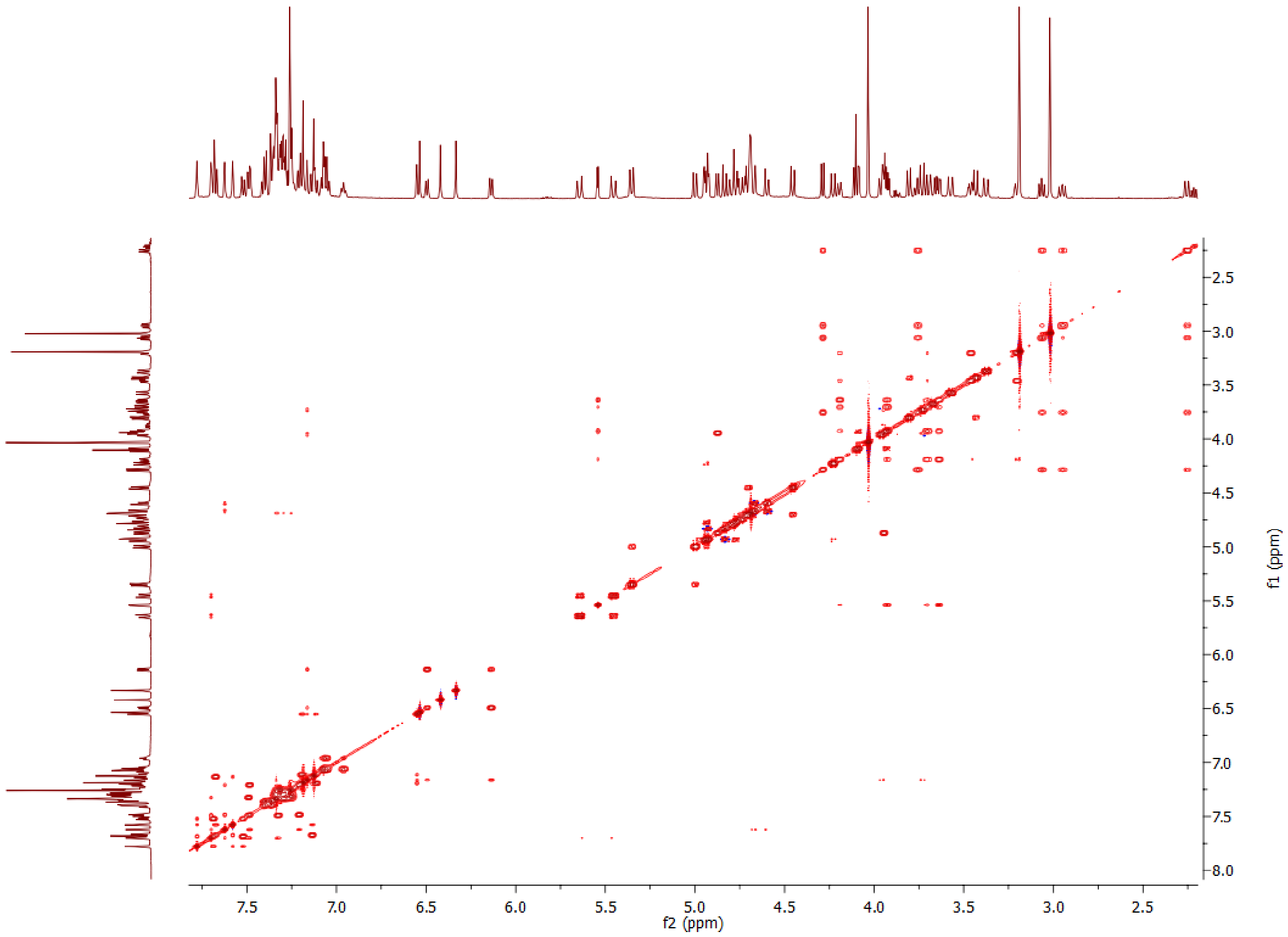

Figure S69. ^{1}H–^{1}H TOCSY (600 MHz, $CDCl_3$) spectrum of compound ***M*-5b.**

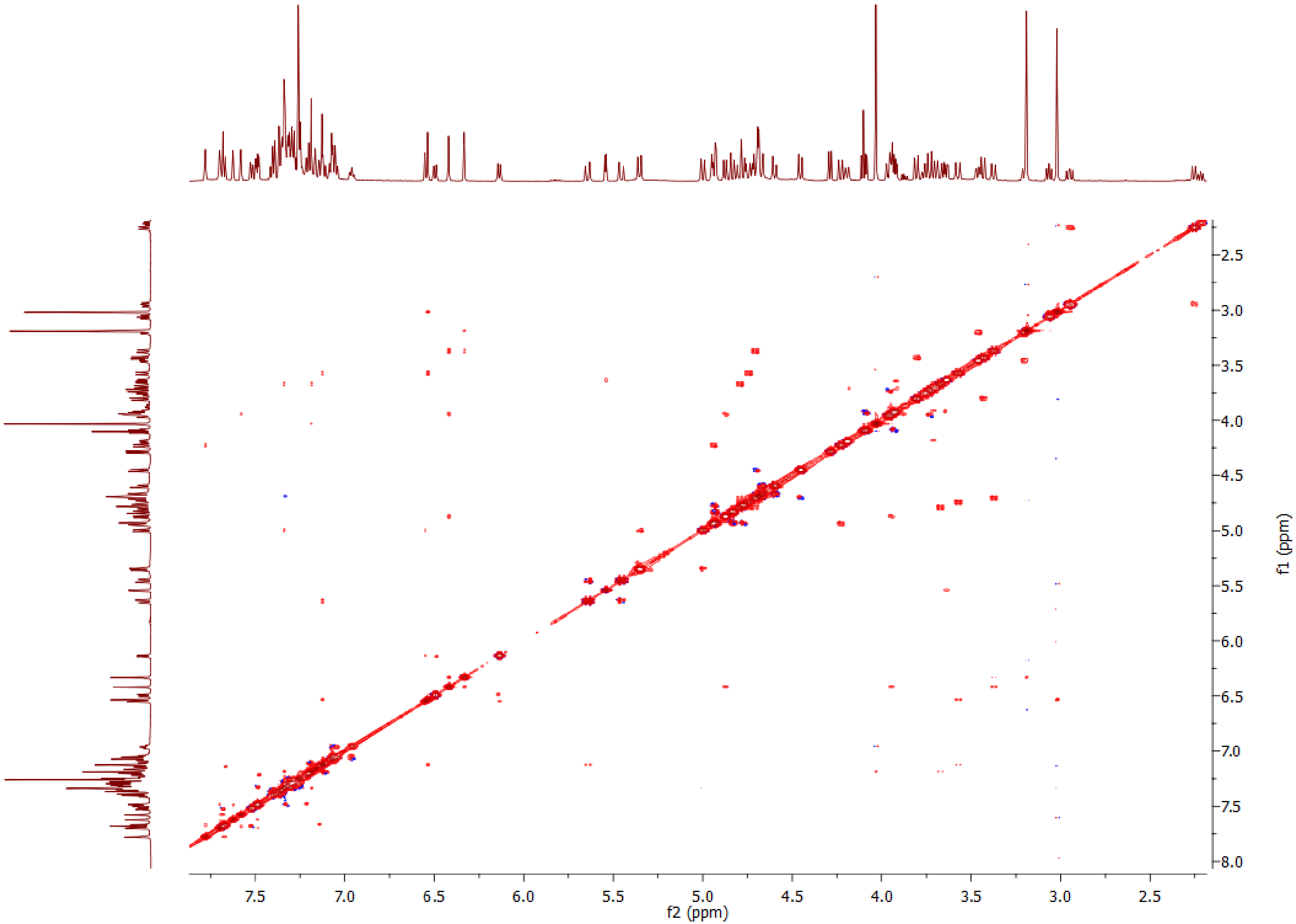

Figure S70. $^{1}H–^{1}H$ NOESY (600 MHz, $CDCl_3$) spectrum of compound ***M*-5b.**

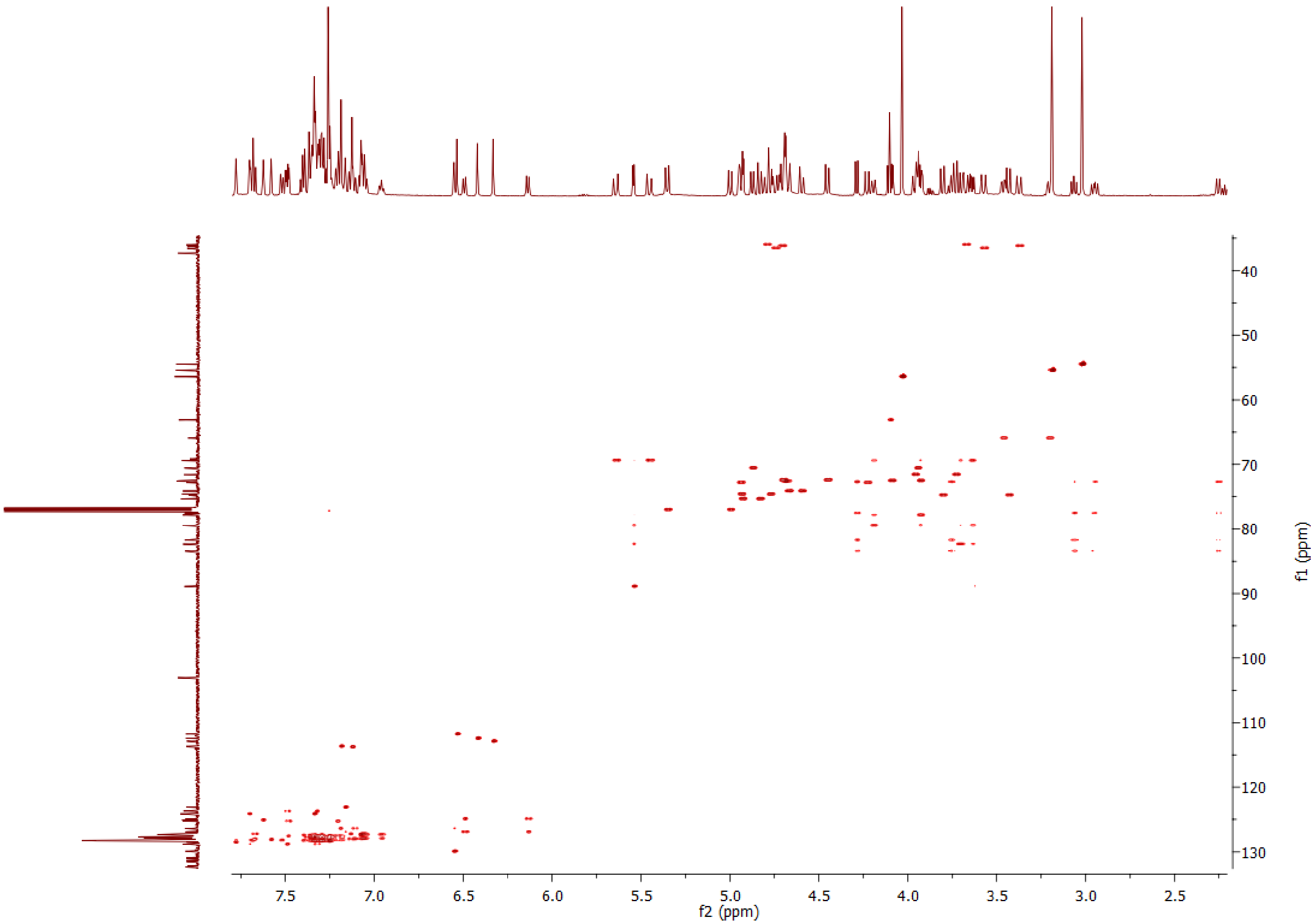

Figure S71. ^{1}H–^{13}C HSQC-TOCSY (600/150 MHz, $CDCl_3$) spectrum of compound ***M*-5b.**

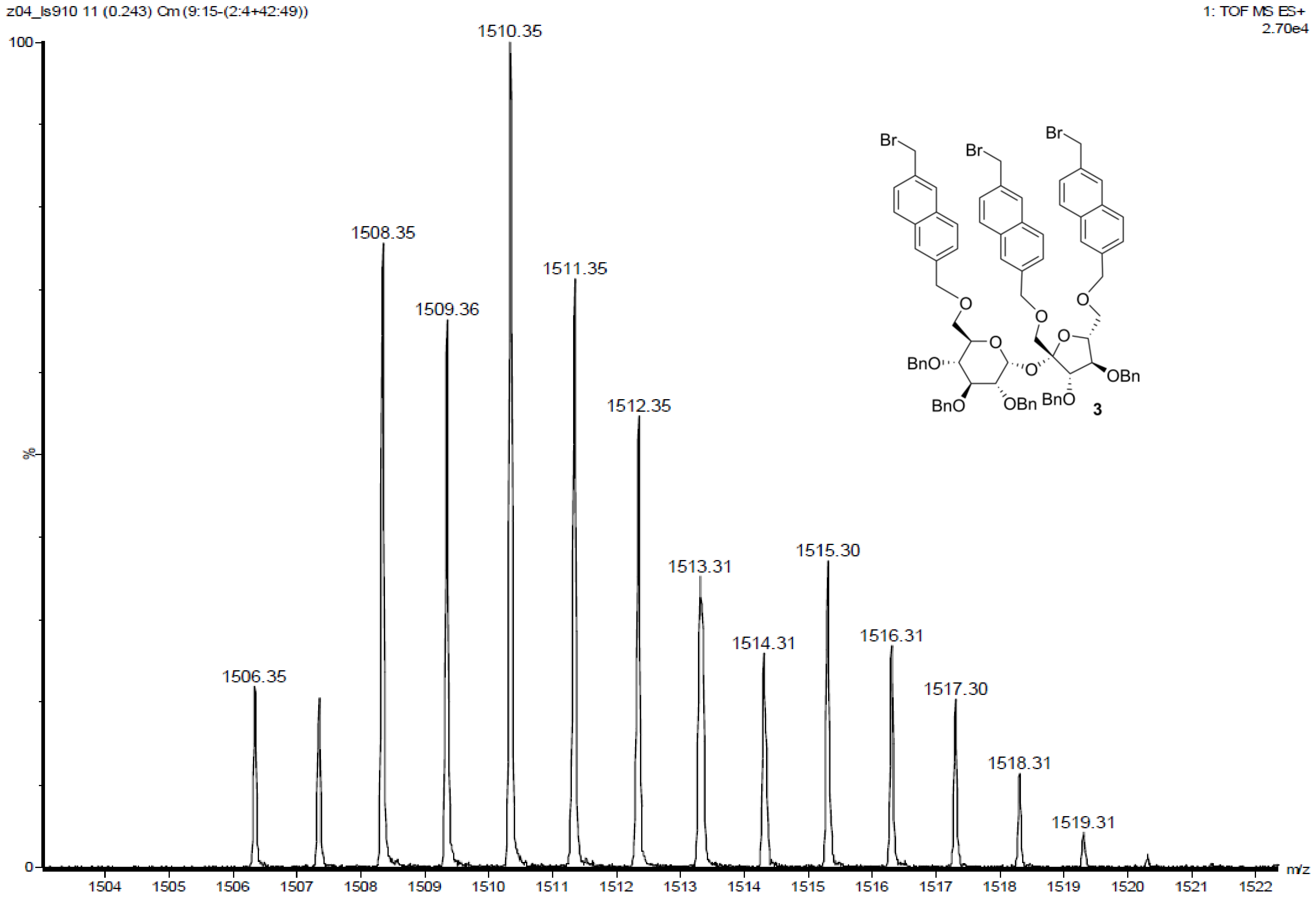

Figure S72. ESI-MS spectrum of compound **3.**

Elements Used:
C: 0-150 H: 0-150 O: 0-17 Na: 1-1

Mass	Calc. Mass	mDa	PPM	DBE	Formula	C	H	O	Na
1679.6844	1679.6858	-1.4	-0.8	57.5	C107 H100 O17 Na	107	100	17	1
	1679.6799	4.5	2.7	66.5	C114 H96 O12 Na	114	96	12	1
	1679.6893	-4.9	-2.9	79.5	C125 H92 O4 Na	125	92	4	1

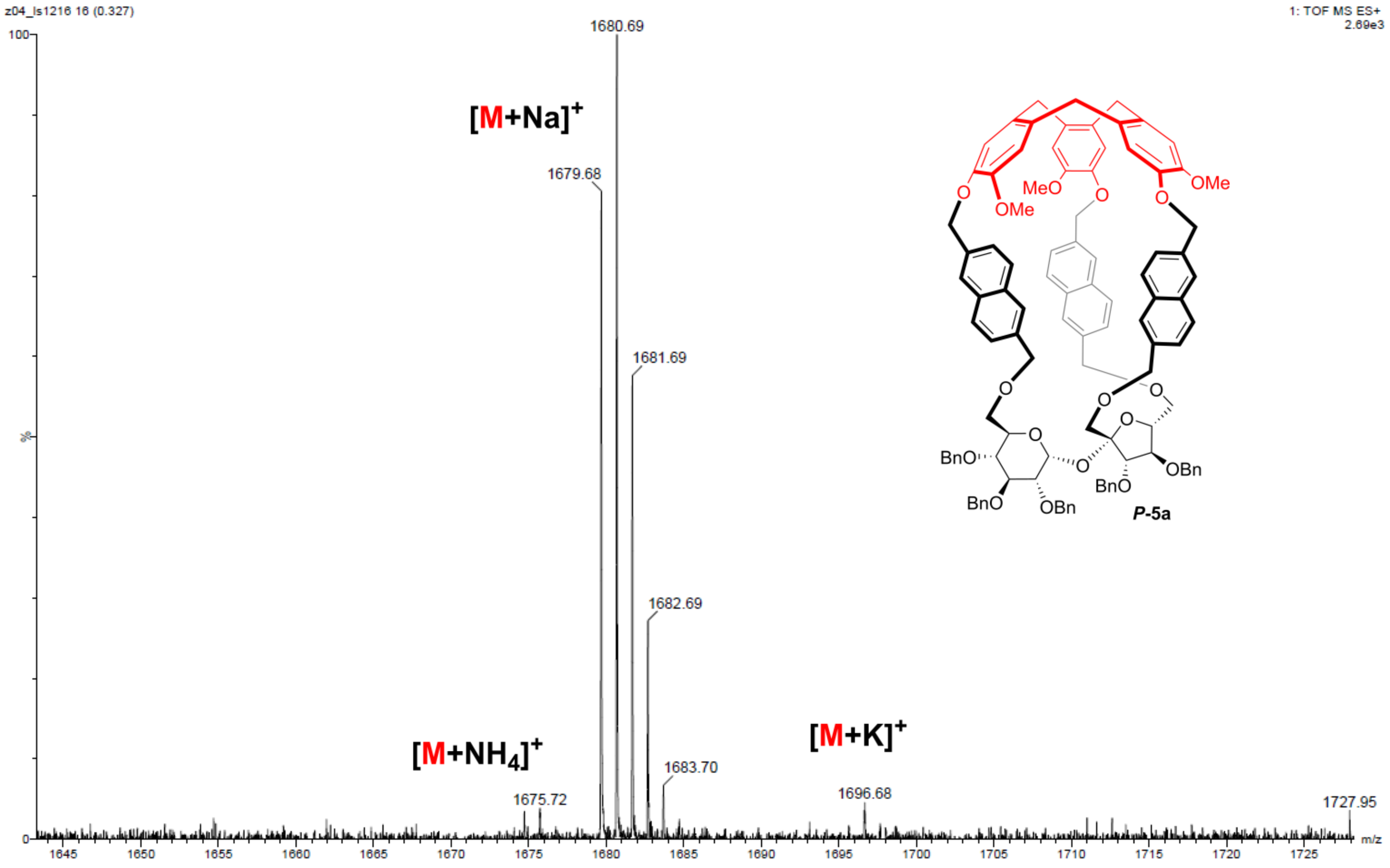

Figure S73. HRMS spectrum of compound ***P*-5a.**

Elements Used:
C: 0-150 H: 0-150 O: 0-17 Na: 0-1

Mass	Calc. Mass	mDa	PPM	DBE	Formula	i-FIT	i-FIT Norm	Fit Conf %	C	H	O	Na
1679.6866	1679.6858	0.8	0.5	57.5	C107 H100 O17 Na	152.4	0.155	85.63	107	100	17	1
	1679.6882	-1.6	-1.0	60.5	C109 H99 O17	154.2	1.940	14.36	109	99	17	
	1679.6893	-2.7	-1.6	79.5	C125 H92 O4 Na	162.4	10.119	0.00	125	92	4	1

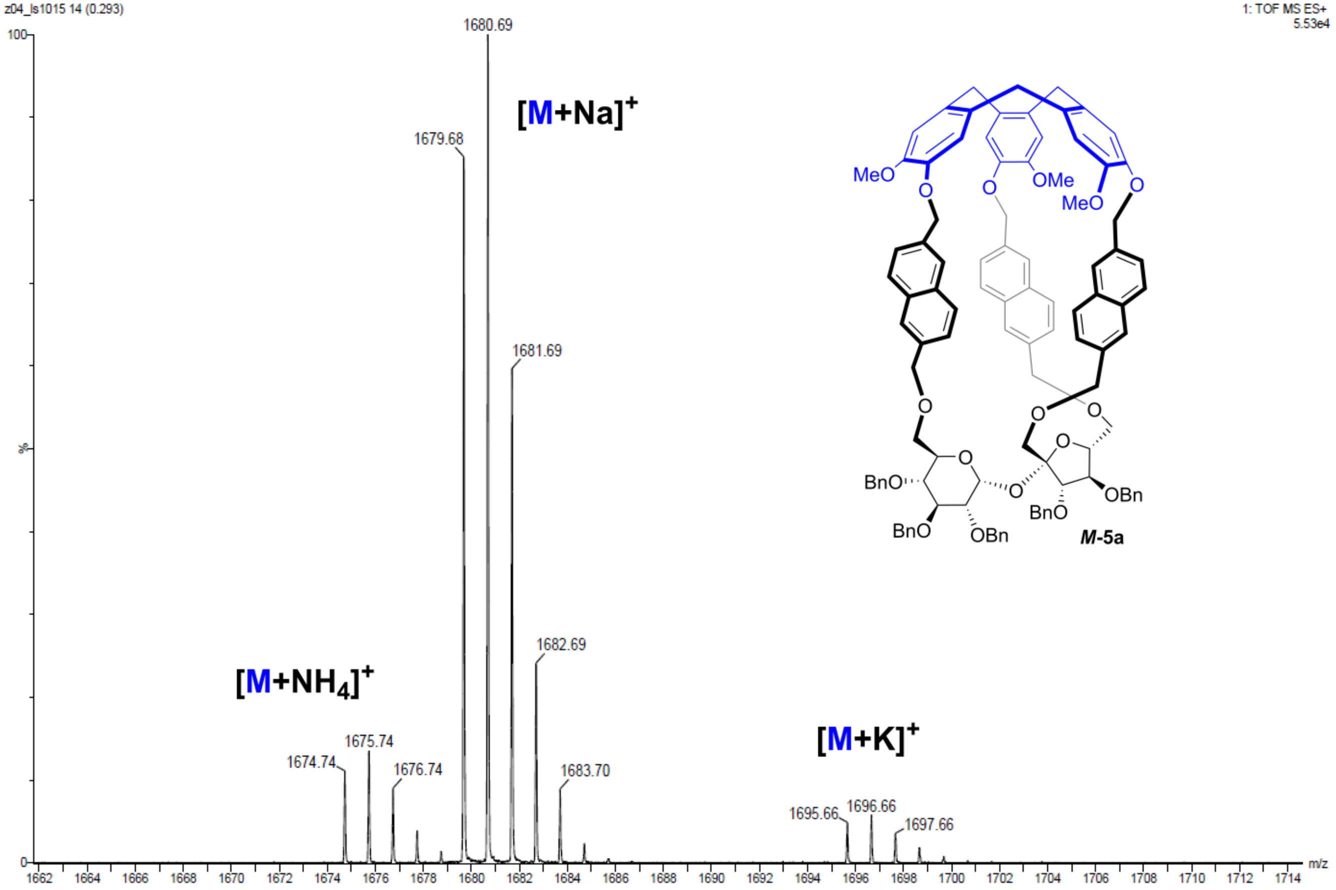

Figure S74. HRMS spectrum of compound ***M*-5a.**

Elements Used:
C: 0-150 H: 0-150 O: 0-20 Na: 1-1

Mass	Calc. Mass	mDa	PPM	DBE	Formula	i-FIT	i-FIT Norm	Fit Conf %	C	H	O	Na
1679.6836	1679.6858	-2.2	-1.3	57.5	C107 H100 O17 Na	97.7	n/a	n/a	107	100	17	1

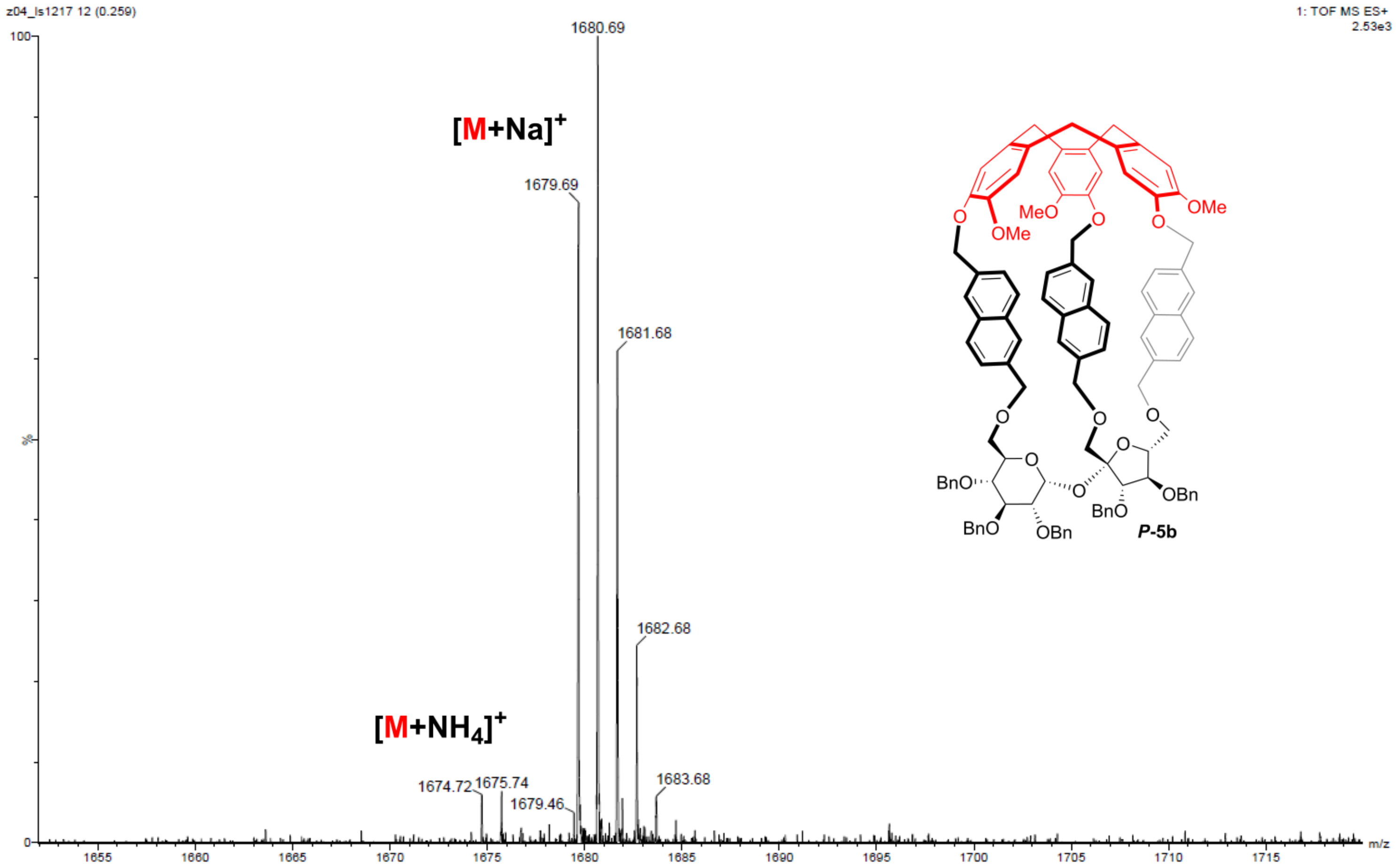

Figure S75. HRMS spectrum of compound ***P*-5b.**

Elements Used:
C: 0-150 H: 0-150 O: 0-17 Na: 0-1

Mass	Calc. Mass	mDa	PPM	DBE	Formula	i-FIT	i-FIT Norm	Fit Conf %	C	H	O	Na
1679.6860	1679.6882	-2.2	-1.3	60.5	C109 H99 O17	154.8	1.985	13.73	109	99	17	
	1679.6858	0.2	0.1	57.5	C107 H100 O17 Na	152.9	0.148	86.27	107	100	17	1

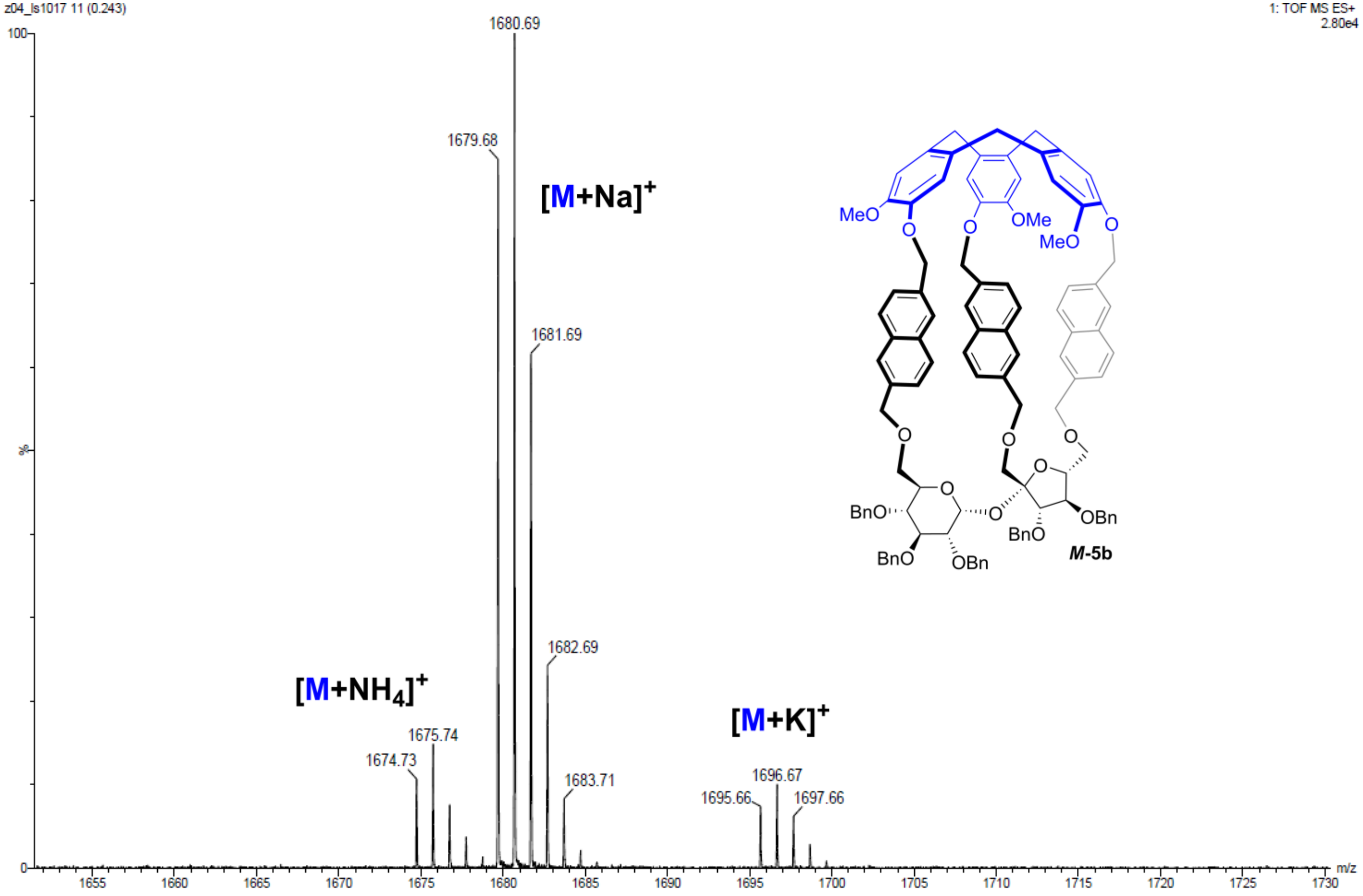

Figure S76. HRMS spectrum of compound ***M*-5b.**

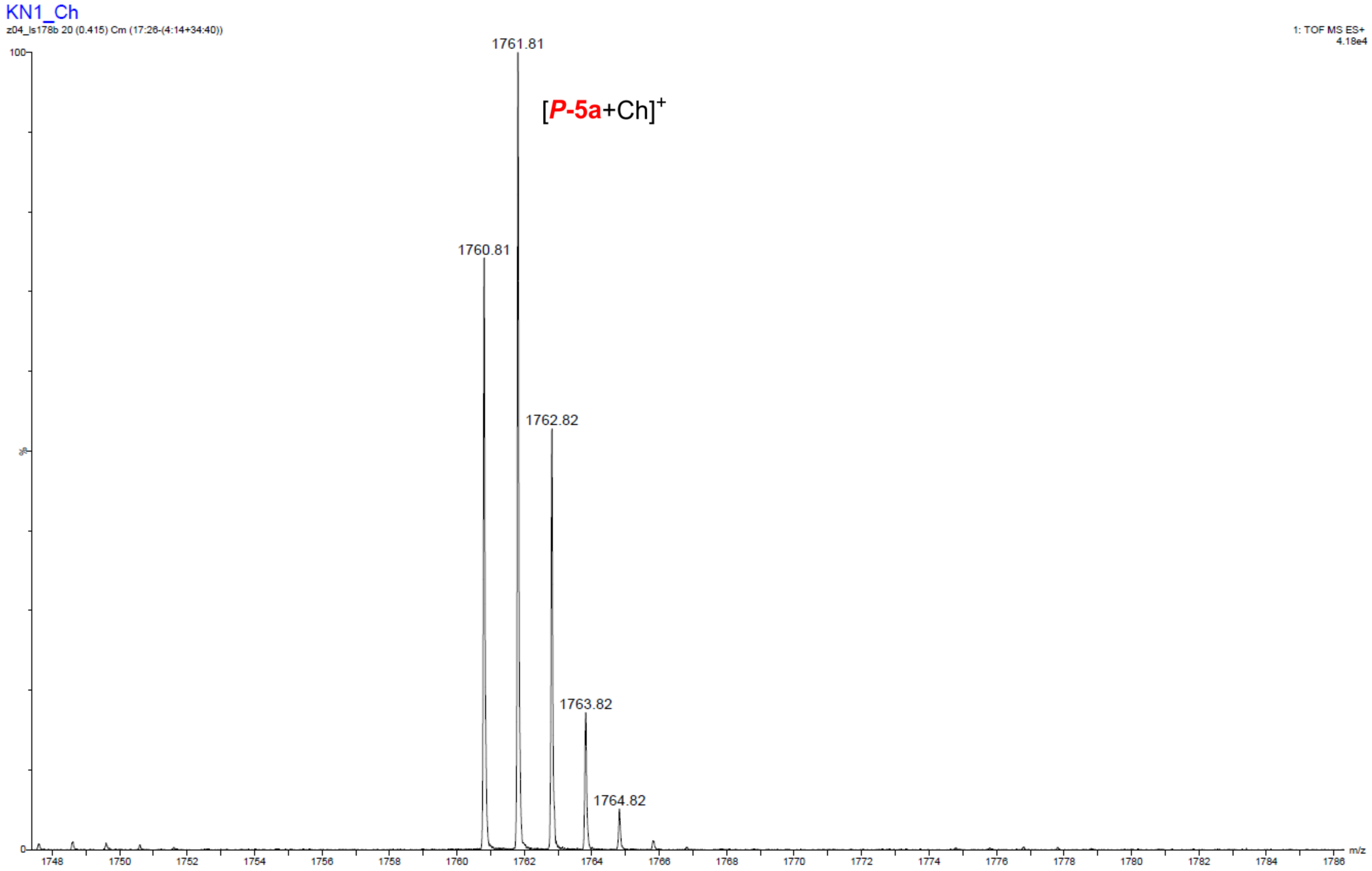

Figure S77. ESI-MS spectrum of adduct [***P*-5a** + Ch]$^+$.

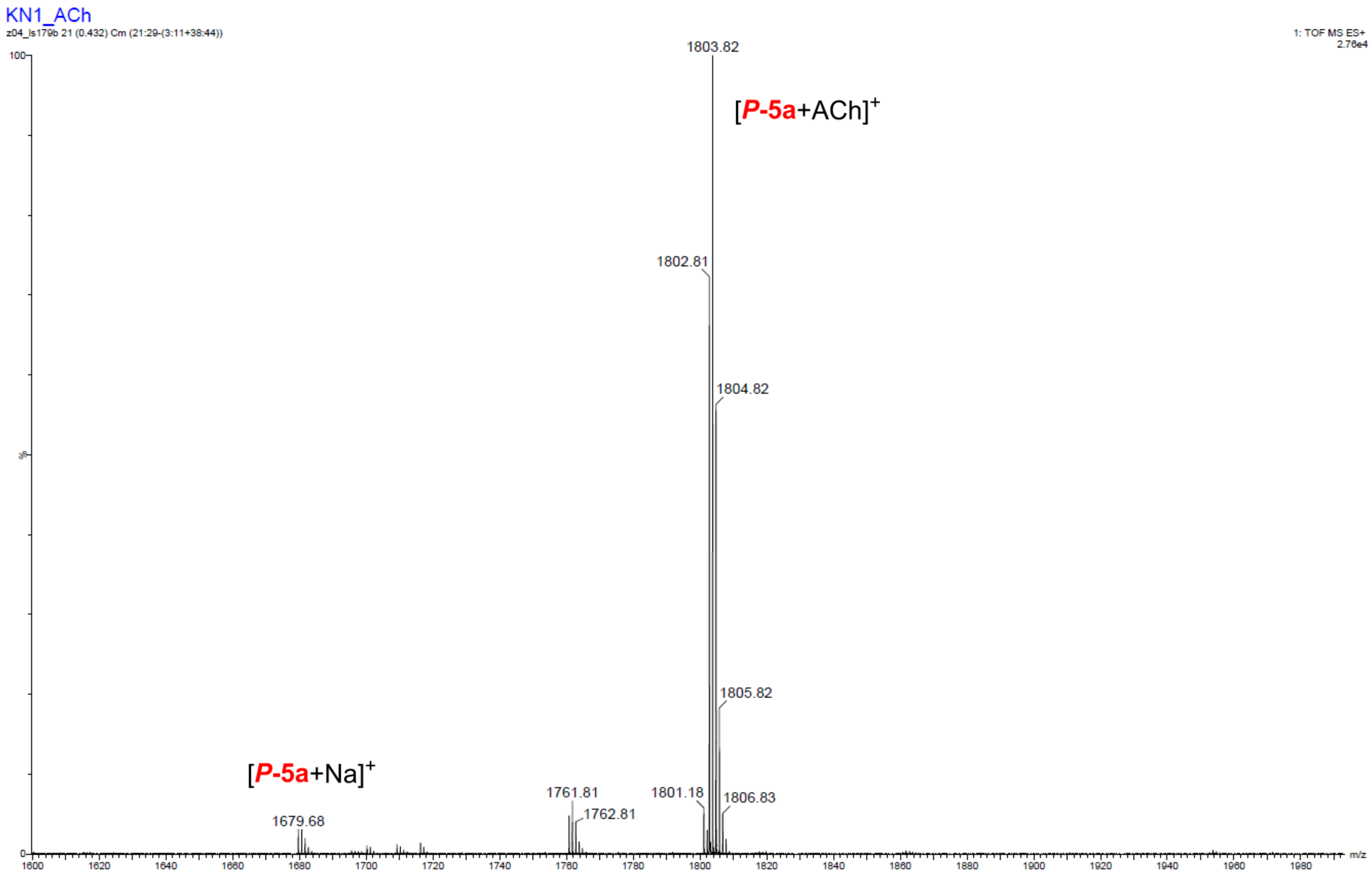

Figure S78. ESI-MS spectrum of adduct [***P*-5a** + ACh]$^+$.

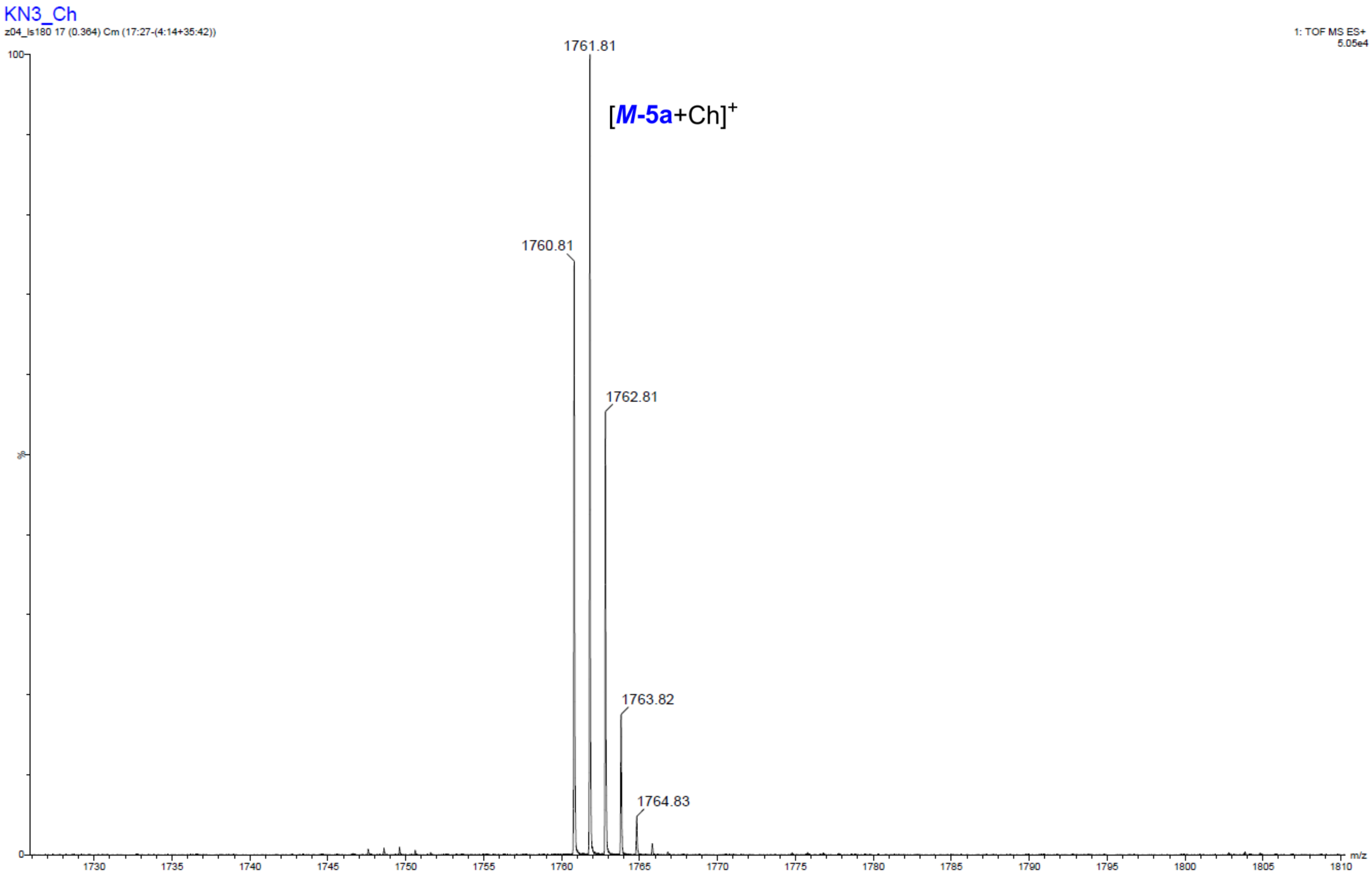

Figure S79. ESI-MS spectrum of adduct [***M*-5a** + Ch]$^+$.

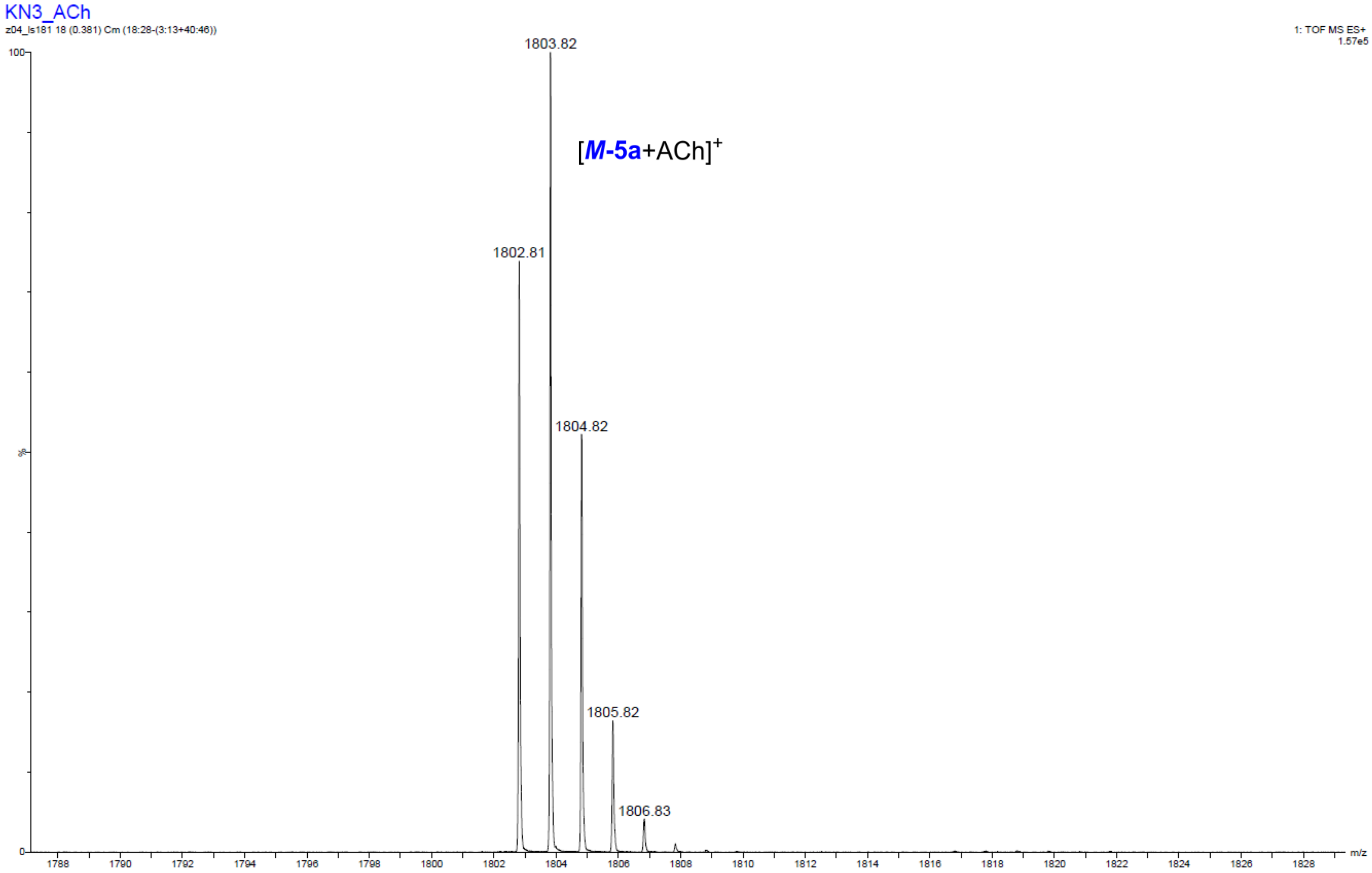

Figure S80. ESI-MS spectrum of adduct [***M*-5a** + ACh]$^+$.

Geometry of calculated structures

P-5a

Conformation #1 (Pop. = 68.98%)

Symbolic Z-matrix:

C	7.04048300	2.16352500	0.03090500
C	7.39308900	1.65453100	-1.36290000
C	6.15137000	1.04199700	-2.03362000
C	4.70910400	2.52736100	-0.81529100
C	5.88632200	3.15908300	-0.04844500
O	5.14509900	2.03469400	-2.08835300
O	8.23163000	2.74531400	0.55846900
O	8.43143400	0.68181500	-1.29949700
O	5.76710000	-0.09268500	-1.27973100
O	5.52997300	3.51344000	1.28554900
C	3.59479100	3.52440700	-1.12896300
O	3.04611100	3.96104700	0.11556800
C	5.78723300	-3.21146000	-0.47286000
C	7.13560800	-2.82310900	-1.06694500
C	5.42737000	-1.34221700	-1.89827700
C	6.69760500	-2.11835900	-2.35525100
O	4.90001800	-2.12649200	-0.83860900
O	7.69496700	-1.36726700	-3.00401900
C	4.38803900	-1.21957600	-3.03351500
C	5.72144800	-3.45721300	1.02269700
O	3.37239700	-0.25443300	-2.86726100
H	7.71678400	2.50380400	-1.98078800
H	6.38495900	-2.89239500	-3.06824900
H	7.64124500	-2.10057000	-0.41267500
H	5.44250100	-4.13044600	-0.96944500
H	6.72932200	1.31028400	0.64925800
H	6.23206700	4.04788700	-0.60103500
H	4.29579100	1.70708400	-0.21597800
H	3.99499700	4.38130200	-1.69133600
H	2.83073200	3.03592700	-1.74565100
H	3.97809400	-2.22910400	-3.18777100
H	4.66352700	-3.50728800	1.31666400
H	4.90559400	-0.92962500	-3.95451600
C	-3.91871600	-4.22891900	1.07533900
C	-4.24732300	-3.31916200	2.08119500
C	-4.74506300	-4.30039100	-0.06495900
C	-5.36360800	-2.46839500	1.99093700
H	-3.62937800	-3.26423800	2.97038700
C	-5.86599600	-3.48308500	-0.14066100
C	-6.19771800	-2.55626300	0.86226700
H	-6.51555400	-3.58276000	-1.00598700
C	-2.93528500	1.13594000	3.72625400
C	-3.60205000	2.32731800	3.36573000
C	-3.61897300	-0.07222200	3.62803600
C	-4.88042900	2.23454600	2.81708500
C	-4.92811100	-0.16774500	3.12797800
H	-3.10086600	-0.96407800	3.97056600
C	-5.54983300	1.00862300	2.67200300
H	-5.39399900	3.14048700	2.51561900
C	-6.49849900	1.81162900	-1.71158100

C	-6.87819400	0.59694300	-2.32822800
C	-6.53004600	1.89873800	-0.32563100
C	-7.18659700	-0.49533400	-1.52112800
C	-6.87856000	0.81174100	0.49627200
H	-6.28044700	2.85868100	0.11780300
C	-7.17278600	-0.41695400	-0.11614900
O	-6.92835600	0.59662200	-3.69387500
O	-2.94794100	3.50327900	3.60478400
C	-7.35670600	-0.59186100	-4.35330400
H	-7.34630400	-0.36000700	-5.41920600
H	-6.67641300	-1.42931200	-4.15640600
H	-8.37247400	-0.87142300	-4.04914000
C	-3.57219200	4.71924800	3.20152600
H	-3.75084500	4.73712900	2.11982100
H	-4.52002000	4.87617500	3.73015900
H	-2.87314700	5.51318400	3.46805700
C	-5.62458900	-1.52806000	3.16474400
H	-5.29029600	-2.03887000	4.07489800
H	-6.70021700	-1.38238800	3.29137700
C	-6.91916500	1.03571200	2.00665600
H	-7.36665100	2.01945900	2.18848300
H	-7.59071000	0.31441900	2.47754300
H	-7.46399600	-1.43645000	-1.98242100
C	-7.44697200	-1.70258000	0.65703000
H	-8.17342600	-2.29611800	0.09055700
H	-7.92378000	-1.48543900	1.61531100
H	6.35759800	0.78008800	-3.07191800
H	4.67478000	3.98218300	1.22328100
H	9.09858900	1.01390700	-0.67391100
H	8.08963200	-0.74649600	-2.35320900
H	8.04976200	2.98128600	1.48271600
O	-2.85708800	-5.08452700	1.11457900
C	-1.99695400	-5.03240400	2.24872000
H	-1.53224700	-4.04458000	2.35486100
H	-2.53444300	-5.28125100	3.17203500
H	-1.22269800	-5.77975400	2.06864100
O	6.40144200	-2.43619800	1.73430800
H	6.17509900	-4.43805600	1.23680900
O	-6.21349100	2.93729400	-2.45149000
O	-1.66539800	1.11615200	4.25930500
C	-0.66778500	1.92290100	3.61979200
H	-0.59496200	2.88821600	4.13264500
H	-0.97008300	2.13412200	2.58652700
O	7.93010200	-3.97890100	-1.27194100
H	8.75219300	-3.68481400	-1.69768600
C	1.97535100	4.90032200	-0.00080400
H	2.28806800	5.75900200	-0.61169400
H	1.81705900	5.26091800	1.02167700
C	0.69146500	4.30565200	-0.55131900
C	-0.17740000	5.08306200	-1.28909700
C	0.34388000	2.95529100	-0.26839800
C	-1.41320400	4.56665200	-1.76549600
H	0.07569800	6.11664600	-1.51802500
C	-0.83290100	2.41575700	-0.73773400
H	1.02802400	2.34849400	0.31666000
C	-2.32711200	5.35410300	-2.51699300
C	-1.74379000	3.19776800	-1.50123500
H	-1.07783300	1.37706400	-0.53057200

C	-3.49646700	4.80922200	-2.99652200
H	-2.08388500	6.39485400	-2.71655600
C	-2.95075500	2.66449200	-2.02753500
C	-3.82013800	3.44311000	-2.76913900
H	-4.18077600	5.42298800	-3.57739600
H	-3.17610800	1.61484400	-1.85056000
C	2.50701500	-0.38592400	-1.72752200
H	2.05328900	0.60415000	-1.62661000
H	3.09250900	-0.58638700	-0.82612000
C	1.40888900	-1.41854600	-1.87891700
C	0.21130000	-1.07749200	-2.47792200
C	1.56768000	-2.73157000	-1.35241600
C	-0.87563700	-1.98899000	-2.53996400
H	0.07253600	-0.07514500	-2.87855500
C	0.53885000	-3.64391400	-1.40339700
H	2.51234200	-2.99460700	-0.88630300
C	-2.13529500	-1.63648900	-3.09487300
C	-0.71648600	-3.29664000	-1.97398400
H	0.66462100	-4.63799400	-0.98074800
C	-3.19368400	-2.51671000	-3.05265600
H	-2.26039700	-0.64778600	-3.52988900
C	-1.82460000	-4.18128700	-1.96023000
C	-3.05202300	-3.80362700	-2.46887200
H	-4.15874900	-2.22106000	-3.45669300
H	-1.71160000	-5.15522900	-1.49255200
C	6.10148700	-2.43140000	3.13521400
H	6.89726500	-1.83392500	3.59088800
H	6.16689000	-3.45035300	3.54351200
C	4.74497900	-1.82073300	3.42546900
C	4.53009200	-0.48654700	3.13633900
C	3.66845100	-2.59347500	3.93401700
C	3.26199500	0.11604200	3.31515500
H	5.34508300	0.12071900	2.74803000
C	2.41928200	-2.03745800	4.12193500
H	3.83488200	-3.64154800	4.17226300
C	3.03102700	1.48511200	3.00458800
C	2.17250800	-0.67499900	3.80654300
H	1.60211400	-2.64543800	4.50275600
C	1.77997400	2.02967200	3.15287400
H	3.85628800	2.08616900	2.63124200
C	0.88466200	-0.07852400	3.93442600
C	0.68079000	1.24226800	3.60333500
H	1.61501500	3.07676300	2.90976900
H	0.05263500	-0.68293200	4.28270700
C	-5.08237300	2.86100900	-3.36173500
H	-5.38510600	3.43452300	-4.24231900
H	-4.92797500	1.82501900	-3.66697000
O	-4.44770300	-5.23859400	-1.03376600
C	-4.24212400	-4.73543700	-2.36916800
H	-4.09311600	-5.63889200	-2.96847200
H	-5.14748200	-4.23773200	-2.73685000

Imaginary Frequency = 0

E(RB3LYP) = -4064.5336189 a.u.

Conformation #2 (Pop. = 20.28%)

Symbolic Z-matrix:

C	-6.57531800	-2.47382400	-0.12229800
C	-7.20312100	-1.92903900	-1.39453600
C	-6.28117300	-0.87372100	-2.02995600
C	-4.34046600	-2.01955400	-1.16166700
C	-5.20835700	-3.06896200	-0.43508500
O	-5.02548300	-1.47830400	-2.30487900
O	-7.48712800	-3.44142900	0.39667000
O	-8.48104800	-1.36226500	-1.12405100
O	-6.15239100	0.22317400	-1.15447500
O	-4.64187800	-3.47641600	0.80228200
C	-3.04690900	-2.61804200	-1.71547700
O	-2.29699200	-3.10713300	-0.60188300
C	-6.97461500	2.84635600	0.27404700
C	-8.21068900	2.12200000	-0.25535600
C	-6.31873100	1.58936900	-1.61865300
C	-7.83355600	1.92176100	-1.73031800
O	-5.87696600	2.39970600	-0.56019700
O	-8.64237000	1.02461500	-2.45081000
C	-5.48329000	1.92358500	-2.88205500
C	-6.62774400	2.66676300	1.74087000
O	-4.19768300	2.45299900	-2.62465100
H	-7.31012500	-2.74750500	-2.12031000
H	-7.92711000	2.89303000	-2.23111300
H	-8.32232800	1.14387000	0.22837600
H	-7.11480700	3.92355800	0.10592200
H	-6.44520000	-1.65208500	0.59488400
H	-5.34834300	-3.93408100	-1.10518600
H	-4.09833900	-1.21821600	-0.45221100
H	-3.28308800	-3.43998500	-2.40680100
H	-2.48184500	-1.85554600	-2.26585900
H	-6.00603500	2.71230300	-3.43058400
H	-5.68513000	3.19641400	1.93641100
H	-5.42118000	1.04638000	-3.54001900
C	3.58460100	3.46648200	2.48785000
C	4.03641000	2.31871600	3.14065900
C	4.38391700	4.01711700	1.46490200
C	5.25025300	1.69181100	2.80641600
H	3.43759400	1.88917100	3.93578400
C	5.60154300	3.42097800	1.16177700
C	6.05660500	2.25736700	1.80398300
H	6.22697000	3.88876100	0.40637100
C	3.15713700	-2.42855800	2.98176800
C	3.88716000	-3.30718200	2.15202200
C	3.76080500	-1.24954900	3.40541600
C	5.15916300	-2.92311200	1.72882700
C	5.05372700	-0.87006200	3.01049200
H	3.18790600	-0.61607800	4.07710700
C	5.75358700	-1.71417600	2.13001900
H	5.72676500	-3.58801600	1.08749600
C	6.97048600	-0.77280900	-2.19094100
C	7.19045000	0.61947700	-2.28896500
C	6.96342900	-1.36613400	-0.93517100
C	7.33453600	1.35746100	-1.11598400

C	7.13222500	-0.63355900	0.25304000
H	6.83108600	-2.44374900	-0.89651600
C	7.29349500	0.75850500	0.15634300
O	7.26540000	1.13820300	-3.55073300
O	3.29327400	-4.49928800	1.84366700
C	7.48444400	2.53951400	-3.69197500
H	7.50830400	2.72922800	-4.76593900
H	6.67262400	3.11965500	-3.23640700
H	8.44018100	2.84055100	-3.24658900
C	3.96012600	-5.37204700	0.93568300
H	4.11831200	-4.89084400	-0.03699200
H	4.92347600	-5.70834500	1.33761000
H	3.30028600	-6.23218100	0.81319800
C	5.62440700	0.42856300	3.57651100
H	5.24844500	0.53724400	4.60014700
H	6.71015500	0.35069900	3.66899600
C	7.13809500	-1.39754600	1.57548200
H	7.66036300	-2.34680900	1.41014900
H	7.73199100	-0.85994400	2.31810900
H	7.50177900	2.42686300	-1.17721600
C	7.40049200	1.68184300	1.36539100
H	8.05709600	2.51811900	1.09992300
H	7.89013600	1.17295500	2.19833600
H	-6.67314900	-0.56892100	-3.00188900
H	-3.68856600	-3.61192800	0.63947700
H	-8.91992000	-1.94440600	-0.47895200
H	-8.73750300	0.20216000	-1.92272400
H	-7.13014900	-3.73718500	1.25009700
O	2.42356900	4.12170800	2.77670200
C	1.58421000	3.58109400	3.79263800
H	1.24861900	2.56795600	3.53885200
H	2.09166600	3.56169200	4.76488200
H	0.72085800	4.24579800	3.84896300
O	-6.52149200	1.29132500	2.06696900
H	-7.41261300	3.15428000	2.34183900
O	6.86909600	-1.56495000	-3.31441300
O	1.90084900	-2.71508500	3.46212600
C	0.90668100	-3.16362600	2.50868600
H	0.84845900	-4.25517500	2.53992800
H	1.21940100	-2.86854200	1.50253100
O	-9.36068200	2.92864600	-0.05139600
H	-10.11382700	2.44790500	-0.43223200
C	-1.20169600	-3.96076500	-0.92737400
H	-1.52991800	-4.75172500	-1.61681300
H	-0.93953300	-4.44043400	0.02239800
C	0.00992100	-3.24088700	-1.49103700
C	0.93919500	-3.93990500	-2.23578500
C	0.24184900	-1.86947200	-1.19983200
C	2.12999700	-3.32266000	-2.70117700
H	0.77330800	-4.99038800	-2.46817700
C	1.37232800	-1.23300100	-1.66288700
H	-0.48966000	-1.32571300	-0.61040700
C	3.11604800	-4.03114100	-3.44349100
C	2.34764200	-1.93447000	-2.42176600
H	1.53283700	-0.17975900	-1.44728200
C	4.25091500	-3.39581100	-3.89085900
H	2.95562000	-5.08515800	-3.65702100
C	3.52334200	-1.30751300	-2.91642200

C	4.46555100	-2.01105000	-3.64083400
H	4.99436900	-3.95008200	-4.45875700
H	3.66918500	-0.24770000	-2.71853500
C	-3.23000200	1.51827300	-2.13627600
H	-2.98509800	0.78329700	-2.91691000
H	-3.63797700	0.97583200	-1.27564700
C	-2.00729200	2.29391100	-1.71657100
C	-0.79268000	2.14132600	-2.34964700
C	-2.11756400	3.20911200	-0.63017300
C	0.35690100	2.86889100	-1.93035700
H	-0.69701500	1.45145500	-3.18552800
C	-1.02770500	3.92432600	-0.19639100
H	-3.08447600	3.33005900	-0.15047700
C	1.62041000	2.73188600	-2.56449100
C	0.24105200	3.77507700	-0.82637400
H	-1.11977100	4.61731300	0.63659200
C	2.71366700	3.44587300	-2.12170000
H	1.71683100	2.04771600	-3.40412000
C	1.38495900	4.49482400	-0.39893000
C	2.60699300	4.34109300	-1.02530700
H	3.67505600	3.32178400	-2.61466800
H	1.29799900	5.16121400	0.45404600
C	-5.93850300	1.01752400	3.35057500
H	-6.58862400	0.29251400	3.85261100
H	-5.92876500	1.93134900	3.95906600
C	-4.54349600	0.44585600	3.21264400
C	-4.29837500	-0.88463900	3.48130500
C	-3.46715700	1.26659200	2.76391800
C	-3.00766600	-1.45641200	3.30579600
H	-5.10732100	-1.52670300	3.82355500
C	-2.20848900	0.74832200	2.57752200
H	-3.64464100	2.32046300	2.56930600
C	-2.74295200	-2.82828100	3.55642900
C	-1.94004600	-0.62651500	2.83243900
H	-1.40143600	1.38497200	2.22343800
C	-1.49124500	-3.35775400	3.32646400
H	-3.54798000	-3.46098600	3.92110500
C	-0.66497200	-1.20414900	2.60870900
C	-0.43362600	-2.54900400	2.83344900
H	-1.30597300	-4.41277300	3.51296100
H	0.13647600	-0.57171400	2.23247700
C	5.71079100	-1.33656400	-4.16223100
H	6.00019100	-1.75914100	-5.12834000
H	5.55478700	-0.26289700	-4.28637000
O	3.96897800	5.18487500	0.85523000
C	3.81498400	5.13891900	-0.57414200
H	3.71546000	6.18997500	-0.86453700
H	4.72383600	4.75068500	-1.04854300

Imaginary Frequency = 0

E(RB3LYP) = -4064.5324641 a.u.

***M*-5a**

Conformation #1 (Pop. = 81.87%)

Symbolic Z-matrix:

C	-5.80016800	-0.71927100	3.10539100
C	-6.86809800	-1.52981300	2.38272100
C	-6.42566900	-1.82120200	0.94043400
C	-4.11168000	-1.81589900	1.59341900
C	-4.46098100	-1.45032300	3.04999700
O	-5.19618600	-2.51798400	0.96911900
O	-6.26107600	-0.53720000	4.44345000
O	-6.34247200	-0.58743500	0.25625900
C	-2.92890400	-2.77726700	1.49683700
O	-1.79427300	-2.16753000	2.11023900
C	-7.42087800	1.81154100	-1.50694600
C	-8.57397700	1.17992300	-0.73347800
C	-6.84792800	-0.44765300	-1.08363900
O	-6.38044700	0.80581400	-1.53225000
C	-6.33043800	-1.52750200	-2.03393300
O	-4.91984300	-1.56355600	-1.98220700
H	-6.98820900	-2.49541900	2.89382600
H	-8.42902500	1.32204800	0.34537500
H	-7.75614300	1.99295300	-2.53843800
H	-5.69366800	0.25353300	2.60619300
H	-4.55445800	-2.38203900	3.63268600
H	-3.88748900	-0.89682700	1.03841400
H	-3.18253400	-3.71608800	2.01207000
H	-2.72080100	-3.00695500	0.44362500
H	-6.68570300	-1.27423000	-3.04435400
H	-6.75682500	-2.50833600	-1.77054600
C	4.97649600	-3.41454300	-1.45188500
C	5.13722200	-2.40565100	-2.39325900
C	5.80674100	-3.42348100	-0.30960300
C	6.05895200	-1.35711800	-2.23292400
H	4.51273900	-2.45381500	-3.28084300
C	6.70790300	-2.37558900	-0.13078000
C	6.83550900	-1.32877600	-1.06197600
H	7.34762200	-2.36837200	0.74465500
C	2.77679100	1.45275400	-3.60871000
C	3.00315900	2.66765000	-2.91907200
C	3.82976500	0.55629800	-3.74590300
C	4.27747300	2.94405900	-2.43997500
C	5.10670500	0.80627100	-3.20457100
H	3.66600500	-0.37742600	-4.27161000
C	5.34056800	2.02564300	-2.55498900
H	4.46345700	3.89963800	-1.96071800
C	6.22350900	2.95537800	1.81699900
C	6.62532300	1.71405000	2.35644400
C	6.27760300	3.13111800	0.43444200
C	7.12829200	0.74447500	1.49579900
C	6.74699200	2.13191100	-0.43629100
H	5.96382500	4.07759900	0.00911400
C	7.21491100	0.92280200	0.10598100
O	6.65234100	1.43635600	3.70450300
O	1.92062500	3.49430900	-2.79587900

C	1.90294700	4.46718900	-1.74229200
H	2.54295500	4.12489600	-0.91905500
H	2.30593700	5.42075000	-2.10602900
C	6.14576200	-0.30220400	-3.33355800
H	5.98975500	-0.80549300	-4.29471300
H	7.15345600	0.11639400	-3.37901500
C	6.67948200	2.41626800	-1.93316700
H	6.82532900	3.49195700	-2.08524200
H	7.50320400	1.92744400	-2.45780200
H	7.46905700	-0.18298200	1.94752900
C	7.81448100	-0.20890900	-0.72702600
H	8.64209200	-0.64241400	-0.15386700
H	8.26412200	0.19161000	-1.63826600
H	-7.12895600	-2.49920500	0.45555200
H	-5.60396800	0.01867200	4.89375200
O	4.07414200	-4.42097200	-1.70642000
O	-6.57034600	2.99877400	0.41062400
O	-9.80567500	1.73618100	-1.16340200
H	-10.50716400	1.29965800	-0.65286500
C	3.06497700	-4.67528800	-0.69399800
H	3.19208100	-5.70885100	-0.35859000
H	3.23319000	-4.01880200	0.16218100
C	5.46290800	1.66122700	4.50362300
H	5.28361200	2.73309600	4.60398700
H	5.74620800	1.25905000	5.48001100
O	5.66776100	-4.48900000	0.53492500
O	5.82103900	3.91672600	2.70323500
O	1.51175700	1.25994400	-4.08571100
C	1.25496300	0.07205800	-4.82888400
H	1.41063000	-0.82594100	-4.21891000
H	0.20665200	0.12806800	-5.12594500
H	1.88647000	0.01700500	-5.72416900
C	6.45173600	-4.51142700	1.72466500
H	6.23640400	-3.64456300	2.36098700
H	6.17081900	-5.42635000	2.24830700
H	7.52407000	-4.53753600	1.49642500
C	5.41858000	5.18432600	2.18951900
H	4.54955400	5.09011500	1.52744300
H	5.14908700	5.78439600	3.05967300
H	6.23774400	5.67255500	1.64839400
C	-6.87247500	3.12158200	-0.97090000
H	-7.62408700	3.91081400	-1.13139900
H	-5.97588600	3.38742000	-1.54635100
C	-0.67801000	-3.04780200	2.19136500
H	-0.91391000	-3.89156700	2.85821300
H	-0.47975300	-3.47206200	1.19456500
C	-5.70817100	4.01731500	0.90547000
H	-5.72619200	3.89200800	1.99460100
H	-6.11276600	5.01617200	0.68212700
C	0.54732700	-2.31805500	2.68872200
C	0.67718200	-0.94849100	2.59861700
C	1.61709500	-3.08559100	3.23067800
C	1.85609900	-0.29019900	3.04705200
H	-0.13221600	-0.35434700	2.18502500
C	2.77291100	-2.48099300	3.66512200
H	1.51054000	-4.16523900	3.30609200
C	2.00974300	1.11994500	2.97625700
C	2.92905100	-1.06846600	3.59238600

H	3.57987900	-3.07809800	4.08280100
C	3.16032500	1.72770700	3.42860800
H	1.20249000	1.71603800	2.55761200
C	4.09654700	-0.40851900	4.05507400
C	4.22279900	0.96658300	3.98597000
H	3.26479400	2.80757500	3.36781100
H	4.90043300	-1.00663400	4.47994300
C	-4.28465700	3.90213100	0.38917500
C	-3.51924300	5.02410400	0.14860100
C	-3.72195800	2.61230000	0.17319400
C	-2.17471600	4.91981300	-0.30388900
H	-3.94239200	6.01583300	0.29882300
C	-2.43017600	2.47630600	-0.27491900
H	-4.34518100	1.73761400	0.32886100
C	-1.36297800	6.05599200	-0.56521700
C	-1.61992500	3.61784300	-0.52880000
H	-2.01098700	1.48753400	-0.44728600
C	-0.07358700	5.91262400	-1.03202200
H	-1.77776500	7.04770500	-0.40173000
C	-0.29154800	3.50690200	-1.00743200
C	0.47793600	4.62562300	-1.26105200
H	0.52967500	6.79403800	-1.23590100
H	0.11653200	2.51744900	-1.19528900
C	-2.89198100	-2.65250300	-2.59831900
C	-2.35128400	-3.91836800	-2.51697400
C	-2.06353200	-1.52432000	-2.33412000
C	-0.99315400	-4.12345400	-2.14818500
H	-2.97256900	-4.78958400	-2.71362300
C	-0.74855700	-1.68630500	-1.96573100
H	-2.49191900	-0.52891500	-2.40426200
C	-0.42832200	-5.42036100	-2.02434700
C	-0.17660100	-2.98540000	-1.84462200
H	-0.12899800	-0.81865500	-1.75187500
C	0.86763000	-5.58487500	-1.58747700
H	-1.04142000	-6.28599900	-2.26287800
C	1.15298800	-3.19320600	-1.39388000
C	1.67445100	-4.46663000	-1.24758800
H	1.28039000	-6.58513800	-1.48114500
H	1.75653100	-2.32586400	-1.13463900
C	-4.34878100	-2.45247100	-2.93951300
H	-4.46728900	-2.02816200	-3.95089300
H	-4.87201900	-3.41999900	-2.92820900
O	-9.10964400	-1.19065300	-0.23259600
H	-8.90801500	-0.96507100	0.70129100
O	-3.48780400	-0.58997600	3.63014300
H	-2.62452300	-1.01918600	3.47352000
O	-8.11441200	-0.83949600	2.35533600
H	-8.28207700	-0.50854300	3.25430400
C	-8.39747400	-0.30670500	-1.06733400
H	-8.77223400	-0.47382600	-2.08459400

Imaginary Frequency = 0

E(RB3LYP) = -4064.5398342 a.u.

Conformation #2 (Pop. = 12.04%)

Symbolic Z-matrix:

C	-5.79521100	-0.67942100	3.06253500
C	-6.84753000	-1.48341600	2.31054600
C	-6.39366400	-1.73257600	0.86277600
C	-4.08482100	-1.72349800	1.54199700
C	-4.44988200	-1.39699700	3.00392500
O	-5.15695700	-2.41790600	0.88694800
O	-6.27423400	-0.53391600	4.39918600
O	-6.31213400	-0.48570600	0.20493300
C	-2.89576500	-2.67547100	1.43504200
O	-1.77080400	-2.08148300	2.08135500
C	-7.36411200	2.00044900	-1.36809700
C	-8.52265700	1.34182300	-0.62239500
C	-6.84710400	-0.29604600	-1.11807000
O	-6.36942000	0.96132700	-1.54016600
C	-6.38751500	-1.35919700	-2.11477800
O	-4.97855600	-1.45851900	-2.10517600
H	-6.95788900	-2.46413200	2.79441300
H	-8.36327500	1.40487100	0.46146000
H	-7.72193800	2.30150100	-2.36283100
H	-5.69295000	0.30619800	2.58863200
H	-4.54074500	-2.34325900	3.56320500
H	-3.86107400	-0.78795700	1.01477200
H	-3.15393900	-3.62939100	1.91959200
H	-2.67117300	-2.87570300	0.37964800
H	-6.75884100	-1.06015900	-3.10698900
H	-6.84919000	-2.32938600	-1.87133900
C	4.75469700	-3.55627000	-1.41835500
C	4.94268000	-2.56876800	-2.37680100
C	5.59714300	-3.57976200	-0.28580900
C	5.90420100	-1.55427600	-2.24321500
H	4.30787000	-2.60794200	-3.25742700
C	6.54383500	-2.56656500	-0.13682000
C	6.70139600	-1.54043300	-1.08607000
H	7.19668200	-2.57356900	0.72891300
C	2.71902600	1.41178600	-3.48157900
C	3.05239300	2.64943100	-2.89676600
C	3.70866100	0.45138800	-3.63871200
C	4.36969300	2.87208200	-2.50059000
C	5.02777100	0.64628500	-3.19662800
H	3.42149000	-0.49013100	-4.09842500
C	5.36201300	1.88200300	-2.61530500
H	4.63730800	3.83703300	-2.08159300
C	6.39319500	2.85500400	1.69214100
C	6.72368300	1.60289800	2.25465900
C	6.43646300	2.99606300	0.30552600
C	7.14796700	0.58320600	1.41041800
C	6.82656000	1.94868800	-0.54796900
H	6.17567000	3.95052200	-0.13720500
C	7.22293900	0.72361600	0.01527800
O	6.75274300	1.36001700	3.60938600
O	2.04141500	3.56185900	-2.77272700
C	2.05447600	4.48697400	-1.67687600
H	2.74958100	4.13118100	-0.90604500

H	2.41376200	5.46339400	-2.02448000
C	6.00427600	-0.51419900	-3.35434400
H	5.78440000	-1.01411400	-4.30455000
H	7.02886600	-0.14706800	-3.44338300
C	6.74409700	2.20291400	-2.05036000
H	6.95358600	3.26264900	-2.23420400
H	7.51868100	1.64744400	-2.58296800
H	7.43520900	-0.35412300	1.87859700
C	7.74065100	-0.46274600	-0.79641400
H	8.55543200	-0.92413700	-0.22663400
H	8.19335700	-0.11222100	-1.72669800
H	-7.08658900	-2.40661900	0.35739200
H	-5.63403000	0.02590800	4.86839700
O	3.81122200	-4.52824700	-1.65794900
O	-6.38256400	2.93312000	0.62455200
O	-9.74469400	1.95868400	-0.99650500
H	-10.45059200	1.51154000	-0.50154100
C	2.81320400	-4.76202100	-0.63788600
H	2.93100500	-5.79031400	-0.28226800
H	2.99307000	-4.09523200	0.20963900
C	5.57214100	1.64632700	4.40376400
H	5.42556500	2.72607200	4.46798300
H	5.84495900	1.26808900	5.39246300
O	5.42675400	-4.62468800	0.57842700
O	6.06479900	3.85783900	2.56230800
O	1.41990100	1.10463200	-3.82800600
C	0.90996600	1.78889700	-4.98142100
H	1.51983200	1.56296600	-5.86499000
H	-0.10386800	1.41323600	-5.13439400
H	0.88142600	2.87025000	-4.81929500
C	6.22438100	-4.65928900	1.75886800
H	6.05327100	-3.77341300	2.38224100
H	5.91240500	-5.55257700	2.30189500
H	7.29166600	-4.73428300	1.51812700
C	5.73803200	5.13813000	2.02658000
H	4.85409200	5.08732700	1.37964100
H	5.52242900	5.77289900	2.88706200
H	6.57866800	5.55970700	1.46281900
C	-6.74474000	3.22456700	-0.71667100
H	-7.47532600	4.04874500	-0.74822300
H	-5.86759200	3.52859000	-1.30275100
C	-0.66094900	-2.97134200	2.16302500
H	-0.90934300	-3.81967500	2.81916800
H	-0.45916900	-3.38761300	1.16354500
C	-5.46547600	3.85901600	1.19411600
H	-5.43487000	3.60024900	2.25922200
H	-5.84880700	4.88809200	1.11754100
C	0.56591100	-2.25614200	2.67663700
C	0.74294500	-0.89729200	2.52208400
C	1.59052000	-3.02688700	3.29482900
C	1.92866700	-0.25418300	2.97313300
H	-0.03293000	-0.30095900	2.05109600
C	2.75146800	-2.43689200	3.73624400
H	1.44547700	-4.09703300	3.42255000
C	2.13422700	1.14408500	2.82929500
C	2.95760500	-1.03671300	3.59266700
H	3.52420300	-3.03606600	4.21168500
C	3.29388800	1.73616400	3.27879900

H	1.36085800	1.74262000	2.35400900
C	4.13545700	-0.39201500	4.05074900
C	4.31407100	0.97105800	3.90612900
H	3.43885000	2.80669900	3.16098300
H	4.90648000	-0.99262700	4.52960300
C	-4.07082400	3.77665200	0.59622400
C	-3.25210300	4.88630100	0.55069500
C	-3.59025700	2.53313400	0.09862100
C	-1.93430200	4.81180700	0.02259000
H	-3.61194000	5.84589900	0.91828800
C	-2.32793900	2.43183800	-0.43687100
H	-4.25454300	1.67517100	0.10915500
C	-1.06806300	5.93700500	-0.03868000
C	-1.46487900	3.56056500	-0.49415000
H	-1.97550000	1.48171000	-0.83205000
C	0.19067400	5.83165500	-0.58887000
H	-1.41792000	6.89179600	0.34650000
C	-0.16589900	3.48719500	-1.05746500
C	0.65557300	4.59532700	-1.10997600
H	0.83573500	6.70596200	-0.63562900
H	0.17303100	2.54496700	-1.47856300
C	-3.03554100	-2.66335100	-2.78778400
C	-2.62484000	-3.90287900	-2.34532600
C	-2.07145600	-1.62482300	-2.93381500
C	-1.26959900	-4.15534700	-1.99677700
H	-3.34854800	-4.70743300	-2.23264700
C	-0.75012500	-1.83354500	-2.61305500
H	-2.39490500	-0.65323100	-3.29810100
C	-0.83966700	-5.41194100	-1.49640700
C	-0.31160800	-3.09533500	-2.11508600
H	-0.02433800	-1.03228400	-2.73421800
C	0.46583100	-5.60139200	-1.09918800
H	-1.56031600	-6.22236600	-1.41894500
C	1.02476800	-3.32752400	-1.69836800
C	1.41597800	-4.54966700	-1.17963700
H	0.77768600	-6.56634800	-0.70705400
H	1.74176500	-2.51285600	-1.77018200
C	-4.49131500	-2.39233800	-3.07229100
H	-4.63028300	-1.97842500	-4.08428800
H	-5.06489900	-3.32905500	-3.01618300
O	-9.10767600	-1.04819000	-0.27776000
H	-8.90103000	-0.87859500	0.66689100
O	-3.48864200	-0.54314200	3.61239400
H	-2.62021200	-0.95947400	3.44850500
O	-8.10206500	-0.80792600	2.30169700
H	-8.26248000	-0.48581100	3.20544400
C	-8.39055900	-0.12229200	-1.06045500
H	-8.78435700	-0.20862900	-2.08070000

Imaginary Frequency = 0

E(RB3LYP) = -4064.5380266 a.u.

***P*-5b**

Conformation #1 (Pop. = 75.32%)

Symbolic Z-matrix:

C	-5.74916800	4.67505000	-1.18282900
C	-5.74829200	4.39628600	0.31511100
C	-4.69552300	3.33385500	0.65252800
C	-3.34115300	3.98482800	-1.22653300
C	-4.34621300	5.07931300	-1.63043600
O	-3.42680700	3.74490600	0.19491800
O	-6.70400100	5.70577700	-1.41050700
O	-7.01343000	3.94676800	0.78279500
O	-5.12283000	2.11066700	0.06935000
O	-4.39509700	5.24859900	-3.04762700
C	-1.89380300	4.32524900	-1.53925600
O	-1.58506900	5.60583700	-1.00634000
C	-5.86001400	-1.21335500	0.51959700
C	-5.86225500	-0.85441600	2.02426400
C	-4.78213100	0.88561900	0.72722600
C	-5.65849500	0.67123400	1.99890800
O	-5.17436100	-0.13518000	-0.16817100
O	-6.94016600	1.25703500	1.85334400
O	-4.88065700	-1.54667800	2.77432100
C	-3.24948800	0.83058800	0.91966000
O	-2.78650900	-0.45646600	1.33694500
O	-7.99860900	-2.44097500	0.43561100
H	-5.46158800	5.31865200	0.84010600
H	-5.15693600	1.01970000	2.91270900
H	-6.82491600	-1.08808900	2.48377200
H	-5.28116900	-2.12671500	0.37028500
H	-6.04268600	3.76105200	-1.71990900
H	-4.07267900	6.01887400	-1.13722700
H	-3.59120900	3.06240500	-1.76683300
H	-1.24627800	3.55028000	-1.10806000
H	-1.75043200	4.32004000	-2.63052200
H	-2.92372400	1.55160200	1.67436300
H	-2.78811700	1.10436300	-0.03181100
C	5.20040600	2.55837300	3.91397700
H	6.24063200	2.43710600	3.59389700
H	5.20443600	3.22032200	4.78472400
C	1.57382400	-4.04251200	-3.96615800
H	1.94918100	-3.02659600	-4.13548700
H	0.66816200	-4.19826300	-4.55408500
O	4.66603300	1.32988000	4.43296600
O	1.18949000	-4.22802300	-2.60537900
C	2.14565300	-4.08416800	-1.64076700
C	3.48563100	-3.79115400	-1.88601100
C	1.72469000	-4.24830100	-0.30392400
C	4.41818400	-3.62506100	-0.84619400
H	3.82609400	-3.67237800	-2.90818200
C	2.65515300	-4.12425700	0.71947600
C	4.00367000	-3.80372400	0.48464800
H	2.29938900	-4.27303700	1.73542800
C	5.68876300	0.28298000	-2.74794000
C	6.31958600	1.06948200	-1.76699600

C	5.59050100	-1.09014300	-2.54180100
C	6.74902800	0.46138900	-0.59110400
C	6.02693400	-1.71149300	-1.36237500
H	5.12938300	-1.67885400	-3.32938600
C	6.58431900	-0.91040700	-0.34648100
H	7.22372800	1.09395100	0.15381500
C	4.78200600	0.17867800	3.69421900
C	3.85297000	-0.84421600	3.99686500
C	5.76499600	-0.07056400	2.74443400
C	3.95544200	-2.06354200	3.33412100
C	5.86517300	-1.29898000	2.06270500
H	6.49539700	0.69923800	2.52305200
C	4.94131200	-2.31140500	2.35927300
O	2.92271700	-0.54550000	4.95061500
O	6.47601900	2.43458100	-1.90476800
O	0.39628200	-4.44351400	0.00409500
C	1.95701900	-1.54055200	5.27433300
H	2.42995600	-2.44148600	5.68454800
H	1.31213600	-1.09369100	6.03268400
H	1.35503000	-1.81469700	4.39916100
C	7.40557700	2.83376400	-2.92480800
H	8.40717600	2.44540400	-2.70383600
H	7.08611500	2.48456500	-3.91140100
H	7.42576300	3.92524000	-2.90829600
C	5.83529500	-3.22187900	-1.24006000
H	6.05878600	-3.66926300	-2.21504600
H	6.56404500	-3.64387000	-0.54515600
C	6.97983300	-1.43967400	1.02733200
H	7.84572300	-0.86734500	1.37893600
H	7.31486000	-2.47708100	0.96316300
H	3.25606300	-2.85660500	3.57341700
C	4.93610700	-3.68197100	1.68748700
H	4.61603700	-4.41921100	2.43285100
H	5.95193400	-3.97019600	1.40705800
H	2.33459100	-4.77120600	-4.27048600
H	-4.59283100	3.23415300	1.73738600
H	-3.72796400	5.90753700	-3.29497800
H	-7.69159200	4.46424700	0.31523900
H	-6.88107200	2.08232600	1.32953900
H	-4.01120200	-1.31300000	2.38527000
H	-6.67651600	5.89772500	-2.36316200
C	4.09343500	1.75550100	-3.76259100
H	4.42103400	2.58432800	-3.12980500
H	3.92297300	2.12825700	-4.77540800
O	5.19503900	0.81423500	-3.92149800
C	-0.20147700	5.93348900	-1.05095200
H	0.20861300	5.75690000	-2.05665300
H	-0.16484100	7.01418700	-0.86816100
C	-2.19009500	-1.26360500	0.30089000
H	-2.91782500	-1.45354600	-0.49532800
H	-1.96640800	-2.21459400	0.79537700
C	0.63519300	5.21014800	-0.00877100
C	1.96296400	4.91981900	-0.24518100
C	0.06515800	4.88316700	1.25347300
C	2.78088600	4.32424300	0.75447400
H	2.40969200	5.15461800	-1.20969200
C	0.82232700	4.29058700	2.23620900
H	-0.98721000	5.09113000	1.41852400

C	4.15602400	4.03510700	0.54469900
C	2.19922000	3.99973100	2.02423100
H	0.37596600	4.03887800	3.19532600
C	4.91843900	3.46650400	1.54282600
H	4.60583400	4.26093000	-0.41876600
C	3.01274600	3.41041300	3.02519700
C	4.35262600	3.14928200	2.80518700
H	5.96999400	3.25929600	1.36229500
H	2.56516600	3.16419100	3.98547000
C	-0.92940500	-0.64887000	-0.26873400
C	-0.68571200	-0.67028000	-1.62720700
C	0.02481600	-0.05067100	0.60097400
C	0.49828500	-0.10564400	-2.17558600
H	-1.40882300	-1.12333100	-2.30230800
C	1.16892900	0.52538800	0.10187800
H	-0.17258300	-0.03738700	1.66887600
C	0.77625300	-0.11816000	-3.57059700
C	1.43646800	0.52300100	-1.29558800
H	1.88624100	0.99047000	0.77305600
C	1.91974900	0.46683900	-4.06753000
H	0.06529800	-0.59144700	-4.24343300
C	2.59371700	1.13403100	-1.84519200
C	2.84526000	1.11563000	-3.20427600
H	2.11585600	0.45072200	-5.13699400
H	3.28751000	1.62969500	-1.17058800
C	-0.17703600	-5.73382200	-0.33427500
H	-0.01384000	-6.41989900	0.50599800
H	0.32063700	-6.13639400	-1.22039600
C	-6.57714800	-4.35227000	-0.16319000
C	-5.68705200	-4.36534700	-1.21759600
C	-6.16831900	-4.87327000	1.09652300
C	-4.34897300	-4.81305500	-1.05076600
H	-5.98812100	-3.97607200	-2.18821300
C	-4.88780800	-5.33606800	1.28770200
H	-6.87734600	-4.87844700	1.92053200
C	-3.39011000	-4.74186400	-2.09829600
C	-3.93192100	-5.28779100	0.23468400
H	-4.58026400	-5.71794100	2.25816900
C	-2.07432900	-5.06964300	-1.86974800
H	-3.70839600	-4.39524600	-3.07854900
C	-2.56978400	-5.64211000	0.42799400
C	-1.64492800	-5.51510600	-0.58885700
H	-1.33887200	-4.97451300	-2.66261400
H	-2.25362800	-5.98490700	1.41113200
C	-7.25227400	-1.35696800	-0.11353700
H	-7.14561200	-1.46539200	-1.20175800
H	-7.82184000	-0.44498700	0.08782100
C	-7.92538200	-3.67904500	-0.28676200
H	-8.71382600	-4.29875500	0.15256300
H	-8.17112900	-3.50874600	-1.34454500

Imaginary Frequency = 0

E(RB3LYP) = -4064.5350289 a.u

Conformation #2 (Pop. = 21.14%)

Symbolic Z-matrix:

C	-5.87160200	4.53565500	-1.29345700
C	-5.91307200	4.25748200	0.20403600
C	-4.84564000	3.22310900	0.57976200
C	-3.44959500	3.90043000	-1.26335500
C	-4.46490600	4.97316900	-1.69770400
O	-3.57412100	3.65814800	0.15450100
O	-6.84275700	5.54288000	-1.55358600
O	-7.18021800	3.77535700	0.63116900
O	-5.22776200	1.98317300	0.00074500
O	-4.47594200	5.14054800	-3.11598400
C	-1.99855500	4.26716300	-1.52646700
O	-1.71727900	5.53116800	-0.94083600
C	-5.86437700	-1.35765200	0.53958900
C	-5.87709400	-0.95475400	2.03357500
C	-4.85999000	0.77994500	0.68369100
C	-5.72550600	0.57535400	1.96284800
O	-5.23804800	-0.26811200	-0.18598500
O	-7.02601200	1.11464800	1.80017100
O	-4.87273500	-1.59130800	2.80236400
C	-3.32526000	0.76273500	0.87049000
O	-2.82888300	-0.51075500	1.29008900
O	-7.94158800	-2.68457700	0.52639600
H	-5.66703100	5.18768600	0.73603600
H	-5.23632500	0.96757200	2.86542000
H	-6.83102900	-1.20660100	2.50145900
H	-5.24236300	-2.24575200	0.41391200
H	-6.12512000	3.61450900	-1.83892000
H	-4.22791100	5.91893200	-1.19707000
H	-3.66417800	2.97414200	-1.81257300
H	-1.35547200	3.48440800	-1.10367200
H	-1.82538600	4.29965700	-2.61286100
H	-3.01400500	1.49639700	1.61918200
H	-2.87537500	1.04287100	-0.08448500
C	4.43786300	2.23803800	4.60332000
H	4.90263300	3.03179400	5.19892400
H	3.72155500	1.71168700	5.23712300
C	1.45926300	-3.80385100	-4.03985000
H	1.81335200	-2.78245500	-4.22151900
H	0.51821700	-3.96152500	-4.56876200
O	5.55274800	1.38440500	4.28400500
O	1.16712700	-4.00898700	-2.65904000
C	2.18356700	-3.86927400	-1.75861200
C	3.50163000	-3.55986200	-2.08769000
C	1.85432600	-4.06101700	-0.40017100
C	4.50171300	-3.41248900	-1.11019600
H	3.77267000	-3.42058300	-3.12785500
C	2.84946900	-3.94670800	0.56190300
C	4.17837200	-3.61654500	0.24268000
H	2.56493500	-4.11954400	1.59649900
C	5.62522000	0.56290300	-2.92906300
C	6.33124700	1.30777700	-1.96657900
C	5.54333100	-0.81816300	-2.77833100
C	6.86289600	0.64885700	-0.86247700

C	6.07983500	-1.49017800	-1.66968900
H	5.01614100	-1.37228100	-3.54939100
C	6.72669400	-0.73492800	-0.67291500
H	7.39150000	1.25023300	-0.12802900
C	5.36029200	0.22345400	3.56635600
C	4.42367300	-0.77677100	3.90716300
C	6.24345400	-0.01950500	2.52049000
C	4.40518700	-1.95546100	3.15887600
C	6.23968300	-1.20547300	1.77135500
H	6.97401500	0.75515600	2.30510000
C	5.29233500	-2.19252500	2.09416600
O	3.61924500	-0.53518800	4.98590000
O	6.46555700	2.67901400	-2.05162600
O	0.54999400	-4.29181500	-0.01937300
C	2.64963500	-1.51759600	5.34220700
H	3.12600500	-2.46378100	5.62557800
H	2.11615300	-1.10881400	6.20139700
H	1.94260800	-1.69641200	4.52348100
C	7.31359200	3.13607000	-3.11701000
H	8.33295800	2.75259400	-2.98722900
H	6.92671800	2.82709500	-4.09283500
H	7.32344400	4.22599300	-3.05236800
C	5.88904200	-3.00319400	-1.59321100
H	6.03509500	-3.41396200	-2.59846800
H	6.66348100	-3.45615000	-0.97112100
C	7.24749400	-1.32680200	0.63185200
H	8.15390000	-0.78234300	0.91901800
H	7.55397800	-2.36594600	0.49490400
H	3.70189300	-2.73659600	3.42476700
C	5.19551300	-3.53822400	1.37890800
H	4.91461700	-4.29042200	2.12500800
H	6.18185900	-3.83900000	1.01726200
H	2.20488900	-4.52146500	-4.40242000
H	-4.77038600	3.13668600	1.66801600
H	-3.82196700	5.81864600	-3.34565300
H	-7.85658400	4.28423500	0.15188600
H	-6.99373000	1.91714300	1.24038500
H	-4.01339200	-1.36282000	2.38874900
H	-6.78624500	5.73697300	-2.50457000
C	3.94624200	2.06236200	-3.74502500
H	4.29530500	2.83342500	-3.05332300
H	3.73028400	2.52470500	-4.71131400
O	5.04365400	1.14882100	-4.03457400
C	-0.32983300	5.82646000	-0.83362500
H	0.17893100	5.66821700	-1.79646200
H	-0.28945900	6.90070100	-0.61686500
C	-2.20384200	-1.29747200	0.25429600
H	-2.92180300	-1.50341600	-0.54681600
H	-1.95586400	-2.24364100	0.74589800
C	0.37863500	5.05307300	0.26763000
C	1.73272700	4.79337600	0.19046400
C	-0.34355500	4.64069800	1.42052500
C	2.42342500	4.14232700	1.24835700
H	2.29690400	5.09588500	-0.69013100
C	0.29063300	3.98704600	2.45206900
H	-1.41158200	4.82764000	1.46009800
C	3.82302000	3.88498900	1.20855800
C	1.68650300	3.72401800	2.40330300

H	-0.27258300	3.66676800	3.32559700
C	4.46048600	3.26586500	2.26014300
H	4.39022600	4.19320300	0.33314600
C	2.37176400	3.05983000	3.45572900
C	3.73373500	2.84115800	3.40677600
H	5.53128500	3.09129700	2.22031900
H	1.80034800	2.73484800	4.32317800
C	-0.95936500	-0.64098600	-0.30303700
C	-0.73553900	-0.58797400	-1.66397100
C	-0.00216900	-0.07148900	0.58264700
C	0.42783500	0.02967200	-2.19921700
H	-1.46084000	-1.01792100	-2.35167700
C	1.12446600	0.54945300	0.09799300
H	-0.18343700	-0.11625800	1.65264900
C	0.68017100	0.10065200	-3.59736700
C	1.36861800	0.62856600	-1.30154500
H	1.84396900	0.99117300	0.78208000
C	1.80010100	0.73970000	-4.08038500
H	-0.03296700	-0.35028300	-4.28315700
C	2.50289200	1.29480300	-1.83598300
C	2.72717000	1.36038200	-3.19808300
H	1.97634800	0.78888300	-5.15227700
H	3.19958400	1.76594500	-1.14660700
C	0.02232400	-5.61824400	-0.28748600
H	0.23867200	-6.26096100	0.57475000
H	0.51520800	-6.03655000	-1.16962800
C	-6.43498300	-4.54588100	-0.02185500
C	-5.56806900	-4.56371100	-1.09521000
C	-5.97345500	-4.98756000	1.24996900
C	-4.20593200	-4.93644100	-0.93992200
H	-5.90900900	-4.23575000	-2.07525800
C	-4.66720200	-5.37645400	1.43168900
H	-6.66354000	-4.98999100	2.08987300
C	-3.27575400	-4.86925400	-2.01295700
C	-3.73724100	-5.32840400	0.35561700
H	-4.32012700	-5.69709300	2.41094100
C	-1.94057300	-5.12050100	-1.80160000
H	-3.63307000	-4.58759100	-3.00054300
C	-2.35455900	-5.60391600	0.53231200
C	-1.46012600	-5.47972200	-0.51199100
H	-1.22903400	-5.03028300	-2.61631600
H	-1.99993400	-5.88382700	1.52224200
C	-7.25253100	-1.59027900	-0.07513800
H	-7.15079900	-1.73775100	-1.15917500
H	-7.86253900	-0.69833300	0.09569900
C	-7.81795800	-3.94714300	-0.14493400
H	-8.56549000	-4.58613100	0.33629900
H	-8.09167800	-3.83523600	-1.20363800

Imaginary Frequency = 0

E(RB3LYP) = -4064.5338306 a.u

***M*-5b**

Conformation #1 (Pop. = 52.95%)

Symbolic Z-matrix:

C	7.28986400	-2.78527500	-2.16707300
C	6.36770200	-1.69230000	-2.72740900
C	6.46688500	-0.44477000	-1.84339600
C	6.92881700	-3.07787100	-0.69188400
O	6.06553100	-0.75992500	-0.51859300
O	8.62321500	-2.30392400	-2.31966000
O	5.03220200	-2.20045200	-2.72957200
O	5.58923900	0.51314600	-2.40594300
O	5.72002400	-3.82818900	-0.63322000
C	5.55318600	2.35399000	-0.69546400
O	4.15557800	2.22111700	-2.61994700
O	6.85089900	1.96081700	-0.30245800
O	5.86480400	4.81885700	-0.84082400
H	6.68391800	-1.42963400	-3.74574500
H	4.92399600	2.59234500	0.17089100
H	7.13452600	-3.70927400	-2.73981800
H	7.70891100	-3.71360000	-0.25402200
O	-0.86786500	-3.53945600	2.16725300
C	-6.36437400	2.74559900	1.01693800
C	-7.01704100	1.53558600	0.81524800
C	-6.81396300	0.41539000	1.63837800
H	-7.72599400	1.47898400	-0.00700000
C	-5.27151800	1.77029400	2.93141200
C	-5.90615200	0.53565000	2.70609900
H	-4.60052300	1.85639300	3.77868100
C	-6.87843000	-2.28916600	-2.11979800
C	-5.97436700	-3.33504100	-1.83057200
C	-7.36709700	-1.51532200	-1.06664300
C	-5.64313300	-3.57876100	-0.50157800
C	-7.01735100	-1.75638500	0.27323400
H	-8.06682100	-0.71514300	-1.27985000
C	-6.15056300	-2.82270700	0.56625500
H	-4.97561700	-4.41399700	-0.30850200
C	-1.99011900	-2.81357200	2.50749700
C	-1.90507900	-1.65428700	3.29884400
C	-3.24381600	-3.27385300	2.12045900
C	-3.07650900	-0.98401000	3.64151000
C	-4.42991000	-2.60720200	2.46304500
H	-3.28284000	-4.18916900	1.53623100
C	-4.34416400	-1.42714000	3.22907700
C	-7.62012100	-0.84604400	1.33808300
H	-8.61046300	-0.53148400	0.98976600
H	-7.79762600	-1.40749200	2.25838200
C	-5.74308700	-3.21606800	1.98250300
H	-5.63228700	-4.30588000	2.00724500
H	-6.54771400	-2.98589600	2.68424100
H	-2.98488200	-0.09527900	4.25999300
C	-5.56163600	-0.60944700	3.65570200
H	-5.35002800	-0.17974800	4.64089200
H	-6.42639100	-1.26172300	3.79642400
H	7.48588100	-0.04487100	-1.85035000

H	5.11569300	-3.37682900	-1.25974100
H	9.23012000	-3.02807700	-2.09723500
H	4.41686100	-1.46871300	-2.48804600
H	6.82266900	1.17604400	0.27929200
H	6.69449100	4.73653100	-0.34265200
C	-0.06427200	-2.94621100	1.13461100
H	-0.63473900	-2.86164400	0.20186300
H	0.30087400	-1.95975500	1.43596100
H	0.78231700	-3.61908000	0.98340600
O	-7.23344900	-2.11931400	-3.43032000
C	-8.17755200	-1.09910300	-3.75029900
H	-7.80097800	-0.10573800	-3.47936600
H	-8.31584200	-1.15294600	-4.83091100
H	-9.13679000	-1.27473000	-3.24925800
O	-0.68805000	-1.14789600	3.70366700
O	-5.49068800	-4.21857400	-2.76796300
C	-5.48441200	2.87966700	2.11061800
O	-4.92020300	4.10540200	2.29516800
C	-3.99653100	4.26463600	3.36763000
H	-3.13672900	3.59253500	3.25844800
H	-3.65653800	5.29997500	3.31457300
H	-4.47394200	4.08802700	4.33934600
C	5.70227700	3.61064500	-1.55921100
H	6.56437900	3.43565100	-2.21993000
C	6.81763300	-1.79152100	0.15665100
H	6.18940400	-2.04106200	1.01389800
C	8.15509700	-1.24509600	0.67581400
H	8.82266000	-0.99579700	-0.15243900
H	8.64627300	-2.01902500	1.28211600
O	8.00329100	-0.04481800	1.43559800
C	4.76192600	1.38627500	-1.63371500
C	3.62599000	0.60581100	-0.93941900
H	4.00357600	0.08864300	-0.05971000
H	2.86746700	1.33456800	-0.63765200
C	4.43280400	3.61790400	-2.41244400
H	4.61790100	4.05651100	-3.39949100
O	3.04742500	-0.40478600	-1.76517900
C	3.23250100	4.35520900	-1.82534300
H	2.37213200	4.21603300	-2.49626100
H	3.46798100	5.42907300	-1.79108400
O	2.93989800	3.87392800	-0.52125300
O	-6.63113200	3.83095400	0.20523600
C	-5.97428900	3.81580300	-1.08926300
H	-6.10154300	2.82819300	-1.54759900
H	-6.52711500	4.54981900	-1.68144200
C	-4.85218600	-3.72609900	-3.97542100
H	-5.58795800	-3.20484900	-4.59038300
H	-4.55074200	-4.64422000	-4.48523000
C	0.05321900	-1.96349900	4.63157500
H	-0.23709700	-1.67169000	5.65181400
H	-0.20823300	-3.01657900	4.49866700
C	7.72611200	-0.19731400	2.83871200
H	8.14238700	0.70525100	3.29981000
H	8.27559900	-1.06224700	3.23153300
C	2.17152000	0.05833100	-2.81229600
H	1.83833600	1.07602800	-2.59465700
H	2.74121400	0.09952300	-3.75019100
C	1.94494400	4.61965400	0.17636900

H	2.10910700	4.38135700	1.23417300
H	2.11638700	5.69812600	0.04916200
C	6.25224300	-0.31205600	3.16501000
C	5.73069100	-1.43675300	3.77062400
C	5.37944400	0.76335800	2.83604300
C	4.33882200	-1.55805700	4.03581400
H	6.38557500	-2.26308500	4.04037400
C	4.02731000	0.67663300	3.07149200
H	5.79607900	1.66150900	2.38715800
C	3.77645800	-2.71748900	4.63405500
C	3.46249200	-0.48988400	3.65949500
H	3.37032200	1.50170100	2.80726500
C	2.41707900	-2.82532100	4.81919400
H	4.43492700	-3.53208000	4.92536500
C	2.06490500	-0.63084700	3.86931100
C	1.53857600	-1.78001800	4.42354000
H	2.00264500	-3.72919800	5.25937500
H	1.40335000	0.17328800	3.56122800
C	0.97962300	-0.85913200	-2.94653500
C	-0.28207100	-0.32225400	-3.09811700
C	1.13847300	-2.27355400	-2.97144900
C	-1.42297100	-1.14551600	-3.29474200
H	-0.41976200	0.75655500	-3.07392900
C	0.05230700	-3.10096300	-3.14324000
H	2.13117200	-2.69842400	-2.85307200
C	-2.72283400	-0.60529300	-3.48575800
C	-1.25686800	-2.56812500	-3.31597800
H	0.18462500	-4.18009900	-3.16080700
C	-3.80301500	-1.43071300	-3.69987400
H	-2.85005500	0.47452200	-3.47348600
C	-2.39582900	-3.38921000	-3.52816600
C	-3.65123500	-2.84448600	-3.72512100
H	-4.78867000	-1.00599500	-3.86724200
H	-2.26327500	-4.46922000	-3.55164600
C	0.51976300	4.26558700	-0.20250100
C	-0.43438500	5.24474900	-0.38976500
C	0.13240200	2.89894600	-0.28384900
C	-1.79839900	4.91672100	-0.62374100
H	-0.15454800	6.29504400	-0.33321800
C	-1.17203700	2.54549500	-0.53669300
H	0.88015000	2.12795800	-0.12787300
C	-2.80898100	5.90773100	-0.76309300
C	-2.17711900	3.53789900	-0.70069800
H	-1.45528300	1.49729500	-0.59072000
C	-4.12675100	5.55004100	-0.93237300
H	-2.52575400	6.95662800	-0.71868200
C	-3.54265100	3.20561000	-0.90326900
C	-4.51361500	4.18296400	-0.99585300
H	-4.89078000	6.31919100	-1.01651300
H	-3.81989200	2.15521300	-0.96051200

Imaginary Frequency = 0

E(RB3LYP) = -4064.5224152 a.u.

Conformation #2 (Pop. = 40.31%)

Symbolic Z-matrix:

C	-7.28565600	2.80017600	-2.15052900
C	-6.36295200	1.70456900	-2.71924400
C	-6.46544300	0.45336100	-1.84150400
C	-6.92819300	3.07877600	-0.67757700
O	-6.06490800	0.76104600	-0.51519000
O	-8.65158600	2.39592500	-2.20225600
O	-5.02551800	2.20834000	-2.72495100
O	-5.59203100	-0.50631400	-2.40734700
O	-5.71554100	3.82174600	-0.61373400
C	-5.55854100	-2.35126200	-0.70111500
O	-4.16197800	-2.21654300	-2.62681200
O	-6.85744300	-1.95893300	-0.31142500
O	-5.86723300	-4.81644500	-0.84699800
H	-6.67492600	1.44175800	-3.74133000
H	-4.93129200	-2.58935700	0.16667200
H	-7.12738100	3.73050000	-2.71209300
H	-7.71054500	3.71239500	-0.24485800
O	0.87072900	3.54757700	2.15640700
C	6.36112800	-2.74627600	1.02811400
C	7.01506100	-1.53756200	0.82274300
C	6.81284100	-0.41453500	1.64221600
H	7.72436100	-1.48428800	0.00057200
C	5.26884100	-1.76384600	2.93925200
C	5.90474100	-0.53054300	2.71016800
H	4.59762700	-1.84663900	3.78667400
C	6.88060100	2.27866000	-2.12429200
C	5.97779000	3.32651100	-1.83834600
C	7.36815100	1.50737400	-1.06873900
C	5.64662400	3.57458600	-0.51014500
C	7.01852300	1.75289800	0.27036000
H	8.06696200	0.70573200	-1.27944900
C	6.15296600	2.82113200	0.56003600
H	4.98009600	4.41120900	-0.31967700
C	1.99208400	2.82163900	2.49948300
C	1.90567000	1.66465100	3.29404400
C	3.24639600	3.27976600	2.11186300
C	3.07637800	0.99451000	3.63941200
C	4.43174900	2.61311300	2.45697700
H	3.28648500	4.19332900	1.52497300
C	4.34462600	1.43541000	3.22646000
C	7.62024700	0.84518100	1.33806100
H	8.61030300	0.52857100	0.99079900
H	7.79821900	1.40928400	2.25663900
C	5.74566400	3.21923400	1.97500400
H	5.63597800	4.30922700	1.99642100
H	6.54989400	2.99034400	2.67761600
H	2.98362300	0.10760200	4.26033500
C	5.56117000	0.61791300	3.65604900
H	5.34889100	0.19157300	4.64255300
H	6.42653700	1.26977600	3.79489600
H	-7.48669200	0.05896000	-1.85165800
H	-5.11022700	3.37023100	-1.23903300

H	-8.90217400	2.29178900	-3.13480000
H	-4.41137100	1.47360900	-2.48877600
H	-6.83089800	-1.17961800	0.27781700
H	-6.69637700	-4.73422400	-0.34788500
C	0.06664100	2.95176100	1.12561700
H	0.63730800	2.86306300	0.19337800
H	-0.29998800	1.96690300	1.43036800
H	-0.77893100	3.62529400	0.97171700
O	7.23568600	2.10449000	-3.43423800
C	8.17874800	1.08230900	-3.75098100
H	7.80102700	0.09015300	-3.47723400
H	8.31736400	1.13282500	-4.83171200
H	9.13805500	1.25835700	-3.25021900
O	0.68808100	1.15984500	3.69916600
O	5.49541600	4.20794500	-2.77838700
C	5.48072900	-2.87598900	2.12195300
O	4.91515500	-4.10055200	2.31015900
C	3.99152800	-4.25556800	3.38328600
H	3.13236200	-3.58296300	3.27220900
H	3.65050800	-5.29073200	3.33350600
H	4.46930500	-4.07642100	4.35435700
C	-5.70555000	-3.60802900	-1.56516000
H	-6.56756700	-3.43377300	-2.22622800
C	-6.81938500	1.78795200	0.16430600
H	-6.19280200	2.03469000	1.02351700
C	-8.15636000	1.23768800	0.68050400
H	-8.82341200	0.98713800	-0.14744600
H	-8.64982400	2.00909100	1.28792700
O	-8.00071900	0.03628000	1.43858200
C	-4.76579300	-1.38279900	-1.63780400
C	-3.62764800	-0.60603300	-0.94303500
H	-4.00360300	-0.08843400	-0.06290600
H	-2.87104600	-1.33702800	-0.64204400
C	-4.43599000	-3.61375300	-2.41807500
H	-4.62003700	-4.05324100	-3.40492300
O	-3.04646100	0.40360000	-1.76835600
C	-3.23472600	-4.34910900	-1.83031600
H	-2.37409900	-4.20844500	-2.50058800
H	-3.46865600	-5.42332500	-1.79658700
O	-2.94379900	-3.86795400	-0.52579900
O	6.62707200	-3.83457000	0.22010200
C	5.97198100	-3.82238100	-1.07528300
H	6.10098700	-2.83624200	-1.53628100
H	6.52476000	-4.55870800	-1.66463000
C	4.85679100	3.71287100	-3.98469300
H	5.59219700	3.18918500	-4.59801800
H	4.55647100	4.62992800	-4.49707600
C	-0.05377200	1.97811100	4.62402900
H	0.23935600	1.69314500	5.64542000
H	0.20393600	3.03130400	4.48460700
C	-7.72317600	0.18706600	2.84176500
H	-8.13636400	-0.71760600	3.30153800
H	-8.27513700	1.04958700	3.23639500
C	-2.17142700	-0.06113000	-2.81549200
H	-1.83933900	-1.07906100	-2.59725900
H	-2.74135900	-0.10229200	-3.75324200
C	-1.95128800	-4.61472700	0.17420800
H	-2.11744000	-4.37577800	1.23155600

H	-2.12398200	-5.69300100	0.04705700
C	-6.24944300	0.30590400	3.16729800
C	-5.73094000	1.43124400	3.77438200
C	-5.37341400	-0.76585600	2.83516900
C	-4.33909700	1.55697700	4.03756000
H	-6.38828400	2.25486900	4.04640900
C	-4.02123900	-0.67486500	3.06877800
H	-5.78755300	-1.66468500	2.38532400
C	-3.77976900	2.71748300	4.63665800
C	-3.45962100	0.49264500	3.65781700
H	-3.36174900	-1.49712600	2.80203600
C	-2.42044000	2.83007900	4.81910000
H	-4.44065500	3.52915000	4.93063100
C	-2.06210200	0.63843500	3.86494600
C	-1.53889500	1.78870400	4.41973700
H	-2.00842200	3.73480800	5.25982000
H	-1.39820100	-0.16266500	3.55410400
C	-0.97848100	0.85480200	-2.95072700
C	0.28264700	0.31618100	-3.10084300
C	-1.13568700	2.26936600	-2.97840900
C	1.42455800	1.13772200	-3.29877100
H	0.41908300	-0.76273400	-3.07450000
C	-0.04851000	3.09516300	-3.15158400
H	-2.12790000	2.69567200	-2.86106100
C	2.72383500	0.59559500	-3.48834200
C	1.26008900	2.56046900	-3.32299700
H	-0.17957300	4.17441400	-3.17131000
C	3.80499700	1.41931500	-3.70404700
H	2.84981800	-0.48433500	-3.47367200
C	2.40001800	3.37979100	-3.53680300
C	3.65481900	2.83319500	-3.73244100
H	4.79020900	0.99312200	-3.87026500
H	2.26871800	4.45989900	-3.56263400
C	-0.52472300	-4.26287200	-0.20159800
C	0.42882100	-5.24351300	-0.38423100
C	-0.13550700	-2.89686000	-0.28455700
C	1.79383100	-4.91754300	-0.61527100
H	0.14762600	-6.29336800	-0.32627600
C	1.17000600	-2.54544500	-0.53471900
H	-0.88269600	-2.12464600	-0.13208500
C	2.80363500	-5.90998600	-0.75001600
C	2.17433800	-3.53932700	-0.69421100
H	1.45459700	-1.49768400	-0.59011500
C	4.12218200	-5.55415100	-0.91718400
H	2.51914300	-6.95846600	-0.70389400
C	3.54068600	-3.20901700	-0.89453900
C	4.51074000	-4.18765700	-0.98305000
H	4.88551500	-6.32435300	-0.99795200
H	3.81920000	-2.15905900	-0.95362000

Imaginary Frequency = 0

E(RB3LYP) = -4064.5221581 a.u

***M*-5a⊂Ach**

Symbolic Z-matrix:

C	-5.498030	-0.383334	2.720378
C	-6.721538	-1.171521	2.276302
C	-6.476420	-1.690676	0.852081
C	-4.139629	-2.006684	1.314958
C	-4.270402	-1.284733	2.677047
O	-5.380700	-2.580580	0.882503
O	-5.767596	0.094652	4.040015
O	-6.246423	-0.562649	0.035516
C	-3.186685	-3.197706	1.423700
O	-1.934671	-2.737743	1.925576
C	-7.119786	1.896969	-1.579752
C	-8.307092	1.411306	-0.752283
C	-6.860678	-0.428615	-1.251027
O	-6.305332	0.728763	-1.834351
C	-6.564971	-1.619343	-2.160904
O	-5.171807	-1.828898	-2.225398
H	-6.857885	-2.039497	2.936833
H	-8.088601	1.512242	0.319038
H	-7.500504	2.266142	-2.542050
H	-5.354235	0.458284	2.031573
H	-4.389310	-2.044908	3.467639
H	-3.784296	-1.288918	0.566108
H	-3.631494	-3.929006	2.115449
H	-3.059476	-3.678850	0.445799
H	-6.982059	-1.397765	-3.156605
H	-7.080087	-2.515630	-1.777361
C	4.613090	-3.415787	-1.837405
C	4.839327	-2.307300	-2.648422
C	5.419758	-3.585065	-0.687776
C	5.775671	-1.309357	-2.335551
H	4.258634	-2.243205	-3.565113
C	6.314541	-2.572099	-0.345553
C	6.493100	-1.423063	-1.134346
H	6.909184	-2.673439	0.555386
C	2.976600	1.857785	-4.455871
C	3.110134	3.050829	-3.707124
C	3.907332	0.840480	-4.272388
C	4.128421	3.137108	-2.762558
C	4.968267	0.945810	-3.353250
H	3.843784	-0.052798	-4.884371
C	5.069755	2.104877	-2.566361
H	4.247048	4.051330	-2.190286
C	5.300601	2.317419	2.138257
C	5.932049	1.135220	2.596697
C	5.384577	2.635419	0.778087
C	6.588037	0.340462	1.660540
C	6.072958	1.837103	-0.156879
H	4.951678	3.569372	0.434410
C	6.681980	0.655029	0.297808
O	6.073640	0.747293	3.896574
O	2.253927	4.053641	-4.067449
C	1.999584	5.161150	-3.213292
H	2.898151	5.471855	-2.666095

H	1.754752	5.980420	-3.899860
C	5.994369	-0.181710	-3.338334
H	5.999364	-0.630900	-4.338542
H	6.994648	0.238219	-3.207092
C	6.212866	2.362409	-1.586065
H	6.346027	3.448825	-1.521003
H	7.144986	1.986454	-2.017829
H	7.073637	-0.551812	2.045499
C	7.416997	-0.335130	-0.601197
H	8.210195	-0.808829	-0.012011
H	7.924122	0.182267	-1.419124
H	-7.321021	-2.284039	0.498357
H	-4.957486	0.536315	4.342315
O	3.674042	-4.322712	-2.257231
O	-5.748303	2.546968	0.275482
O	-9.471430	2.139490	-1.109516
H	-10.211083	1.733043	-0.629292
C	2.793989	-4.914081	-1.287355
H	3.066065	-5.968778	-1.169036
H	2.941303	-4.435025	-0.313621
C	5.034375	0.895945	4.894605
H	4.834911	1.953536	5.077993
H	5.526694	0.480050	5.782138
O	5.271240	-4.752838	0.008418
O	4.683534	3.094266	3.073573
O	1.934669	1.822410	-5.336288
C	1.809808	0.688092	-6.178542
H	1.650385	-0.233135	-5.600689
H	0.935413	0.876312	-6.803747
H	2.693483	0.560669	-6.817355
C	6.105680	-4.977214	1.136312
H	5.923244	-4.239077	1.927801
H	5.845208	-5.970959	1.504347
H	7.167129	-4.957492	0.858207
C	4.034939	4.291148	2.660034
H	3.285939	4.097303	1.884808
H	3.517173	4.671137	3.540710
H	4.763820	5.028649	2.297873
C	-6.288691	3.005914	-0.952844
H	-6.938002	3.882946	-0.791985
H	-5.491279	3.296407	-1.648513
C	-1.192013	-3.735262	2.671055
H	-1.821429	-4.048587	3.521233
H	-1.022900	-4.613567	2.032982
C	-4.750740	3.375452	0.842015
H	-4.549511	2.929112	1.824061
H	-5.138313	4.392735	1.019828
C	0.107374	-3.133678	3.108008
C	0.110771	-1.947040	3.872092
C	1.329752	-3.678961	2.695150
C	1.311860	-1.253683	4.162466
H	-0.828254	-1.532145	4.234946
C	2.539172	-3.055681	3.007502
H	1.332412	-4.596238	2.108451
C	1.339992	-0.000454	4.834890
C	2.570066	-1.813542	3.715777
H	3.480824	-3.507921	2.702228
C	2.532845	0.696778	5.019795

H	0.403080	0.418099	5.198555
C	3.762610	-1.097663	3.964679
C	3.755419	0.158524	4.593579
H	2.514860	1.672029	5.501816
H	4.708140	-1.526340	3.640689
C	-3.469355	3.458210	0.026627
C	-2.624061	4.546147	0.169250
C	-3.129490	2.429432	-0.888072
C	-1.435170	4.670604	-0.596591
H	-2.878932	5.346326	0.863026
C	-2.001465	2.542131	-1.678512
H	-3.795714	1.578115	-0.995821
C	-0.547073	5.771902	-0.459708
C	-1.134218	3.665013	-1.574544
H	-1.782255	1.775987	-2.419584
C	0.566085	5.888022	-1.264538
H	-0.759912	6.527922	0.292262
C	0.000126	3.836535	-2.413934
C	0.839529	4.924189	-2.267345
H	1.232695	6.739754	-1.143449
H	0.192961	3.114516	-3.202628
C	-3.336987	-3.173571	-2.945324
C	-2.843894	-4.281018	-2.278509
C	-2.421824	-2.243872	-3.500125
C	-1.450737	-4.490142	-2.106547
H	-3.534504	-5.007430	-1.854554
C	-1.060345	-2.423676	-3.366164
H	-2.808198	-1.376696	-4.029794
C	-0.930975	-5.601426	-1.390732
C	-0.530058	-3.534616	-2.652021
H	-0.369590	-1.705475	-3.805075
C	0.425868	-5.738528	-1.184585
H	-1.622482	-6.343648	-0.998869
C	0.862908	-3.715243	-2.430858
C	1.341939	-4.782399	-1.688191
H	0.803748	-6.591916	-0.625434
H	1.563687	-3.004152	-2.861520
C	-4.821522	-2.935894	-3.050633
H	-5.113545	-2.725230	-4.094121
H	-5.370902	-3.835784	-2.731397
O	-9.134685	-0.848917	-0.202227
H	-8.860876	-0.608527	0.708009
O	-3.130683	-0.469204	2.944976
H	-2.364370	-1.062808	2.813709
O	-7.899538	-0.368607	2.298839
H	-7.837090	0.196453	3.087846
C	-8.368488	-0.079997	-1.100951
H	-8.844915	-0.184270	-2.083972
C	1.717219	0.983690	1.285769
H	2.060367	0.416960	2.152248
H	2.509072	1.673846	0.988041
C	0.454828	1.765891	1.654711
H	-0.059807	2.188757	0.784649
H	-0.241296	1.130629	2.205924
O	0.951109	2.829915	2.472804
C	1.450950	0.732519	-1.170999
H	1.334456	-0.004607	-1.966371
H	0.586979	1.394842	-1.153173

H	2.364483	1.306686	-1.328922
C	2.783884	-0.869914	0.107578
H	2.798091	-1.482120	1.010502
H	2.733759	-1.502060	-0.778435
H	3.666413	-0.230915	0.058551
C	0.335346	-0.871269	0.330693
H	0.343529	-1.645142	-0.438494
H	0.371735	-1.327479	1.319429
H	-0.561888	-0.260145	0.225509
N	1.552279	0.001810	0.138232
C	0.181167	3.458798	3.411951
C	-1.260530	3.024335	3.560780
H	-1.797481	3.108683	2.609393
H	-1.339226	1.983680	3.893731
H	-1.727952	3.671981	4.303105
O	0.695262	4.337190	4.060599

Imaginary Frequency = 0

E(RB3LYP) = -4545.97271 a.u.

***M*-5a⊂Ch**

Symbolic Z-matrix:

C	-5.304476	-1.134014	2.743393
C	-6.550413	-1.748385	2.116832
C	-6.360365	-1.838035	0.594812
C	-4.006441	-2.244373	0.873044
C	-4.105799	-2.005821	2.395686
O	-5.256852	-2.685162	0.332367
O	-5.550825	-1.075760	4.148602
O	-6.177658	-0.526475	0.113753
C	-3.024104	-3.375663	0.558997
O	-1.750371	-3.064488	1.132626
C	-7.176336	2.263488	-0.752640
C	-8.288104	1.546889	0.007653
C	-6.872836	-0.059917	-1.053641
O	-6.358267	1.221744	-1.335338
C	-6.639448	-0.959121	-2.266408
O	-5.256321	-1.145484	-2.456838
H	-6.678084	-2.770228	2.501863
H	-7.980727	1.363366	1.046011
H	-7.638585	2.837689	-1.567828
H	-5.163156	-0.125003	2.333409
H	-4.239971	-2.975929	2.899249
H	-3.698381	-1.313263	0.381348
H	-3.419727	-4.303632	0.997845
H	-2.934957	-3.512970	-0.524382
H	-7.103174	-0.472064	-3.139532
H	-7.150902	-1.924829	-2.117013
C	4.489213	-2.682073	-2.609511
C	4.673800	-1.372831	-3.043168
C	5.370782	-3.202235	-1.635283
C	5.663473	-0.528040	-2.517813
H	4.029418	-1.026390	-3.847246
C	6.323753	-2.353266	-1.075131
C	6.476664	-1.018675	-1.484679
H	6.984500	-2.733124	-0.303972

C	2.935255	3.275551	-3.407291
C	3.232035	4.222626	-2.401278
C	3.777891	2.179027	-3.568269
C	4.305371	3.979046	-1.549059
C	4.902617	1.966777	-2.749665
H	3.598788	1.487251	-4.384328
C	5.151137	2.861334	-1.694699
H	4.549751	4.711240	-0.785481
C	5.634017	1.577070	2.804968
C	6.228409	0.293665	2.839029
C	5.660055	2.300069	1.608529
C	6.802357	-0.187199	1.666825
C	6.267882	1.814406	0.434864
H	5.245173	3.302915	1.590589
C	6.845844	0.534787	0.467300
O	6.415913	-0.476280	3.949886
O	2.491974	5.374535	-2.443643
C	2.233479	6.137386	-1.272460
H	3.086974	6.129765	-0.582752
H	2.131404	7.168184	-1.632766
C	5.854813	0.844996	-3.150677
H	5.758011	0.716382	-4.235340
H	6.883761	1.178618	-2.994387
C	6.360852	2.754355	-0.768637
H	6.564534	3.759793	-0.381233
H	7.243330	2.493021	-1.360347
H	7.262358	-1.169340	1.725420
C	7.481873	-0.155521	-0.734200
H	8.293644	-0.796825	-0.372993
H	7.951590	0.570500	-1.402172
H	-7.213805	-2.329094	0.125215
H	-4.765131	-0.687115	4.565001
O	3.520487	-3.423206	-3.234243
O	-5.617615	2.490390	1.051420
O	-9.480451	2.313290	-0.048144
H	-10.180532	1.766703	0.344393
C	2.671051	-4.263148	-2.437574
H	2.970554	-5.307595	-2.579444
H	2.813171	-4.027902	-1.376393
C	5.392575	-0.671850	4.957424
H	5.209705	0.263257	5.492165
H	5.894487	-1.365328	5.643078
O	5.233349	-4.526767	-1.322176
O	5.116556	2.051923	3.980849
O	1.843666	3.547030	-4.181952
C	1.564326	2.681660	-5.270334
H	1.355729	1.656710	-4.932724
H	0.675348	3.089325	-5.754554
H	2.392621	2.659013	-5.990307
C	6.150712	-5.099296	-0.401769
H	6.064133	-4.643487	0.592885
H	5.885575	-6.155993	-0.336844
H	7.185045	-5.003614	-0.756552
C	4.556981	3.352204	3.997564
H	3.700189	3.435756	3.314244
H	4.215435	3.518216	5.020905
H	5.300361	4.116602	3.734161
C	-6.326548	3.222274	0.067170

H	-6.994203	3.966981	0.532924
H	-5.636410	3.759001	-0.596531
C	-0.964299	-4.241161	1.485265
H	-1.569203	-4.824672	2.200074
H	-0.817363	-4.851190	0.584944
C	-4.660255	3.238461	1.776415
H	-4.323514	2.569076	2.577594
H	-5.128765	4.114827	2.255470
C	0.347724	-3.810810	2.061279
C	0.390872	-2.921277	3.155640
C	1.557496	-4.260161	1.509937
C	1.615269	-2.392550	3.636481
H	-0.530365	-2.608921	3.645012
C	2.785125	-3.825113	2.008808
H	1.533394	-4.958712	0.675106
C	1.673555	-1.371928	4.624179
C	2.855849	-2.850480	3.052719
H	3.709995	-4.194268	1.571579
C	2.886755	-0.801473	4.998441
H	0.743912	-1.009157	5.057745
C	4.070573	-2.283583	3.499249
C	4.098100	-1.251028	4.451502
H	2.898838	0.000519	5.734489
H	5.004119	-2.637954	3.068069
C	-3.450777	3.682199	0.968442
C	-2.604202	4.645664	1.488239
C	-3.136467	3.080272	-0.275309
C	-1.411960	5.024903	0.821590
H	-2.834743	5.112195	2.444911
C	-2.009675	3.467558	-0.976625
H	-3.797119	2.314727	-0.672610
C	-0.490544	5.957295	1.372620
C	-1.111564	4.436039	-0.451731
H	-1.794859	3.027268	-1.948393
C	0.673405	6.278105	0.708802
H	-0.711617	6.403517	2.339365
C	0.078825	4.819923	-1.128409
C	0.967922	5.709530	-0.556830
H	1.373318	6.982253	1.155235
H	0.291838	4.402927	-2.108725
C	-3.476018	-2.235159	-3.597706
C	-2.973736	-3.477355	-3.247852
C	-2.571098	-1.191342	-3.910439
C	-1.578130	-3.716612	-3.148687
H	-3.659017	-4.289975	-3.013958
C	-1.207245	-1.392639	-3.841278
H	-2.965910	-0.219175	-4.195135
C	-1.044759	-4.967796	-2.737958
C	-0.667250	-2.645445	-3.435103
H	-0.521962	-0.584487	-4.091295
C	0.314456	-5.134718	-2.562657
H	-1.727079	-5.793872	-2.550954
C	0.725153	-2.860018	-3.262455
C	1.216873	-4.069775	-2.801262
H	0.703063	-6.097139	-2.236014
H	1.417440	-2.052883	-3.486511
C	-4.958291	-1.970008	-3.581823
H	-5.282621	-1.462222	-4.506399

H	-5.514103	-2.918714	-3.514278
O	-9.080949	-0.793936	-0.033917
H	-8.760994	-0.804977	0.892485
O	-2.917465	-1.381970	2.896292
H	-2.183667	-1.922076	2.527232
O	-7.711153	-0.979743	2.415359
H	-7.633691	-0.709346	3.346590
C	-8.369360	0.205460	-0.726738
H	-8.901840	0.364415	-1.673144
C	0.267284	0.358419	1.820783
H	0.032426	-0.682131	2.056664
H	0.980682	0.709379	2.569861
C	-1.009273	1.214830	1.853790
H	-0.782854	2.273105	1.702954
H	-1.708503	0.912060	1.065555
O	-1.599713	1.095059	3.132058
H	-2.143367	0.279876	3.123937
C	1.539293	1.670393	0.111928
H	2.069619	1.590268	-0.838615
H	0.715204	2.375506	0.017078
H	2.234293	1.997748	0.886776
C	2.181354	-0.632571	0.623869
H	1.803704	-1.638414	0.801183
H	2.759361	-0.600374	-0.301960
H	2.800738	-0.318576	1.464260
C	0.107790	-0.206638	-0.599379
H	0.711952	-0.405361	-1.484670
H	-0.355585	-1.133197	-0.254372
H	-0.652686	0.539503	-0.827940
N	1.012651	0.314164	0.487669

Imaginary Frequency = 0

E(RB3LYP) = -4393.317892 a.u.
